# Rhodium-catalyzed asymmetric 1,4-addition reactions of aryl boronic acids with nitroalkenes: reaction mechanism and development of homogeneous and heterogeneous catalysts†
†Electronic supplementary information (ESI) available. See DOI: 10.1039/c7sc03025h


**DOI:** 10.1039/c7sc03025h

**Published:** 2017-10-11

**Authors:** Hiroyuki Miyamura, Kohei Nishino, Tomohiro Yasukawa, Shū Kobayashi

**Affiliations:** a Department of Chemistry , School of Science , The University of Tokyo , Hongo, Bunkyo-ku , Tokyo 113-0033 , Japan . Email: shu_kobayshi@chem.s.u-tokyo.ac.jp

## Abstract

Asymmetric 1,4-addition reactions with nitroalkenes are valuable because the resulting chiral nitro compounds can be converted into various useful species often used as chiral building blocks in drug and natural product synthesis. In the present work, asymmetric 1,4-addition reactions of arylboronic acids with nitroalkenes catalyzed by a rhodium complex with a chiral diene bearing a tertiary butyl amide moiety were developed. Just 0.1 mol% of the chiral rhodium complex could catalyze the reactions and give the desired products in high yields with excellent enantioselectivities. The homogeneous catalyst thus developed could be converted to a reusable heterogeneous metal nanoparticle system using the same chiral ligand as a modifier, which was immobilized using a polystyrene-derived polymer with cross-linking moieties, maintaining the same level of enantioselectivity. To our knowledge, this is the first example of asymmetric 1,4-addition reactions of arylboronic acids with nitroalkenes in a heterogeneous system. Wide substrate generality and high catalytic turnover were achieved in the presence of sufficient water without any additives such as KOH or KHF_2_ in both homogeneous and heterogeneous systems. Various insights relating to a rate-limiting step in the catalytic cycle, the importance of water, role of the secondary amide moiety in the ligand, and active species in the heterogeneous system were obtained through mechanistic studies.

## Abstract 2

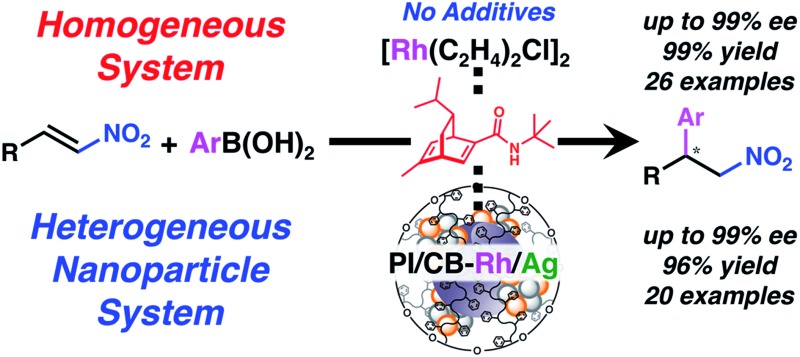
Asymmetric 1,4-additions of arylboronic acids with nitroalkenes catalyzed by rhodium complexes or heterogeneous Rh–Ag bimetallic nanoparticles with a chiral diene ligand bearing a tertiary butyl amide moiety are developed.

## Introduction

Asymmetric synthesis is crucial in fine chemical production, such as the synthesis of drugs, biologically active compounds, and natural products. In particular, asymmetric carbon–carbon bond formation is useful because both chiral centers and carbon-based skeletons of target molecules are constructed simultaneously. Asymmetric 1,4-addition reactions with nitroalkenes are valuable because the resulting chiral nitro compounds can be converted into various useful species often used as chiral building blocks in drug and natural product synthesis. The first rhodium-catalyzed asymmetric 1,4-addition reaction of aryl boronic acids with nitroalkenes was reported by Hayashi *et al.* and several homogeneous systems for this transformation have been developed using chiral rhodium and palladium complexes.^1^–^12^ In the rhodium-catalyzed 1,4-addition reaction of aryl boronic acids with nitroalkenes, it was suggested that the catalyst regeneration step is relatively slow because of the strong coordination ability of the generated nitronate to the rhodium complex. This slow catalytic regeneration often caused low catalytic turnover and by-product formation, and then, in most previously reported rhodium-catalyzed reactions, stoichiometric to substoichiometric additives, such as KOH or KHF_2_, were required to achieve an efficient catalytic turnover.^2^–^6^,^9^–^11^

In contrast, to our knowledge heterogeneous catalyst systems for this asymmetric reaction have never been developed hitherto, probably because the strong coordination ability of the nitro group can induce serious metal leaching and poisoning of the catalytically active center, which might be generally less robust compared with that of corresponding homogeneous complexes. Heterogeneous catalysts have attracted great attention because of their facile separation from products, reusability, and application to industrial processes.^13^–^17^ As general strategies to develop heterogeneous metal catalysts for asymmetric transformations, immobilized chiral ligands to form immobilized chiral metal complexes have been widely explored.^18^–^21^ However, these strategies suffer from complicated preparation of immobilized ligands, metal leaching during asymmetric reaction, and often instability of immobilized ligands. In addition, immobilized metal catalysts for organic transformations are generally less active than the corresponding homogeneous catalysts, and they often suffer from serious leaching of metals during reactions and decreased activity during recovery and reuse. As a strategy to convert homogeneous catalysis to heterogeneous catalysis, the use of heterogeneous metal nanoparticle catalysts is of great interest because of their reusability, robustness and high reactivity.^22^–^25^ Recently, several organic transformations originally catalyzed by homogeneous catalysts have been successfully converted to heterogeneous metal nanoparticle catalysis.^26^,^27^ Asymmetric organic synthesis with heterogeneous metal nanoparticle systems using chiral ligands as “chiral modifiers” have been investigated, especially in asymmetric hydrogenation reactions pioneered by Orito *et al.*^28^,^29^ Several mechanistic studies, even including theoretical calculations, have been conducted for Orito-type reactions.^30^–^35^ In contrast, asymmetric C–C bond formation reactions using heterogeneous metal nanoparticle systems is a very limited and developing field, and their reaction mechanisms are obscure in contrast to matured asymmetric hydrogenation reactions.^27^,^36^–^43^

**Fig. 1:**
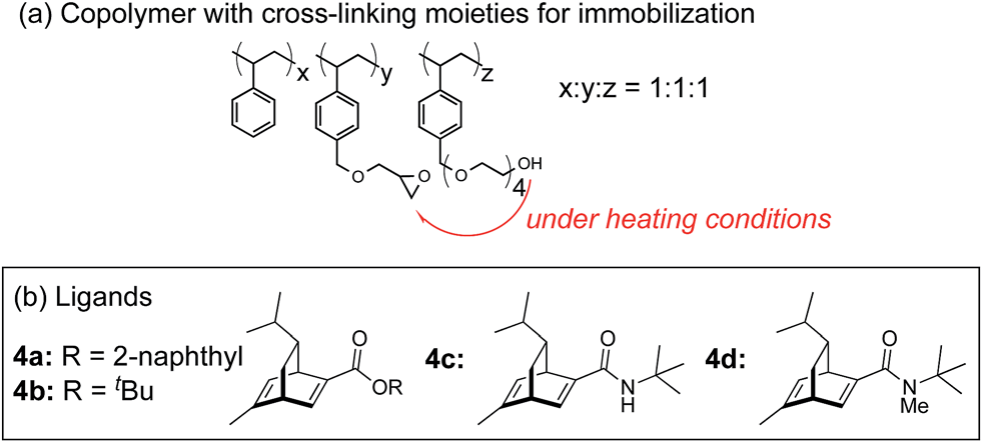
Copolymer for immobilization and chiral dienes.

In this context, we have developed chirally modified rhodium and bimetallic nanoparticle systems based on a polymer-incarcerated (PI) strategy, in which polystyrene derivatives with cross-linking moieties (Fig. 1a) are used to immobilize metal nanoparticles.^44^–^48^ The metal nanoparticles are encapsulated and stabilized with weak, but multiple interactions of benzene rings in the polymer and physical entrapment by cross-linked polymer cage.^49^ The chirally modified rhodium-based metal nanoparticle systems thus developed can be applied to asymmetric 1,4-addition reactions of aryl boronic acids with α,β-unsaturated carbonyl compounds, such as ketones, esters and amides. In addition, we have newly developed a chiral diene ligand, including a secondary amide moiety with bifunctionality, interacting with a metal nanoparticle through a diene moiety and activating a substrate through hydrogen bonding (ligand **4c** in Fig. 1b).^45^,^50^ In the course of these studies, we obtained insight into the reaction mechanism of chirally modified metal nanoparticle systems, in which active species in heterogeneous systems are different from those in corresponding homogeneous systems, and a direct interaction between the chiral ligand and metal nanoparticle surface was proven.^45^,^51^

In the present article, we report the development of asymmetric 1,4-addition reactions of aryl boronic acids with nitroalkenes with a high catalytic turnover and excellent enantioselectivity using both homogeneous metal complex and heterogeneous metal nanoparticle systems of chiral diene ligand **4c** without any additives, such as KOH and KHF_2_. This is the first heterogeneous system for this asymmetric transformation, and we have also found various insights into the reaction mechanism, especially that are different from asymmetric 1,4-addition reactions with α,β-unsaturated carbonyl compounds.

## Results and discussion

### Development of homogeneous reaction systems

A family of chiral dienes with bicyclo[2,2,2]hexadiene structure were developed independently by Hayashi *et al.* and Carreira *et al.* They have been widely used for transition metal-catalyzed asymmetric reactions, particularly Rh-catalyzed asymmetric 1,4-addition-type reactions.^52^–^55^ We previously designed and developed chiral diene ligands containing secondary amide functionality as highly efficient and selective bifunctional ligands for asymmetric 1,4-additions of α,β-unsaturated carbonyl compounds in both homogeneous and heterogeneous systems.^45^ In this context, we initiated an investigation of the asymmetric 1,4-addition reaction of 3-methoxy phenyl boronic acid (**2a**) with nitrostyrene (**1a**) using homogeneous systems of 0.05 mol% of [Rh(C_2_H_4_)_2_Cl]_2_ (0.1 mol% of Rh) and different types of chiral diene ligands at 100 °C in a 2 : 1 ratio of toluene and water solvents (Table 1). Chiral diene ligands with bulky ester moieties (**4a** and **4b**) gave the desired product **3aa** in high yields with good enantioselectivities (entries 1 and 2). The diene ligand with secondary bulky amide **4c** gave **3aa** with excellent enantioselectivity, even in moderate yield (entry 3). In contrast, the enantioselectivity was dramatically reduced with chiral diene ligand **4d** containing a tertiary amide moiety, although an excellent yield was observed (entry 4). We determined ligand **4c** to be the best in terms of enantioselectivity. Next, we investigated the solvent system using phenyl boronic acid (**2b**) as the substrate (Table 2). Phenyl boronic acid (**2b**) gave a higher yield under the same reaction conditions of Table 1, entry 3, obtaining an excellent yield (entry 1). When a 1 : 1 ratio of toluene and water maintaining the same concentration was employed, the yield was improved slightly (entry 2). Finally, an almost quantitative yield was obtained under twice-diluted conditions in a 1 : 1 ratio of toluene and water solvents even in the presence of 0.1 mol% of the rhodium source under almost neutral conditions (entry 3). Remarkably, additives such as bases were not required in this catalytic system, while previously developed metal complex systems for asymmetric 1,4-addition reactions of aryl boronic acids with nitroalkenes required either basic conditions or higher metal loadings of more than 3 mol%.^2^–^6^,^9^–^12^ The rhodium complex of ligand **4c** would provide an ideal chiral reaction environment for this enantioselective transformation.

**Table 1:** Effect of chiral dienes

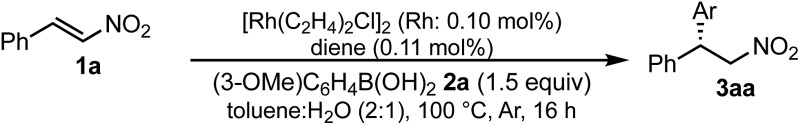			
Entry	Diene	Yield (%)	ee^*a*^ (%)
1	**4a**	88	71
2	**4b**	71	81
3	**4c**	62	90
4	**4d**	92	7

^*a*^As determined by HPLC.

**Table 2:** Optimization of solvent ratio in a homogeneous system

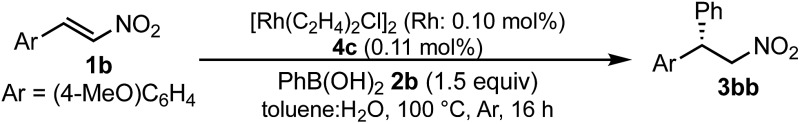			
Entry	Toluene : water for 0.3 mmol **1b** (μL)	Yield (%)	ee^*a*^ (%)
1	750 : 375	91	93
2	562 : 562	93	93
3	1000 : 1000	98	94

^*a*^As determined by HPLC.

Various combinations of nitroalkenes and arylboronic acids were examined under the optimal conditions. In almost all cases, the products were obtained in more than 90% yield with high enantioselectivities. Electron-rich and -deficient arylboronic acids could be used (Table 3, entries 1–7). *ortho*-Substituted arylboronic acids afforded 1,4-adducts with excellent enantioselectivities (entries 6 and 7). Alternatively, electron-rich and -deficient nitroalkenes could be used to give the desired products in high yields with high enantioselectivities (entries 8–15). In these nitroalkenes, a bromo-substituted nitroalkene could be used to afford the 1,4-adducts in 98% yield with 90% ee with tolerance of a bromine atom (entries 13 and 14). Twice the amount of chiral rhodium complex could afford the products in high yields with high enantioselectivities using heteroaromatic moieties including nitroalkenes (entries 17–20). When aliphatic nitroalkenes were examined, the reactions proceeded smoothly to afford the products in high yields with high enantioselectivities (entries 21–24). In the case of *n*-butyl-substituted nitroalkene, both the yield and enantioselectivity were slightly reduced. However, moderate yields of the desired products were observed and then ee values were high (entries 25 and 26). We note that no additives were required for any of these substrates.

**Table 3:** Substrate scope in the homogeneous system^*a*^

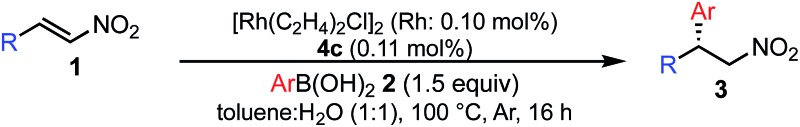					
Entry	R	Ar	Product	Yield (%)	ee (%)
1	Ph	(3-MeO)C_6_H_4_	**3aa**	99	91
2	Ph	(4-MeO)C_6_H_4_	**3ac**	95	84
3	Ph	(4-Cl)C_6_H_4_	**3ad**	87	94
4	Ph	(4-F)C_6_H_4_	**3ae**	82	94
5	Ph	(4-Me)C_6_H_4_	**3af**	92	86
6^*b*^	Ph	(2-MeO)C_6_H_4_	**3ag**	88	>99
7	Ph	(2-Me)C_6_H_4_	**3ah**	83	99
8	(3-MeO)C_6_H_4_	Ph	**3cb**	97	92
9	(4-MeO)C_6_H_4_	Ph	**3bb**	98	94
10	(4-MeO)C_6_H_4_	(3-MeO)C_6_H_4_	**3ba**	99	95
11	(4-Me)C_6_H_4_	Ph	**3db**	93	91
12	(4-Me)C_6_H_4_	(3-MeO)C_6_H_4_	**3ea**	94	93
13	(4-Br)C_6_H_4_	Ph	**3fb**	98	88
14	(4-Br)C_6_H_4_	(3-MeO)C_6_H_4_	**3fa**	98	90
15	(4-F)C_6_H_4_	Ph	**3gb**	96	89
16	(4-F)C_6_H_4_	(3-MeO)C_6_H_4_	**3ga**	90	91
17^*c*^	3-Furyl	Ph	**3hb**	92	94
18^*c*^	3-Furyl	(3-MeO)C_6_H_4_	**3ha**	88	95
19^*c*^	2-Thienyl	Ph	**3ib**	92	93
20^*c*^	2-Thienyl	(3-MeO)C_6_H_4_	**3ia**	96	94
21	Cyclohexyl	Ph	**3jb**	89	90
22^*c*^	Cyclohexyl	(3-MeO)C_6_H_4_	**3ja**	96	90
23^*c*^	i-Pr	Ph	**3kb**	86	90
24^*d*^	i-Pr	(3-MeO)C_6_H_4_	**3ka**	80	91
25^*c*^	*n*-Bu	Ph	**3lb**	78	84
26^*d*^	*n*-Bu	(3-MeO)C_6_H_4_	**3la**	66	86

^*a*^As determined by HPLC.

^*b*^0.25 mol% of [Rh(C_2_H_4_)_2_Cl]_2_ and 0.55 mol% of the chiral diene.

^*c*^0.1 mol% of [Rh(C_2_H_4_)_2_Cl]_2_ and 0.22 mol% of the chiral diene.

^*d*^0.15 mol% of [Rh(C_2_H_4_)_2_Cl]_2_ and 0.33 mol% of the chiral diene.

### Discussion of proposed reaction mechanism

**Fig. 2:**
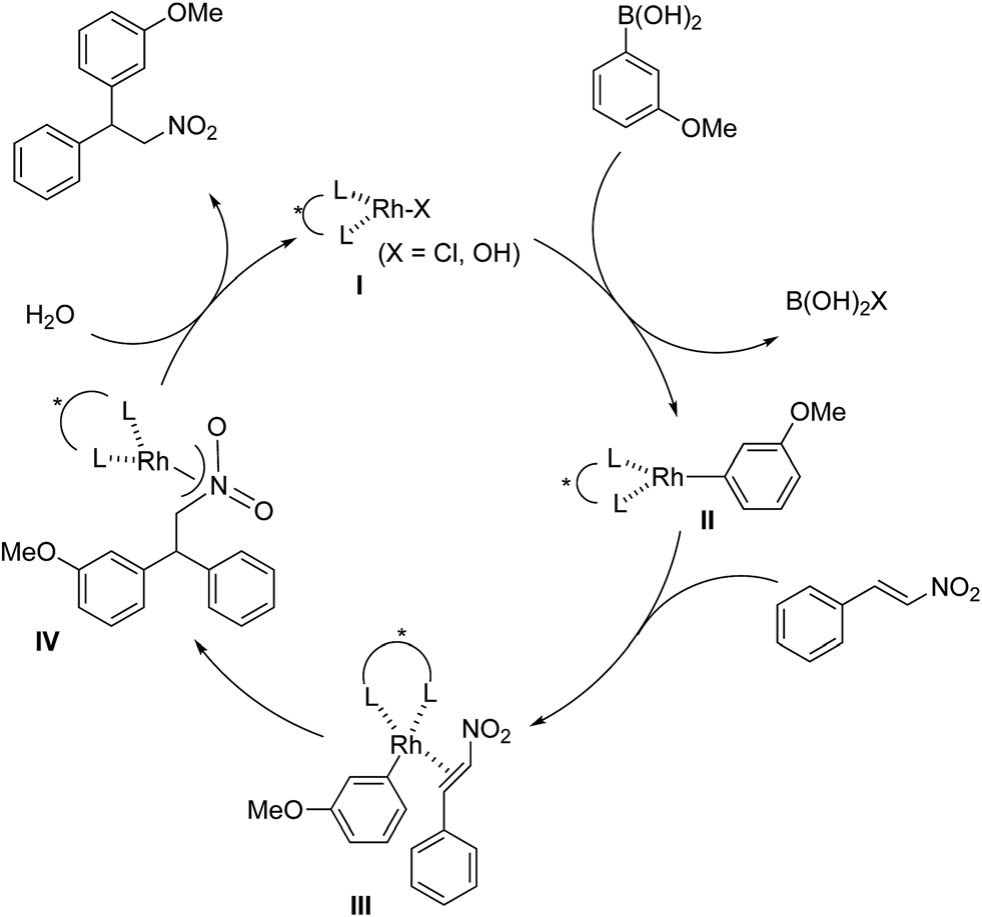
The assumed catalytic cycle.

The assumed catalytic cycle of the asymmetric 1,4-addition reactions of arylboronic acids with nitroalkenes is shown in Fig. 2.^2^

The induction of chirality occurs during the step where the aryl group is added to the β position of the nitroalkene (**III** → **IV**) from the viewpoint of the formation of the chirality in the product. However, some of the enantioselectivity is determined in the previous step (**II** → **III**); that is, the approach direction of nitroalkenes to the rhodium aryl complex **II** and the stability of the intermediate **III** are critical for stereocontrol of the desired product. A comparison of the yields and the enantioselectivities using ligands **4b**, **4c** and **4d** in homogeneous systems is shown in Table 1. For comparison, the results of the asymmetric 1,4-addition reaction of an arylboronic acid with an α,β-unsaturated ester in homogeneous systems depending on chiral dienes are shown in Table 4.^45^

**Table 4:** Comparison of chiral dienes in asymmetric 1,4-addition to an ester^*a*^

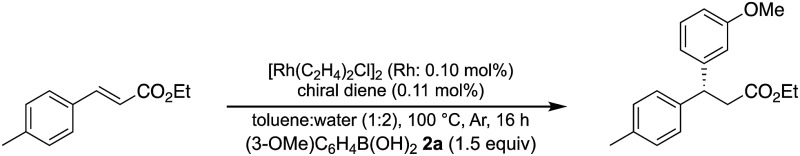			
Entry	Diene	Yield (%)	ee^*b*^ (%)
1	**4b**	72	94
2	**4c**	94	98
3	**4d**	69	67

^*a*^As determined by ^1^H NMR.

^*b*^As determined by HPLC.

In the case of the asymmetric 1,4-addition reaction of a nitroalkene, good to high yields of the desired product were obtained, while the enantioselectivities of the desired product were extremely diverse (Table 1). In the case of chiral diene **4b** bearing a tertiary butyl ester, the desired product was obtained with 81% ee (Table 1, entry 2), and the enantioselectivity was 90% ee when chiral diene **4c** bearing a tertiary butyl amide was used (entry 3). When a ligand lacking a proton on the nitrogen atom of the amide moiety, chiral diene **4d**, was used, the enantioselectivity was dramatically reduced (entry 4). These results suggest the importance of the presence of an amide proton for the enantioselectivities.

**Fig. 3:**
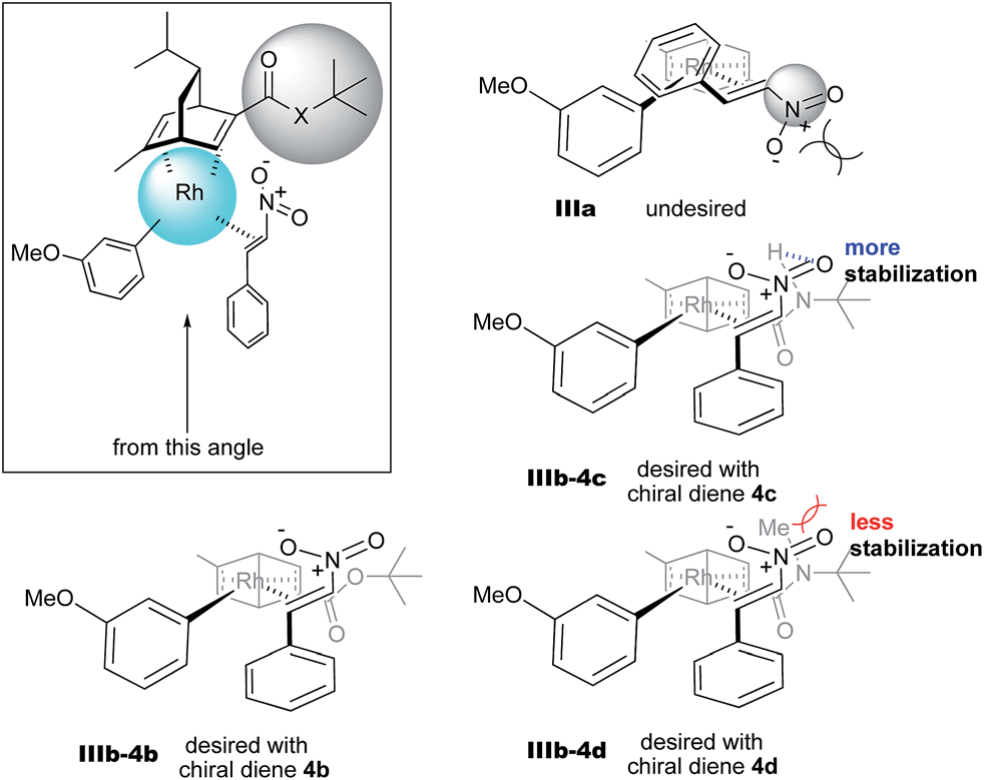
Assumed stereoselective models for intermediate **III**.

In contrast, in the case of the asymmetric 1,4-addition reaction of an α,β-unsaturated ester (Table 4), the presence of the proton on the secondary amide notably affects both the yields and the enantioselectivities (entry 2). It was suggested that the amide proton of **4c** not only participated in the stabilization of the ester substrate on the rhodium center through hydrogen bonding, but also accelerated the 1,4-addition step (or both) through its Brønsted acidity.^45^ Following our development, a similar bifunctional role of diene ligands containing a secondary amide moiety in asymmetric 1,4-addition reactions was discussed.^9^ Similarly, the amide proton in ligand **4c** might participate in the rigid fixation of a nitroalkene to realize high stereoselectivity, stabilizing key intermediate **III** more than in the case without hydrogen bonding. Several assumed transition state models of both desired and undesired intermediates **III** with three different ligands are shown in Fig. 3.

In asymmetric 1,4-addition reactions of arylboronic acids with nitroalkenes, the major enantiomer formed *via* the desired intermediate **IIIb** is shown in Fig. 3. During the approach of nitroalkenes to the rhodium aryl complex (**II** → **III**), because there is a large steric repulsion between the nitro group and the bulky part of the chiral diene (represented by a gray ball), the formation of the undesired intermediate (**II** → **IIIa**) is slower than the formation of the desired intermediate (**II** → **IIIb**). In addition, the stability of the intermediate **IIIa** may be lower than that of the intermediates **IIIb** because of the steric repulsion. The stereocontrol of the desired products derives from both the kinetics of the process (**II** → **III**) and thermodynamic stability of intermediate **IIIb**. Next, the structures of intermediates **IIIb** with different chiral dienes are compared. In the case of chiral diene **4c** bearing a secondary amide moiety, the nitroalkene would be fixed as shown by **IIIb-4c** in Fig. 3 *via* hydrogen bonding between the oxygen atoms of the nitro group and the proton of the amide. However, in the case where chiral diene **4b** bears a tertiary butyl ester moiety, there is no stabilization through hydrogen bonding. Therefore, the stability of **IIIb-4b** could be lower than that of **IIIb-4c**, thus resulting in the difference in enantioselectivity. In the case of chiral diene **4d**, bearing tertiary amide moiety, there is also no stabilization through hydrogen bonding. In addition, there appears to be steric repulsion between the nitro group and the methyl group on the amide nitrogen (**IIIb-4d**). Because of these factors, the dramatic drop in the enantioselectivity with chiral diene **4d** might be rationalized. That is, in the series of stereoselective pathways (**II** → **III** and stability of **III**), the amide proton of chiral diene **4c** plays an important role in achieving excellent enantioselectivities.

In contrast, improvement of the yield was not observed through the introduction of a secondary amide moiety in the ligand, unlike in the case of 1,4-addition to an α,β-unsaturated ester. In the case of 1,4-addition to an α,β-unsaturated ester, either the approach of the ester to a rhodium aryl complex or the 1,4-addition step is the rate-determining step and the secondary amide moiety in the ligand **4c** might accelerate these steps.^45^ This difference suggests that there is a variation in kinetic behavior (for example, a rate-determining step) between the case of a nitroalkene and the case of an α,β-unsaturated ester.

**Scheme 1:**
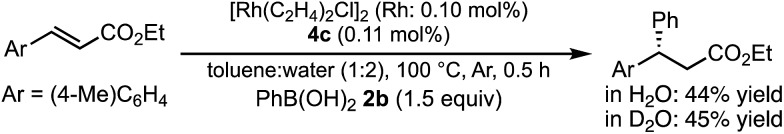
Reactions in nondeuterated and deuterated water in the case of esters.

In a previous report, it was postulated that the hydrolysis of Rh nitronate would be relatively slow.^2^ To investigate whether or not this is true in our present catalytic system, reactions in nondeuterated water and deuterated water were compared to examine whether the protonation step (**IV** → **I**) was the rate-determining step (Table 5). If protonation is the rate-determining step, the initial rate of the reaction in deuterated water should be slower than that in nondeuterated water because of the kinetic isotope effect. The reactions were conducted for 2 h in both cases and the yields and ee values of the desired product were examined. There was a clear kinetic isotope effect, that is, the yield in nondeuterated water (Table 5, entry 1) was much higher than that in deuterated water (entry 2), and this suggested protonation (**IV** → **I**) as the rate-determining step. In contrast, in the case of the asymmetric 1,4-addition of an arylboronic acid to an α,β-unsaturated ester, no difference in the yields between nondeuterated water and deuterated water was observed (Scheme 1). From these results, we concluded that the rate-determining step of the 1,4-addition to a nitroalkene was different from that of the 1,4-addition to an α,β-unsaturated ester.

**Table 5:** Reactions in nondeuterated and deuterated water

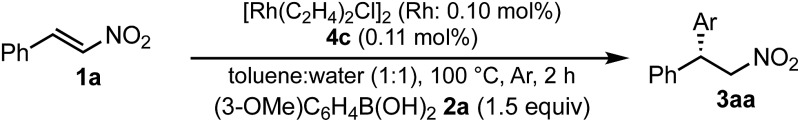			
Entry	Water	Yield (%)	ee (%)
1	H_2_O	74	90
2	D_2_O	29	90

**Fig. 4:**
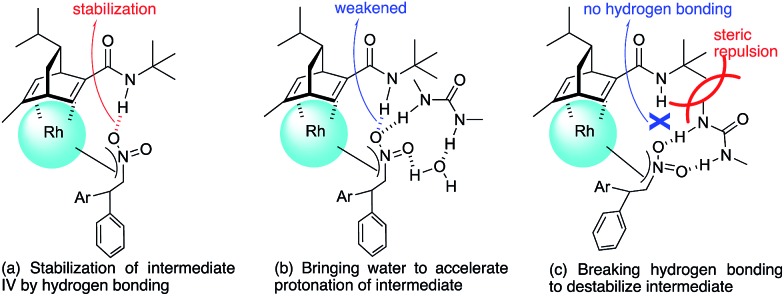
Assumed role of dimethylurea in the protonation step (**IV** → **I**).

Under conditions with a smaller proportion of water, that is, a 2 : 1 ratio of toluene : water, the reaction was much slower than that in a 1 : 1 ratio of toluene : water (Table 5, entry 1 [2 h; 74% yield] *vs.*Table 6, entry 3 [16 h; 62% yield]), probably because of slower protonation as a result of the lower availability of water. When we screened additives to enhance the reaction, we found that adding 0.5 mol% dimethylurea notably accelerated the reaction under these conditions. The chiral dienes bearing ester moiety **4b**, bearing secondary amide moiety **4c**, and bearing tertiary amide moiety **4d** were used as ligands and the effect of dimethylurea is summarized in Table 6. When the reactions were conducted without dimethylurea (Table 6, entries 1, 3 and 5, as in Table 1, entries 2–4), the yield was lower (**1a** remained) using chiral diene **4c** than using chiral dienes **4b** and **4d**. When we focus on the rate-determining step (**IV** → **I**), these results may be understood as a stabilizing effect of the rhodium nitronate intermediate **IV** through the amide proton of chiral diene **4c**, which makes the catalytic regenerations slow. The yields of the desired product were improved around 20% in the presence of dimethylurea, when either chiral diene **4b** or **4c** was used (entries 1 *vs.* 2 and entries 3 *vs.* 4). The enantioselectivities of the desired product were not associated with the presence of dimethylurea, and they depend on the structure of chiral dienes in these cases. These results suggest that dimethylurea affects only the rate-determining catalytic regeneration step (**IV** → **I**), which is separated from the chiral-inducing step. Based on these findings, we propose the possible transition states of the protonation, in which the amide proton of ligand **4c** stabilizes the rhodium nitronate intermediate (Fig. 4a) and dimethylurea accelerates the protonation of this intermediate (Fig. 4b and c). The role of dimethylurea in accelerating the regeneration of the catalyst is expected to attract a water molecule through hydrogen-bonding networks near the intermediate (Fig. 4b). Another possible role of dimethylurea is to be in perturbing the stabilizing effect of the amide proton through steric repulsion between the ligand and dimethylurea interacting with the nitro group (Fig. 4c). In both cases, newly formed hydrogen bonding between the nitro group and dimethylurea might weaken the existing hydrogen bonding to the secondary amide proton in ligand **4c** and the regeneration of the catalyst might be facilitated. We note that no acceleration of the reaction with the addition of dimethylurea was observed under the optimized conditions (in which toluene : water = 1 : 1), probably because the protonation was so fast that the effect of dimethylurea became undetectable in the presence of sufficient water (see ESI†). In other words, our catalytic system involving diene ligand **4c** with the secondary amide moiety possesses sufficiently high performance to achieve high catalytic turnover up to 1000 in the presence of sufficient water, even without any additives, while previously reported rhodium-catalyzed 1,4-addition reaction of aryl boronic acid with nitroalkenes required such additives to realize efficient catalytic turnover.^2^–^6^,^9^–^11^

**Table 6:** Effect of dimethylurea^*a*^

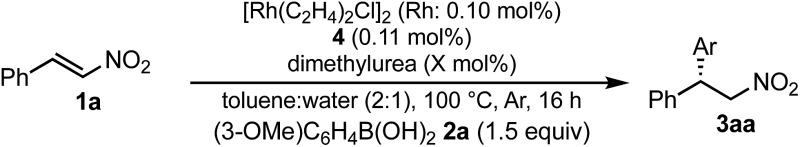				
Entry	Chiral diene	*X* (mol%)	Yield (%)	ee (%)
1	**4b**	0	71	81
2	**4b**	0.5	88	81
3	**4c**	0	62	90
4	**4c**	0.5	85	90
5	**4d**	0	91	7
6	**4d**	0.5	92	7

^*a*^As determined by ^1^H NMR analysis. Isolated yield in parentheses.

In contrast, when ligand **4d** with its tertiary amide moiety was used, very high yield was obtained regardless of the addition of dimethylurea, while enantioselectivities were extremely poor (Table 6, entries 5 *vs.* 6). In these cases, an intermediate before catalyst regeneration step **IV** might already be destabilized through the steric effect of the bulky tertiary amide moiety, and thus the protonation step (**IV** → **I** in Fig. 2) would be sufficiently fast.

### Development of heterogeneous metal nanoparticle systems

We next investigated heterogeneous chiral metal nanoparticle systems for the asymmetric 1,4-addition of aryl boronic acids to nitroalkenes. We initiated the study using PI metal nanoparticle catalysts containing rhodium and silver bimetallic nanoparticles with different ratios, PI/CB Rh/Ag (Table 7).^44^–^49^ The role of silver in the bimetallic nanoparticles was found to be in producing small nanoparticles with good dispersibility in the polymer support.^44^ We used 1 mol% of Rh in the heterogeneous catalysts with 0.1 mol% of ligand **4c**, because the Rh atoms that make up the core of the nanoparticles would not participate in catalysis. When the Rh nanoparticle catalyst was used under the optimized reaction conditions for homogeneous catalyst systems, a high yield and excellent enantioselectivity were observed (entry 1). When the ratio of silver was increased from 2 : 1 to 1 : 1, the yields were slightly improved maintaining excellent enantioselectivity (entries 2 and 3). Further increasing the proportion of silver in the heterogeneous catalysts resulted in decreased activity (entries 4 and 5). We considered the heterogeneous catalyst containing a 1 : 1 ratio of rhodium and silver as optimized. However, in all cases, a small amount of rhodium leaching was observed, probably because of the strong coordination ability of the nitro moiety in the substrate and the product.

**Table 7:** Effect of the ratio of Rh : Ag

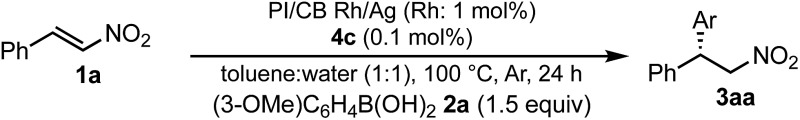				
Entry	Rh : Ag	Yield (%)	ee^*a*^ (%)	Leaching^*b*^ (%)
1	Only Rh	80	90	2.74
2	2 : 1	84	90	3.01
3	1 : 1	86	90	2.63
4	1 : 2	72	90	3.31
5	1 : 3	78	90	2.48
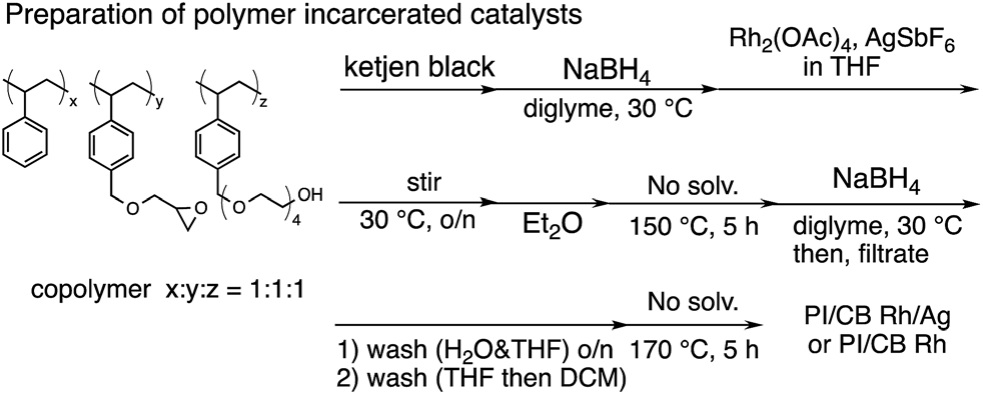				

^*a*^As determined by HPLC.

^*b*^As determined by ICP.

To suppress rhodium leaching and improve the yield, we investigated solvent systems (Table 8). When concentrated conditions were employed, the yield decreased and the amount of leaching increased (entries 2 *vs.* 3). When the proportion of toluene was reduced to 1 : 2, the yield of the product was low, and a significant amount of rhodium leached out (entry 1). These results indicate that the heterogeneous catalyst system is very sensitive to the organic solvent concentration. We continued careful investigations and decreased the proportion of water (entry 4), and it was found that diluted conditions both improved the activity and suppressed leaching (entries 4 and 5). Further decreasing of the proportion of water while maintaining the concentration of toluene (5 : 1 = toluene : water) resulted in an excellent yield without rhodium leaching, while maintaining excellent enantioselectivity as optimized conditions (entry 6). The amphiphilic nature of the polymer support might help the protonation of rhodium nitronate, for example, providing a hydrogen-bonding network with water molecules through ether and alcoholic moieties in polymer side chains, and resulting in smooth catalyst regeneration even with a smaller proportion of water compared with the homogeneous system.

**Table 8:** Effect of the proportion of toluene

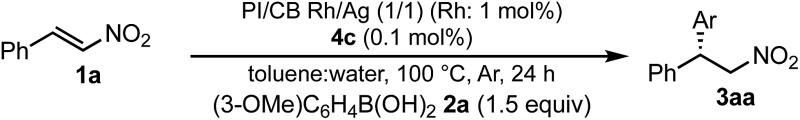					
Entry	Ratio toluene : water	Toluene : water for 0.3 mmol **1a** (μL)	Yield (%)	ee^*a*^ (%)	Leaching^*b*^ (%)
1	1 : 2	250 : 500	10	87	6.17
2	1 : 1	500 : 500	64	90	2.88
3	1 : 1	1000 : 1000	86	90	2.63
4	2 : 1	1000 : 500	89	91	2.09
5	2 : 1	2000 : 1000	89	92	BLD^*c*^
6	5 : 1	2000 : 400	90	92	BLD^*c*^

^*a*^As determined by HPLC.

^*b*^As determined by ICP.

^*c*^BLD = below the limit of detection. LD = 0.39%.

Under optimized conditions, various combinations of arylboronic acids and nitroalkenes were examined. The results are shown in Table 9. Electron-rich and -deficient arylboronic acids were examined (Table 9, entries 1–7). Under the optimized conditions, the desired product **3aa** was obtained in 90% yield with 92% ee (entry 1); however, other arylboronic acids afforded the desired products in around 60% yields. A prolonged reaction time (48 h) afforded the desired products in moderate-to-high yields while maintaining high enantioselectivities (entries 2–5). In the cases of *ortho*-substituted arylboronic acids, the desired products were obtained with excellent enantioselectivities as observed in the homogeneous system (entries 6 and 7). In the case of *o*-methoxyphenylboronic acid, 5 mol% of the catalyst and 0.5 mol% of the chiral diene **4c** gave only a 36% yield of the desired product (entry 6). Steric hindrance of the methoxy group at the *ortho*-position might make the reaction slower. Electron-rich and -deficient nitroalkenes were then examined (entries 8–15). When phenylboronic acid was used, the desired products were obtained in moderate yields. The reactions were conducted for prolonged times and the desired products could be obtained in high yields with high enantioselectivities (entries 8, 9, 12 and 14). In the case of 3-methoxyphenylboronic acids, the optimal conditions gave the desired products in high yields with high enantioselectivities (entries 10, 11, 13 and 15). Nitroalkenes bearing heteroaromatic moieties also gave the 1,4-adducts in moderate-to-high yields with high enantioselectivities through increasing the loading of the catalyst and chiral diene **4c** (entries 16 and 17). While a sulfur atom can usually coordinate to metal nanoparticles and might poison the catalyst, the reaction could be applied to a thienyl-substituted nitroalkene (entry 17). Next, aliphatic nitroalkenes were examined. When a cyclohexyl-substituted nitroalkene was used, the desired product was obtained in 71% yield with 91% ee under the optimal conditions (entry 18). In the case of an isopropyl-substituted nitroalkene, 2 mol% of the catalyst and 0.2 mol% of chiral diene **4c** afforded the desired product in 70% yield with 90% ee (entry 19). However, in the case of *n*-butyl-substituted nitroalkene, the desired product was obtained in only 25% yield. To improve the yield, we tried prolonging the reaction time and increasing the catalyst loading; however, these trials showed no improvement of the yield.

**Table 9:** Substrate scope in a heterogeneous system^*a*^

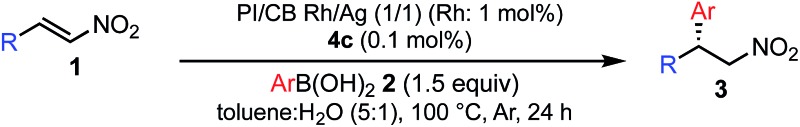					
Entry	R	Ar	Product	Yield (%)	ee (%)
1	Ph	(3-MeO)C_6_H_4_	**3aa**	90	92
2^*b*^	Ph	(4-MeO)C_6_H_4_	**3ac**	73	84
3^*b*^	Ph	(4-Cl)C_6_H_4_	**3ad**	75	95
4^*b*^	Ph	(4-F)C_6_H_4_	**3ae**	71	94
5^*b*^	Ph	(4-Me)C_6_H_4_	**3af**	90	85
6^*c*^	Ph	(2-MeO)C_6_H_4_	**3ag**	36	>99
7^*b*^	Ph	(2-Me)C_6_H_4_	**3ah**	71	99
8^*b*^	(3-MeO)C_6_H_4_	Ph	**3cb**	96	92
9^*b*^	(4-MeO)C_6_H_4_	Ph	**3bb**	93	94
10	(4-MeO)C_6_H_4_	(3-MeO)C_6_H_4_	**3ba**	93	95
11	(4-Me)C_6_H_4_	(3-MeO)C_6_H_4_	**3ea**	89	93
12^*b*^	(4-Br)C_6_H_4_	Ph	**3fb**	90	88
13	(4-Br)C_6_H_4_	(3-MeO)C_6_H_4_	**3fa**	88	90
14^*b*^	(4-F)C_6_H_4_	Ph	**3gb**	87	89
15	(4-F)C_6_H_4_	(3-MeO)C_6_H_4_	**3ga**	95	91
16^*d*^	3-Furyl	(3-MeO)C_6_H_4_	**3ha**	60	94
17^*e*^	2-Thienyl	(3-MeO)C_6_H_4_	**3ia**	88	94
18	Cyclohexyl	(3-MeO)C_6_H_4_	**3ja**	71	91
19^*e*^	i-Pr	(3-MeO)C_6_H_4_	**3ka**	70	90
20	*n*-Bu	(3-MeO)C_6_H_4_	**3la**	25	85

^*a*^As determined by HPLC.

^*b*^48 h.

^*c*^5 mol% of PI/CB Rh/Ag and 0.5 mol% of the chiral diene **4c**.

^*d*^3 mol% of PI/CB Rh/Ag and 0.3 mol% of the chiral diene **4c**.

^*e*^2 mol% of PI/CB Rh/Ag and 0.2 mol% of the chiral diene **4c**.

**Scheme 2:**
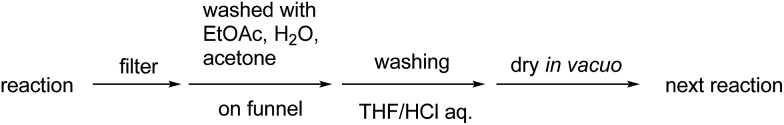
Method of catalyst recovery.

We investigated the recovery and reuse of the PI/CB-Rh/Ag catalyst in the asymmetric 1,4-addition reaction of boronic acid **2a** with nitrostyrene **1a**. We found that a simple recovery method including a wash with an acidic solution was effective for maintaining the high activity of the recovered catalyst (Scheme 2), and the catalytic activity was slightly decreased but almost maintained for at least five uses without changing the enantioselectivity (Table 10).

**Table 10:** Method of catalyst recovery^*a*^

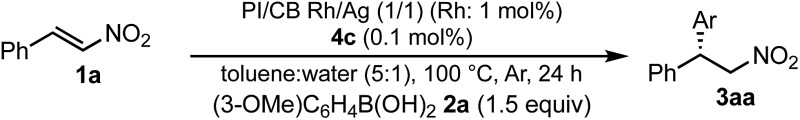					
Run	1^st^	2^nd^	3^rd^	4^th^	5^th^
Yield (%)	93	92	86	82	75
ee (%)^*b*^	91	91	92	92	92

^*a*^ **4c** was added in every run.

^*b*^As determined by HPLC.

**Fig. 5:**
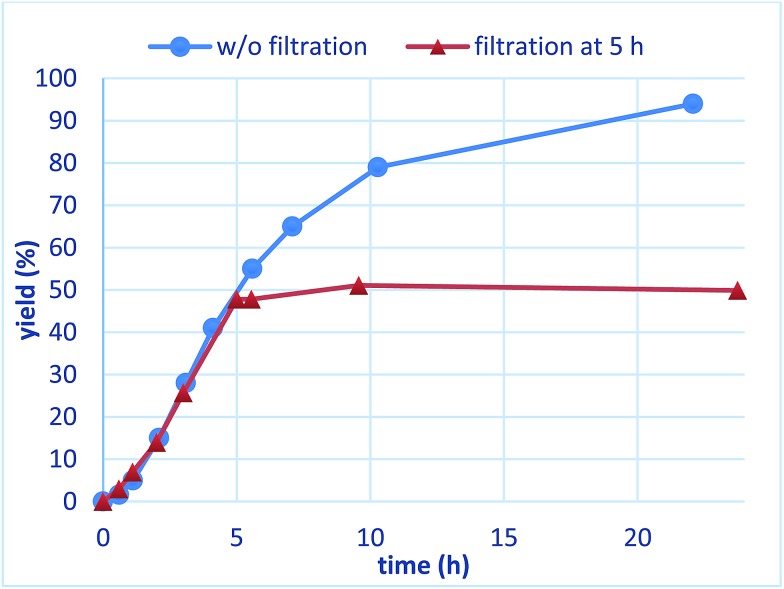
Hot leaching test (yields were determined with GC using an internal standard).

To confirm whether the catalysis proceeds within the solid phase over the entire period of a reaction, a hot filtration test was conducted, and no leaching of metals into the final product was confirmed by using ICP. Under optimized conditions, PI/CB Rh/Ag was removed using filtration at the stage of a 50% conversion ratio, while maintaining a high reaction temperature (Fig. 5). The reaction in the filtrate after removal of the catalyst was allowed to continue, but the yield of the product did not increase any further. This finding clearly demonstrates that the heterogeneous catalyst was responsible for the reaction.

**Fig. 6:**
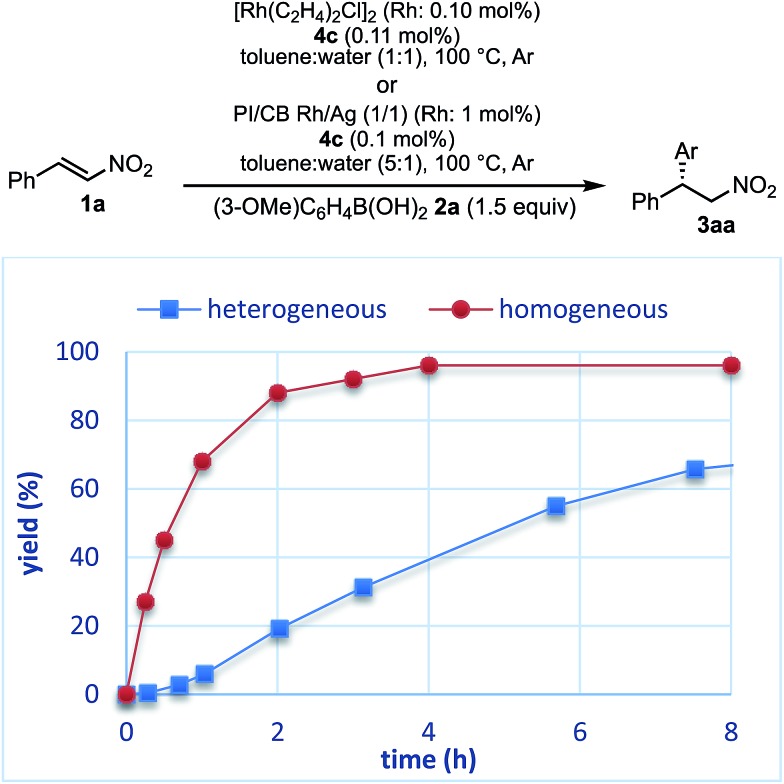
Comparison of reaction plots between homogeneous and heterogeneous catalyst systems.

**Fig. 7:**
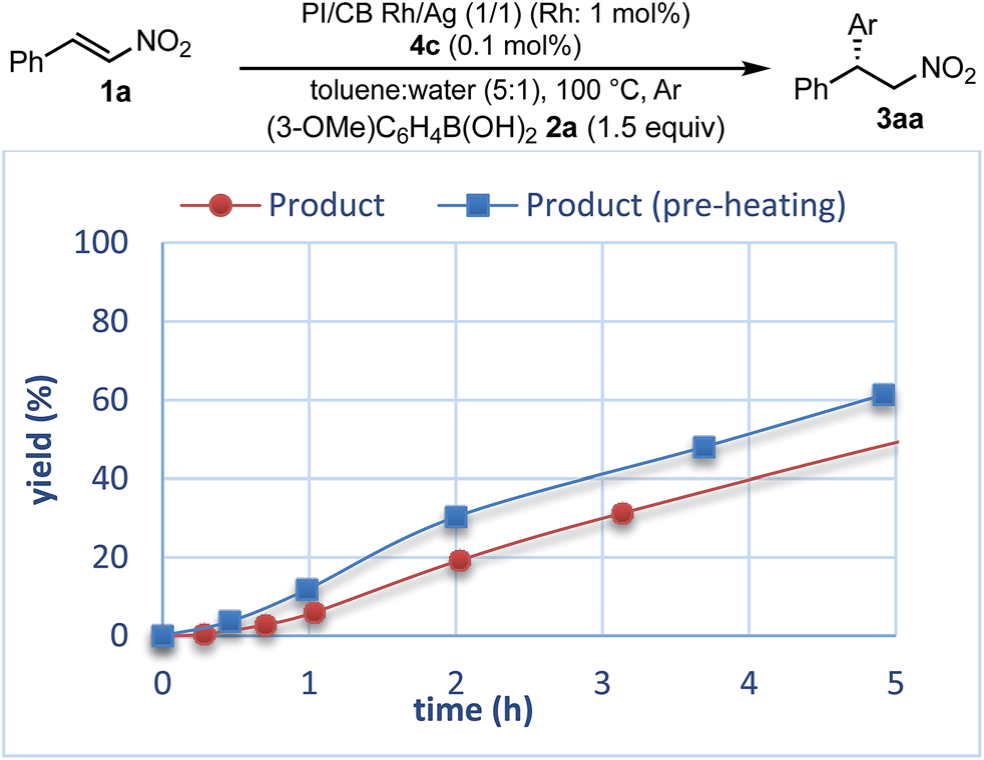
Effect of pre-treatment.

The reaction profiles of the homogeneous and heterogeneous catalyst systems were compared (Fig. 6). Although the reaction proceeded smoothly without an induction period in the case of a homogeneous system, a clear induction period of around 1 h was observed in the heterogeneous catalyst system. After the induction period, the yield increased constantly in the heterogeneous system. In our previous investigation, a similar induction period was observed for the 1,4-addition reaction of an enoate, and it disappeared with preheating with aryl boronic acid and some reductants.^45^ In the case of the nitrostyrene **1a**, a similar phenomenon, reducing the induction period, was effected by preheating with an aryl boronic acid, probably because of a redox process between the phenyl boronic acid **2a** and metal nanoparticles to generate biphenyl or phenol, which were often observed as a small amount of by-product under optimized conditions (Fig. 7). Generation of an active species and shortening the induction period through redox processes were also reported for metal nanoparticle catalysis.^56^–^58^ In order to understand the nature of the active species further, we additionally examined several reaction profiles; (1) monitoring the reaction profile of recovered catalysts during recovery and reuse, and (2) monitoring the profile of the collected catalysts after a hot filtration test (ESI Fig. 7–9†). Monitoring of the recovered catalyst was conducted under the same reaction conditions as in Table 10 (ESI Fig. 7†). The induction period of the 2nd run was slightly prolonged and the reaction rate at steady state was also slightly slower than that of the 1st run. In the 3rd run, a slower reaction rate at steady state was observed; however, the desired product was obtained in 85% yield after 24 h. In contrast, the collected catalyst, after a hot filtration at 4 h, showed almost the same reaction profile as that of the fresh catalyst during 24 h of reaction time with a similar induction period (ESI Fig. 8†). The catalyst was recovered and the reaction profile was monitored; however, a slower reaction rate than that of the previous run was observed. The collected catalyst, after a hot filtration at 6 h, also gave a similar reaction profile as the 1st run (ESI Fig. 9†). The catalyst was again filtered under heated conditions at 6 h and the next run showed almost a similar reaction rate. According to the reaction profile of the fresh catalyst, the reaction rate slowed down after 10 h. We expected that irreversible deactivation of the catalyst partially occurred during this period and such deactivated species might not be activated even in an induction period of the next run. On the other hand, the catalyst collected by a hot filtration at steady state, where the reaction proceeded at a constant rate, showed almost the same reaction profile including an induction period as the fresh catalyst. This result indicated that active species could not be maintained during the filtration process, but returned to initial potentially active species, which required activation in the induction period in the next run. Notably, no irreversible deactivation of the catalyst may proceed during this steady state. To clarify the difference among these catalysts, XPS analyses of the fresh catalyst, the catalyst after recovery and reuse, the catalyst collected by a hot filtration during an induction period (at 1 h) and the catalyst collected by a hot filtration during steady state (at 5 h) were conducted (ESI Fig. 10 and 11†). Both silver and rhodium species were analysed and the peaks of both rhodium and silver gradually shifted to lower energy values as the catalyst was used for a longer time. In particular, the catalyst after recovery and reuse showed significant peak shifts. Based on these results, we assumed that further reduction of the active species at a latter stage of the reaction (after 10 h) generates irreversibly deactivated species. On the other hand, such deactivation did not proceed during steady state and active species returned to almost the same metal species as the fresh catalyst during a recovery process even when mixtures were filtered under hot filtration conditions.

**Fig. 8:**
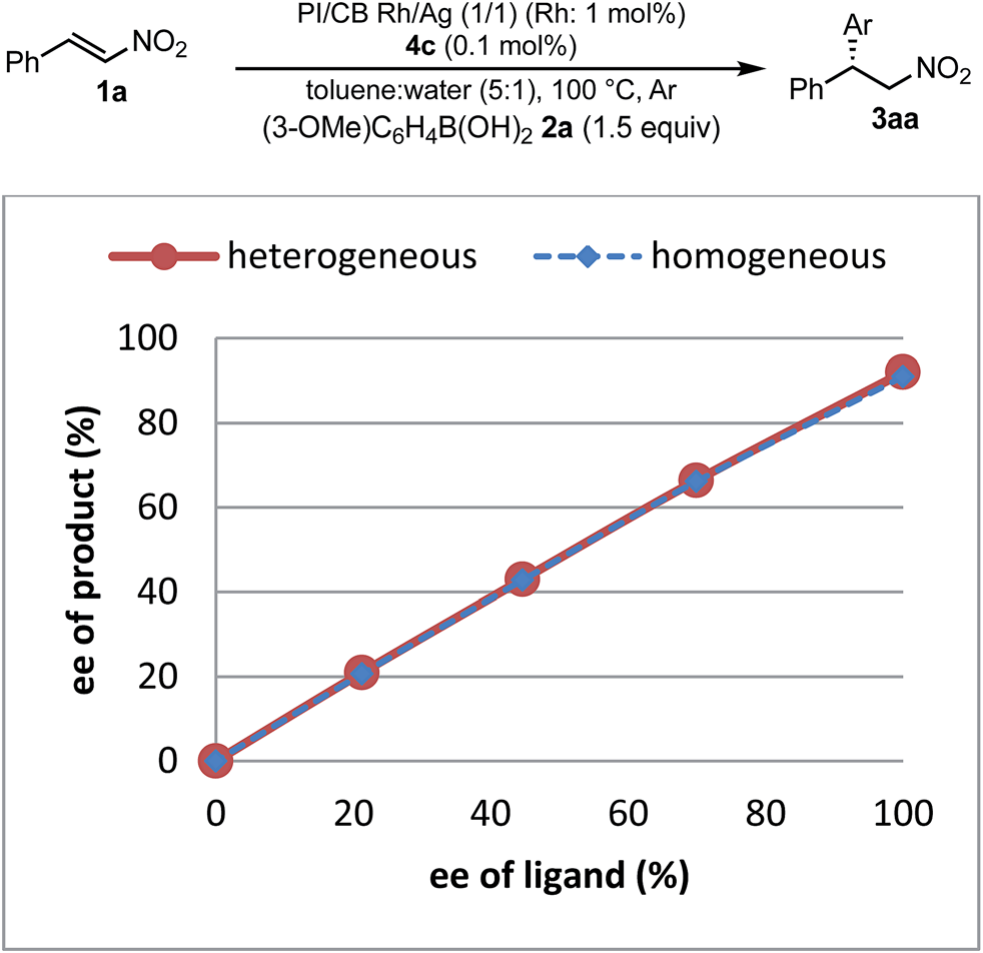
Nonlinear effect.

To understand the similarities and differences between active species in homogeneous and heterogeneous systems, a nonlinear effect analysis was conducted for both reaction systems. Almost linear relationships between the enantiomeric excess of the ligand and the product were observed in both reaction systems (Fig. 8). This is in clear contrast to the case of the 1,4-addition reaction of an enoate in our previous investigation, in which only a heterogeneous system showed a positive nonlinear effect.^45^

As a similarity of homogeneous and heterogeneous systems, the enantioselectivities of the desired product are discussed in addition to nonlinear effect analysis. In the substrate scopes examined in homogeneous (Table 3) and heterogeneous (Table 9) systems, the differences in the enantioselectivities of the desired product from the same combination of the substrates were within just 1%. These results suggest that reaction environments of the active species in homogeneous and heterogeneous systems were almost identical.

**Fig. 9:**
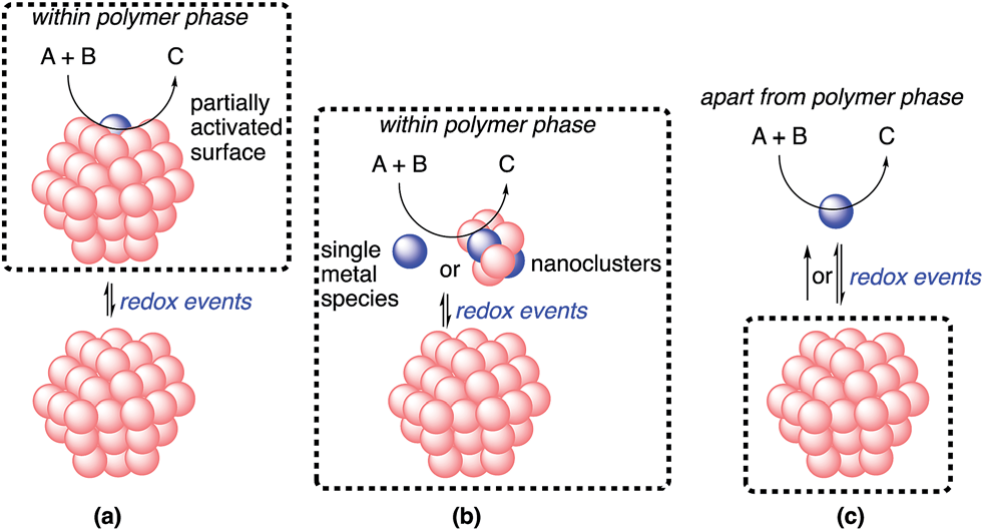
Possible catalytic systems.

The possible catalytic systems explaining how the reaction occurs through metal nanoparticle catalysis were proposed by Ananikov and Beletskaya in 2012.^27^,^59^ Based on these reports, we propose three possibilities (Fig. 9). (a) The reaction proceeds on the surface of the metal nanoparticles that are partially activated through redox events without the separation or departure of any atom. (b) Some metal complexes or nanoclusters depart from the metal nanoparticles as active species through redox events, but remain near the metal nanoparticles. The reaction proceeds on these complexes or nanoclusters, and then these complexes or nanoclusters return to the metal nanoparticles. This cycle proceeds within the reaction environment of the polymer phase. (c) Some metal complexes leach from the nanoparticles through redox events and the reaction proceeds on both these complexes and the surface of the metal nanoparticles. The leached metal complexes do not return to the metal nanoparticles or return after completion of the reactions.

In heterogeneous chiral rhodium nanoparticle-catalyzed asymmetric 1,4-addition reactions of arylboronic acids with nitroalkenes, rhodium leaching could be suppressed completely under optimized conditions. In addition, a hot filtration test clearly demonstrated the heterogeneity of the reaction environment where actual catalysis occurred. Moreover, the catalyst could be recovered and reused at least five times while maintaining almost the same level of reactivity and excellent enantioselectivity. From these results, the possibility of (c) might be excluded.

Considering the similarity of the outcomes of enantioselectivities in homogeneous and heterogeneous systems as discussed, catalysis (b), in which the reaction proceeds on metal complexes or on very small nanoclusters within the polymer phase, may be more likely than catalysis (a). In this case, metal complexes or nanoclusters depart from large nanoparticles to form active species through redox events that result in the induction period, and ligand **4c** coordinates to these active species to provide an efficient chiral reaction environment for asymmetric catalysis. However, the possibility of catalysis (a) cannot be excluded completely.

## Conclusion

In summary, asymmetric 1,4-addition reactions of arylboronic acids with nitroalkenes catalyzed by a rhodium complex of the chiral diene **4c** bearing a tertiary butyl amide moiety were developed, and this complex is more efficient than previously reported homogeneous catalyst systems. Just 0.1 mol% of the chiral rhodium complex could catalyze the reactions and afford the desired products in high yields with excellent enantioselectivities. There was no need for additives in this catalysis and a wide substrate scope was shown. Mechanistic studies using deuterated water and dimethylurea as an additive revealed the rate-limiting step in the catalyst system, using the chiral diene **4c**, to be the protonation step, while extremely high catalytic turnover was achieved by the developed catalytic systems without any additives under optimized conditions using an amount of water sufficient to accelerate the regeneration step for this catalyst.

The developed homogeneous catalyst systems could be converted to heterogeneous metal nanoparticle systems, maintaining completely the same level of enantioselectivity with a wide substrate scope without any additives. To our knowledge, this is the first example of asymmetric 1,4-addition reactions of arylboronic acids with nitroalkenes in a heterogeneous system. In this catalysis, 1 mol% of rhodium nanoparticle catalyst and 0.1 mol% of chiral diene were sufficient to obtain excellent enantioselectivities because of a strong ligand-accelerating effect. The heterogeneous catalyst, PI/CB Rh/Ag, could be recovered and reused while maintaining high enantioselectivity. Although several similarities between homogeneous and heterogeneous systems were demonstrated, we confirmed that the reaction proceeded within the environment of the heterogeneous catalyst system through mechanistic studies. In addition, the catalytic systems developed here have completely different characters from the catalytic systems developed for asymmetric 1,4-addition using enoates previously reported by our group, in terms of both the rate-limiting step in the catalytic cycle, and similarities and differences between homogeneous and heterogeneous systems.

## Conflicts of interest

There are no conflicts to declare.

## Supplementary Material

Supplementary information
