# Supplementary material for: Rhodium-catalyzed asymmetric 1,4-addition reactions of aryl boronic acids with nitroalkenes: reaction mechanism and development of homogeneous and heterogeneous catalysts
Source: Chem Sci. 2017 Oct 11;8(12):8362–72. doi: 10.1039/c7sc03025h (PMC5863615; doi:10.1039/c7sc03025h)
Supplement: Supplementary file 1 [file SC-008-C7SC03025H-s001.pdf]

Electronic Supplementary Material (ESI) for Chemical Science.
This journal is © The Royal Society of Chemistry 2017

Rhodium-Catalyzed Asymmetric 1,4-Addition Reactions of Aryl Boronic Acids with Nitroalkenes: Reaction Mechanism and Development of Homogeneous and Heterogeneous Catalysts

Hiroyuki Miyamura, Kohei Nishino, Tomohiro Yasukawa, and Shū Kobayashi*

Department of Chemistry, School of Science, The University of Tokyo, Hongo, Bunkyo-ku, Tokyo 113-0033, Japan.

Electronic Supplementary Information

Contents

1. General -- S-1
2. Preparation of chiral dienes -- S-3
3. Preparation of polymer incarcerated metal nanoparticle catalysts ------------------- S-4
4. Preparation of substrates -- S-8
5. Asymmetric 1,4-addition reactions -- S-10
6. Reaction profiles in toluene/H_2O = 1/1 --- S-11
7. Reaction profiles during recovery and reuse, after hot filtration ------------------- S-12
8. XPS analysis --- S-14
9. Products --- S-16
10. Charts -- S-23
11. References -- S-136

1. General

- JEOL JMN-LA400, 500 or 600 spectrometers were used for NMR measurement. Chloroform ($\delta = 7.24$) or tetramethylsilane ($\delta = 0.00$) was used as an internal standard for ^{1}H NMR and $CDCl_3$ ($\delta = 77.0$) for ^{13}C NMR. Structures of known compounds were confirmed by comparison with commercially available compounds or data shown in literature.
- IR spectra were measured on a JASCO FT/IR-610 spectrometer.
- Direct Analysis in Real Time (DART) mass spectra were recorded on JEOL JMS-T100TD mass spectrometer.
- Specific rotations were recorded with JASCO P-1010 or P-2100.
- Melting point was determined on a standard melting point apparatus and is uncorrected.
- Preparative thin-layer chromatography was carried out using Wakogel B-5F.
- ICP analysis was performed on Shimadzu ICPS-7510 equipment.
- GC analysis. was performed on Shimadzu GC-2010 apparatus (**Condition :** column = J

& W SCIENTIFIC DB-1 0.25 mm ID, 0.25 μm, 60.0 m; Gas pressure: 157.5 kPa, Total flow: 41.3 mL/min, Column folw: 0.93 mL/min, Velocity: 21.1 cm/sec; Purge flow: 3.0 mL/min; Sprit ratio: 40.1; Injector: 300 °C, FID: 300 °C; Column program: starting from 100 ˚C, 20 ˚C/min to 300 ˚C, 10 min hold).

- HPLC analysis was performed on Shimadu LC-20AB, SPD-M20A and DGU-20A_3.
- XPS analysis was performed on JPS-9010MC with a Mg Kα X-ray source and the O 1s line at 532.9 eV was used as reference to correct the binding energies.
- The absolute configuration of reported compounds was determined by comparison to literature and that of other products was assumed by analogy.
- STEM/EDS images were obtained using a JEOL JEM-2100F instrument operated at 200 kV. All STEM specimens were prepared by placing a drop of the solution on carbon-coated Cu grids and allowed to dry in air (without staining).
- $Rh_2(OAc)_4$ was purchased from Strem Chemical Inc.
- $[Rh(C_2H_4)_2Cl]_2$ and V-70 were purchased from Wako Pure Chemical Company.
- $AgSbF_6$ complex was purchased from Sigma-Aldrich Co., Ltd..
- $NaBH_4$ was purchased from Wako Pure Chemical Company and recrystallized from diglyme by heating according to the literature[1] and stored in a glove box.
- Ketjen black EC300J was purchased from Lion Corporation.
- Toluene was purchased in dried grade from Wako Pure Chemical Company and used without further purification.
- Deionized water from a MILLIPORE MilliQ machine (Gradient A 10) was used as solvent without further treatment.
- Aliphatic unsaturated esters and cinnamate were purchased from Tokyo Kasei Kogyo Co., Ltd..
- Nitroalkenes were prepared by following the literature.
- Arylboronic acids were purchased from Wako Pure Chemical Company or prepared from the corresponding Grignard reagent. The ratio of boronic acid to boroxine was determined by ^{1}H NMR analysis before use.
- Chiral diene **4a**, **4b**, and **S1** were prepared by following the literatures.[2a]
- PI/CB Rh/Ag was prepared by following the literature.[3]
- Asymmetric 1,4-addition reactions were conducted with Carousel™.
- Unless otherwise stated, all reactions were carried out under argon atmosphere.
- 0.45 μm PTFE membrane filter (Whatman™ cat. No. 6784-2504) was used for filtration of solid catalysts during hot filtration test and taking reaction profiles.
- Yields were determined by GC analysis using durene as an internal standard for taking reaction profiles.
- Procedures conducted in previous published paper (Yasukawa, T.; Suzuki, A.; Miyamura, H.; Nishino, K.; Kobayashi, S., *J. Am. Chem. Soc.* **2015**, *137*, 6616.) were followed for hot leaching test and taking reaction profile with preheating.

2. Preparation of chiral dienes

2-1. Saponification of 7-isopropyl-5-methylbicyclo[2.2.2]octa-2,5-diene-2-carboxylate S1[2]

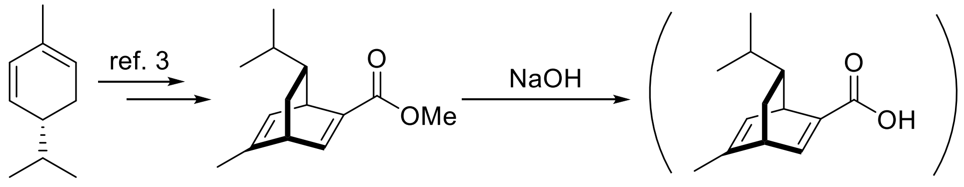

S1 **S2**

Chiral diene **S1** (55 mg, 0.25 mmol) in MeOH (3 mL) and NaOH (40 mg, 1 mmol) in water (1.5 mL) were mixed. After stirring for 6 h at 50 ºC, 1N HCl (5 mL) was added. The water layer was extracted with CH_2Cl_2, and the combined organic layers were washed with brine and dried over Na_2SO_4. The precipitation was removed by filtration and the filtrate was evaporated and dried under vacuum to afford the crude desired compound **S2** (57.0 mg, quantitative yield) as a solid, which was used in the next reaction without further purification.
^{1}H NMR ($CDCl_3$, 500 MHz) δ: 7.44 (1H, dd, *J* = 6.2, 1.7 Hz), 5.81 (1H, dt, *J* = 6.2, 1.7 Hz), 4.06 (1H, dt, *J* = 6.2, 1.7 Hz), 3.43-3.40 (1H, m), 1.59-1.54 (1H, m), 1.56 (3H, ddd, *J* = 11.3, 9.1, 2.8 Hz), 1.20-1.14 (1H, m), 1.13-1.05 (1H, m), 0.98 (3H, d, *J* = 6.8 Hz), 0.98-0.95 (1H, m), 0.82 (3H, d, *J* = 6.8 Hz). ^{1}H NMR ($CDCl_3$, 125 MHz) δ: 170.3, 148.9, 143.1, 140.5, 124.1, 47.6, 44.2, 39.2, 33.7, 31.4, 21.8, 21.3, 18.9.

2-2. Preparation of (1*R*,4*R*,7*R*)-*N*-(*tert*-butyl)-7-isopropyl-5-methylbicyclo[2.2.2]octa-2,5-diene-2-carboxamide (4c)

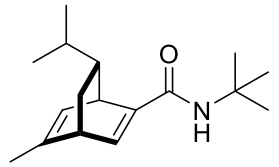

tert-Butylamine (21.5 μL, 0.2 mmol) and *N,N'*-diisopropylcarbodiimide (34.7 μL, 0.22 mmol) in CH_2Cl_2 (500 μL) were added to a solution of carboxylic acid **S2** (41.3 mg, 0.2 mmol), 1-hydroxybenzotriazole (27.0 mg, 0.2 mmol) and *N,N*-dimethyl-4-aminopyridine (1.2 mg, 0.01 mmol) in CH_2Cl_2 (500 μL) under argon. After stirring for 9 h at 50 ºC, triethylamine (0.4 mmol) and then *tert*-butylamine (21.5 μL, 0.2 mmol) were added. After stirring for 24 h at 50 ºC, 1N HCl (3 mL) was added. The precipitation was removed by filtration and the water layer was extracted with CH_2Cl_2. The combined organic layers were washed with brine, dried over Na_2SO_4 and concentrated under vacuum. The residue was purified by preparative TLC (hexane/EtOAc = 5/1) to afford **4c** (28.3 mg, 54% yield) as a white solid.
$[\alpha]^{20}_{D}$ = +25.6 (c = 0.09, $CHCl_3$). IR (KBr) cm^{-1}: 3341, 2961, 2938, 2869, 1662, 1630, 1605, 1536, 1447, 1364, 1285, 1220, 815. M.p. = 123-126 ºC. ^{1}H NMR ($CDCl_3$, 600 MHz) δ: 6.68 (1H, dd, *J* = 6.2, 1.4 Hz), 5.80 (1H, d, *J* = 6.2 Hz), 5.49 (1H, br s), 4.01-4.00 (1H, m), 3.30 (1H, dt, *J* = 6.2, 2.1 Hz), 1.81 (3H, d, *J* = 1.4 Hz), 1.58-1.56 (1H, m), 1.37 (9H, s), 1.26-1.19 (1H, m), 1.10-1.05 (1H, m), 1.00 (3H, d, *J* = 6.9 Hz), 0.94 (1H, ddd, *J* = 11.7, 4.8, 2.8 Hz), 0.81 (3H, d, *J* = 6.9 Hz). ^{13}C NMR ($CDCl_3$, 125 MHz) δ: 165.8, 146.1, 143.9, 136.6, 124.3, 51.0, 47.7, 43.5, 40.0, 33.9, 31.9, 28.9, 21.8, 21.3, 19.0. HRMS (DART) calculated for

$C_{17}H_{28}NO^+$ [M+H^+] 262.21709, found 262.21643.

2-3. (1*R*,4*R*,7*R*)-*N*-(*tert*-butyl)-7-isopropyl-*N*,5-dimethylbicyclo[2.2.2]octa-2,5-diene-2-carboxamide (4d)

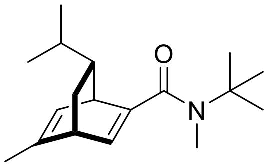

Oxalyl chloride (65 μL, 0.75 mmol) and dimethylformamide (8.5 μL, 0.11 mmol) were added to the solution of carboxylic acid **S2** (103 mg, 0.5 mmol) in CH_2Cl_2 (1 mL) at 0 ºC under argon. After stirring for 3 h at 0 ºC, triethylamine (350 μL, 2.5 mmol) and then *tert*-butyl methyl amine (1.5 mmol) in CH_2Cl_2 (1 mL) were added at 0 ºC. After the mixture was stirred for 24 h at room temperature, sat. NH_4Cl aq. (4 mL) was added. The water layer was extracted with CH_2Cl_2 and the combined organic layers were washed with brine, dried over Na_2SO_4 and concentrated under vacuum. The residue was purified by preparative TLC (hexane/EtOAc = 5/1) to afford **4d** (42.9 mg, 62 % yield) as a liquid.

$[\alpha]^{20}_D$ = +28.4 (c = 0.31, $CHCl_3$). IR (neat) cm^{-1}: 3037, 2957, 2869, 1632, 1472, 1365, 1150, 1057, 821. ^1H NMR ($CDCl_3$, 500 MHz) δ: 6.37 (1H, dd, J = 6.2, 1.7 Hz), 5.79 (1H, d, J = 6.2 Hz), 3.63 (1H, dt, J = 6.2, 1.7 Hz), 3.27-3.24 (1H, m), 2.78 (3H, s), 1.80 (3H, d, J = 1.7 Hz), 1.60 (1H, ddd, J = 11.9, 8.5, 2.8 Hz), 1.39 (9H, s), 1.37-1.32 (1H, m), 1.08-1.01 (1H, m), 0.94 (3H, d, J = 6.8 Hz), 0.90 (1H, ddd, J = 11.3, 4.5, 2.3 Hz), 0.79 (3H, d, J = 6.8 Hz). ^{13}C NMR ($CDCl_3$, 125 MHz) δ: 172.2, 147.2, 144.1, 134.8, 123.9, 56.0, 48.0, 43.2, 42.4, 34.5, 34.0, 32.2, 27.8, 21.7, 21.4, 19.1. HRMS (DART) calculated for $C_{18}H_{30}NO^+$ [M+H^+] 276.23274, found 276.23370.

3. Preparation of polymer incarcerated metal nanoparticle catalysts

3-1. Preparation of 2-(2-(2-(2-(4-vinylbenzyloxy)ethoxy)ethoxy)ethoxy)ethanol: To sodium hydride (55% in mineral oil, 5.7 g) suspended in THF (150 mL), tetraethyleneglycol (25.2 g) was added at 0 °C. After the reaction mixture was stirred for 1 hour at room temperature, 1-(chloromethyl)-4-vinylbenzene (13.3 g) was added and the mixture was further stirred for 2 h. The mixture was cooled to 0 °C and diluted with diethyl ether. Saturated aqueous ammonium chloride was added to quench the reaction and the aqueous layer was extracted with diethyl ether. The combined organic layers were dried over sodium sulfate and the solvent was removed in vacuo. The residue was purified by column chromatography to afford 2-(2-(2-(2-(4-vinylbenzyloxy)ethoxy)ethoxy)ethoxy)ethanol ether (21.6 g, 68%). ^1H NMR ($CDCl_3$, 600 MHz) δ: 7.38 (2H, d, J = 7.9 Hz), 7.30 (2H, d, J = 7.9 Hz), 6.70 (1H, dd, J = 17.8, 11.1 Hz), 5.74 (1H, d, J = 17.6 Hz), 5.23 (1H, d, J = 10.8 Hz), 4.55 (2H, s), 3.71-3.57 (17H, m), 2.82 (1H, s). ^{13}C NMR ($CDCl_3$, 150 MHz) δ: 137.7, 136.9, 136.4, 127.9, 126.1, 113.7, 72.8, 72.4, 70.53, 70.51, 70.48, 70.2, 69.3, 61.6.

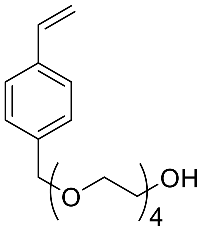

3-2. Preparation of 4-Vinylbenzyl glycidyl ether: To sodium hydride (55% in mineral oil, 17.5 g) suspended in DMF (300 mL), glycidol (33.1 mL) was added at 0 °C. After the reaction mixture was stirred for 1 h at 0 °C, 1-(chloromethyl)-4-vinylbenzene (28.3 mL) was added and the mixture was further stirred for 4 h. The mixture was diluted with diethyl ether at 0 °C. Saturated aqueous ammonium chloride was added to quench the reaction and the aqueous layer was extracted with dichloromethane. The combined organic layers were dried over sodium sulfate and the solvent was removed in vacuo. The residue was purified by column chromatography to afford 4-vinylbenzyl glycidyl ether (20.2 g, 53%). ^{1}H NMR ($CDCl_3$, 400 MHz) δ: 7.39 (2H, d, J = 7.8 Hz), 7.30 (2H, d, J = 7.8 Hz), 6.71 (1H, dd, J = 17.9, 11.0 Hz), 5.74 (1H, d, J = 17.4 Hz), 5.24 (1H, d, J = 11.0 Hz), 4.57 (2H, q, J = 11.8 Hz), 3.76 (1H, dd, J = 11.5, 2.8 Hz), 3.42 (1H, q, J = 5.8 Hz), 3.20-3.15 (1H, m), 2.79 (1H, t, J = 4.6 Hz), 2.61 (1H, q, J = 2.4 Hz). ^{13}C NMR ($CDCl_3$, 100 MHz) δ: 137.4, 137.1, 136.4, 127.9, 126.2, 113.8, 73.0, 70.7, 50.8, 44.2.

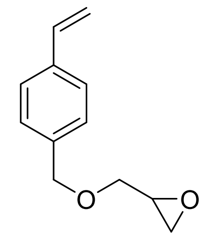

3-3. Preparation of Copolymer: Styrene (0.98 g), 4-vinylbenzyl glycidyl ether (1.8 g), 2-(2-(2-(2-(4-vinylbenzyloxy)ethoxy)ethoxy)ethoxy)ethanol (2.93 g) and 2,2'-azobis(4-methoxy)-2,4-dimethylvaleronitrile (V-70, 92.4 mg) were combined in chloroform (5.5 mL). The mixture was stirred for 48 h at room temperature. The resulting polymer solution was slowly poured into ether. Solvent was removed by decantation and remaining precipitated polymers were washed with ether several times. Polymers were dissolved in THF, repeated to precipitate for 2 times and dried *in vacuo* to afford the desired copolymer (P4, 6.86 g, 49 % yield). The molar ratio of the components was determined by ^{1}H NMR analysis (x: y: z = 40: 25: 35).

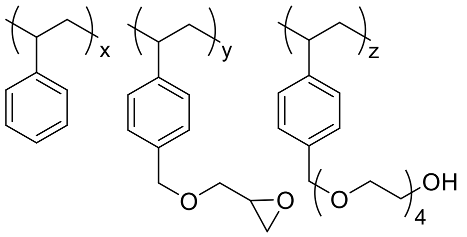

3-4. Typical preparation procedure of PI/CB Rh/Ag:

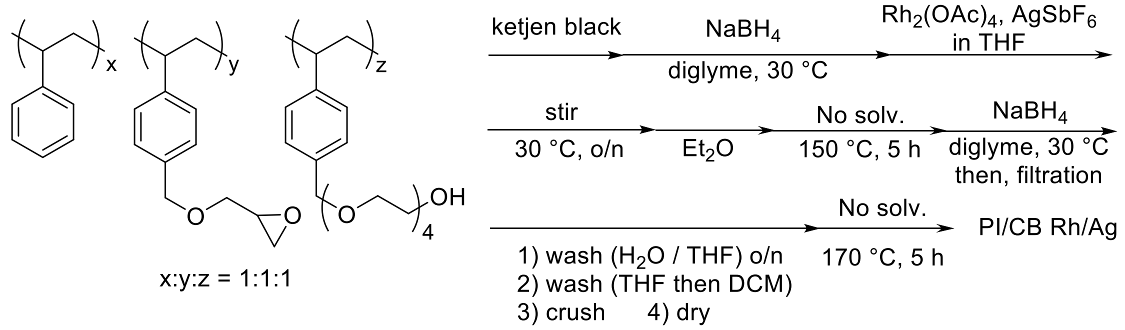

Copolymer (500.0 mg), ketjen black EC300J (500.0 mg) and $NaBH_4$ (113.5 mg) were combined in diglyme (35 mL) at room temperature, to this solution was slowly added rhodium(II) acetate dimer (44.2 mg) and silver hexafluoroantimonate (68.7 mg) with 20 mL of THF. The mixture was stirred overnight at room temperature and diethyl ether (200 mL)

was slowly added to the mixture at room temperature. After the catalysts, which were black powders, were filtered and crashed, they were washed with diethyl ether several times and dried at room temperature. Next, the catalysts were heated at 150 ˚C for 5 h without solvent. The catalysts were transferred to the solution of $NaBH_4$ (113.5 mg) in diglyme (28 mL) and this solution was stirred under air at room temperature for 6 h. The catalysts were filtered and stirred in 1:1 ratio of THF/water co-solvent overnight. The catalysts were filtered, washed with dichloromethane and THF and dried to afford black powder. This powder was heated at 170 ˚C for 5 h without solvent to afford PI/CB Rh/Ag. PI/CB Rh/Ag (10-20 mg) was heated in sulfuric acid (1 mL) at 200 ºC, nitric acid was added until no emission of brown gas was observed. After complete evaporation of nitric acid, the mixture was cooled to room temperature and water was added to dilute to 50 mL solution in a volumetric flask. The amount of Rh and Ag in the resulting solution was measured by ICP analysis to determine the loading of Rh and Ag. Preparations of the PI catalysts with different ratios of Rh/Ag were conducted following this procedure.

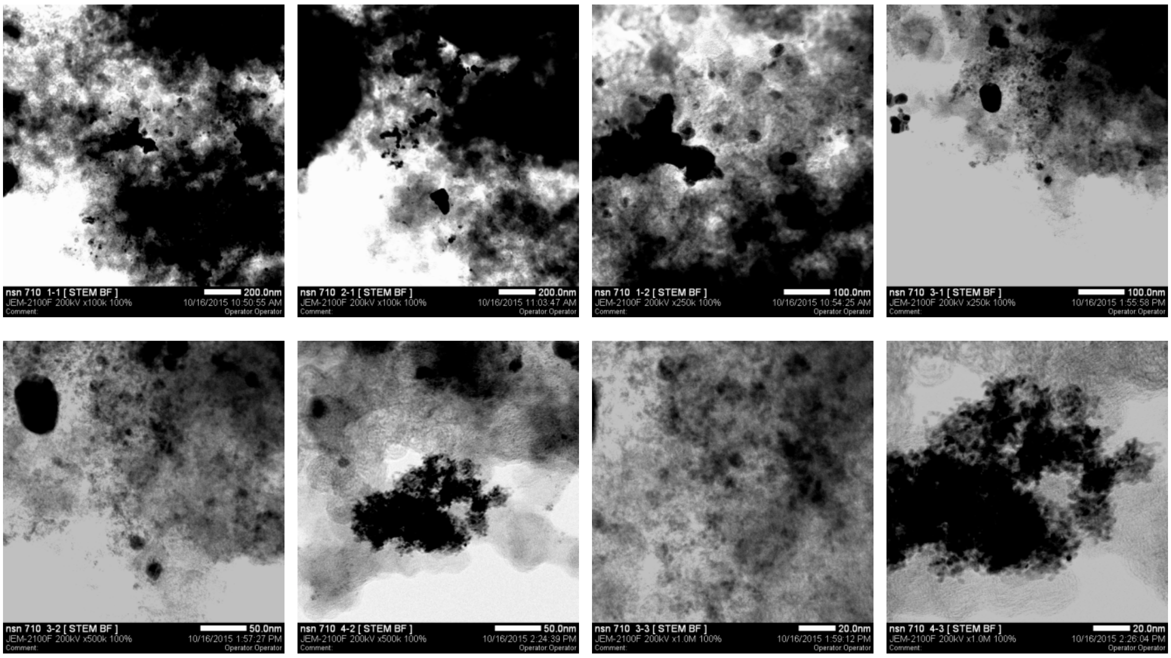

ESI Figure 1. STEM images of PI/CB Rh/Ag.

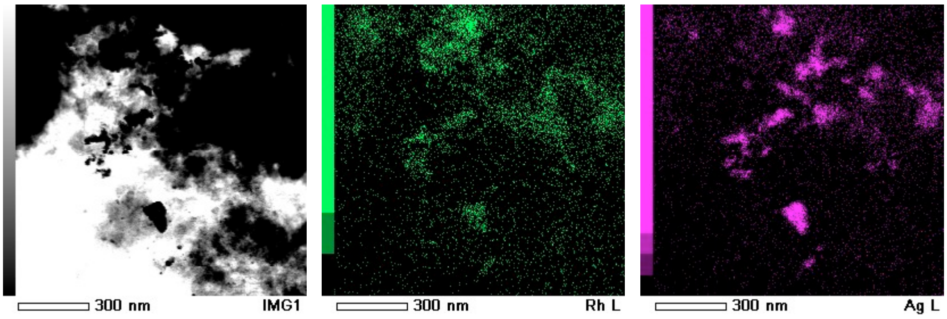

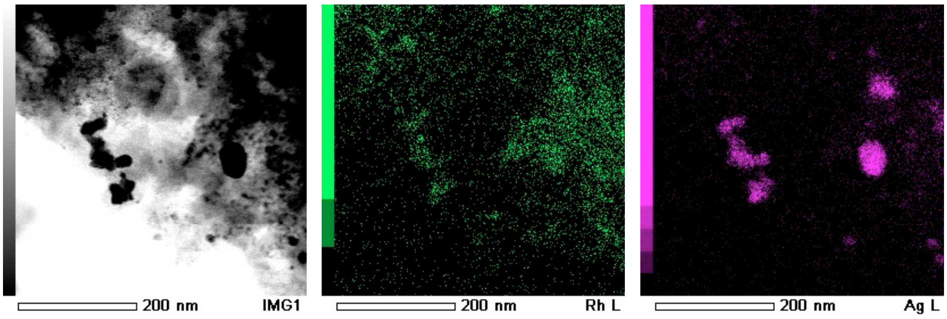

ESI Figure 2. EDS mapping of PI/CB Rh/Ag.

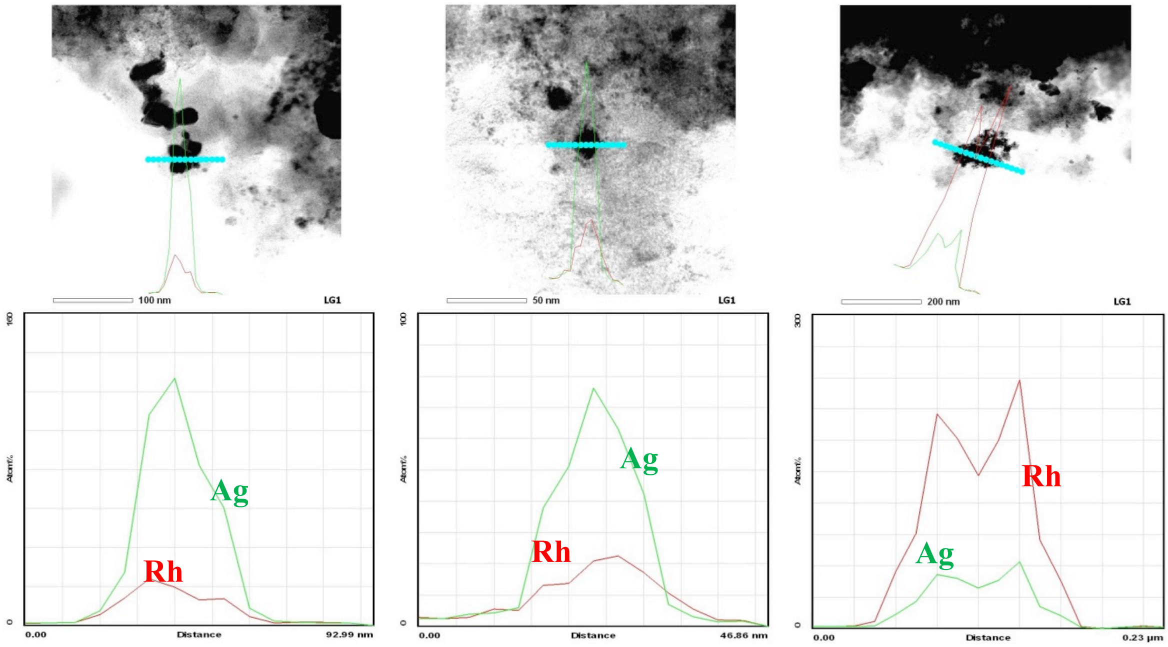

ESI Figure 3. Line analysis of PI/CB Rh/

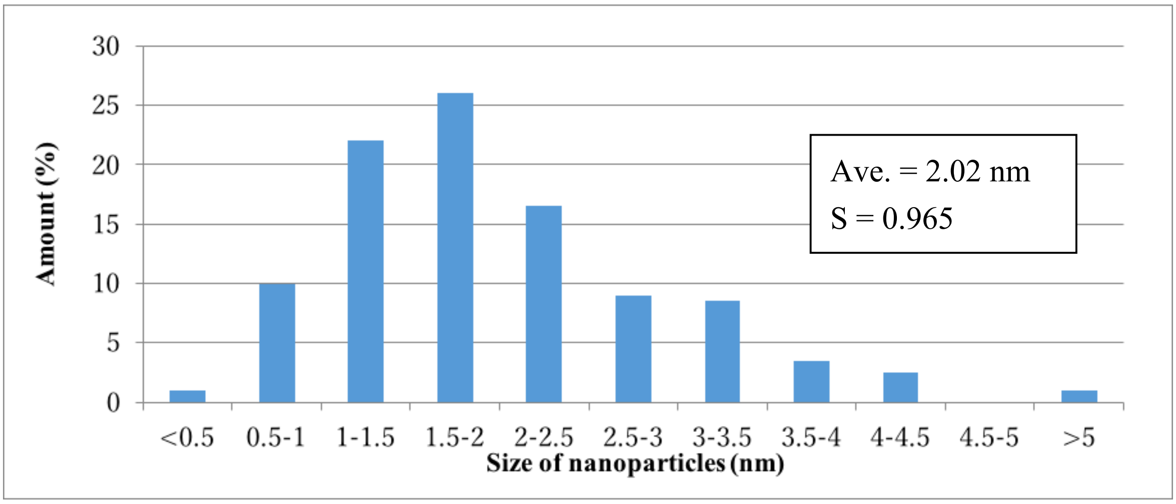

ESI Figure 4. Size distribution of PI/CB Rh/Ag.

4. Preparation of substrates

(*E*)-β-nitrostyrene[4]

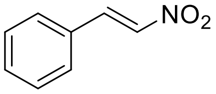

To benzaldehyde (20.2 mL, 0.2 mol) and nitromethane (10.7 mL, 0.2 mol) in MeOH (50 mL) under argon, NaOH (9.0 g, 0.225 mol) in water (30 mL) was slowly added for 30 min at 0 ºC. After stirring for another 30 min at 0 ºC, water (100 mL) was added. The reaction mixture was carefully poured to crushed ice containing concentrated HCl (32 mL). The yellow solids which were precipitated out was filtered, dried in a vacuum desiccator, and recrystallized from hot EtOH to afford 16.5 g of desired product (62% yield) as a yellow needle crystal.

^{1}H NMR ($CDCl_3$, 600 MHz) δ: 8.01 (1H, d, *J* = 13.8 Hz), 7.59 (1H, d, *J* = 13.8 Hz), 7.55 (2H, d, *J* = 7.6 Hz), 7.53-7.48 (1H, m), 7.47-7.44 (2H, m). ^{13}C NMR ($CDCl_3$, 125 MHz) δ: 139.0, 137.1, 132.1, 130.0, 129.4, 129.1.

(*E*)-1-Nitrohex-1-ene[5]

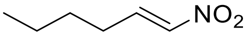

To valeraldehyde (8.6 mL, 80 mmol) and nitromethane (4.5 mL, 84 mmol) in MeOH (20 mL) under argon, NaOH (3.52 g, 88 mmol) in water (10 mL) was slowly added for 30 min at 0 ºC. After stirring for another 30 min at 0 ºC, water (75 mL) and methanol (15 mL) were added. The reaction mixture was carefully poured to 1/1 solution (100 mL) of H_2O and concentrated HCl. The mixture was stirred for 1 hour at at 0 ºC. The mixture was extracted with dichloromethane and the combined organic layers are dried over with Na_2SO_4. The solution was concentrated under vacuum and column chromatography and distillation afforded the desired product as yellow oil.

^{1}H NMR ($CDCl_3$, 600 MHz) δ: 7.31-7.26 (1H, m), 6.99 (1H, d, *J* = 13.1 Hz), 2.28 (2H, q, *J* = 7.6 Hz), 1.53-1.48 (2H, m), 1.39 (2H, td, *J* = 14.8, 7.6 Hz), 0.94 (4H, t, *J* = 7.2 Hz). ^{13}C NMR ($CDCl_3$, 150 MHz) δ: 142.7, 139.5, 29.7, 28.0, 22.1, 13.6.

(*E*)-3-Methyl-1-nitrobut-1-ene[6]

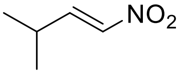

To isobutyraldehyde (7.3 mL, 80 mmol) and nitromethane (4.5 mL, 84 mmol) in MeOH (20 mL) under argon, NaOH (3.52 g, 88 mmol) in water (10 mL) was slowly added for 30 min at 0 ºC. After stirring for another 30 min at 0 ºC, water (75 mL) and methanol (15 mL) were added. The reaction mixture was carefully poured to 1/1 solution (100 mL) of H_2O and concentrated HCl. The mixture was stirred for 1 hour at at 0 ºC. The mixture was extracted with dichloromethane and the combined organic layers are dried over with Na_2SO_4. The solution was concentrated under vacuum and column chromatography and distillation afforded 3.1 g of desired product (34% yield) as yellow oil.

^{1}H NMR ($CDCl_3$, 400 MHz) δ: 7.27 (1H, q, *J* = 6.9 Hz), 6.96 (1H, d, *J* = 13.3 Hz), 2.62-2.59 (1H, m), 1.17 (6H, d, *J* = 6.9 Hz). ^{13}C NMR ($CDCl_3$, 100 MHz) δ: 148.4, 138.1, 28.3, 21.0.

(*E*)-(2-Nitrovinyl)cyclohexane[7]

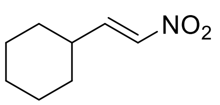

To cyclohexanecarboxaldehyde (4.85 mL, 40 mmol) and nitromethane (2.14 mL, 40 mmol) in MeOH (10 mL) under argon, NaOH (1.76 g, 44 mmol) in water (5 mL) was slowly added for 30 min at 0 ºC. After stirring for another 30 min at 0 ºC, water (37.5 mL) and methanol (7.5) were added. The reaction mixture was carefully poured to 1/1 solution (50 mL) of H_2O and concentrated HCl. The mixture was stirred for 1 hour at at 0 ºC. The mixture was extracted with dichloromethane and the combined organic layers are dried over with Na_2SO_4. The solution was concentrated under vacuum and column chromatography afforded desired product as yellow oil.

^{1}H NMR ($CDCl_3$, 600 MHz) δ: 7.20 (1H, q, J = 6.9 Hz), 6.91 (1H, d, J = 13.1 Hz), 2.27-2.21 (1H, m), 1.82-1.75 (4H, m), 1.72-1.67 (1H, m), 1.36-1.27 (2H, m), 1.24-1.15 (3H, m). ^{13}C NMR ($CDCl_3$, 150 MHz) δ: 147.2, 138.3, 37.6, 31.5, 25.6, 25.6.

(*m*- or *p*-)Methoxyphenylboronic acid

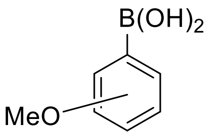

To Mg (1.34g, 55 mmol) suspended in THF (4 mL) in 100 mL 3-necked round bottom flask, (*m*- or *p*-)bromoanisole (9.35g, 50 mmol) in THF (40 mL) was carefully added dropwise to prepare Grinard reagent. After stirring for 3 h at room temperature, this Grinard reagent was transferred to the dropping funnel by cannula and slowly added to trimethoxyborate (6.7 mL, 60 mmol) in THF (40 mL) at -78 ºC. After stirring over night at room temperature, 1N HCl (150 mL) was added, ant the aqueous layer was extracted with extracted with CH_2Cl_2. The combined organic layers were dried over Na_2SO_4, the solvent was removed *in vacuo*. The residue was purified by recrystallization (hexane/EtOAc) to afford mixture of boronic acid and boroxine.

Tris(3-methoxyphenyl)Boroxine[8]

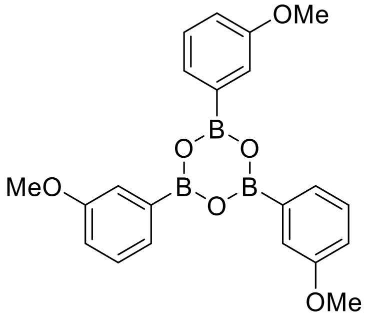

The ratio of boronic acid to boroxine was determined by ^{1}H NMR analysis. ^{1}H NMR ($CDCl_3$, 500 MHz) δ: 7.83 (1H, d, J = 7.4 Hz), 7.75 (1H, d, J = 2.3 Hz), 7.45 (1H, t, J = 7.9 Hz), 7.15 (1H, dd, J = 7.9, 2.8 Hz), 3.92 (3H, s). ^{13}C NMR ($CDCl_3$, 150MHz) δ: 159.2, 129.2, 128.0, 120.4, 118.4, 55.3. These peaks derive from boroxine.

Tris(4-methoxyphenyl)Boroxine[8]

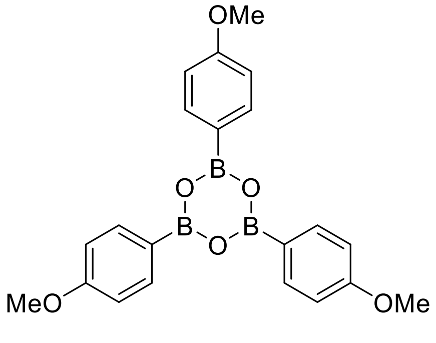

The ratio of boronic acid to boroxine was determined by ^{1}H NMR analysis. ^{1}H NMR ($CDCl_3$, 600 MHz) δ: 8.16 (2H, d, J = 8.3 Hz), 7.01 (2H, d, J = 8.3 Hz), 3.89 (3H, s). ^{13}C NMR ($CDCl_3$, 150MHz) δ: 163.2, 137.5, 113.5, 55.2. These peaks derive from boroxine.

5. Asymmetric 1,4-addition reactions

5-1. Preparation of a stock solution of Rh complex with chiral diene: To chiral diene **4c** (0.00165 mmol, 0.110 mL of 3.9 mg/mL solution of toluene) in toluene (0.3 mL), chlorobis(ethylene)rhodium(I) dimer (0.00075 mmol, 0.09 mL of 3.2 mg/mL solution of toluene) was added at room temperature. The mixture was stirred at room temperature over 30 minutes. Aliquot of the obtained solution (0.5 mL) was taken for asymmetric 1,4-addition reaction in homogeneous system.

5-2. A typical procedure of asymmetric 1,4-addition of arylboronic acids to nitroolefins in homogeneous system: A stirring bar, 3-methoxyphenylboronic acid (0.45 mmol as B) and (*E*)-β-nitrostyrene (44.7 mg, 0.3 mmol) were combined in a reaction tube, then toluene (900 μL) and water (1000 μL) were added to the mixture. This tube was flushed with argon and sealed up with a septum. Rhodium-chiral diene complex (0.1 mol%) in toluene (100 μL) was added by gas-tight syringe and the mixture was stirred at 100 ºC for 16 h under reflux conditions. After refluxing, the reaction mixture was diluted with EtOAc, washed with brine and dried over with Na_2SO_4. The solid was filtered and the filtrate was concentrated under vacuum. The conversion was determined by ^{1}H NMR analysis with reference to an internal standard 1,1,2,2-tetrachloroethane. The solution was concentrated under vacuum again and the residue was purified by preparative TLC (toluene/hexane = 2/1) to afford 76.3 mg of 1-methoxy-3-(2-nitro-1-phenylethyl)benzene (99% yield) as a light yellow oil. The enantioselectivity of the desired compound was determined as 91% ee by HPLC analysis on a chiralcel OD-H with hexane/2-propanol = 1/1, flow = 1.0 mL/min.

5-3: A typical procedure of asymmetric 1,4-addition of arylboronic acids to nitroolefins in heterogeneous system: A stirring bar, PI/CB Rh/Ag(1/1) (13.6 mg, 1 mol% as Rh), 3-methoxyphenylboronic acid (0.45 mmol as B), (*E*)-β-nitrostyrene (44.7 mg, 0.3 mmol), and chiral diene **4c** (0.1 mol% in 20 μL toluene) were combined in a reaction tube, then toluene (1980 μL) and water (400 μL) were added to the mixture. This tube was flushed with argon and sealed up with a septum. The mixture was stirred at 100 ºC for 24 h under reflux conditions. After refluxing, THF was added to the mixture. The mixture was picked up with syringe and transferred to volumetric flask through membrane filter in order to remove the residual solids and diluted to 25 mL solution by THF. The filtrate (5 mL) was taken by volumetric pipette and the solvent was removed *in vacuo*. Residual crude mixture was heated in mixture of sulfuric acid (0.2 mL) and nitric acid at 200 °C until all nitric acid was removed, the mixture was cooled to room temperature and aqua regia (0.2 mL) was added. The solution was diluted to 10 mL solution by pure water and the resulting solution was measured by ICP analysis to determine the amount of Rh that leached out. Another filtrate (20 mL) was diluted with EtOAc, washed with brine and dried over with Na_2SO_4. The solid was filtered and the

filtrate was concentrated under vacuum. The conversion was determined by ^{1}H NMR analysis with reference to an internal standard 1,1,2,2-tetrachloroethane. The solution was concentrated under vacuum again and the residue was purified by preparative TLC (toluene/hexane = 2/1) to afford 54.6 mg of 1-methoxy-3-(2-nitro-1-phenylethyl)benzene (90% yield) as a light yellow oil. The enantioselectivity of the desired product was determined as 92% ee by HPLC analysis on a chiralcel OD-H with hexane/2-propanol = 1/1, flow = 1.0 mL/min.

5-4: A typical procedure of recovery and reuse of PI/CB Rh/Ag: A reaction was conducted following the procedure as previously mentioned. After the reaction, the catalyst was separated by the filtration. The filtrate was diluted with EtOAc and washed with brine. The organic layer was dried over with Na_2SO_4 and column chromatography afforded the desired product. The filtered catalyst was washed with EtOAc, water and acetone successively on the funnel. The catalyst was washed in 9/1 solution of water/0.5N HCl aq. overnight and filtered. In this procedure, around 5 mg of the catalyst was trapped on the filtrate paper, so the amount of the catalyst gradually decreased. The corrected catalyst was dried under vacuum. Based on the weight of the dried catalyst, the scale of next reaction was calculated.

6. Reaction profiles in toluene/H_2O = 1/1

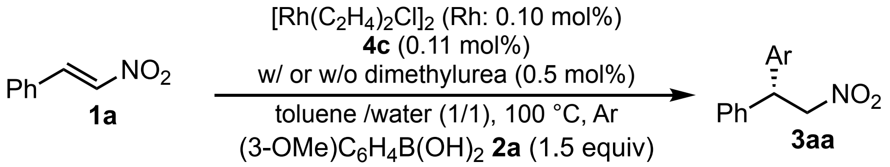

Obtaine reaction profiles in toluene/H_2O = 1/1 were almost same regardless of the presence of dimethylurea.

6.1 In the presence of dimethyl urea

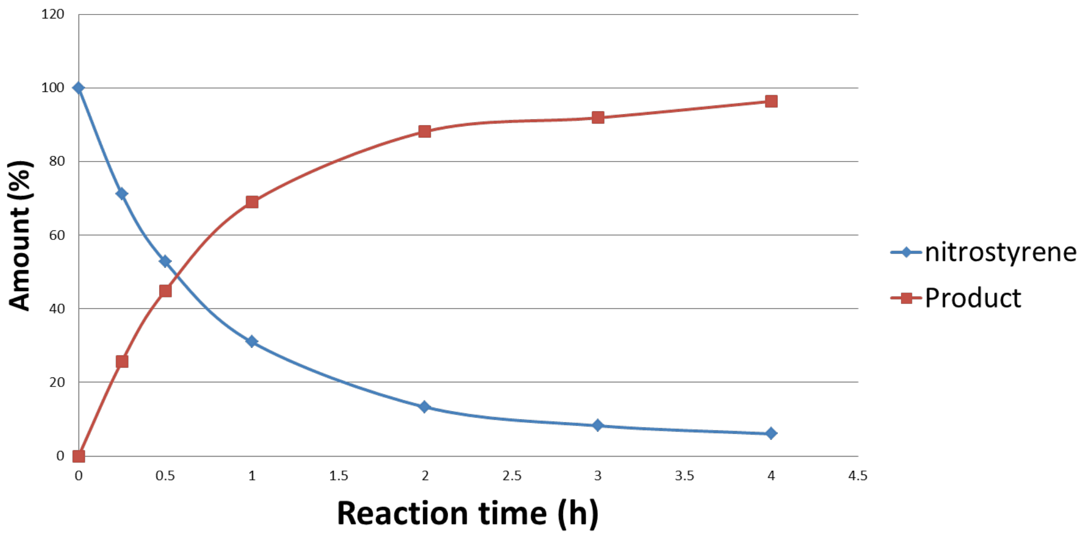

ESI Figure 5

6.2 In the absence of dimethyl urea

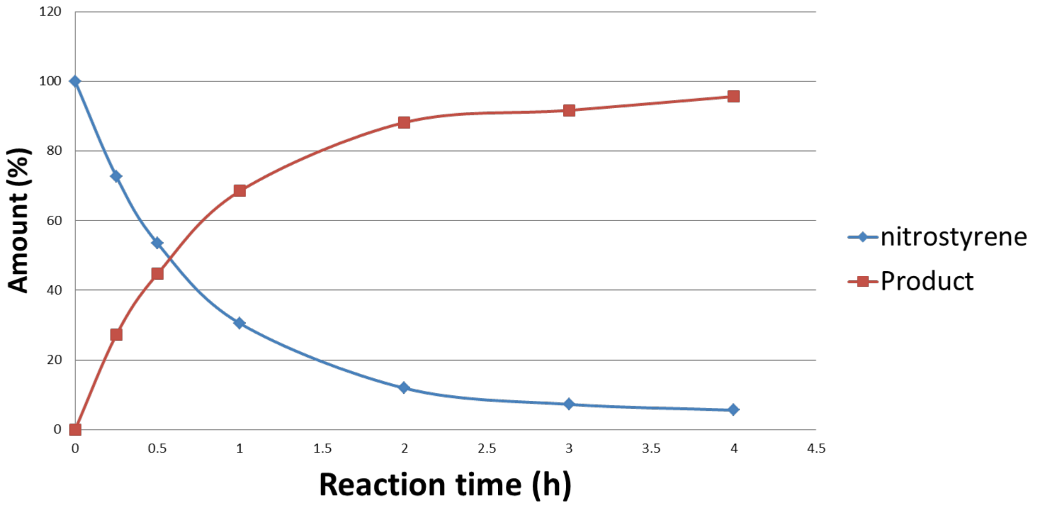

ESI Figure 6

7. Reaction profiles during recovery and reuse, after hot filtration

7.1 Reaction profiles during recovery and reuse (1st to 3rd run)

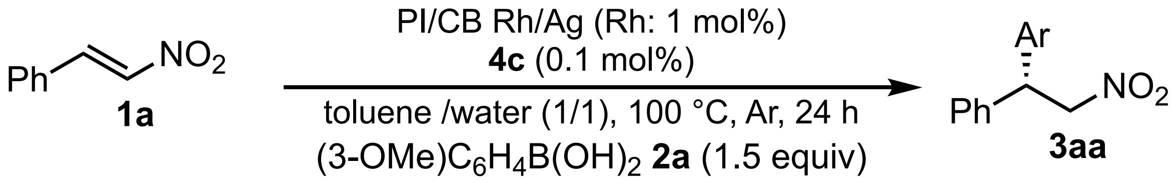

Durene (~50 mg) was added in the reaction mixture as internal standard for GC analysis.

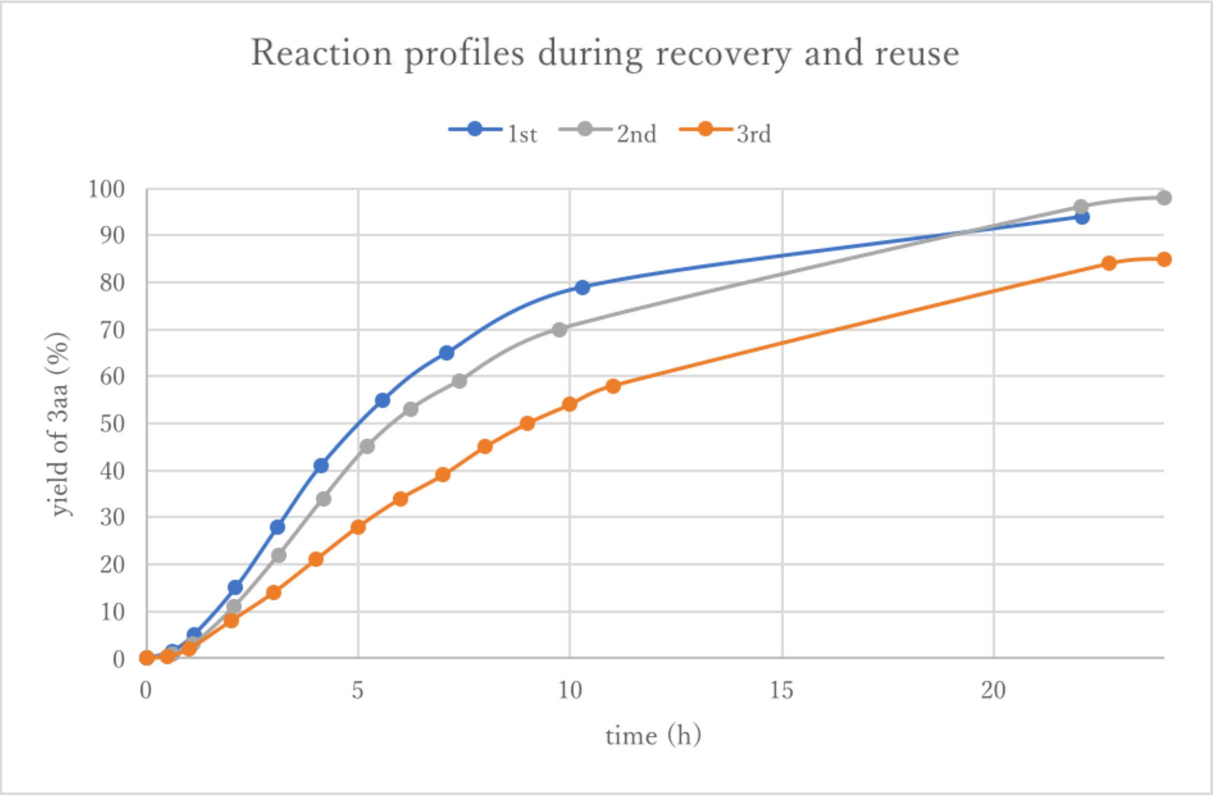

ESI Figure 7. Reaction profiles during recovery and reuse

7.2 Reaction profiles after hot filtration at 4 h and further 2 runs with 24 h reaction time (1st to 3rd run)

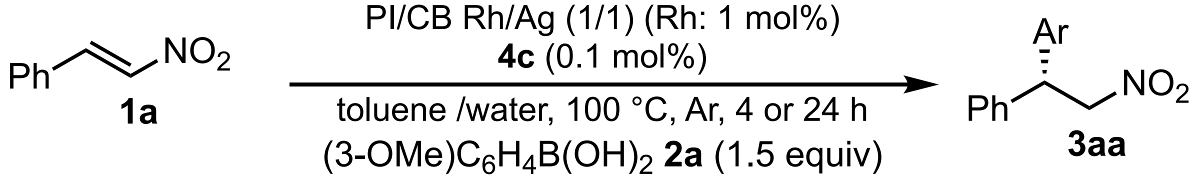

Durene (~50 mg) was added in the reaction mixture as internal standard for GC analysis.

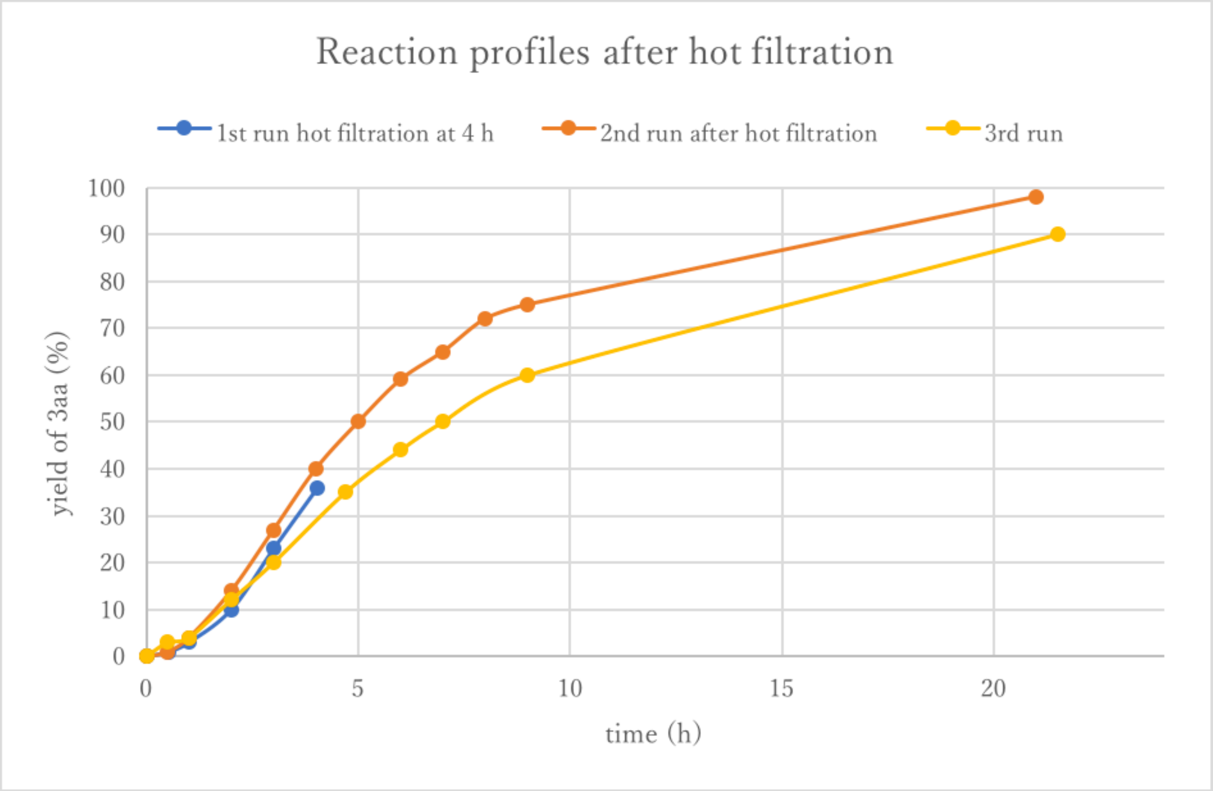

ESI Figure 8. Reaction profiles after hot filtration at 4 h and further 2 runs with 24 h reaction time

7.3 Reaction profiles after hot filtration at 6 h (1st to 3rd run)

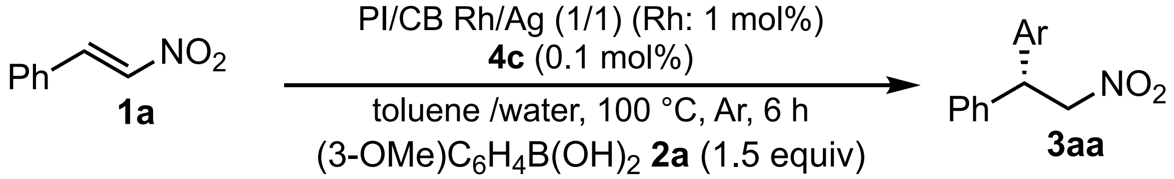

Durene (~50 mg) was added in the reaction mixture as internal standard for GC analysis.

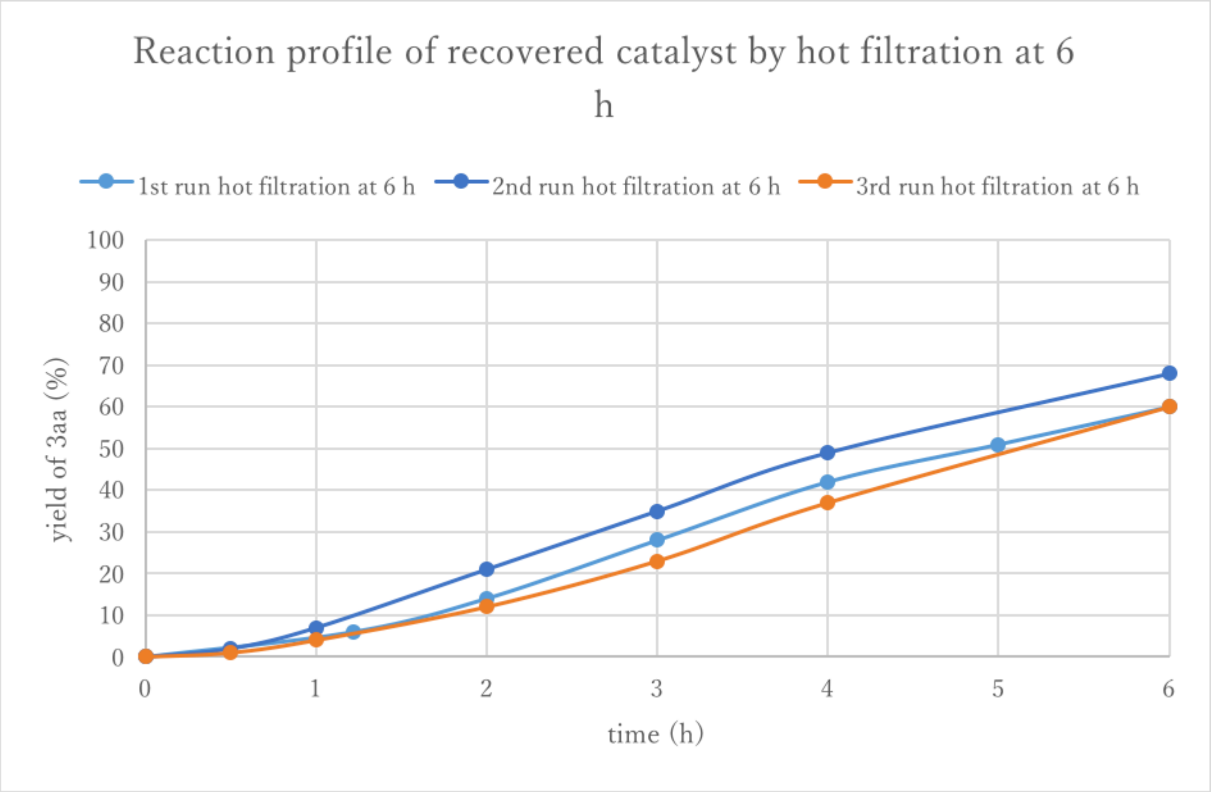

ESI Figure 9. Reaction profiles after hot filtration at 6 h

8. XPS analysis

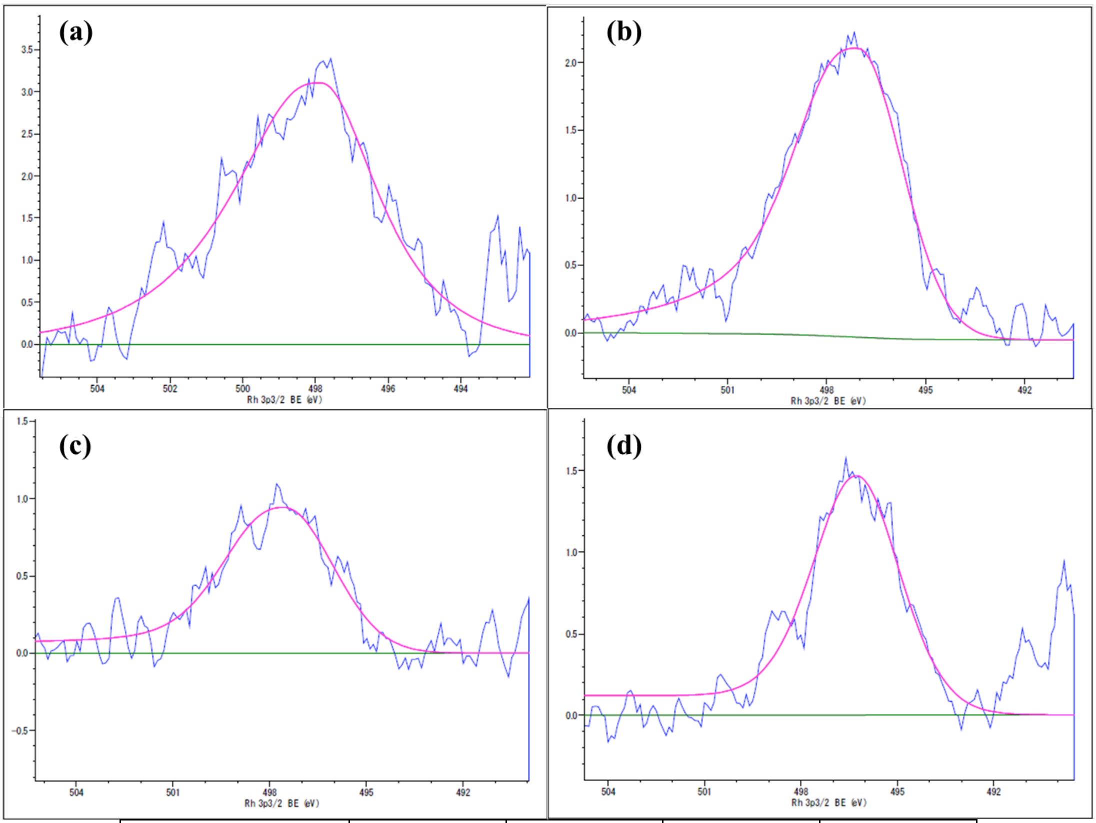

chart	(a)	(b)	(c)	(d)

peak top (eV)	497.859	497.060	497.564	496.282

ESI Figure 10. Rh 3p3/2 XPS spectrum of (a) fresh catalyst (b) catalyst collected by hot filtration during induction period (at 1 h) (c) catalyst collected by hot filtration during induction period (at 5 h) (d) catalyst after recovery reuse

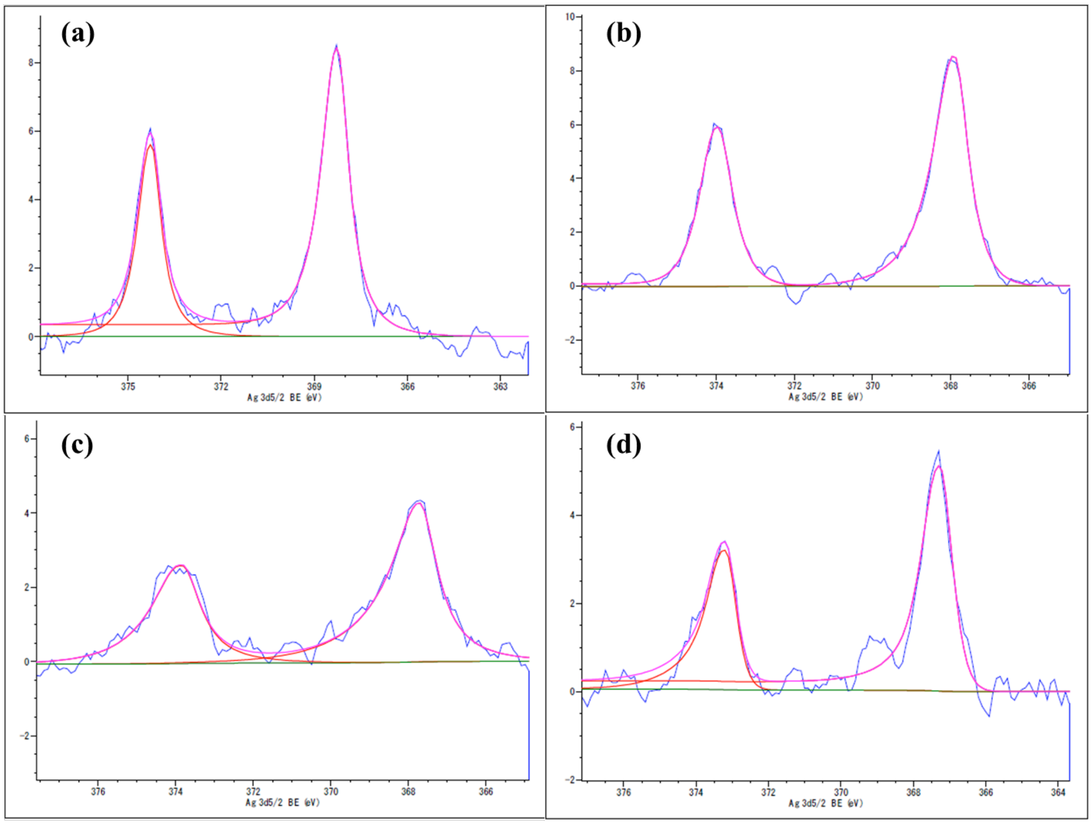

chart	(a)	(b)	(c)	(d)
peak top 3d5/2 (eV)	368.264	367.913	367.720	367.279
peak top 3d3/2 (eV)	374.258	373.965	373.833	373.193

ESI Figure 11. Ag 3d5/2, 3/2 XPS spectrum of (a) fresh catalyst (b) catalyst collected by hot filtration during induction period (at 1 h) (c) catalyst collected by hot filtration during induction period (at 5 h) (d) catalyst after recovery reuse

9. Products

(*R*)-1-Methoxy-3-(2-nitro-1-phenylethyl)benzene (3aa, ent-3cb)[6, 7, 8]

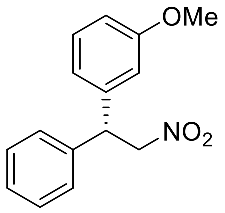

The enantio excess was determined by HPLC analysis on a chiralcel OD-H with hexane/2-propanol = 1/1, flow = 1.0 mL/min. ^{1}H NMR ($CDCl_3$, 500 MHz) δ: 7.34-7.30 (2H, m), 7.27-7.23 (4H, m), 6.84-6.76 (3H, m), 4.98-4.96 (2H, m), 4.89-4.86 (1H, m), 3.77 (3H, s). ^{13}C NMR

($CDCl_3$, 125 MHz) δ: 159.9, 140.7, 139.0, 130.0, 129.0, 127.6, 119.8, 114.0, 112.5, 79.1, 55.2, 48.9.

(*R*)-1-Methoxy-4-(2-nitro-1-phenylethyl)benzene (3ac, ent-3bb)[5, 6, 7, 8]

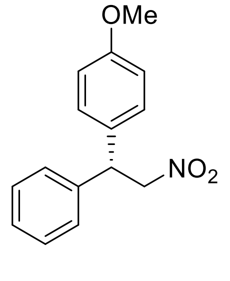

The enantio excess was determined by HPLC analysis on a chiralcel OD-H with hexane/2-propanol = 3/2, flow = 1.0 mL/min. ^{1}H NMR ($CDCl_3$, 600 MHz) δ: 7.32 (2H, t, *J* = 7.6 Hz), 7.26-7.21 (3H, m), 7.15 (2H, d, *J* = 8.3 Hz), 6.85 (2H, dt, *J* = 8.9, 2.8 Hz), 4.95 (2H, dd, *J* = 7.6, 2.1 Hz), 4.86 (1H, t, *J* = 8.3 Hz), 3.77 (3H, s). ^{13}C NMR ($CDCl_3$, 150 MHz) δ: 158.9, 139.5, 131.1, 129.0, 128.7, 127.53, 127.48, 114.4, 79.4, 55.2, 48.2.

(*R*)-1-Chloro-4-(2-nitro-1-phenylethyl)benzene (3ad)[5, 6, 7]

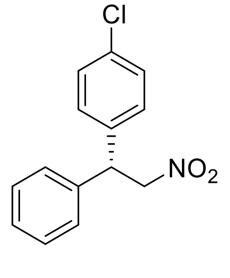

The enantio excess was determined by HPLC analysis on a chiralcel OD-H with hexane/2-propanol = 3/2, flow = 1.0 mL/min. ^{1}H NMR ($CDCl_3$, 600 MHz) δ: 7.35-7.25 (5H, m), 7.21-7.16 (4H, m), 4.95 (2H, d, *J* = 7.6 Hz), 4.88 (1H, t, *J* = 8.2 Hz). ^{13}C NMR ($CDCl_3$, 150 MHz) δ: 138.7, 137.7, 133.6, 129.2, 129.1, 129.0, 127.8, 127.5, 79.0, 48.3.

(*R*)-1-Methyl-4-(2-nitro-1-phenylethyl)benzene (3af, ent-3bd)[5, 6, 7, 8]

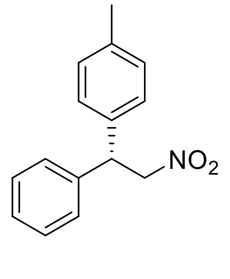

The enantio excess was determined by HPLC analysis on a chiralcel OD-H with hexane/2-propanol = 3/2, flow = 1.0 mL/min. ^{1}H NMR ($CDCl_3$, 600 MHz) δ: 7.31 (2H, t, *J* = 7.6 Hz), 7.25-7.20 (3H, m), 7.14-7.09 (4H, m), 4.95 (2H, d, *J* = 7.6 Hz), 4.86 (1H, t, *J* = 8.2 Hz), 2.30 (3H, s). ^{13}C NMR ($CDCl_3$, 150 MHz) δ: 139.5, 137.3, 136.2, 129.7, 129.0, 127.6, 127.51, 127.48, 79.4, 48.6, 21.0.

(*R*)-1-Methoxy-2-(2-nitro-1-phenylethyl)benzene (3ag)[6, 7]

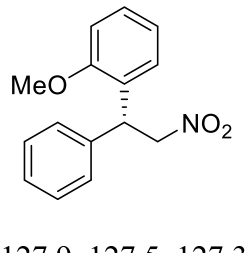

The enantio excess was determined by HPLC analysis on a chiralcel OD-H with hexane/2-propanol = 3/2, flow = 1.0 mL/min. ^{1}H NMR ($CDCl_3$, 600 MHz) δ: 7.33-7.22 (6H, m), 7.06 (1H, dd, *J* = 7.6, 1.4 Hz), 6.91-6.87 (2H, m), 5.29-5.25 (1H, m), 5.05-4.94 (2H, m), 3.83 (3H, s). ^{13}C NMR ($CDCl_3$, 150 MHz) δ: 156.8, 138.9, 128.74, 128.72, 128.4, 127.9, 127.5, 127.3, 120.8, 111.0, 77.9, 55.5, 43.3.

(*R*)-1-Methyl-2-(2-nitro-1-phenylethyl)benzene (3ah)[5]

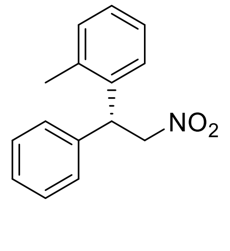

The enantio excess was determined by HPLC analysis on a chiralcel OD-H with hexane/2-propanol = 3/2, flow = 1.0 mL/min. ^{1}H NMR

($CDCl_3$, 600 MHz) δ: 7.30 (2H, t, *J* = 7.6 Hz), 7.26-7.17 (7H, m), 5.11 (1H, t, *J* = 7.9 Hz), 5.00-4.90 (2H, m), 2.31 (3H, s). ^{13}C NMR ($CDCl_3$, 150 MHz) δ: 138.7, 137.1, 136.5, 131.3, 128.9, 128.0, 127.49, 127.48, 126.4, 125.8, 79.2, 45.0, 19.6.

(*S*)-1-Methoxy-3-(1-(4-methoxyphenyl)-2-nitroethyl)benzene (3ba) [6]

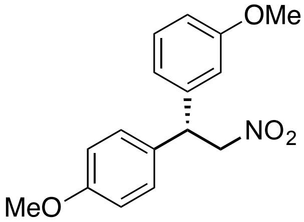

The enantio excess was determined by HPLC analysis on a chiralcel OD-H with hexane/2-propanol = 1/1, flow = 1.0 mL/min. ^{1}H NMR ($CDCl_3$, 600 MHz) δ: 7.26-7.22 (1H, m), 7.15 (2H, d, *J* = 8.9 Hz), 6.86-6.74 (5H, m), 4.94-4.91 (2H, m), 4.82 (1H, t, *J* = 8.2 Hz), 3.77 (6H, s), 3.76 (6H, s).^{13}C NMR ($CDCl_3$, 150 MHz) δ: 160.0, 158.9, 141.1, 131.1, 130.0, 128.7, 119.7, 114.4, 113.9, 112.4, 79.4, 55.24, 55.19, 48.2.

(*R*)-1-Methoxy-3-(2-nitro-1-(*p*-tolyl)ethyl)benzene (3ea)[9]

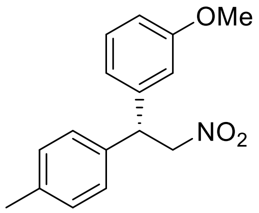

The enantio excess was determined by HPLC analysis on a chiralcel OD-H with hexane/2-propanol = 3/2, flow = 1.0 mL/min. ^{1}H NMR ($CDCl_3$, 600 MHz) δ: 7.25-7.21 (1H, m), 7.12 (4H, s), 6.83-6.75 (3H, m), 4.94 (2H, d, *J* = 8.2 Hz), 4.83 (1H, t, *J* = 7.9 Hz), 3.76 (3H, s), 2.30 (3H, s). ^{13}C NMR ($CDCl_3$, 150 MHz) δ: 160.0, 141.0, 137.3, 136.1, 130.0, 129.7, 127.5, 119.8, 113.9, 112.5, 79.3, 55.2, 48.6, 21.0.

(*S*)-1-Bromo-4-(2-nitro-1-phenylethyl)benzene (3fb)[5]

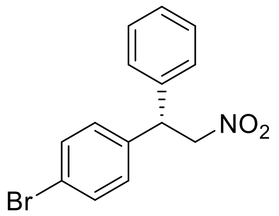

The enantio excess was determined by HPLC analysis on a chiralcel OD-H with hexane/2-propanol = 3/2, flow = 0.7 mL/min. ^{1}H NMR ($CDCl_3$, 600 MHz) δ: 7.46-7.44 (2H, m), 7.35-7.31 (2H, m), 7.28-7.25 (1H, m), 7.20 (2H, d, *J* = 6.9 Hz), 7.13-7.10 (2H, m), 4.95 (2H, d, *J* = 8.2 Hz), 4.86 (1H, t, *J* = 8.2 Hz). ^{13}C NMR ($CDCl_3$, 150 MHz) δ: 138.6, 138.2, 132.2, 129.4, 129.2, 127.8, 127.5, 121.6, 78.9, 48.4.

(*R*)-1-(1-(4-Bromophenyl)-2-nitroethyl)-3-methoxybenzene (3fa)

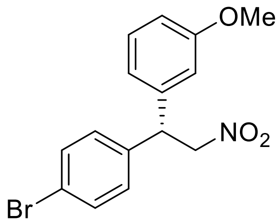

The enantio excess was determined by HPLC analysis on a chiralcel OD-H with hexane/2-propanol = 1/1, flow = 1.0 mL/min. $[\alpha]^{16}_{D}$ = +3.5 (c = 1.46, $CHCl_3$, as 90% ee). IR (neat) cm^{-1}: 2938, 2837, 1601, 1586, 1553, 1490, 1454, 1435, 1407, 1377, 1319, 1294, 1263, 1154, 1074, 1041, 1011, 828, 778, 722, 696, 664. ^{1}H NMR ($CDCl_3$, 600 MHz) δ: 7.46-7.44 (2H, m), 7.27-7.23 (1H, m), 7.11 (2H, d, *J* = 8.2 Hz), 6.81-6.77 (2H, m), 6.72 (1H, t, *J* = 2.1 Hz), 4.93 (2H, d, *J* = 8.9 Hz), 4.83 (1H, t, *J* = 8.2 Hz), 3.77 (3H, s). ^{13}C NMR ($CDCl_3$, 150 MHz) δ: 160.1, 140.1, 138.1, 132.2, 130.2, 129.3, 121.7, 119.7, 114.0, 112.7, 78.8, 55.2, 48.3. HRMS (DART) calculated for $C_{15}H_{15}BrNO_3^+$ [M+H^+] 336.02353, found 336.02376.

(*S*)-1-Fluoro-4-(2-nitro-1-phenylethyl)benzene (3gb, ent-3ae)[6, 7, 8]

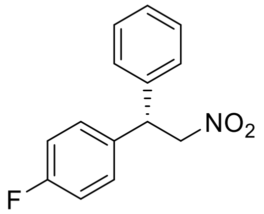

The enantio excess was determined by HPLC analysis on a chiralcel OD-H with hexane/2-propanol = 3/2, flow = 1.0 mL/min. ^{1}H NMR ($CDCl_3$, 600 MHz) δ: 7.33 (t, 2H, *J* = 7.6 Hz), 7.28-7.24 (m, 1H), 7.22-7.19 (m, 4H), 7.03-6.99 (m, 2H), 4.96-4.93 (m, 2H), 4.90 (q, 1H, *J* = 8.5 Hz). ^{13}C NMR ($CDCl_3$, 150 MHz) δ: 162.1 (d, *J* = 245.6 Hz), 139.0, 135.0, 129.3 (d, *J* = 8.6 Hz), 129.1, 127.7, 127.5, 115.9 (d, *J* = 21.5 Hz), 79.3, 48.2.

(*R*)-1-(1-(4-Fluorophenyl)-2-nitroethyl)-3-methoxybenzene (3ga)

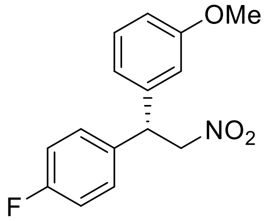

The enantio excess was determined by HPLC analysis on a chiralcel OD-H with hexane/2-propanol = 1/1, flow = 1.0 mL/min. $[\alpha]^{16}_D$ = -8.2 (c = 1.04, $CHCl_3$, as 91% ee). IR (neat) cm^{-1}: 3007, 2940, 2920, 2839, 1604, 1586, 1554, 1510, 1491, 1455, 1436, 1377, 1321, 1264, 1228, 1161, 1104, 1042, 875, 838, 803, 781, 739, 721, 697. ^{1}H NMR ($CDCl_3$, 600 MHz) δ: 7.25 (1H, t, *J* = 8.2 Hz), 7.22-7.19 (2H, m), 7.01 (2H, t, *J* = 8.2 Hz), 6.80 (2H, d, *J* = 8.2 Hz), 6.74-6.72 (1H, m), 4.93 (2H, q, *J* = 4.1 Hz), 4.85 (1H, t, *J* = 7.9 Hz), 3.77 (3H, s). ^{13}C NMR ($CDCl_3$, 150 MHz) δ: 162.1 (d, *J* = 245.6 Hz), 160.1, 140.5, 134.9, 130.1, 129.3 (d, *J* = 7.2 Hz), 119.7, 115.9 (d, *J* = 21.5 Hz), 114.0, 112.6, 79.2, 55.2, 48.1. HRMS (DART) calculated for $C_{15}H_{15}FNO_3^+$ [*M*+H$^+$] 276.10360, found 276.10470.

(*S*)-3-(2-Nitro-1-phenylethyl)furan (3hb)

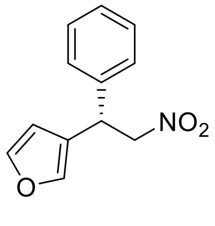

The enantio excess was determined by HPLC analysis on a chiralcel OD-H with hexane/2-propanol = 3/2, flow = 1.0 mL/min. $[\alpha]^{16}_D$ = +35.4 (c = 0.72, $CHCl_3$, as 94% ee). IR (neat) cm^{-1}: 3608, 3583, 3146, 3064, 3032, 2919, 1766, 1553, 1498, 1454, 1432, 1379, 1199, 1160, 1076, 1026, 875, 792, 771, 732, 703, 665, 600. ^{1}H NMR ($CDCl_3$, 400 MHz) δ: 7.38 (1H, t, *J* = 1.8 Hz), 7.37-7.23 (6H, m), 6.26-6.24 (1H, m), 4.87-4.74 (3H, m). ^{13}C NMR ($CDCl_3$, 150 MHz) δ: 143.8, 139.5, 138.5, 129.0, 127.9, 127.6, 123.9, 109.7, 79.6, 40.9. HRMS (DART) calculated for $C_{12}H_{12}NO_3^+$ [*M*+H$^+$] 218.08172, found 218.08162.

(*S*)-3-(1-(3-Methoxyphenyl)-2-nitroethyl)furan (3ha)

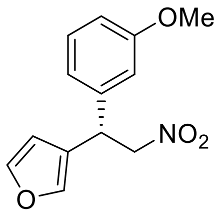

The enantio excess was determined by HPLC analysis on a chiralcel OD-H with hexane/2-propanol = 3/2, flow = 1.0 mL/min. $[\alpha]^{16}_D$ = +23.9 (c = 0.78, $CHCl_3$, as 95% ee). IR (neat) cm^{-1}: 3145, 3006, 2920, 2838, 1766, 1602, 1553, 1493, 1455, 1436, 1379, 1322, 1262, 1198, 1158, 1028, 874, 782, 733, 705. ^{1}H NMR ($CDCl_3$, 400 MHz) δ: 7.38 (1H, t, *J* = 1.6 Hz), 7.28-7.23 (2H, m), 6.85-6.77 (3H, m), 6.26 (1H, s), 4.86-4.70 (3H, m), 3.78 (3H,s). ^{13}C

NMR ($CDCl_3$, 150 MHz) δ: 160.0, 143.7, 140.1, 139.5, 130.1, 123.8, 119.8, 113.9, 112.9, 109.7, 79.5, 55.2, 40.8. HRMS (DART) calculated for $C_{13}H_{14}NO_4^+$ [M+H$^+$] 248.09228, found 248.09289.

(*R*)-2-(2-Nitro-1-phenylethyl)thiophene (3ib)[10]

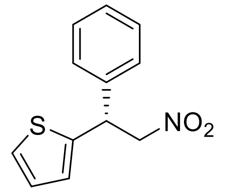

The enantio excess was determined by HPLC analysis on a chiralcel OD-H with hexane/2-propanol = 3/2, flow = 1.0 mL/min. ^{1}H NMR ($CDCl_3$, 600 MHz) δ: 7.27 (t, 2H, J = 7.6 Hz), 7.23-7.20 (3H, m), 7.13 (1H, d, J = 4.8 Hz), 6.87-6.82 (2H, m), 5.05 (1H, t, J = 7.9 Hz), 4.92-4.82 (2H, m). ^{13}C NMR ($CDCl_3$, 150 MHz) δ: 142.4, 138.8, 129.1, 128.1, 127.5, 127.0, 125.2, 125.1, 79.9, 44.7.

(*R*)-2-(1-(3-Methoxyphenyl)-2-nitroethyl)thiophene (3ia)

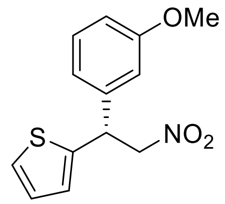

The enantio excess was determined by HPLC analysis on a chiralcel OD-H with hexane/2-propanol = 3/2, flow = 1.0 mL/min. $[\alpha]^{16}_D$ = -11.6 (c = 1.13, $CHCl_3$, as 94% ee). IR (neat) cm^{-1}: 3426, 3109, 3072, 3006, 2960, 2939, 2919, 2837, 1602, 1587, 1553, 1491, 1455, 1435, 1377, 1319, 1292, 1264, 1201, 1161, 1083, 1044, 994, 907, 875, 852, 835, 782, 703. ^{1}H NMR ($CDCl_3$, 600 MHz) δ: 7.31-7.28 (1H、m), 7.24 (1H, d, J = 4.8 Hz), 6.97 (1H, t, J = 4.5 Hz), 6.94 (1H, d, J = 3.4 Hz), 6.92 (1H, d, J = 8.2 Hz), 6.86 (2H, t, J = 2.7 Hz), 5.13 (1H, t, J = 7.9 Hz), 5.01-4.92 (2H, m), 3.81 (3H, s). ^{13}C NMR ($CDCl_3$, 150 MHz) δ: 160.1, 142.3, 140.3, 130.1, 127.0, 125.2, 125.1, 119.7, 113.8, 113.1, 79.9, 55.2, 44.7. HRMS (DART) calculated for $C_{13}H_{14}NO_3S^+$ [M+H$^+$] 264.06944, found 264.06984.

(*R*)-(1-Cyclohexyl-2-nitroethyl)benzene (3jb)[9, 8]

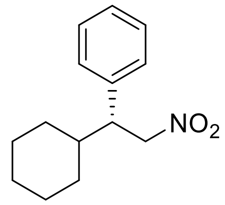

The enantio excess was determined by HPLC analysis on a chiralcel OD-H with hexane/2-propanol = 3/2, flow = 1.0 mL/min. ^{1}H NMR ($CDCl_3$, 600 MHz) δ: 7.23 (2H, t, J = 7.6 Hz), 7.18-7.15 (1H, m), 7.06 (d, 2H, J = 6.9 Hz), 4.70 (1H, dd, J = 12.4, 5.5 Hz), 4.54 (1H, dd, J = 12.4, 10.3 Hz), 3.21-3.17 (1H, m), 1.75-1.67 (2H, m), 1.60-1.49 (3H, m), 1.42-1.37 (1H, m), 1.22-1.13 (1H, m), 1.10-0.91 (3H, m), 0.83-0.75 (1H, m). ^{13}C NMR ($CDCl_3$, 150 MHz) δ: 138.8, 128.5, 128.1, 127.3, 78.8, 50.2, 40.9, 31.0, 30.6, 26.13, 26.09.

(*R*)-1-(1-Cyclohexyl-2-nitroethyl)-3-methoxybenzene (3ja)

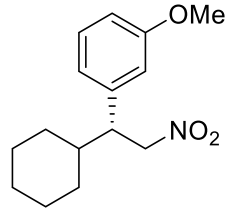

The enantio excess was determined by HPLC analysis on a chiralcel OD-H with hexane/2-propanol = 3/2, flow = 1.0 mL/min. $[\alpha]^{16}_D$ = +33.1 (c = 0.79, $CHCl_3$, as 91% ee). IR (KBr) cm^{-1}: 3435, 3036, 3006, 2977, 2930, 2848, 1600, 1551, 1487, 1466, 1446, 1384, 1350, 1329, 1284, 1255,

1216, 1200, 1165, 1131, 1086, 1038, 1008, 991, 963, 890, 865, 804, 784, 720, 700, 652, 617, 566, 541. M.p. = 84-86 ºC. ^{1}H NMR ($CDCl_3$, 600 MHz) δ: 7.20 (1H, t, *J* = 8.2 Hz), 6.77 (1H, dd, *J* = 8.2, 2.1 Hz), 6.71 (1H, d, *J* = 7.6 Hz), 6.66 (1H, s), 4.76-4.72 (1H, m), 4.61-4.56 (1H, m), 3.77 (3H, s), 3.24-3.20 (1H, m), 1.76 (2H, t, *J* = 16.2 Hz), 1.67-1.52 (3H, m), 1.47 (1H, d, *J* = 13.1 Hz), 1.27-1.19 (1H, m), 1.16-0.97 (3H, m), 0.90-0.82 (1H, m). ^{13}C NMR ($CDCl_3$, 150 MHz) δ: 159.7, 140.5, 129.5, 120.4, 114.5, 112.2, 78.8, 55.2, 50.2, 40.9, 31.0, 30.6, 26.15, 26.10. HRMS (DART) calculated for $C_{15}H_{22}NO_3^+$ [*M*+H$^+$] 264.15997, found 264.16061.

(*R*)-(3-Methyl-1-nitrobutan-2-yl)benzene (3kb)[7, 8, 9]

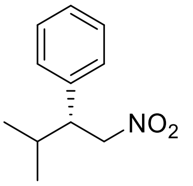

The enantio excess was determined by HPLC analysis on a chiralcel OD-H with hexane/2-propanol = 3/2, flow = 1.0 mL/min. ^{1}H NMR ($CDCl_3$, 600 MHz) δ: 7.31 (2H, t, *J* = 7.6 Hz), 7.27-7.25 (1H, m), 7.16-7.13 (2H, m), 4.76 (1H, q, *J* = 6.2 Hz), 4.64 (1H, dd, *J* = 12.4, 10.3 Hz), 3.26-3.21 (1H, m), 1.96 (1H, td, *J* = 14.1, 7.1 Hz), 1.01 (3H, d, *J* = 6.2 Hz), 0.81 (3H, d, *J* = 6.9 Hz). ^{13}C NMR ($CDCl_3$, 150 MHz) δ: 138.6, 128.6, 128.1, 127.4, 79.1, 51.0, 31.3, 20.6, 20.2.

(*R*)-1-Methoxy-3-(3-methyl-1-nitrobutan-2-yl)benzene (3ka)

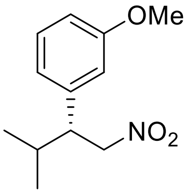

The enantio excess was determined by HPLC analysis on a chiralcel OD-H with hexane/2-propanol = 3/2, flow = 1.0 mL/min. $[\alpha]^{16}_{D}$ = +29.9 (c = 0.85, $CHCl_3$, as 91% ee). IR (neat) cm^{-1}: 3583, 2964, 2875, 2838, 1602, 1586, 1553, 1490, 1467, 1436, 1381, 1322, 1290, 1263, 1205, 1163, 1129, 1044, 990, 876, 783, 736, 704, 664. ^{1}H NMR ($CDCl_3$, 600 MHz) δ: 7.21 (1H, t, *J* = 8.2 Hz), 6.77 (1H, dd, *J* = 8.2, 2.7 Hz), 6.72 (1H, d, *J* = 7.6 Hz), 6.67 (1H, s), 4.72 (1H, q, *J* = 6.2 Hz), 4.60 (1H, dd, *J* = 12.4, 10.3 Hz), 3.77 (3H, s), 3.20-3.15 (1H, m), 1.95-1.89 (1H, m), 0.99 (3H, d, *J* = 6.9 Hz), 0.80 (3H, d, *J* = 6.9 Hz). ^{13}C NMR ($CDCl_3$, 150 MHz) δ: 159.6, 140.3, 129.5, 120.3, 114.4, 112.3, 79.0, 55.2, 51.0, 31.3, 20.6, 20.3. HRMS (DART) calculated for $C_{12}H_{18}NO_3^+$ [*M*+H$^+$] 224.12867, found 224.12934.

(*R*)-(1-Nitrohexan-2-yl)benzene (3lb)[7]

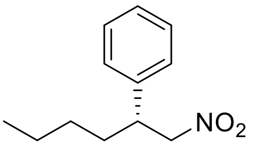

The enantio excess was determined by HPLC analysis on a chiralcel OD-H with hexane/2-propanol = 99/1, flow = 1.0 mL/min. ^{1}H NMR ($CDCl_3$, 600 MHz) δ: 7.33 (2H, t, *J* = 7.6 Hz), 7.27-7.25 (1H, m), 7.20-7.17 (2H, m), 4.59-4.51 (2H, m), 3.47-3.41 (1H, m), 1.68 (2H, dd, *J* = 15.1, 7.6 Hz), 1.34-1.13 (5H, m), 0.84 (3H, t, *J* = 7.2 Hz). ^{13}C NMR ($CDCl_3$, 150 MHz) δ: 139.6, 128.9, 127.53, 127.50, 81.0, 44.4, 32.7, 29.0, 22.4, 13.8.

(*R*)-1-Methoxy-3-(1-nitrohexan-2-yl)benzene (3la)

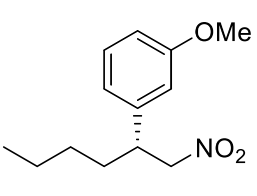

The enantio excess was determined by HPLC analysis on a chiralcel

OD-H with hexane/2-propanol = 99/1, flow = 1.0 mL/min. $[\alpha]^{20}_{D}$ = +16.2 (c = 0.79, $CHCl_3$, as 86% ee). IR (neat) cm^{-1}: 3003, 2957, 2932, 2861, 1602, 1587, 1552, 1490, 1457, 1436, 1379, 1319, 1261, 1193, 1160, 1122, 1047, 874, 783, 730, 701. ^{1}H NMR ($CDCl_3$, 600 MHz) δ: 7.25 (1H, t, J = 7.6 Hz), 6.81-6.76 (2H, m), 6.72 (1H, t, J = 2.1 Hz), 4.56-4.49 (2H, m), 3.80 (3H, s), 3.43-3.38 (1H, m), 1.67 (2H, dd, J = 15.8, 7.6 Hz), 1.34-1.15 (4H, m), 0.84 (3H, t, J = 7.2 Hz). ^{13}C NMR ($CDCl_3$, 150 MHz) δ: 159.9, 141.2, 129.9, 119.7, 113.7, 112.4, 80.9, 55.2, 44.3, 32.6, 29.0, 22.4, 13.8. HRMS (DART) calculated for $C_{13}H_{20}NO_3^+$ [M+H^+] 238.14432, found 238.14468.

10. Charts

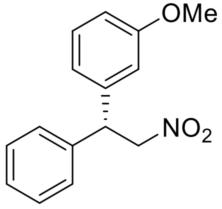

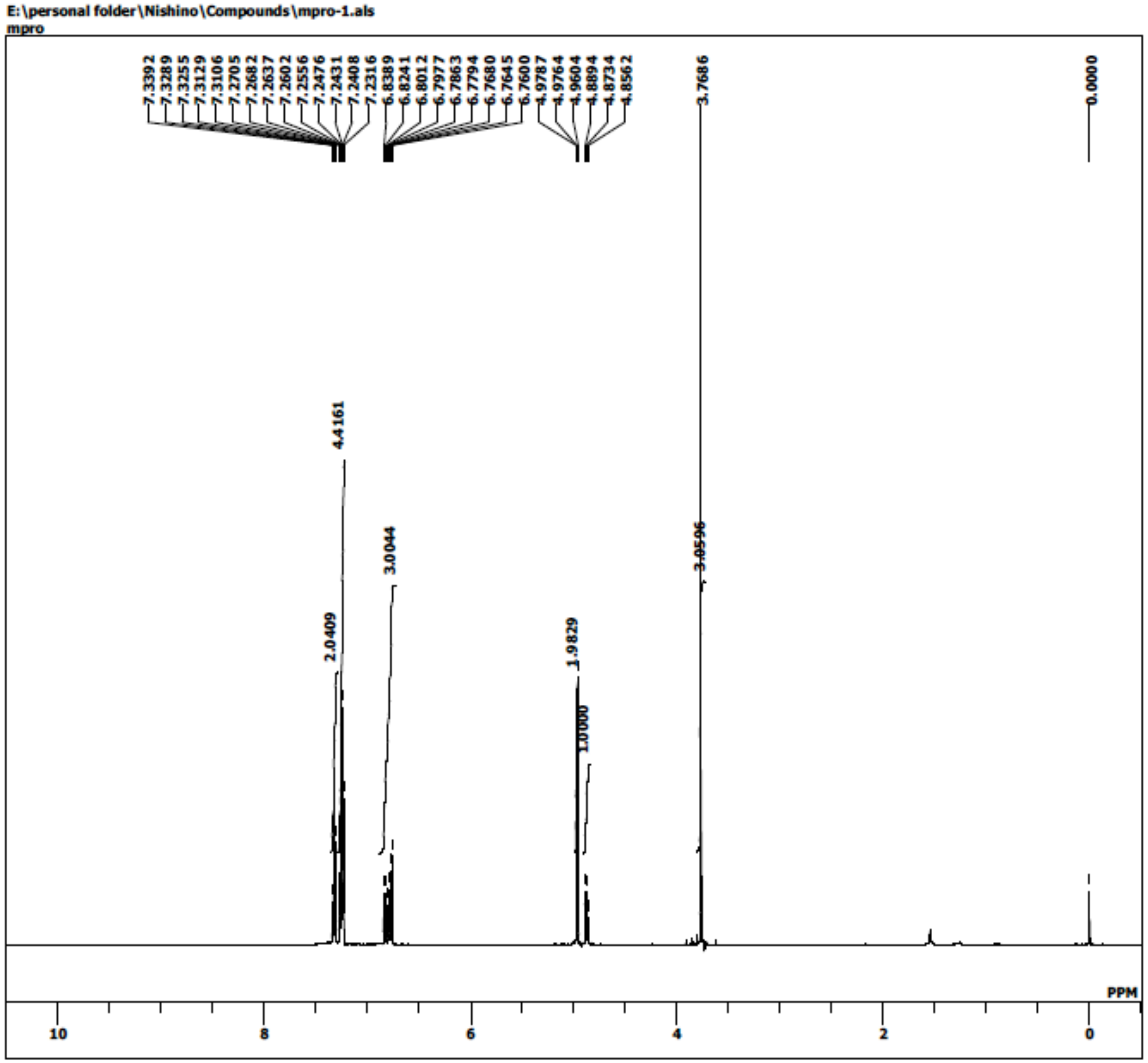

3aa 1H NMR

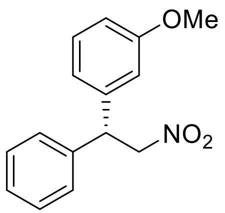
OMe
NO2

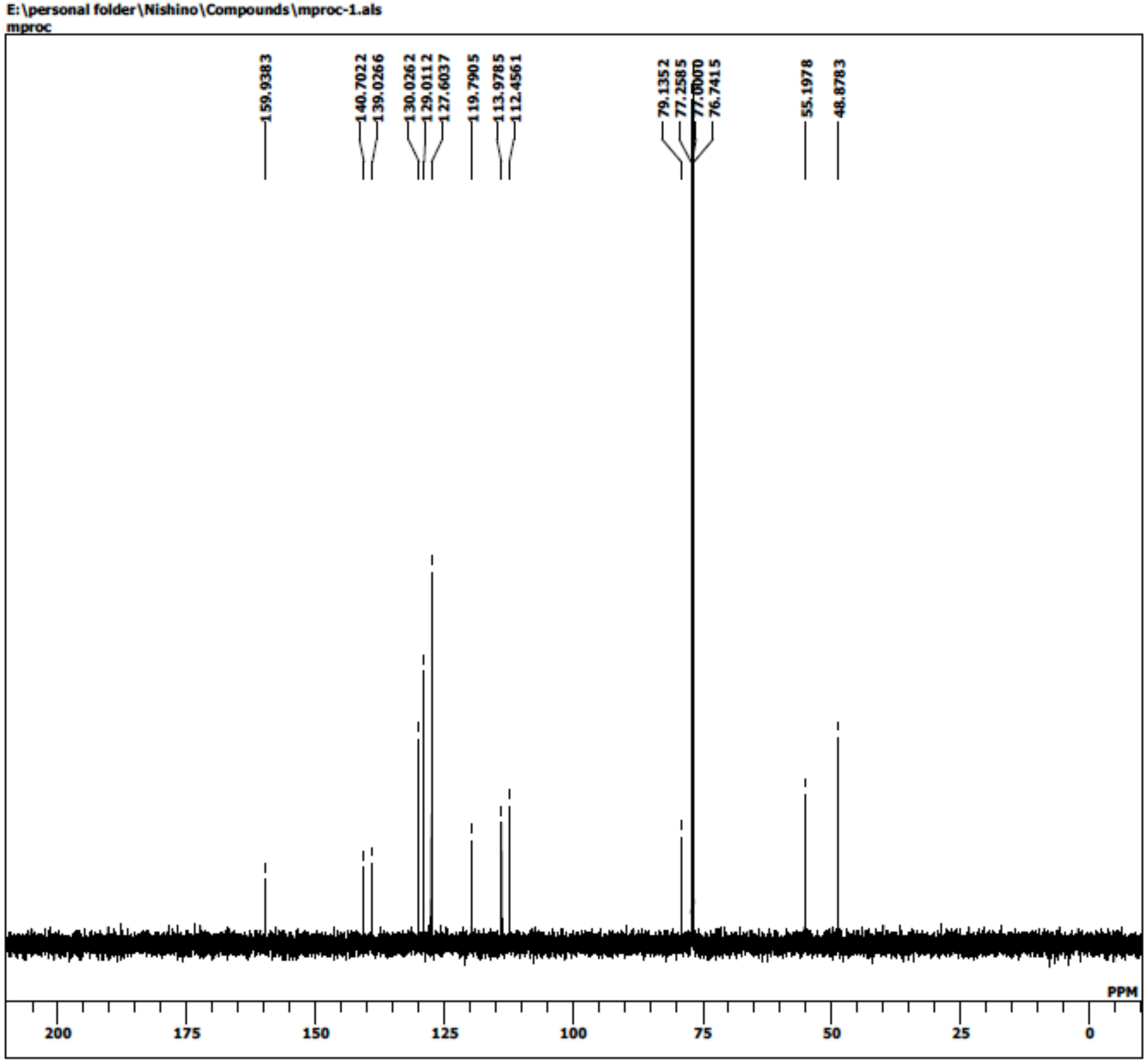
E:\personal folder\Nishino\Compounds\mproc-1.als
mproc
159.9383
140.7022
139.0266
130.0262
129.0112
127.6037
119.7905
113.9785
112.4561
79.1352
77.2585
77.0000
76.7415
55.1978
48.8783
PPM
200
175
150
125
100
75
50
25
0

3aa 13C NMR

Sample Name : nsn363rac
OD-H, 1.0 mL/min, Hex:IsoPro= 1 : 1

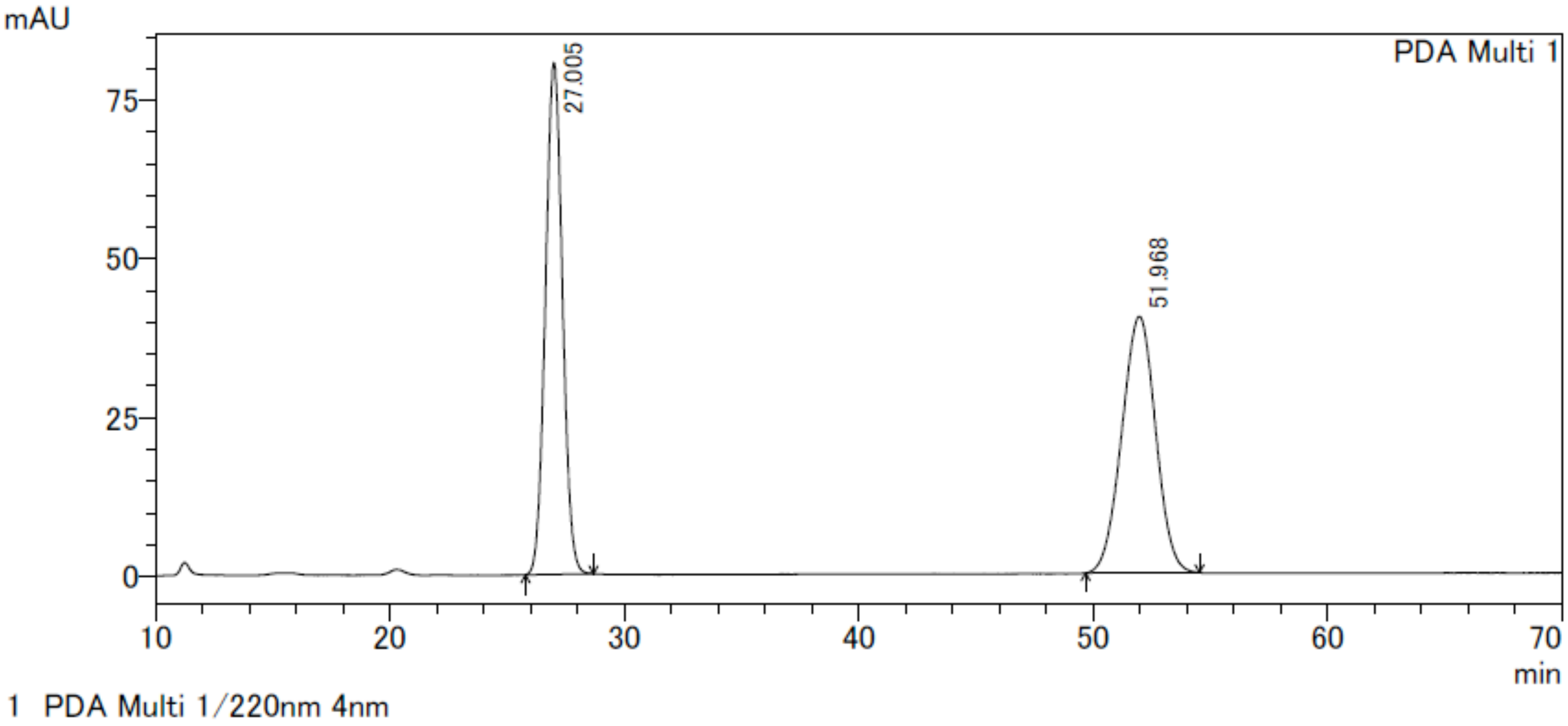

PDA Ch1 220nm 4nm

Peak#	Ret. Time	Area	Height	Area%
1	27.005	4075750	80632	50.094
2	51.968	4060403	40411	49.906

3aa racemic

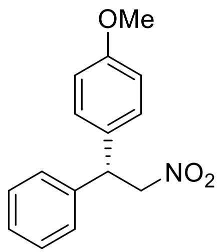

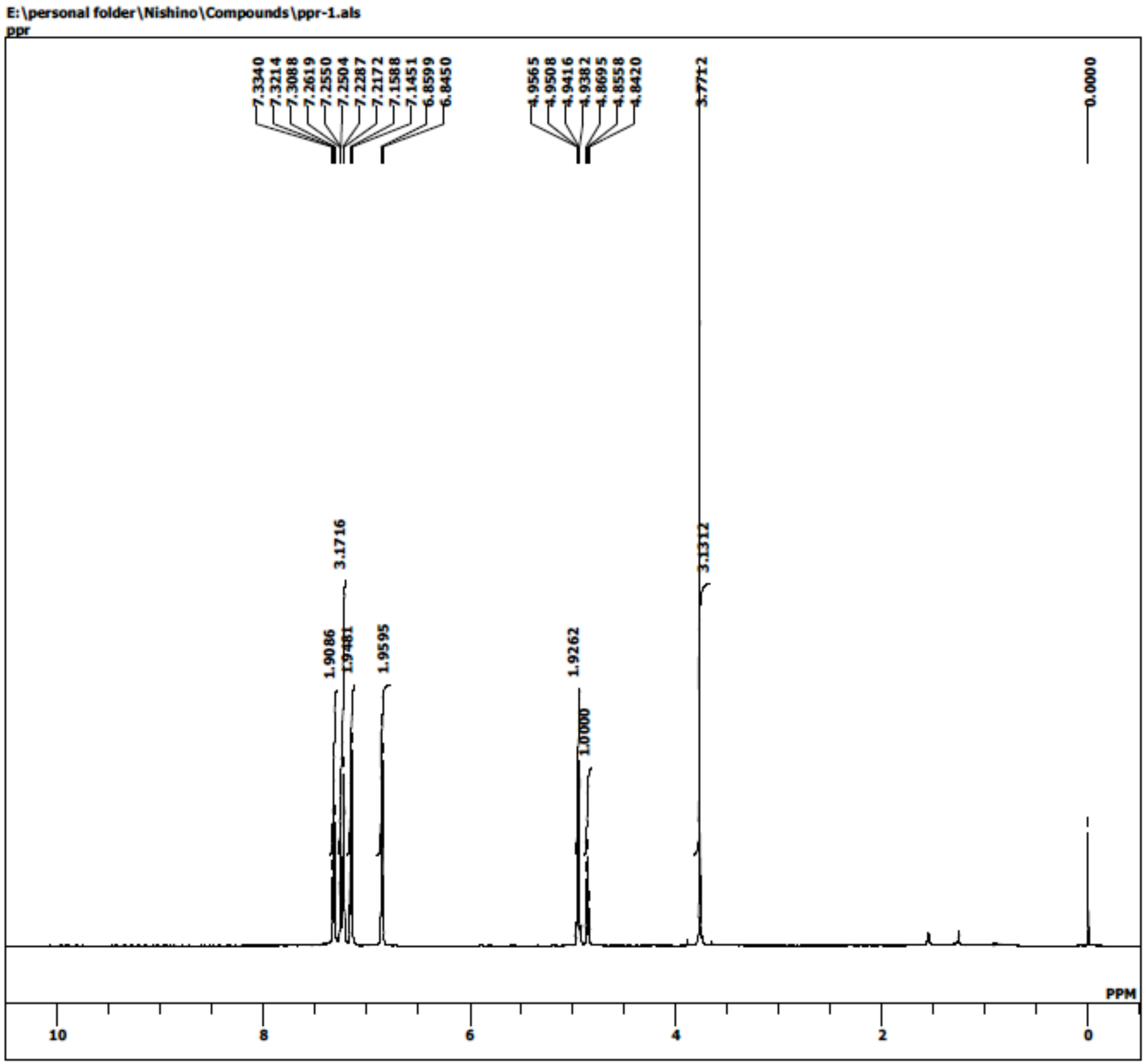
E:\personal folder\Nishino\Compounds\ppr-1.als
ppr
7.3340
7.3214
7.3088
7.2619
7.2550
7.2504
7.2287
7.2172
7.1588
7.1451
6.8599
6.8450
4.9565
4.9508
4.9416
4.9382
4.8695
4.8558
4.8420
3.7712
0.0000
3.1716
1.9086
1.9481
1.9595
1.9262
1.0000
3.1312
PPM
10
8
6
4
2
0

3ac 1H NMR

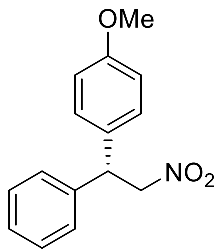

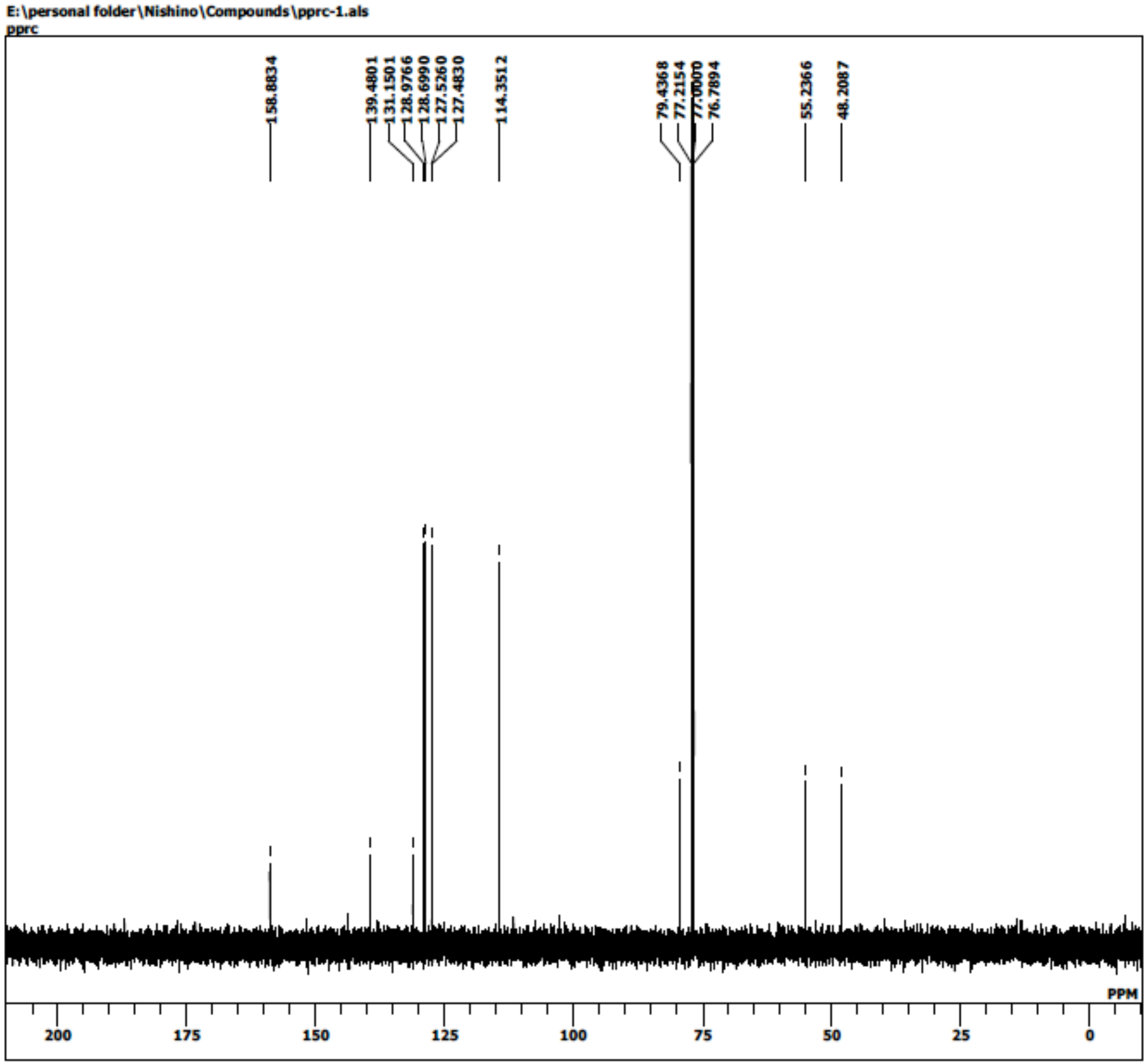
E:\personal folder\Nishino\Compounds\pprc-1.als
pprc
158.8834
139.4801
131.1501
128.9766
128.6990
127.5260
127.4830
114.3512
79.4368
77.2154
77.0000
76.7894
55.2366
48.2087
PPM
200
175
150
125
100
75
50
25
0

3ac 13C NMR

Sample Name : nsn121rac(for560)
OD-H, 1.0 mL/min, Hex:IPA = 3/2

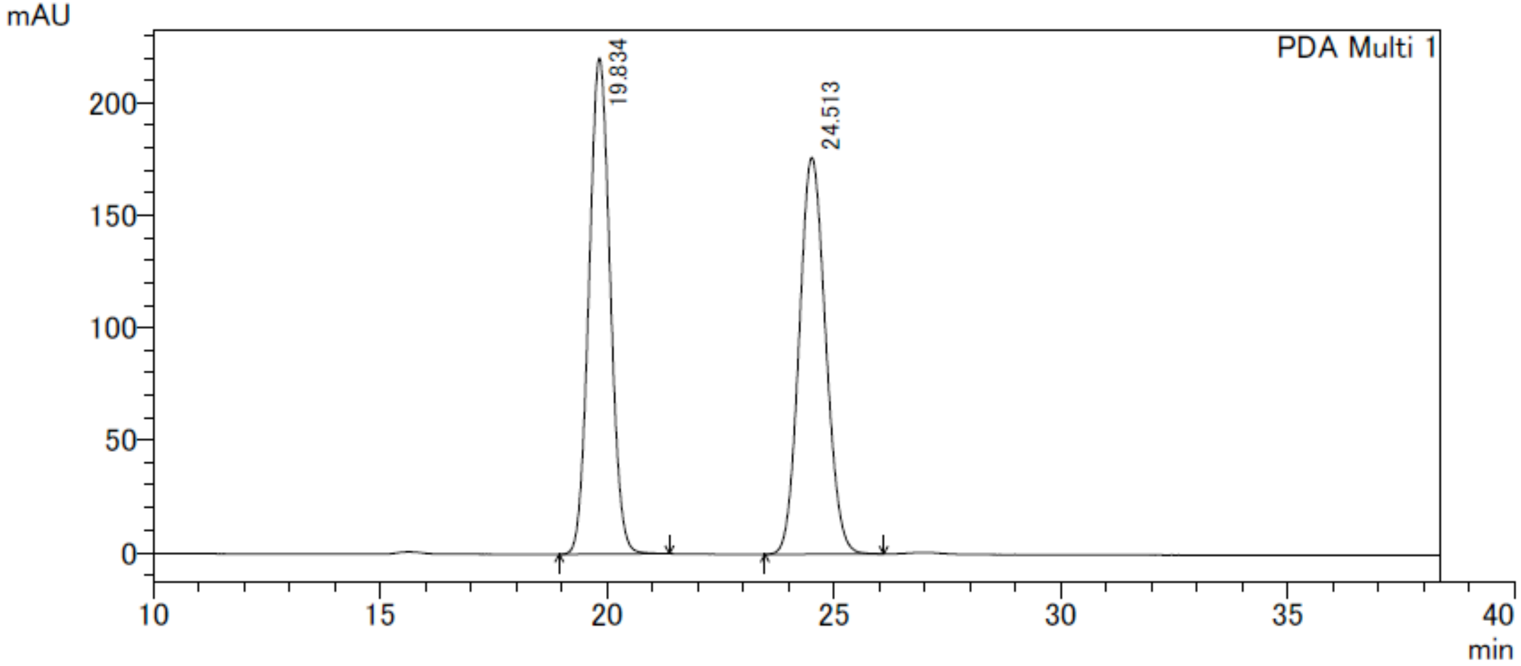

1 PDA Multi 1/220nm 4nm

PDA Ch1 220nm 4nm

Peak#	Ret. Time	Area	Height	Area%
1	19.834	7144237	220552	50.018
2	24.513	7139029	176357	49.982

3ac racemic

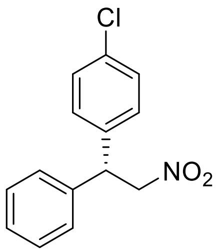

Cl
NO_2

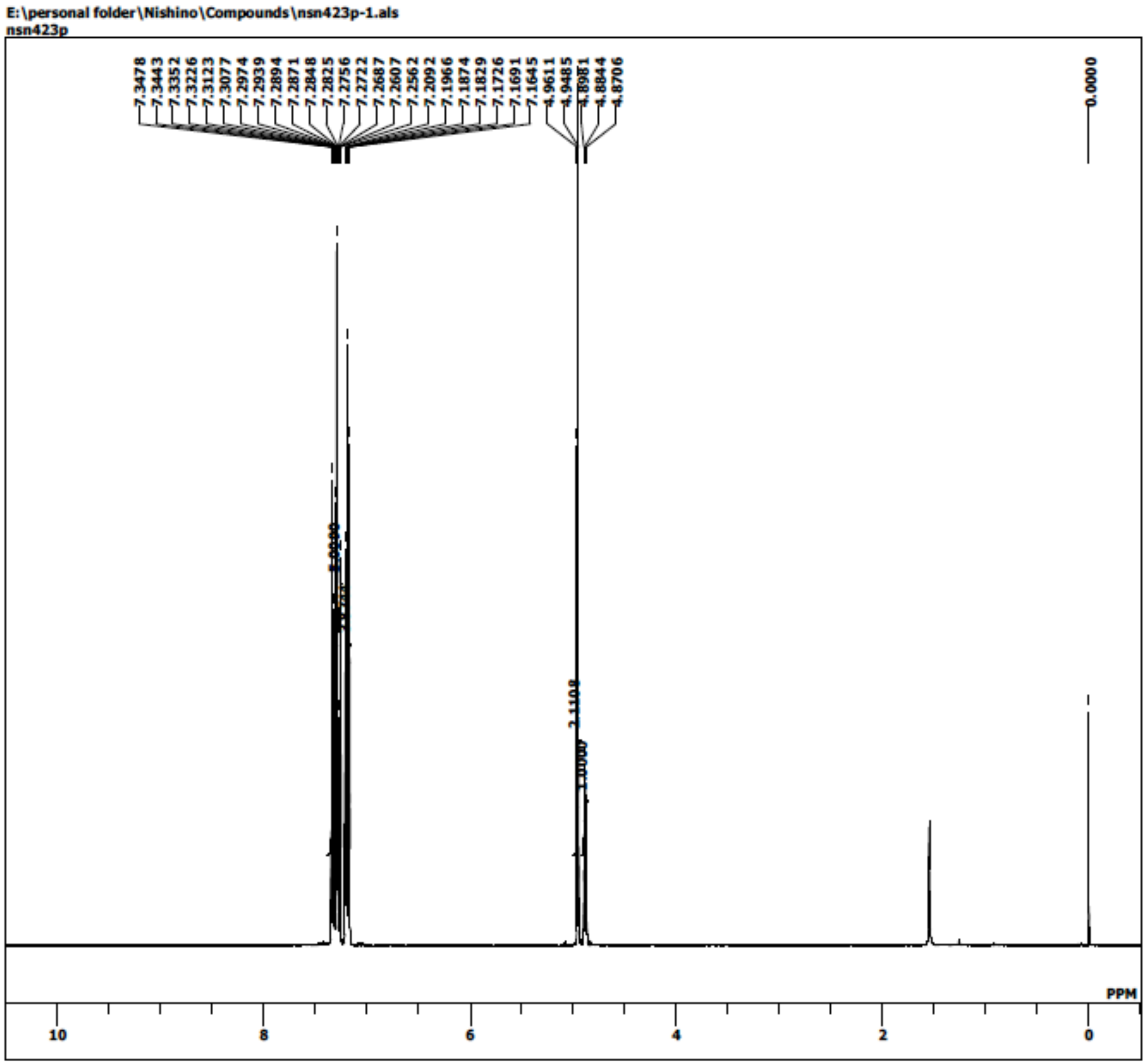

E:\personal folder\Nishino\Compounds\nsn423p-1.als
nsn423p
7.3478
7.3443
7.3352
7.3226
7.3123
7.3077
7.2974
7.2939
7.2894
7.2871
7.2848
7.2825
7.2756
7.2722
7.2687
7.2607
7.2562
7.2092
7.1966
7.1874
7.1829
7.1726
7.1691
7.1645
4.9611
4.9485
4.8981
4.8844
4.8706
0.0000
PPM
10
8
6
4
2
0

3ad 1H NMR

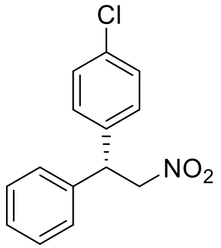

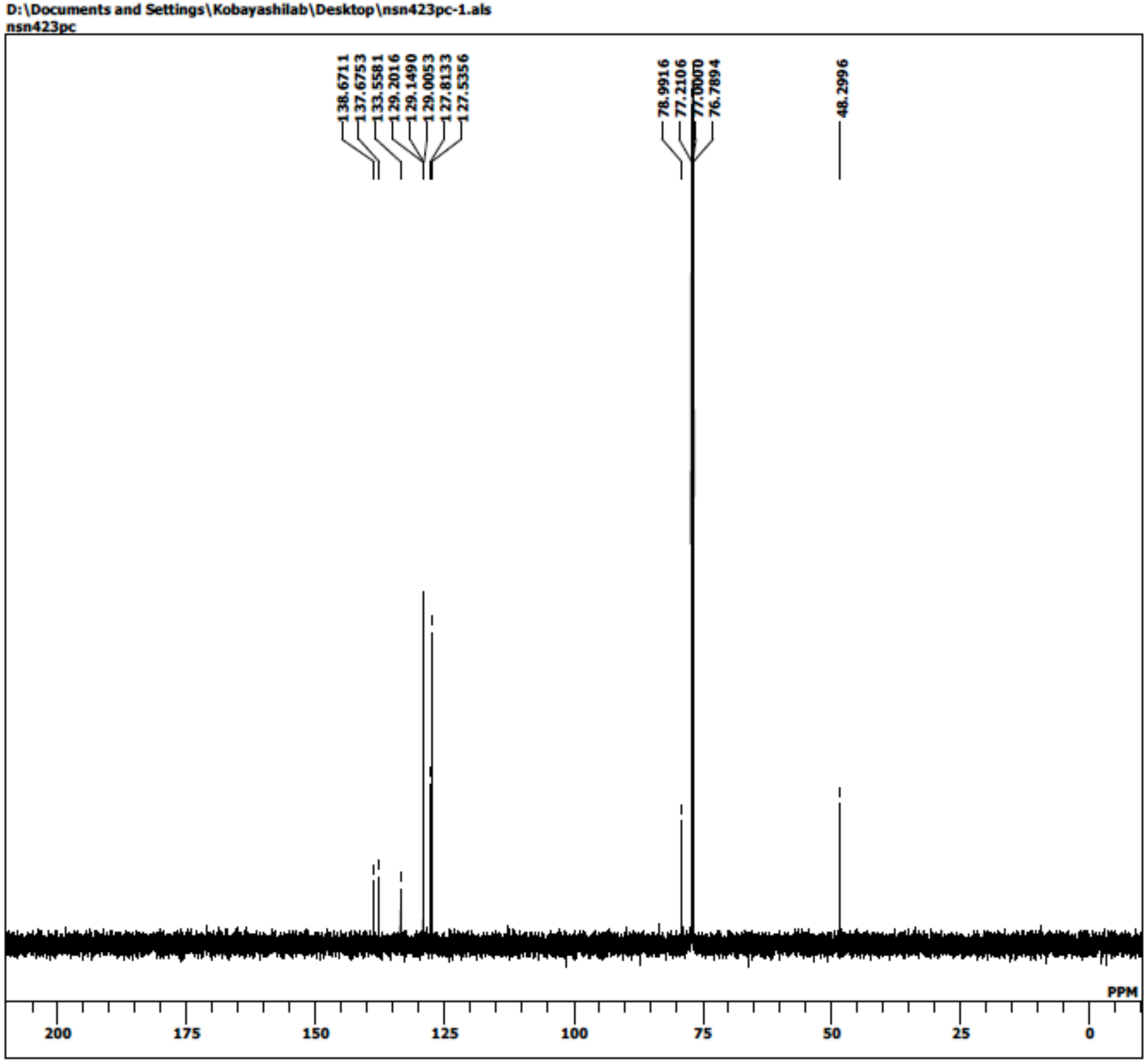
D:\Documents and Settings\Kobayashilab\Desktop\nsn423pc-1.als
nsn423pc
138.6711
137.6753
133.5581
129.2016
129.1490
129.0053
127.8133
127.5356
78.9916
77.2106
77.0000
76.7894
48.2996
PPM
200
175
150
125
100
75
50
25
0

3ad 13H NMR

Sample Name : nsn425rac(for580)
OD-H, 1.0 mL/min, Hex:IPA = 3/2

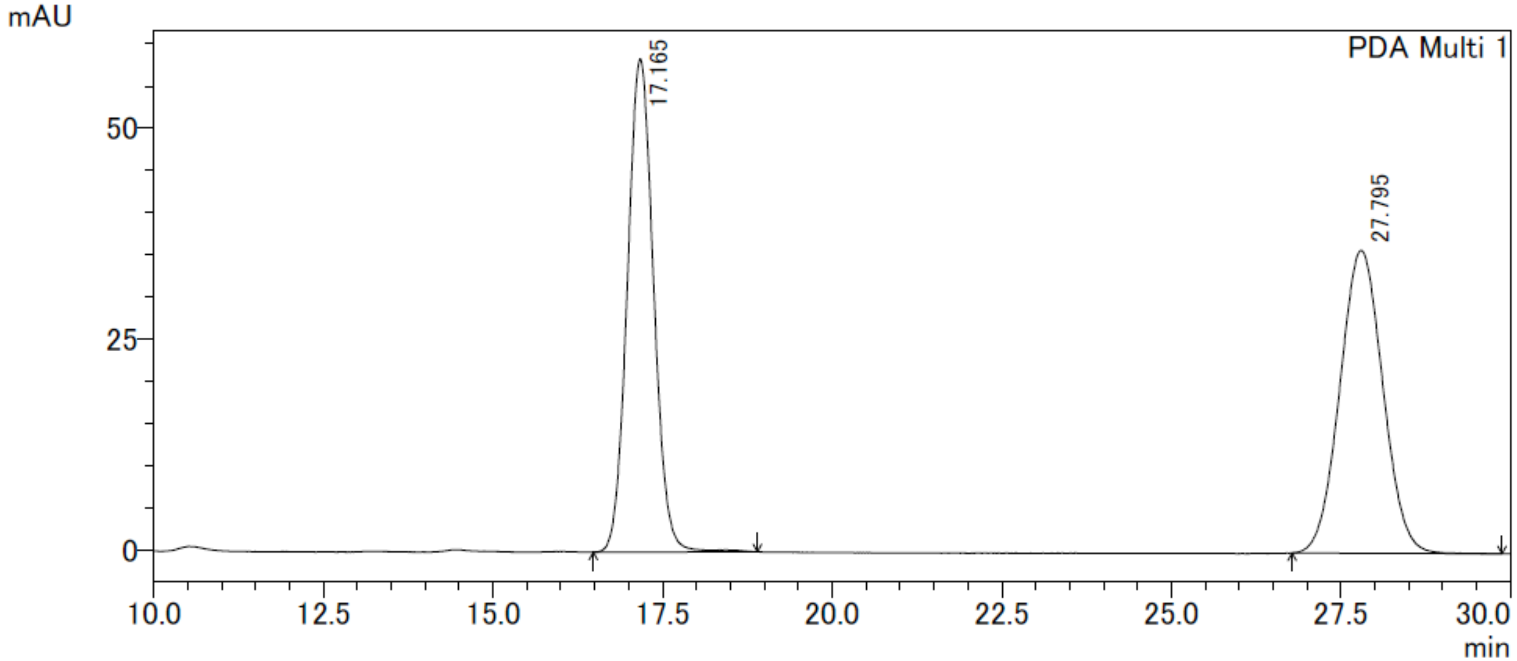

1 PDA Multi 1/220nm 4nm

PDA Ch1 220nm 4nm

Peak#	Ret. Time	Area	Height	Area%
1	17.165	1572780	58446	49.873
2	27.795	1580809	35885	50.127

3ad racemic

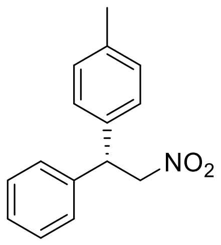

NO2

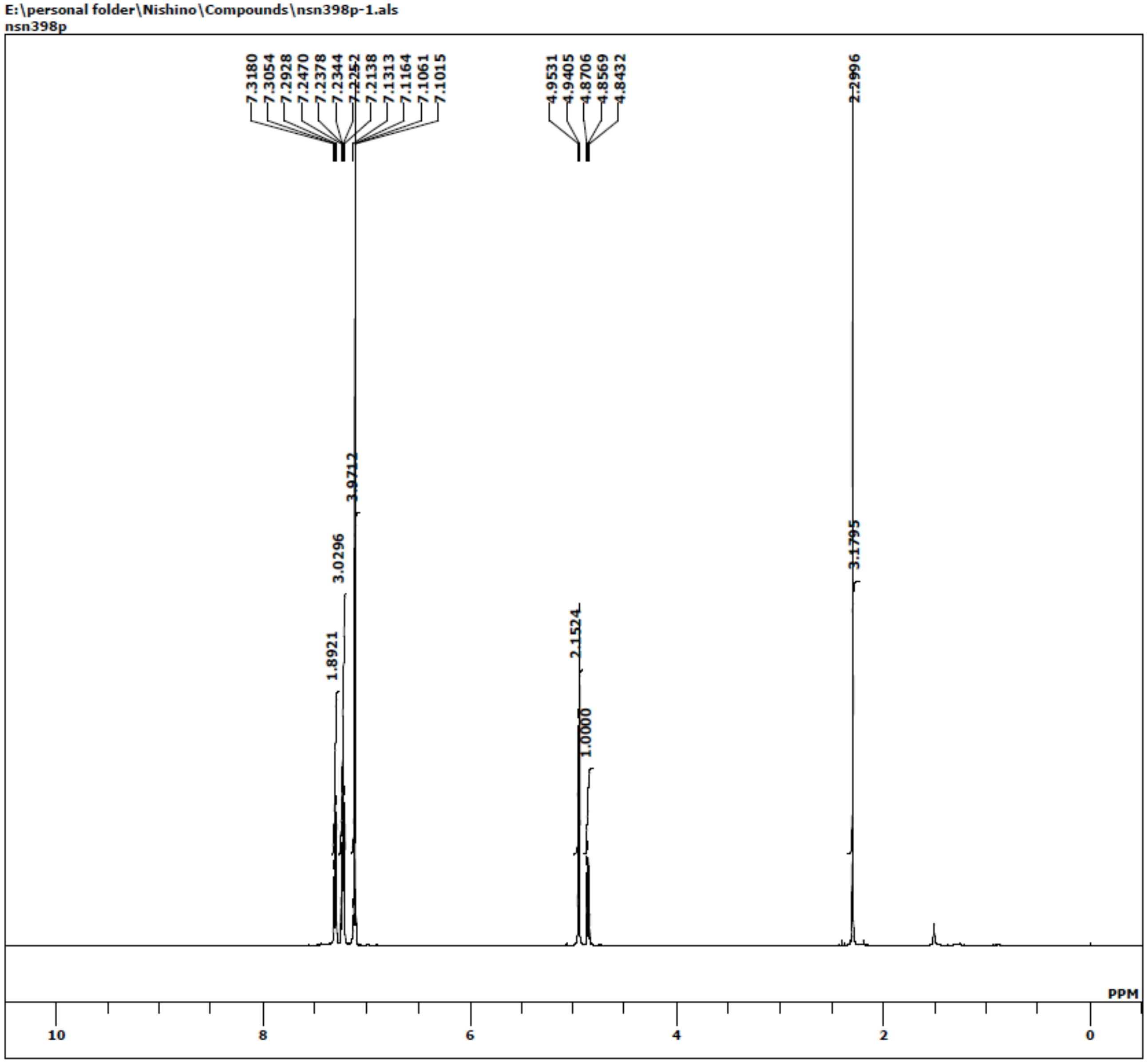

E:\personal folder\Nishino\Compounds\nsn398p-1.als
nsn398p
7.3180
7.3054
7.2928
7.2470
7.2378
7.2344
7.2252
7.2138
7.1313
7.1164
7.1061
7.1015
4.9531
4.9405
4.8706
4.8569
4.8432
2.2996
3.9712
3.0296
1.8921
2.1524
1.0000
3.1795
PPM
10
8
6
4
2
0

3af 1H NMR

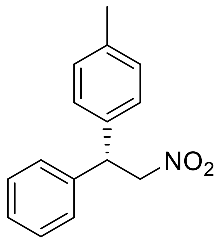
NO2

3af 13C NMR

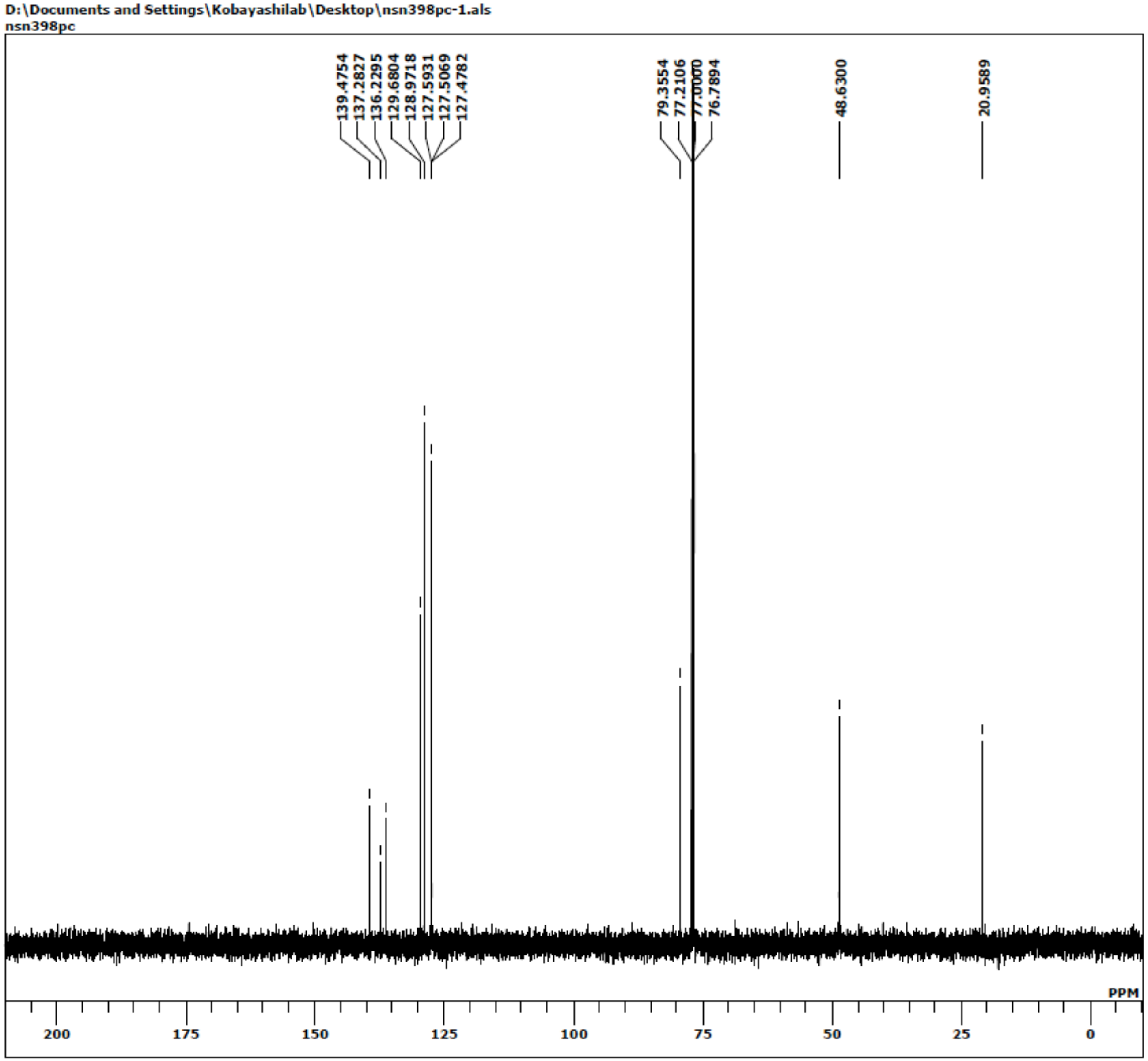
D:\Documents and Settings\Kobayashilab\Desktop\nsn398pc-1.als
nsn398pc
139.4754
137.2827
136.2295
129.6804
128.9718
127.5931
127.5069
127.4782
79.3554
77.2106
77.0000
76.7894
48.6300
20.9589
PPM
200
175
150
125
100
75
50
25
0

Sample Name : nsn389rac(for565and573)
OD-H, 1.0 mL/min, Hex:IPA = 9/1

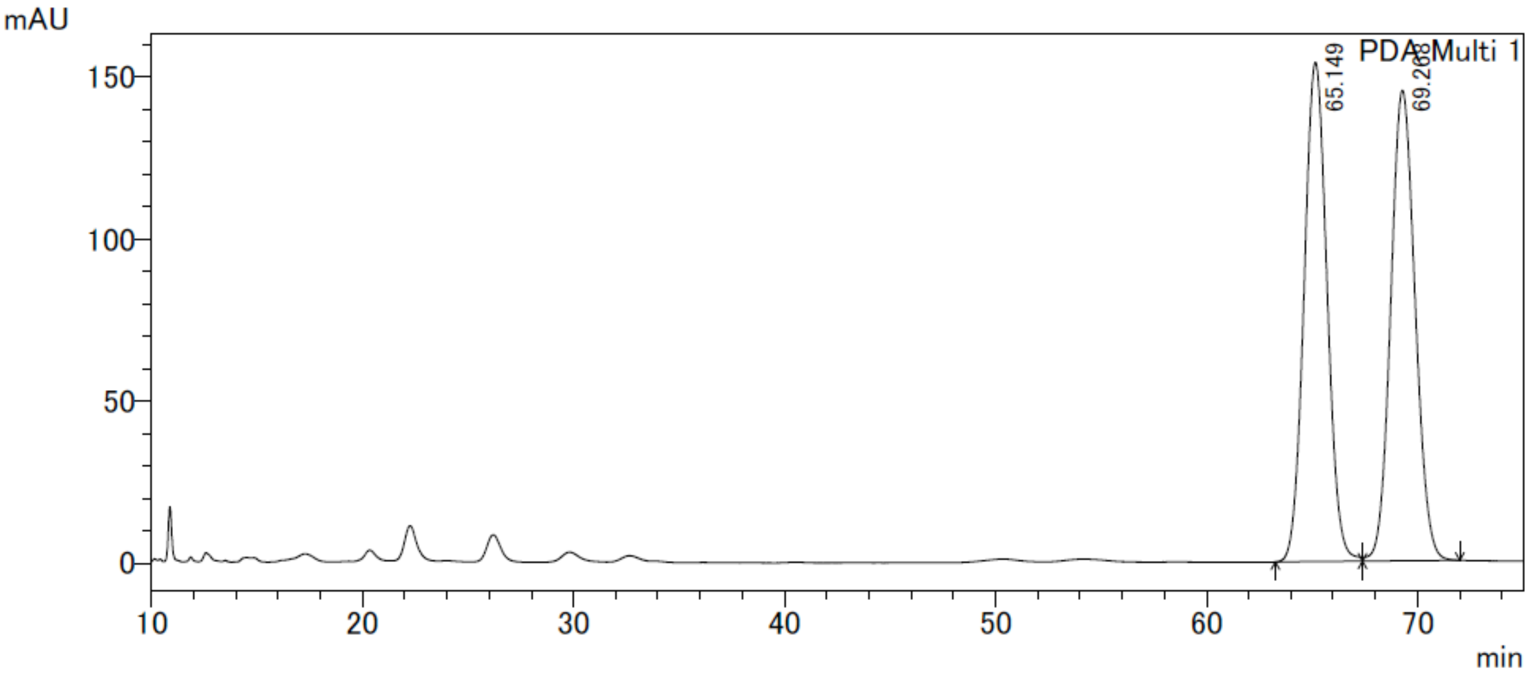

1 PDA Multi 1/220nm 4nm

PDA Ch1 220nm 4nm

Peak#	Ret. Time	Area	Height	Area%
1	65.149	11668543	154080	49.947
2	69.268	11693200	145159	50.053

3af racemic

3af racemic(2)

==== Shimadzu LCsolution 分析レポート ====

D:¥Nishino¥nsn389rac.lcd

分析者 : System Administrator
サンプル名 : nsn389rac
サンプルID : nsn389rac
測定条件 :OD-H, 1.0 mL/min, Hex:IPA = 3 : 2
注入量 : 1 uL
データファイル : nsn389rac.lcd
メソッドファイル : product.lcm

レポートファイル : Default.lcr
分析日時 : 2014/07/28 13:07:28
解析日時 : 2018/07/08 14:17:46

<クロマトグラム>

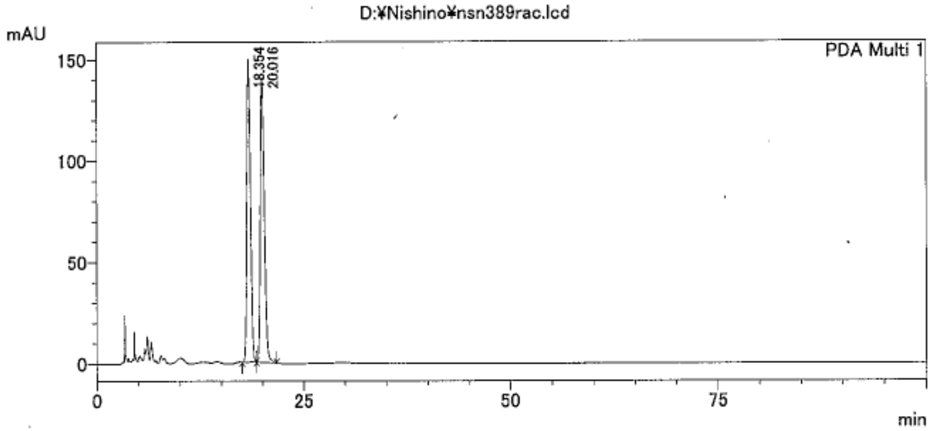

1 PDA Multi 1/220nm 4nm

ピークテーブル D:¥Nishino¥nsn389rac.lcd

PDA Ch1 220nm 4nm

ピーク#	保持時間	面積	高さ	面積%	高さ%
1	18.354	4506665	149506	48.675	51.404
2	20.016	4751977	141338	51.325	48.596
Total		9258641	290844	100.000	100.000

ピーク：**peak** 保持時間:**retention time** 面積:**area** 高さ:**height**

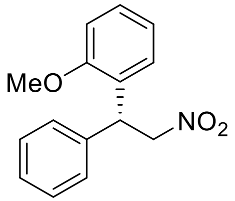

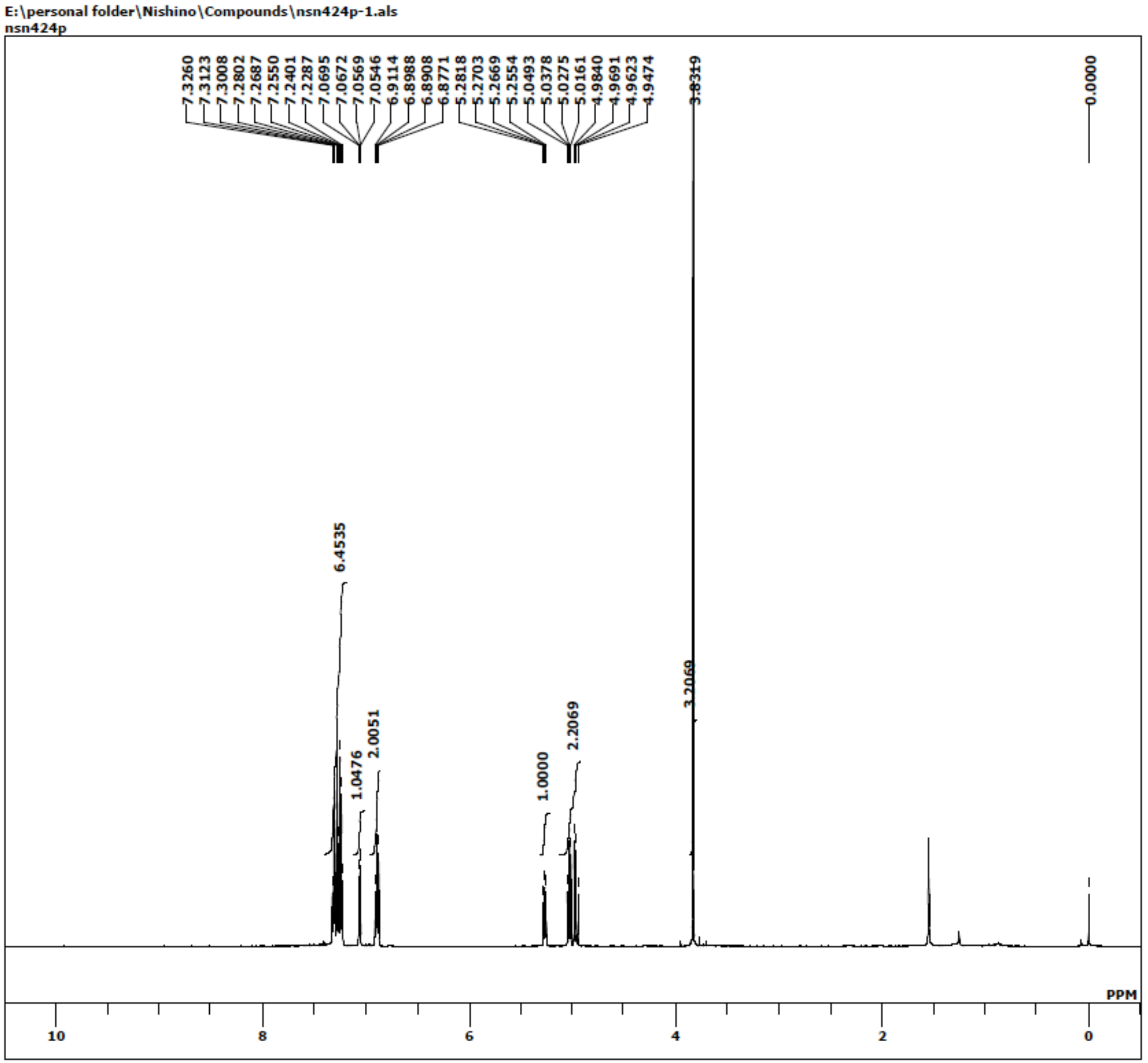
E:\personal folder\Nishino\Compounds\nsn424p-1.als
nsn424p
7.3260
7.3123
7.3008
7.2802
7.2687
7.2550
7.2401
7.2287
7.0695
7.0672
7.0569
7.0546
6.9114
6.8988
6.8908
6.8771
5.2818
5.2703
5.2669
5.2554
5.0493
5.0378
5.0275
5.0161
4.9840
4.9691
4.9623
4.9474
3.8319
0.0000
6.4535
1.0476
2.0051
1.0000
2.2069
3.2069
PPM
10
8
6
4
2
0

3ag 1H NMR

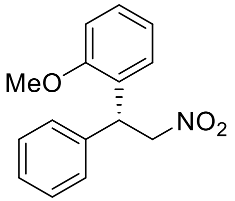

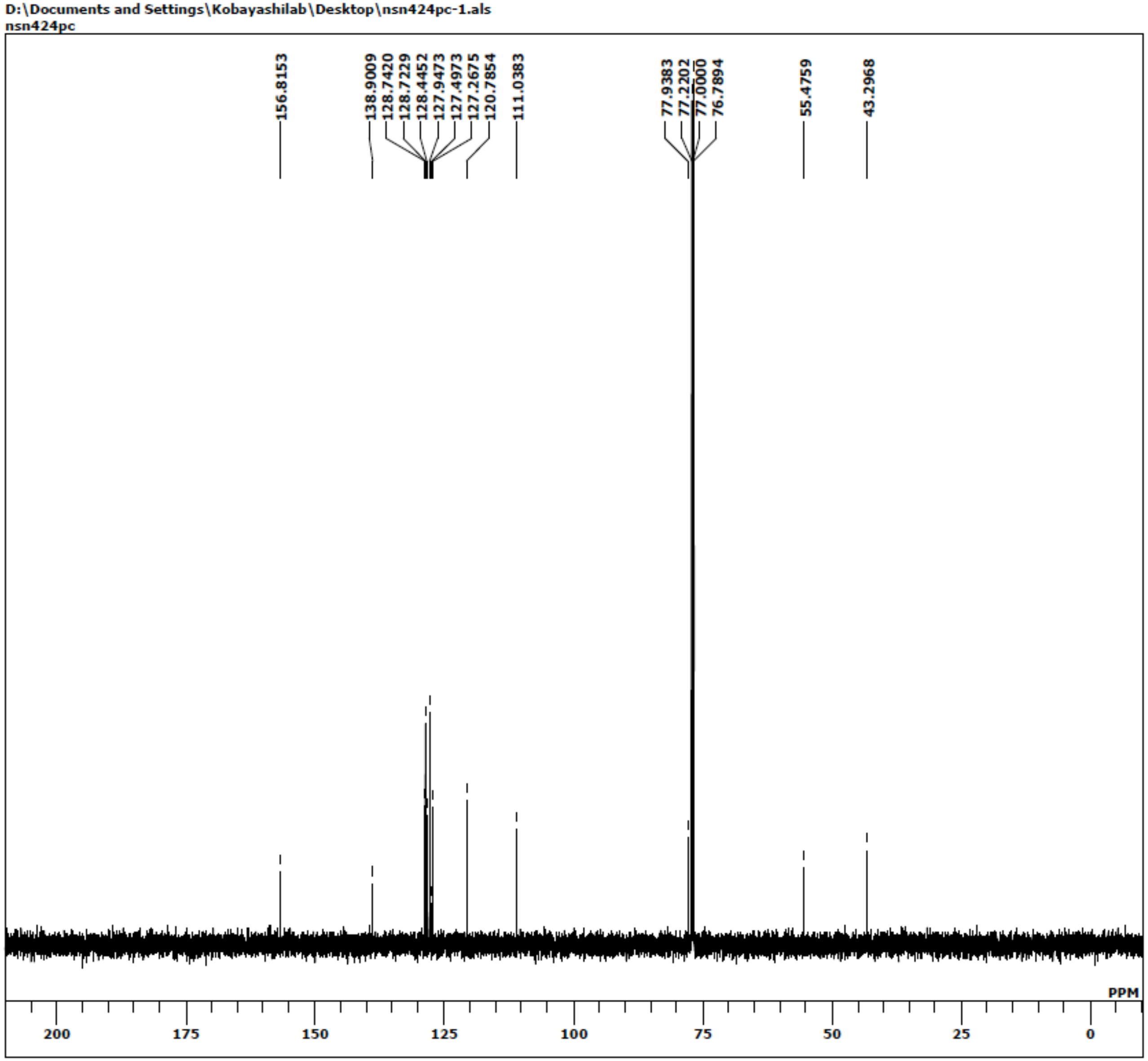

D:\Documents and Settings\Kobayashilab\Desktop\nsn424pc-1.als
nsn424pc
156.8153
138.9009
128.7420
128.7229
128.4452
127.9473
127.4973
127.2675
120.7854
111.0383
77.9383
77.2202
77.0000
76.7894
55.4759
43.2968
PPM
200
175
150
125
100
75
50
25
0

3ag 13C NMR

Sample Name : nsn448rac(for581)
OD-H, 1.0 mL/min, Hex:IPA = 3/2

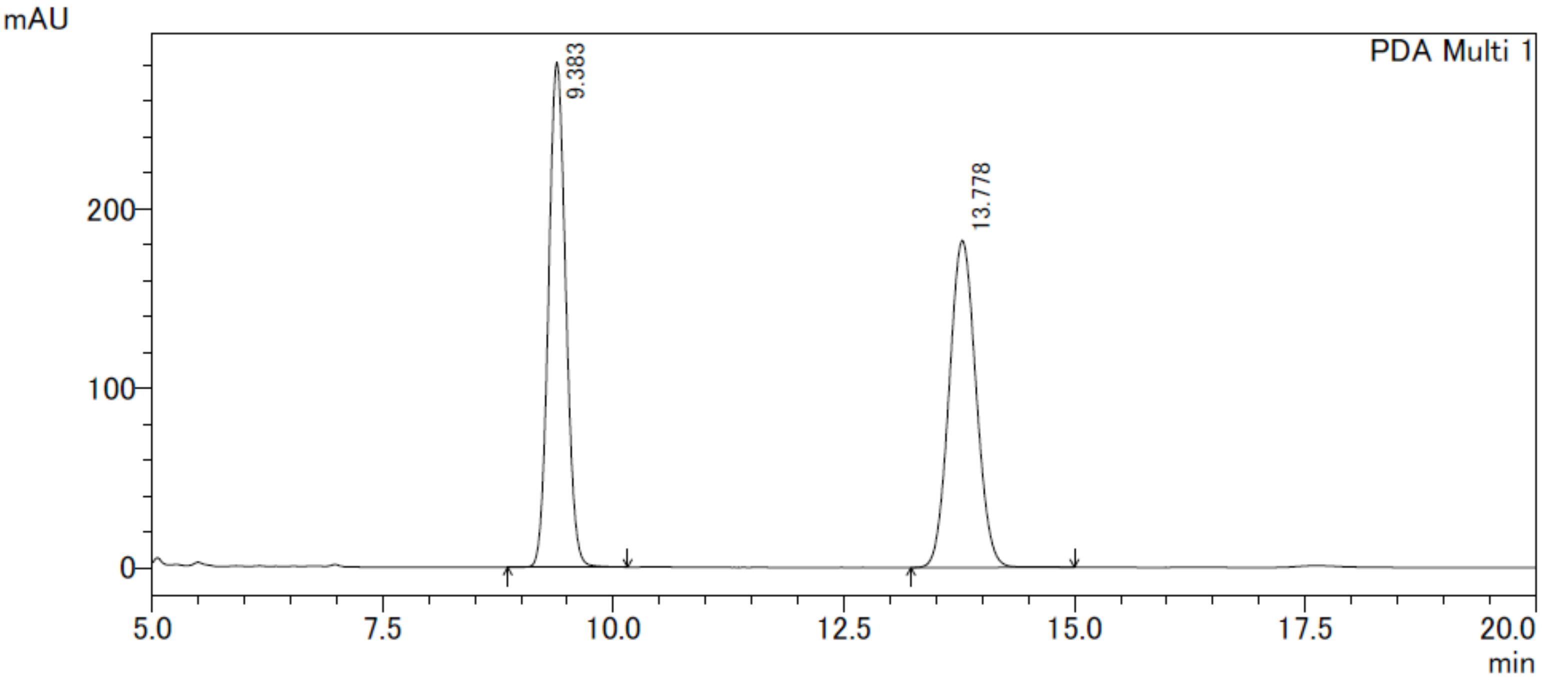

1 PDA Multi 1/220nm 4nm

PDA Ch1 220nm 4nm

Peak#	Ret. Time	Area	Height	Area%
1	9.383	3670882	281722	49.859
2	13.778	3691604	182372	50.141

3ag racemic

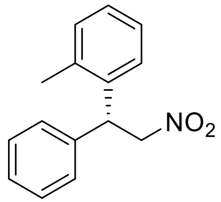

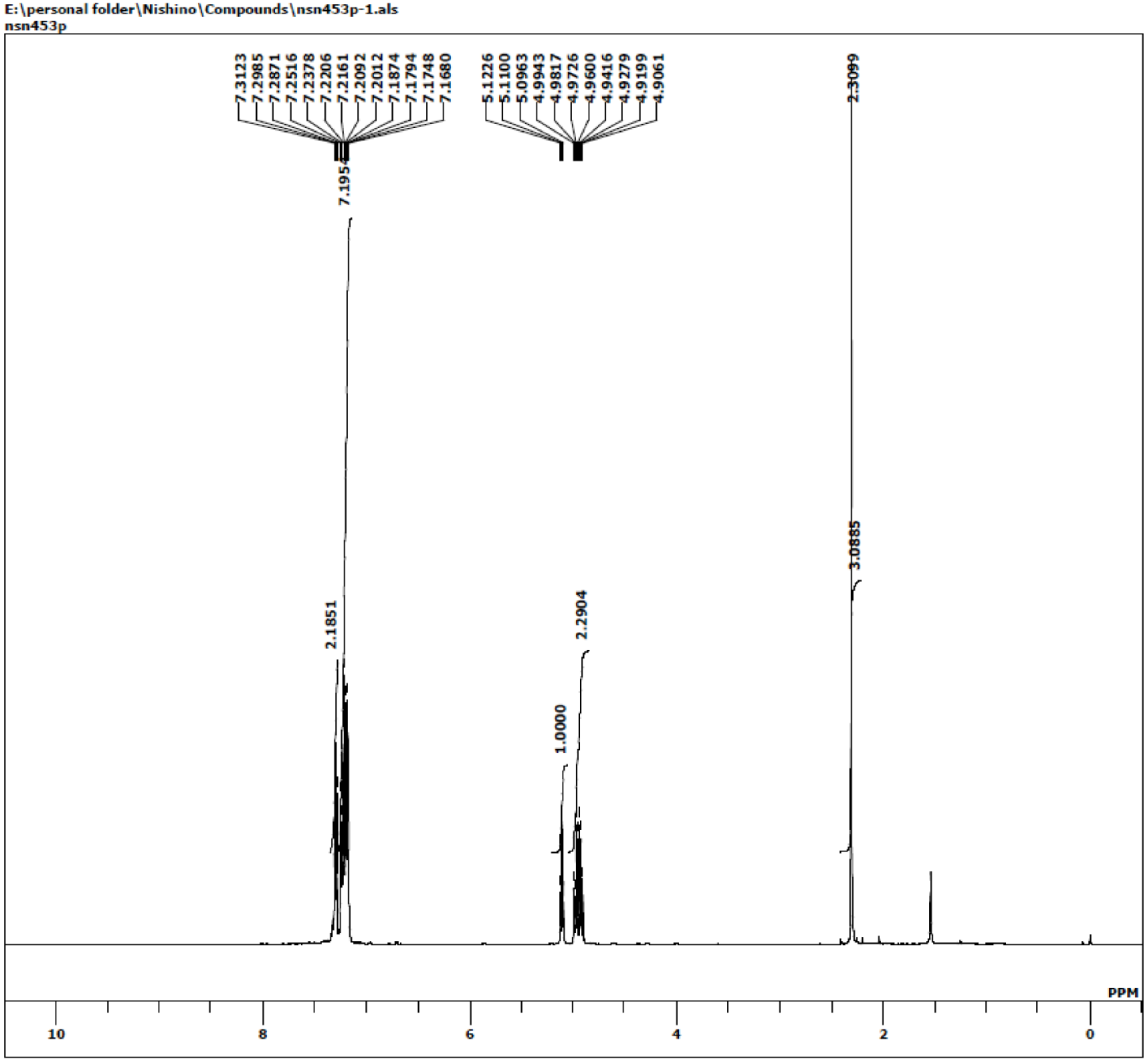
E:\personal folder\Nishino\Compounds\nsn453p-1.als
nsn453p
7.3123
7.2985
7.2871
7.2516
7.2378
7.2206
7.2161
7.2092
7.2012
7.1874
7.1794
7.1748
7.1680
7.1954
5.1226
5.1100
5.0963
4.9943
4.9817
4.9726
4.9600
4.9416
4.9279
4.9199
4.9061
2.3099
3.0885
2.1851
2.2904
1.0000
PPM
10
8
6
4
2
0

3ah 1H NMR

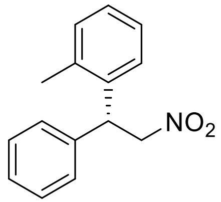
NO2

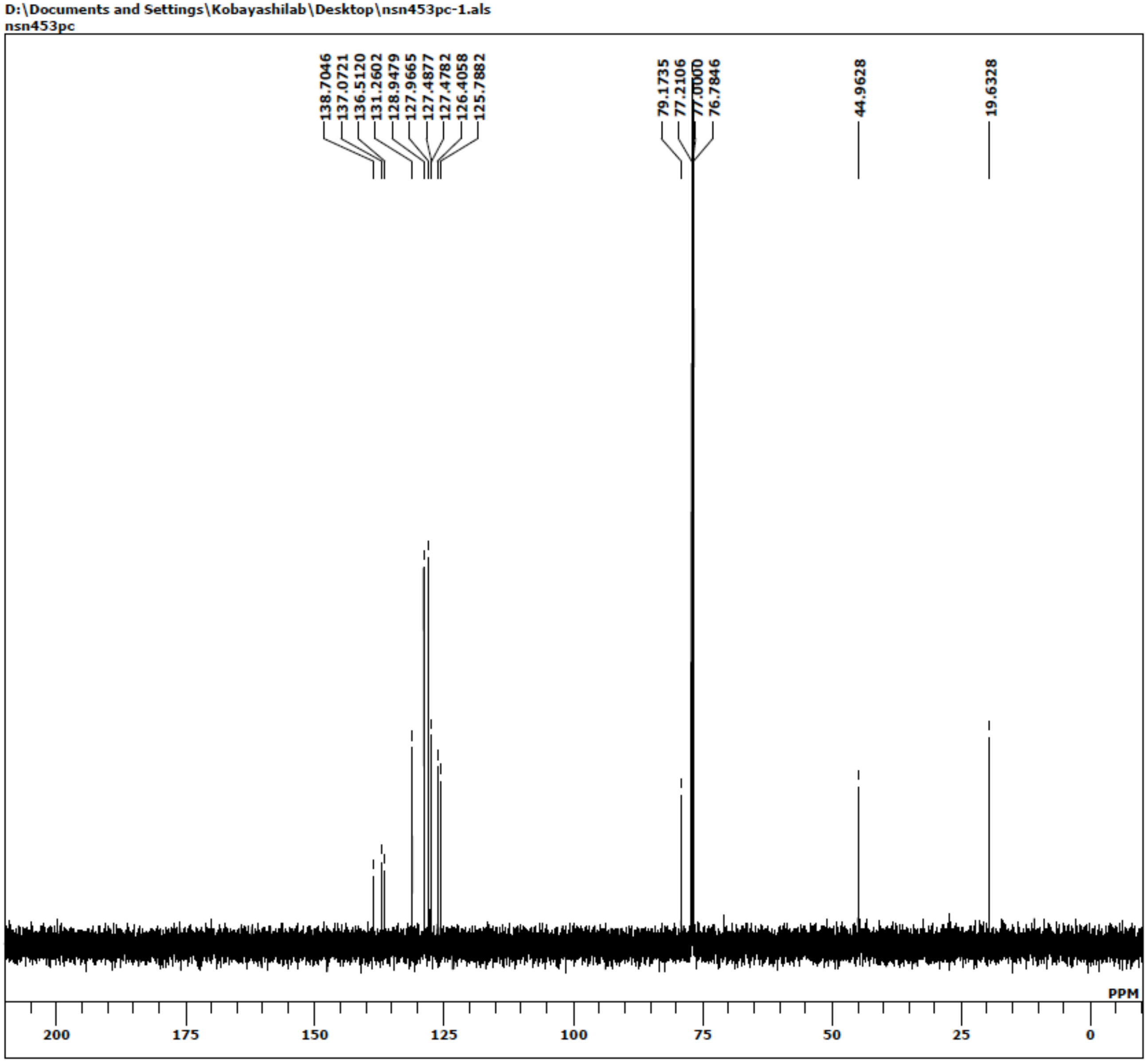
D:\Documents and Settings\Kobayashilab\Desktop\nsn453pc-1.als
nsn453pc
138.7046
137.0721
136.5120
131.2602
128.9479
127.9665
127.4877
127.4782
126.4058
125.7882
79.1735
77.2106
77.0000
76.7846
44.9628
19.6328
PPM
200
175
150
125
100
75
50
25
0

3ah 13C NMR

Sample Name : nsn454rac(for582)
OD-H, 1.0 mL/min, Hex:IPA = 3/2

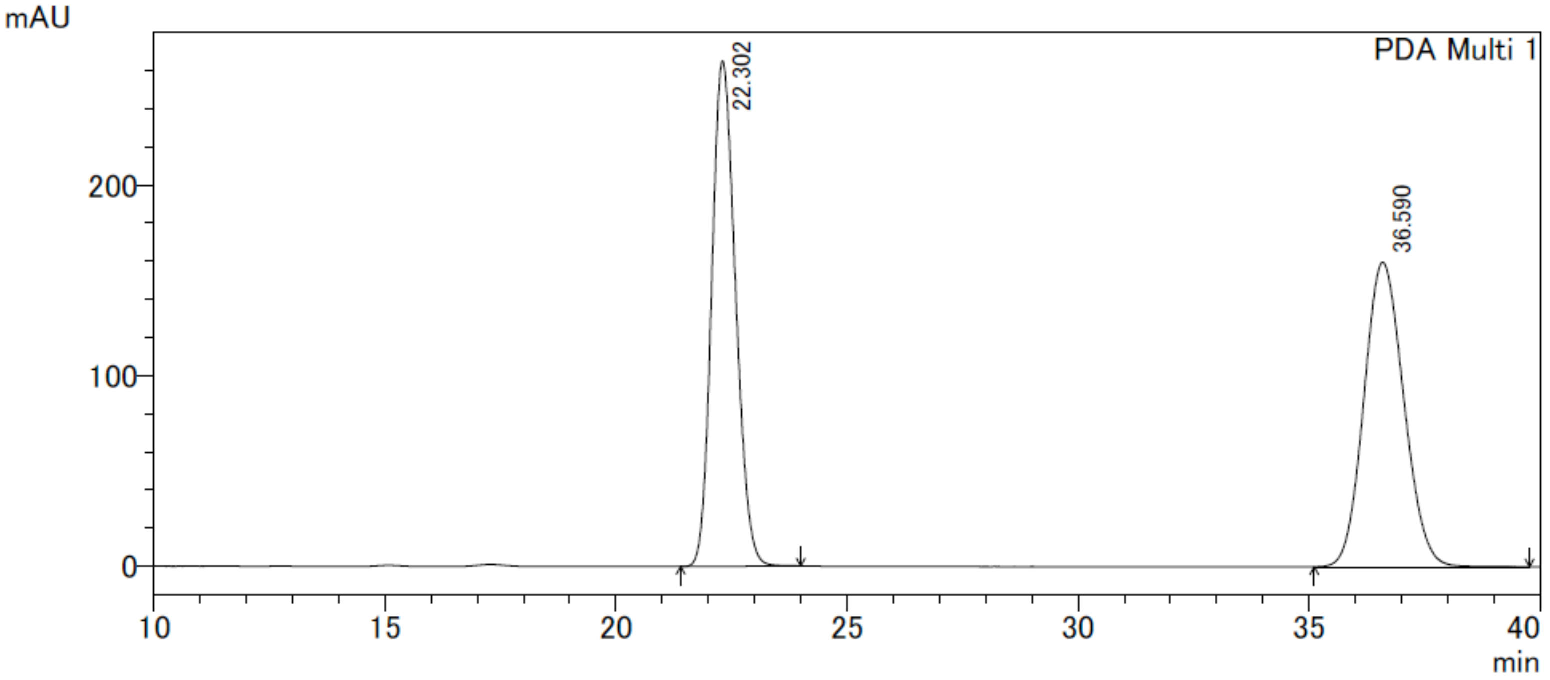

1 PDA Multi 1/220nm 4nm

PDA Ch1 220nm 4nm

Peak#	Ret. Time	Area	Height	Area%
1	22.302	9517617	265377	49.852
2	36.590	9574089	159917	50.148

3ah racemic

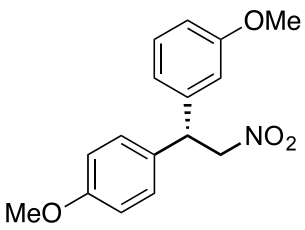
OMe
NO2
MeO

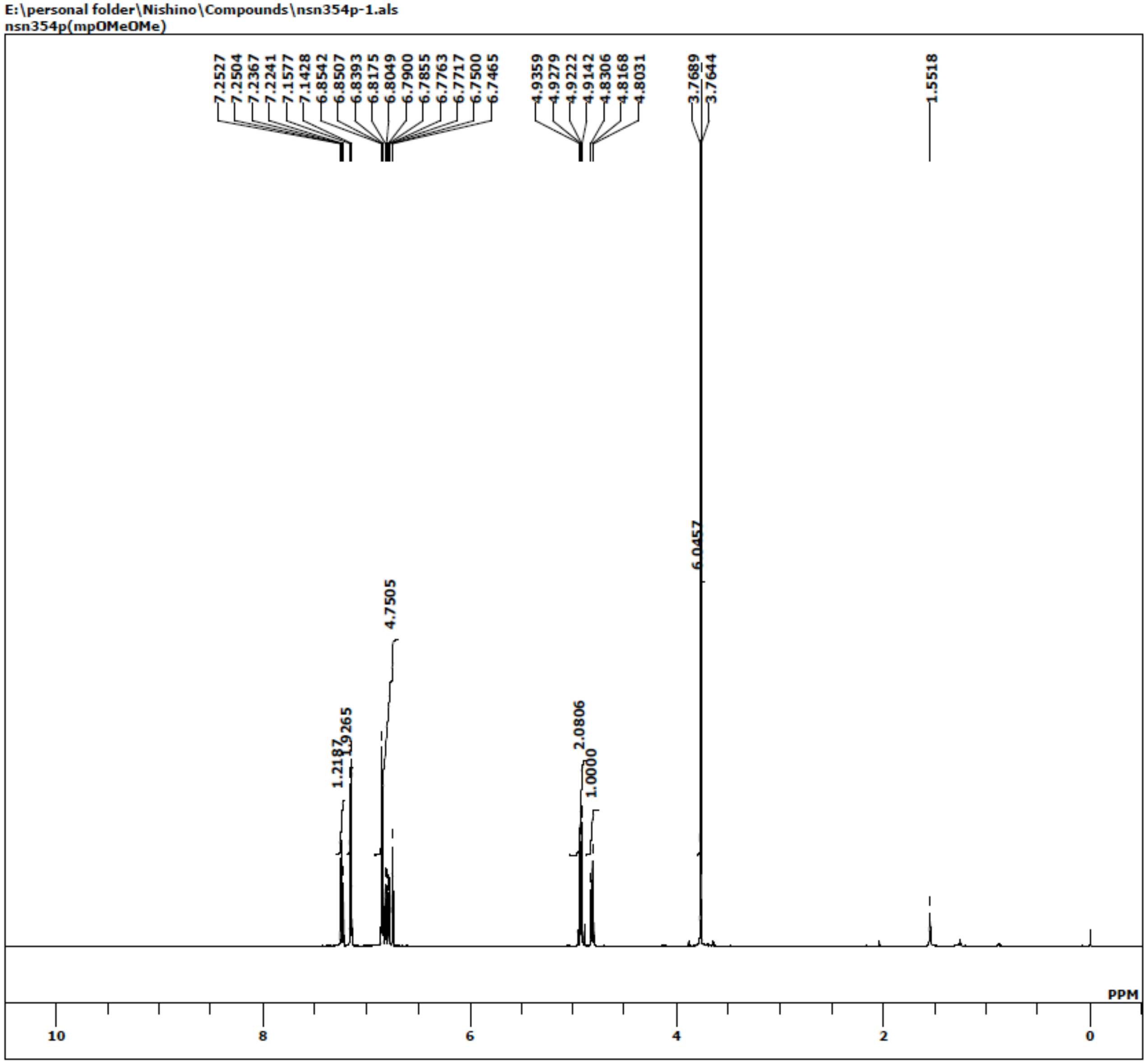
E:\personal folder\Nishino\Compounds\nsn354p-1.als
nsn354p(mpOMeOMe)
7.2527
7.2504
7.2367
7.2241
7.1577
7.1428
6.8542
6.8507
6.8393
6.8175
6.8049
6.7900
6.7855
6.7763
6.7717
6.7500
6.7465
4.9359
4.9279
4.9222
4.9142
4.8306
4.8168
4.8031
3.7689
3.7644
1.5518
6.0457
4.7505
2.0806
1.0000
1.2187
1.9265
PPM
10
8
6
4
2
0

3ba 1H NMR

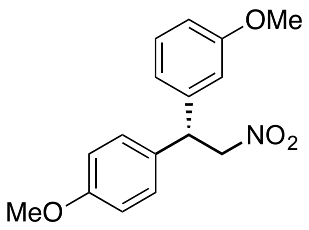

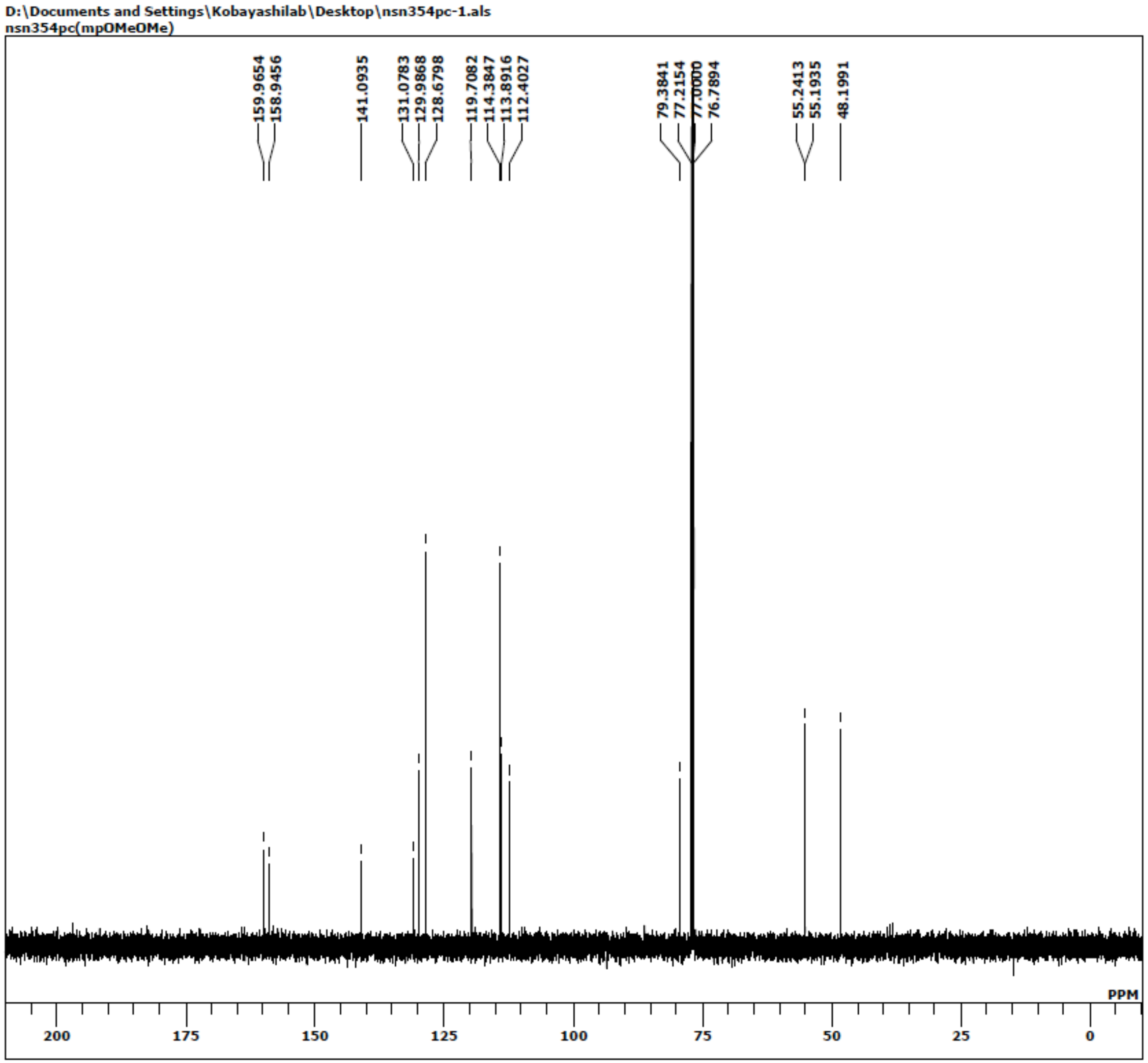

D:\Documents and Settings\Kobayashilab\Desktop\nsn354pc-1.als
nsn354pc(mpOMeOMe)
159.9654
158.9456
141.0935
131.0783
129.9868
128.6798
119.7082
114.3847
113.8916
112.4027
79.3841
77.2154
77.0000
76.7894
55.2413
55.1935
48.1991
PPM
200
175
150
125
100
75
50
25
0

3ba 13C NMR

Sample Name : nsn364rac(for562)
OD-H, 1.0 mL/min, Hex:IPA = 1/1

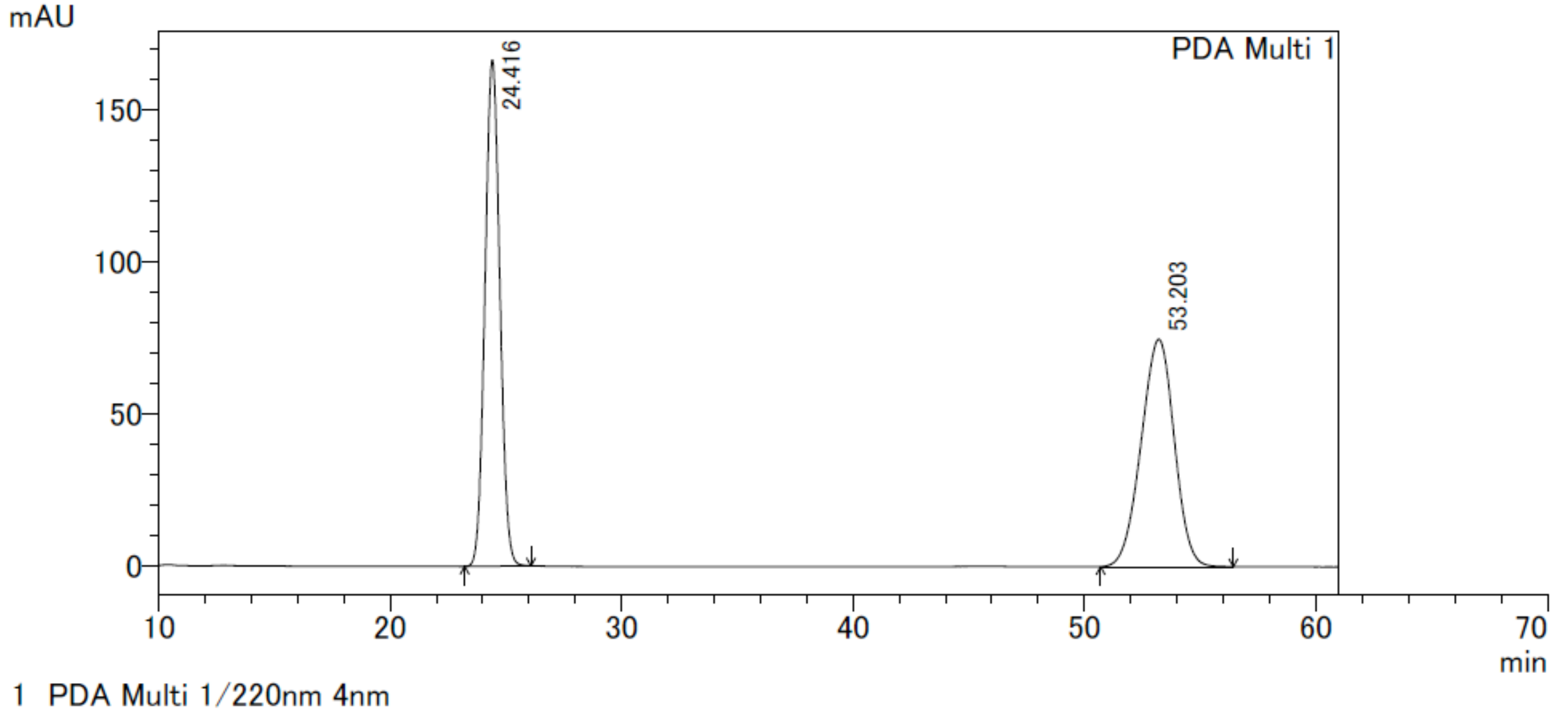

PDA Ch1 220nm 4nm

Peak#	Ret. Time	Area	Height	Area%
1	24.416	7440327	166596	49.929
2	53.203	7461443	74852	50.071

3ba racemic

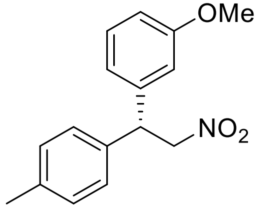

OMe
NO_2

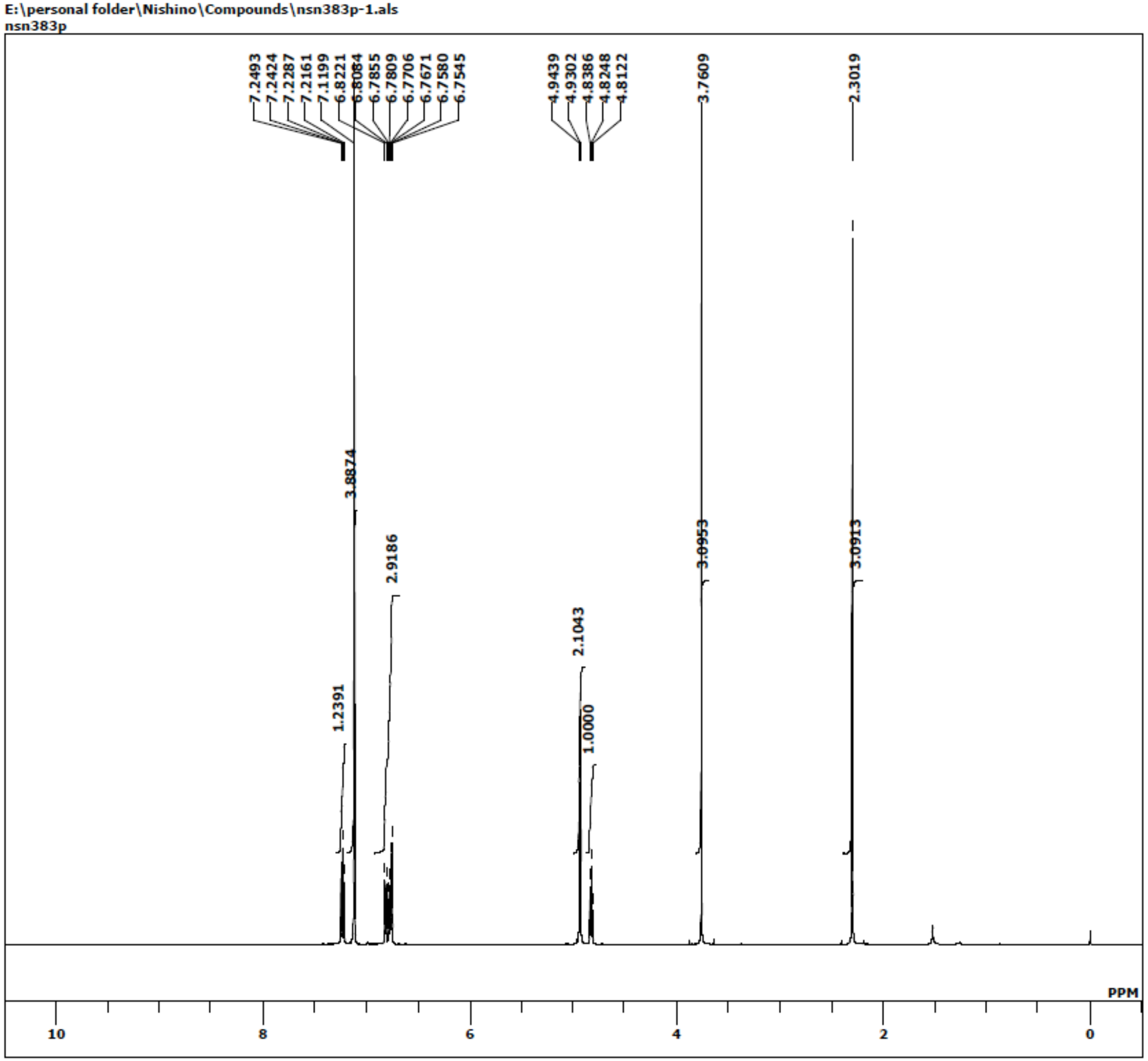

E:\personal folder\Nishino\Compounds\nsn383p-1.als
nsn383p
7.2493
7.2424
7.2287
7.2161
7.1199
6.8221
6.8084
6.7855
6.7809
6.7706
6.7671
6.7580
6.7545
4.9439
4.9302
4.8386
4.8248
4.8122
3.7609
2.3019
3.8874
2.9186
1.2391
2.1043
1.0000
3.0953
3.0913
PPM
10
8
6
4
2
0

3ea 1H NMR

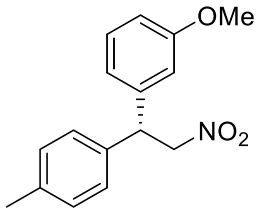

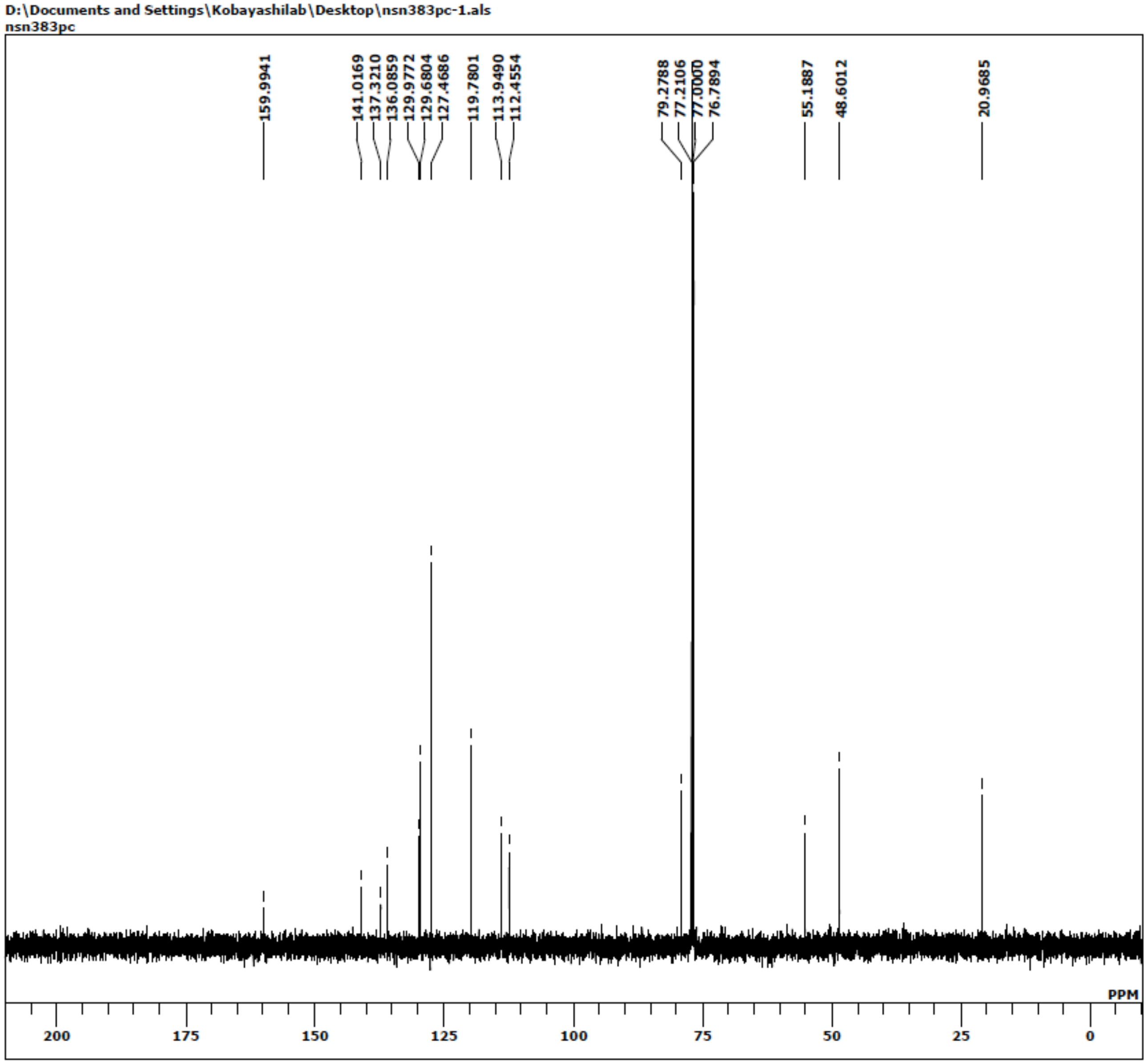
D:\Documents and Settings\Kobayashilab\Desktop\nsn383pc-1.als
nsn383pc
159.9941
141.0169
137.3210
136.0859
129.9772
129.6804
127.4686
119.7801
113.9490
112.4554
79.2788
77.2106
77.0000
76.7894
55.1887
48.6012
20.9685
PPM
200
175
150
125
100
75
50
25
0

3ea 13C NMR

Sample Name : nsn390rac(for566)
OD-H, 1.0 mL/min, Hex:IPA = 3/2

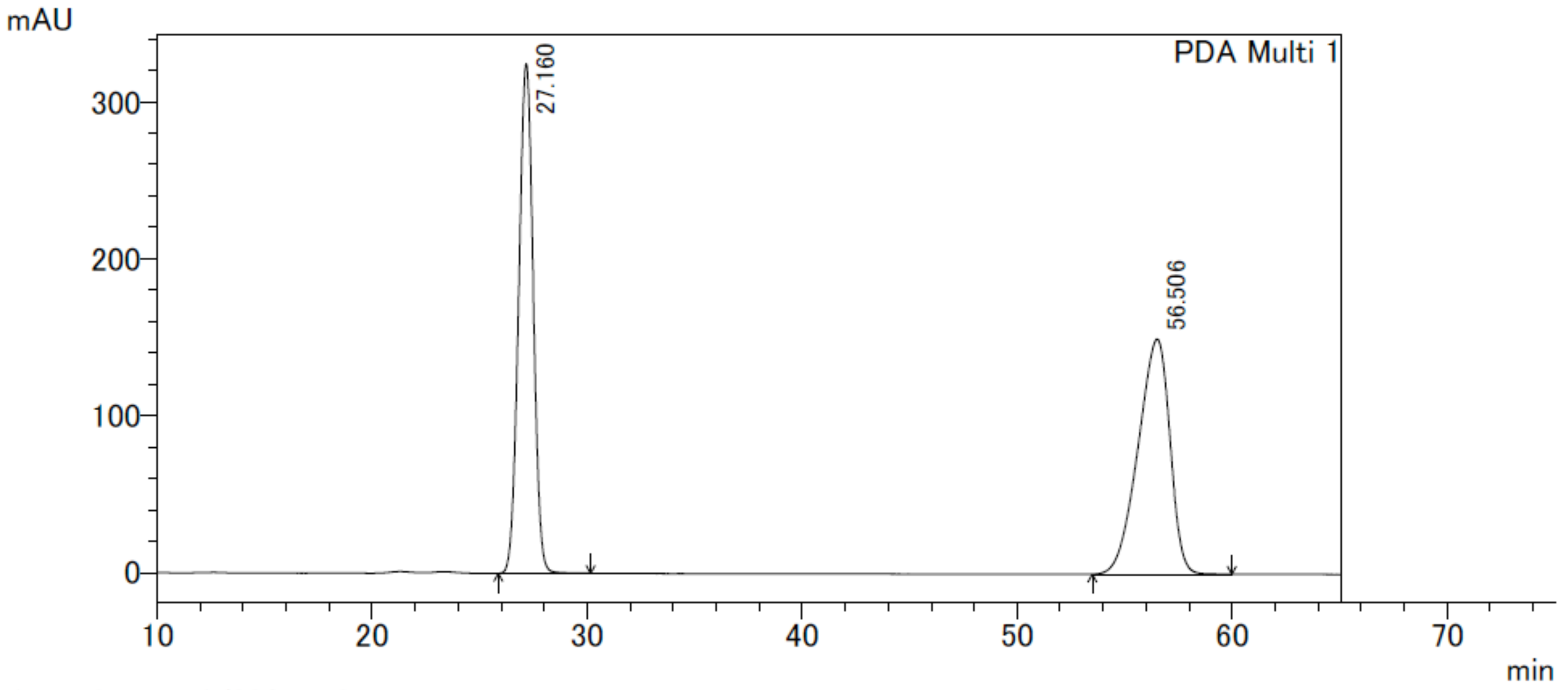

1 PDA Multi 1/220nm 4nm

PDA Ch1 220nm 4nm

Peak#	Ret. Time	Area	Height	Area%
1	27.160	15333372	324629	49.907
2	56.506	15390255	149693	50.093

3ea racemic

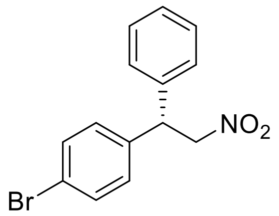

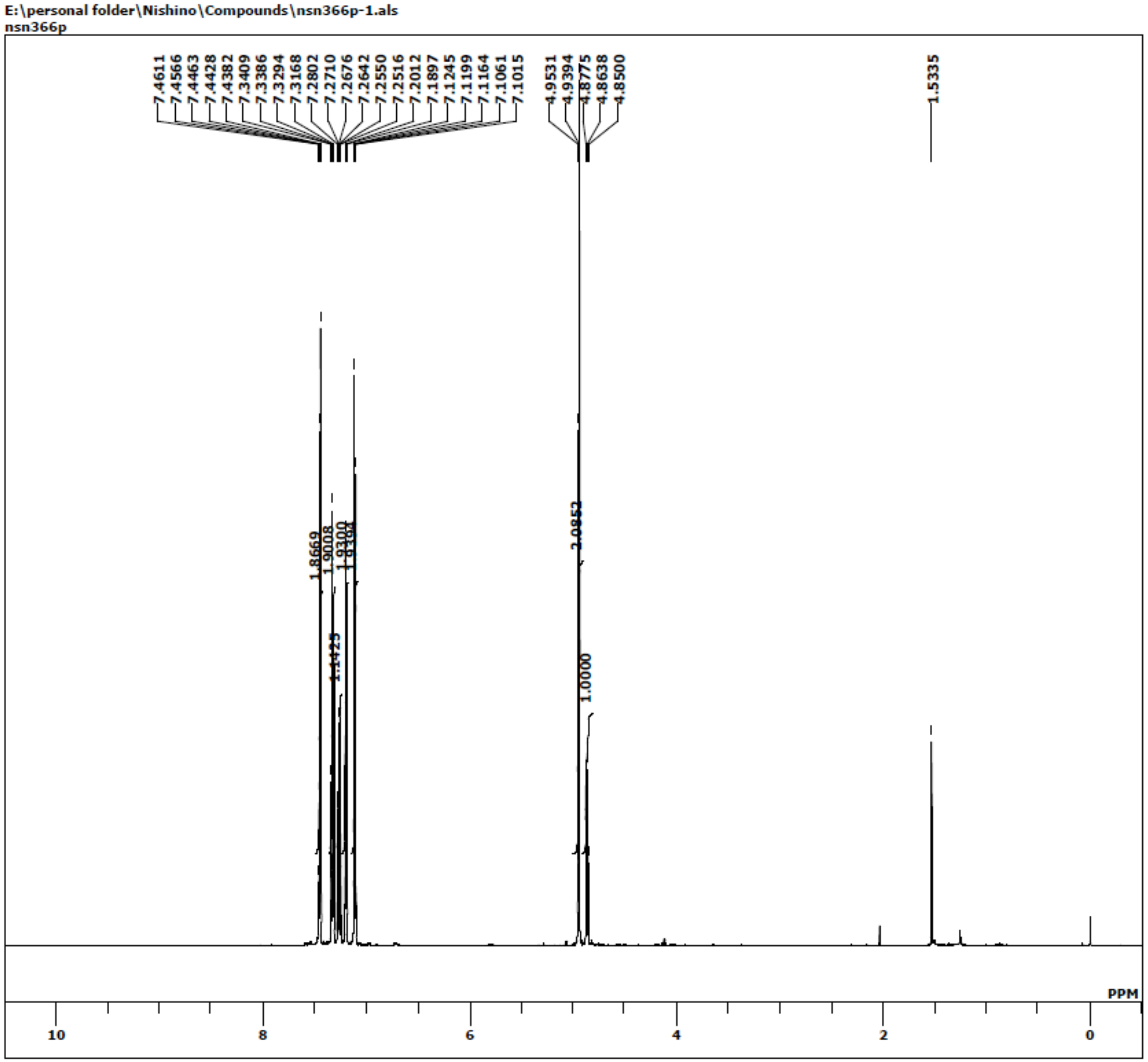

E:\personal folder\Nishino\Compounds\nsn366p-1.als
nsn366p
7.4611
7.4566
7.4463
7.4428
7.4382
7.3409
7.3386
7.3294
7.3168
7.2802
7.2710
7.2676
7.2642
7.2550
7.2516
7.2012
7.1897
7.1245
7.1199
7.1164
7.1061
7.1015
4.9531
4.9394
4.8775
4.8638
4.8500
1.5335
1.8669
1.9008
1.1425
1.9300
2.0852
1.0000
PPM
10
8
6
4
2
0

3fb 1H NMR

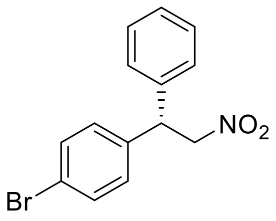

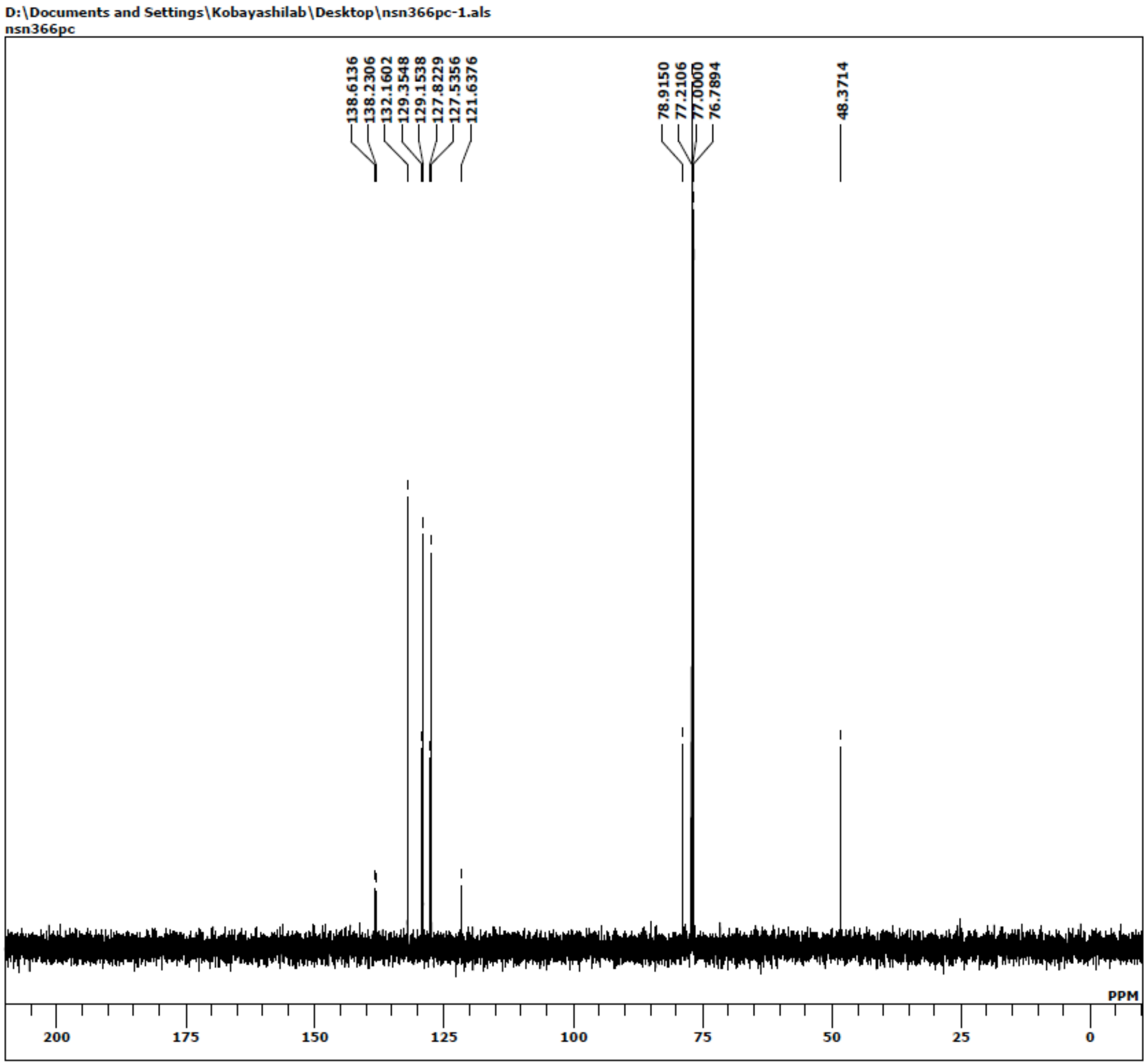
D:\Documents and Settings\Kobayashilab\Desktop\nsn366pc-1.als
nsn366pc
138.6136
138.2306
132.1602
129.3548
129.1538
127.8229
127.5356
121.6376
78.9150
77.2106
77.0000
76.7894
48.3714
PPM
200
175
150
125
100
75
50
25
0

3fb 13C NMR

Sample Name : nsn563
OD-H, 1.0 mL/min, Hex:IPA = 3/2

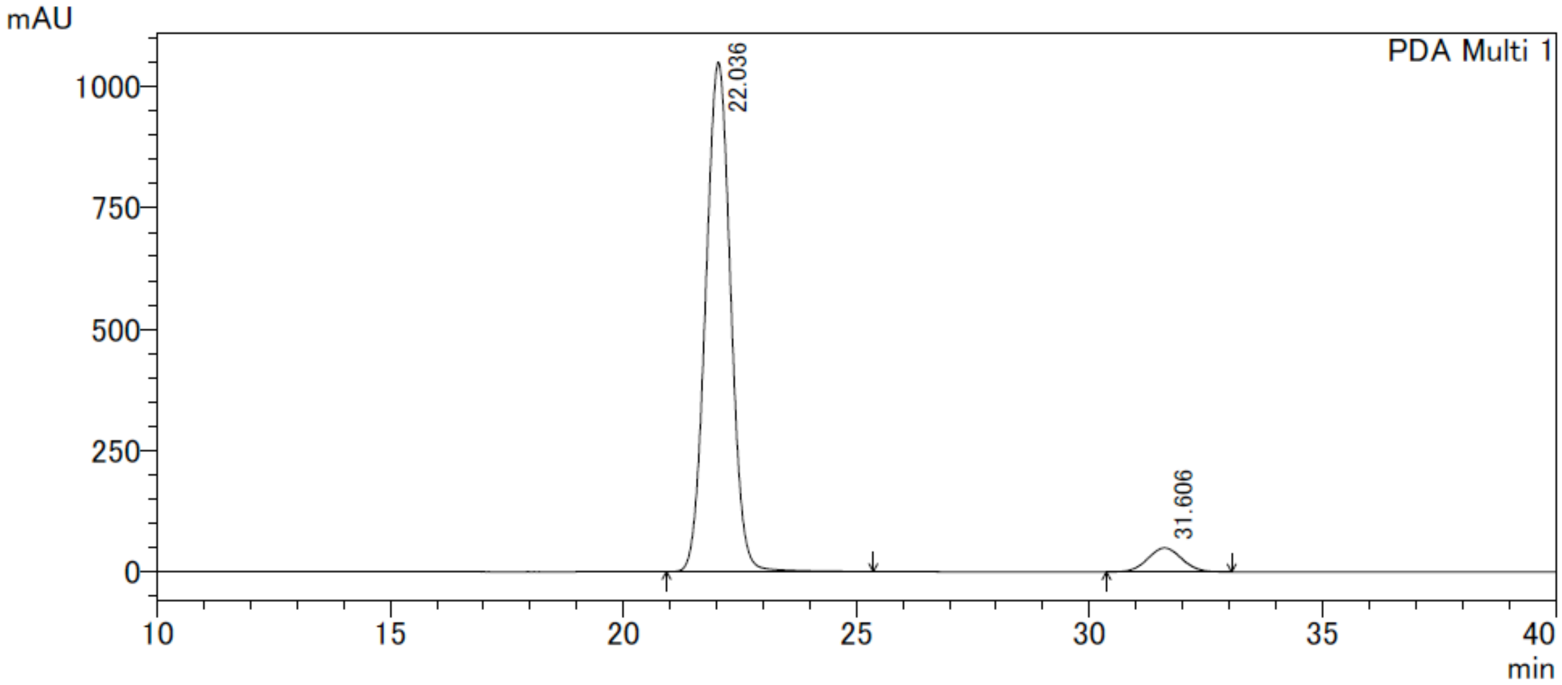

1 PDA Multi 1/220nm 4nm

PDA Ch1 220nm 4nm

Peak#	Ret. Time	Area	Height	Area%
1	22.036	39063611	1050953	93.983
2	31.606	2500839	49153	6.017

3fb HPLC

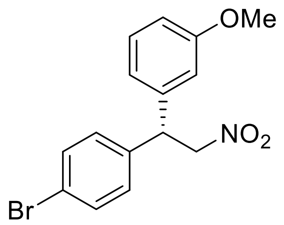

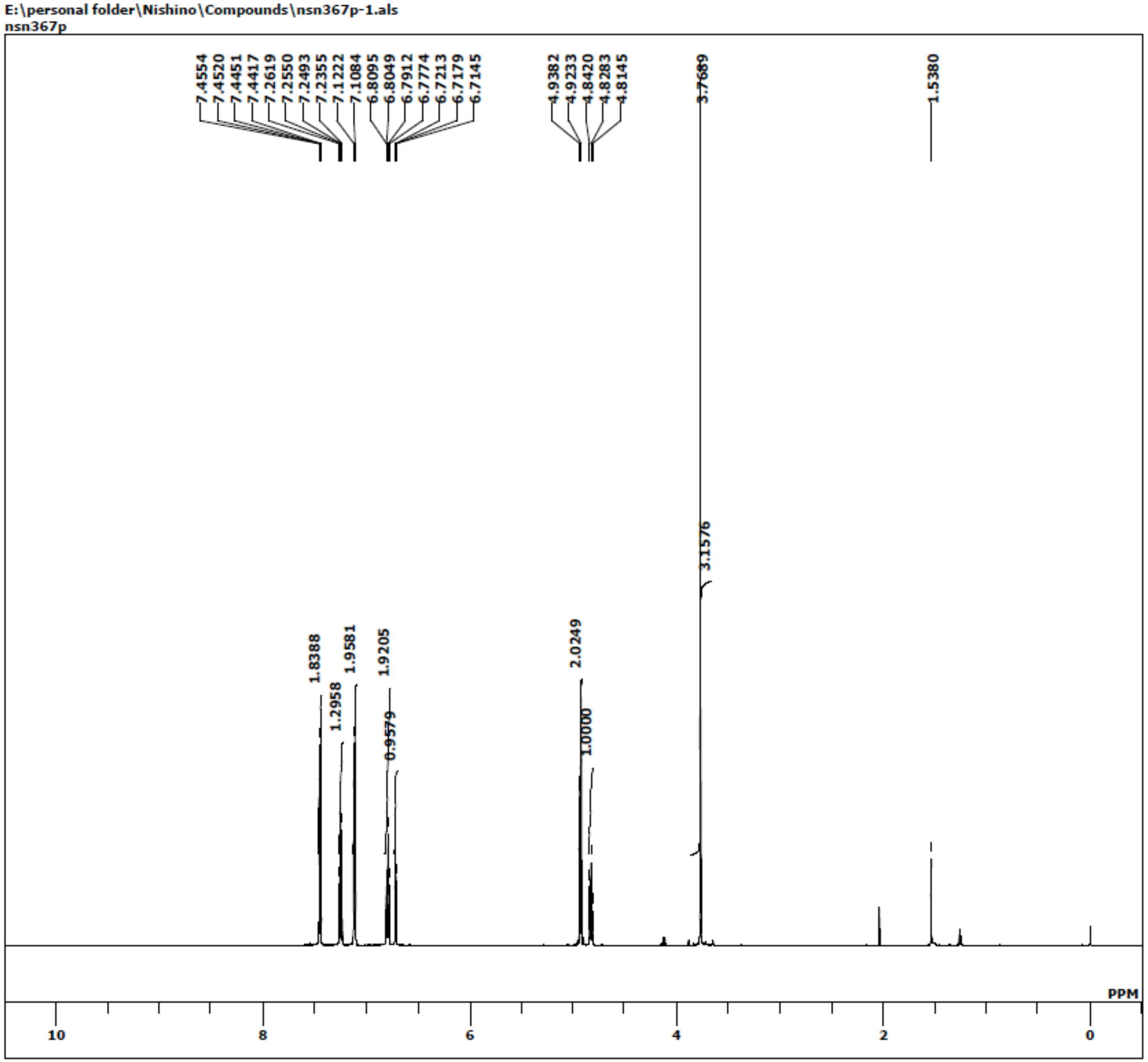
E:\personal folder\Nishino\Compounds\nsn367p-1.als
nsn367p
7.4554
7.4520
7.4451
7.4417
7.2619
7.2550
7.2493
7.2355
7.1222
7.1084
6.8095
6.8049
6.7912
6.7774
6.7213
6.7179
6.7145
4.9382
4.9233
4.8420
4.8283
4.8145
3.7689
1.5380
3.1576
1.8388
1.2958
1.9581
1.9205
0.9579
2.0249
1.0000
PPM
10
8
6
4
2
0

3fa 1H NMR

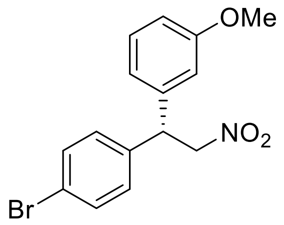

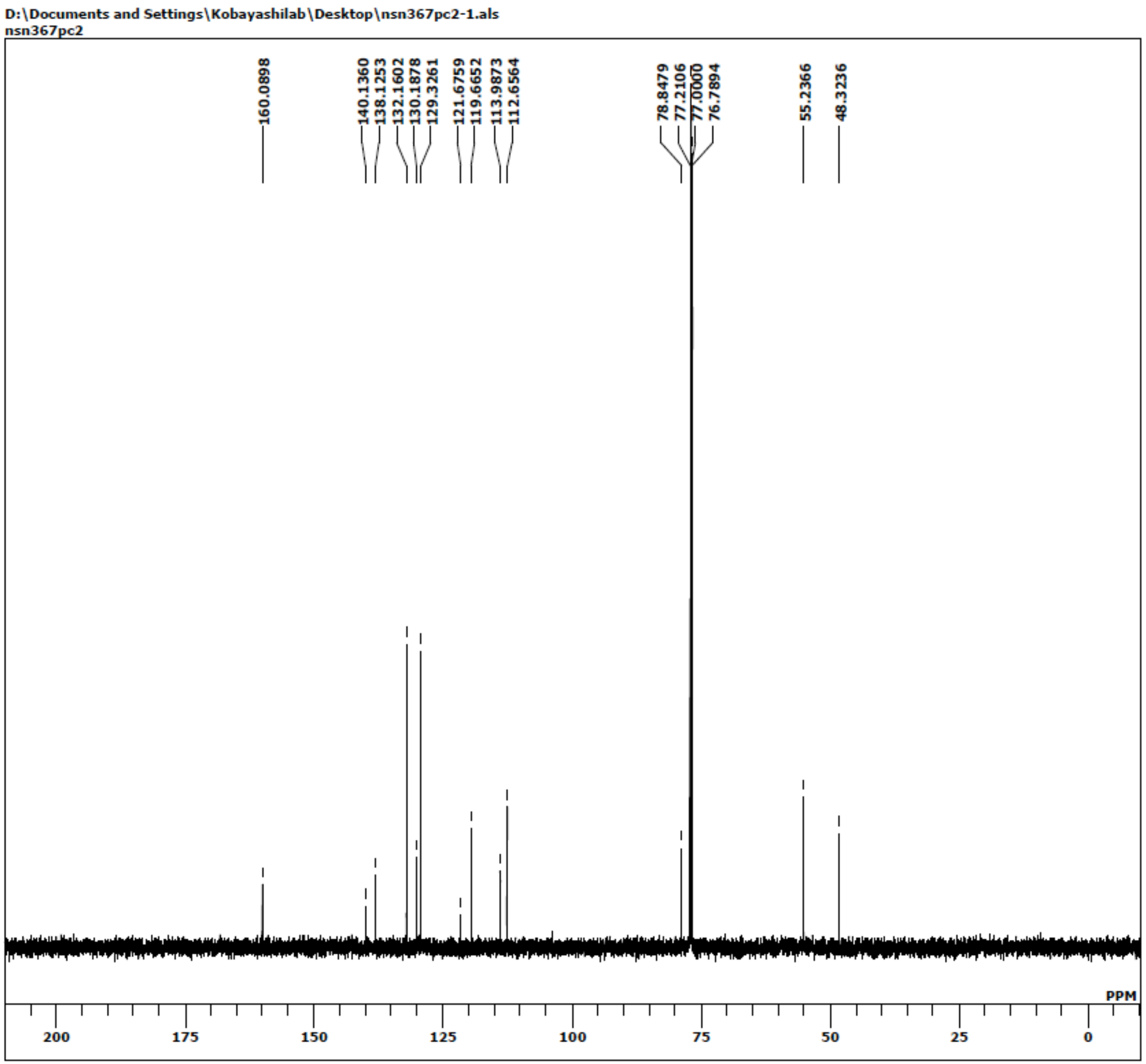

D:\Documents and Settings\Kobayashilab\Desktop\nsn367pc2-1.als
nsn367pc2
160.0898
140.1360
138.1253
132.1602
130.1878
129.3261
121.6759
119.6652
113.9873
112.6564
78.8479
77.2106
77.0000
76.7894
55.2366
48.3236
PPM
200
175
150
125
100
75
50
25
0

3fa 13C NMR

Sample Name : nsn370rac(for564)
OD-H, 1.0 mL/min, Hex:IPA = 1/1

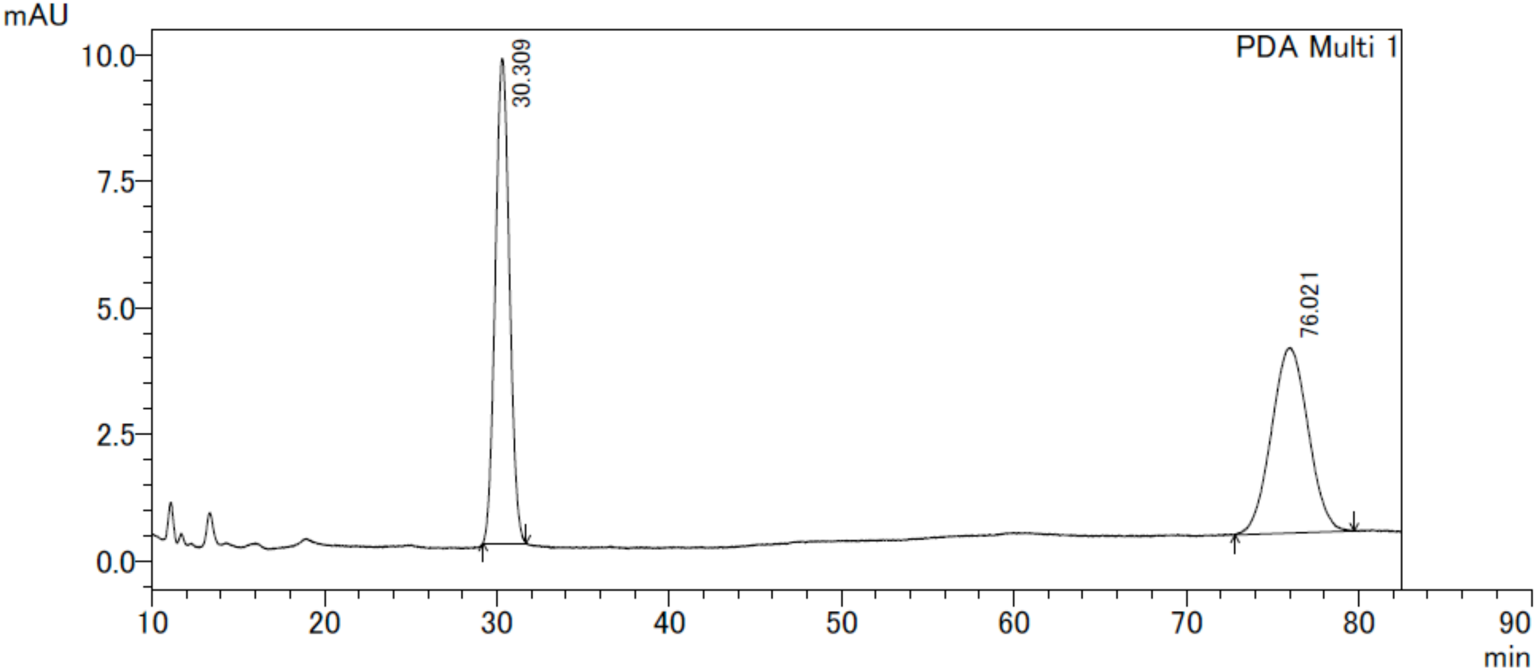

1 PDA Multi 1/220nm 4nm

PDA Ch1 220nm 4nm

Peak#	Ret. Time	Area	Height	Area%
1	30.309	544229	9578	50.083
2	76.021	542421	3660	49.917

3fa racemic

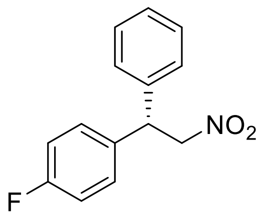

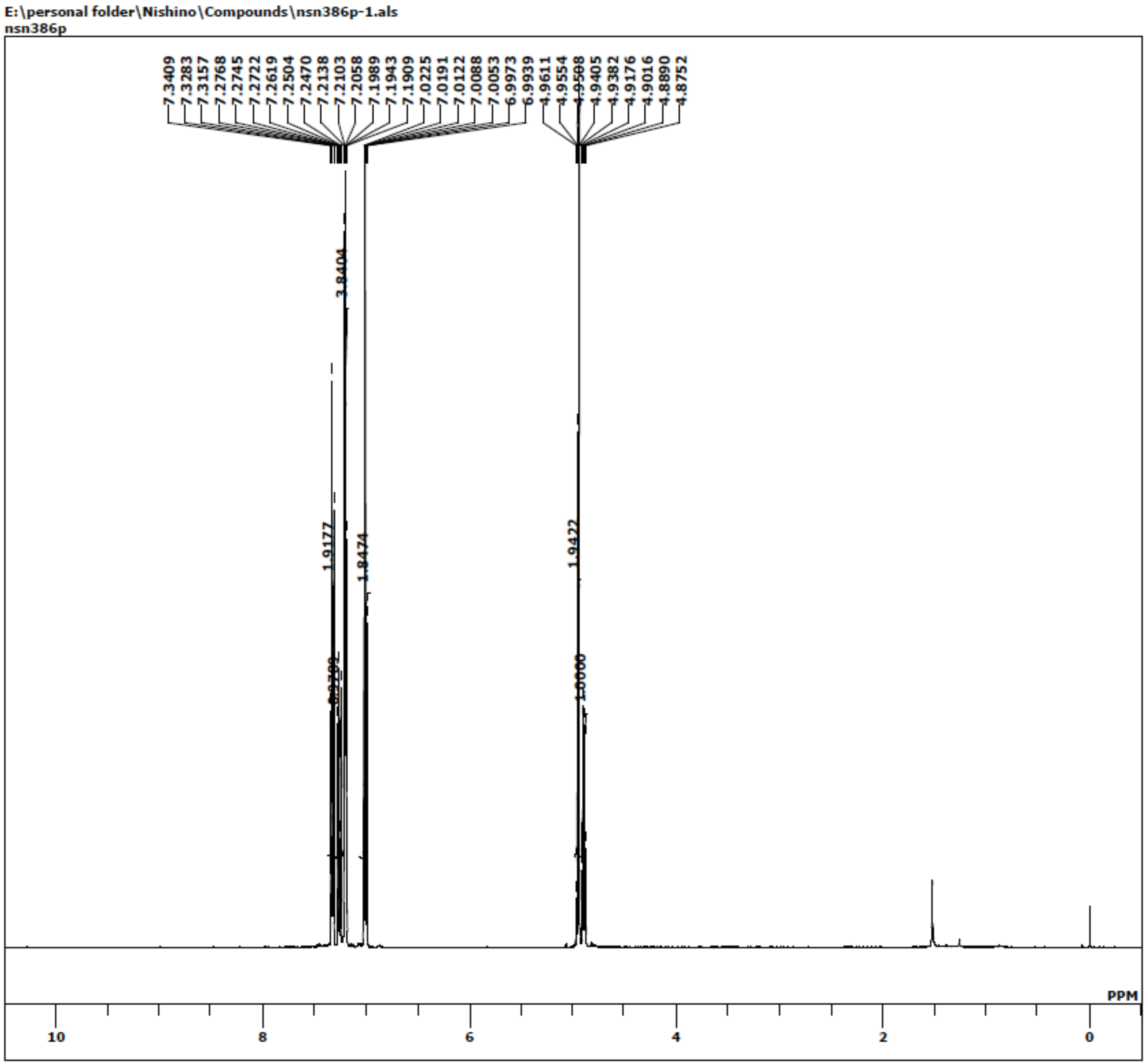

E:\personal folder\Nishino\Compounds\nsn386p-1.als
nsn386p
7.3409
7.3283
7.3157
7.2768
7.2745
7.2722
7.2619
7.2504
7.2470
7.2138
7.2103
7.2058
7.1989
7.1943
7.1909
7.0225
7.0191
7.0122
7.0088
7.0053
6.9973
6.9939
4.9611
4.9554
4.9508
4.9405
4.9382
4.9176
4.9016
4.8890
4.8752
3.8404
1.9177
1.8474
1.9422
1.0000
PPM
10
8
6
4
2
0

3gb 1H NMR

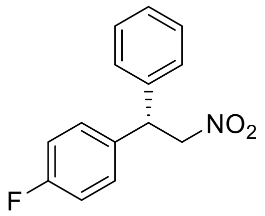
NO2
F

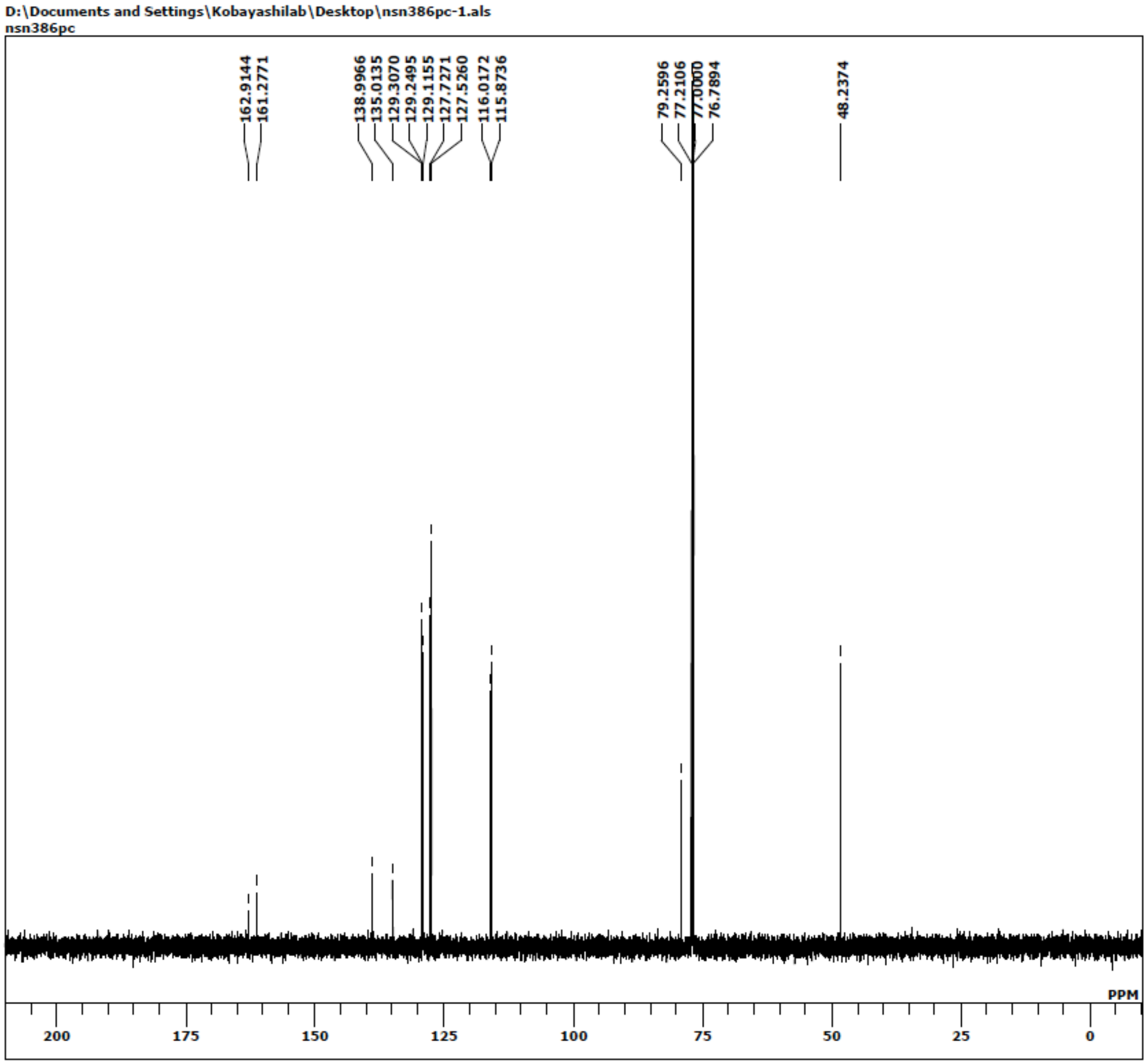
D:\Documents and Settings\Kobayashilab\Desktop\nsn386pc-1.als
nsn386pc
162.9144
161.2771
138.9966
135.0135
129.3070
129.2495
129.1155
127.7271
127.5260
116.0172
115.8736
79.2596
77.2106
77.0000
76.7894
48.2374
PPM
200
175
150
125
100
75
50
25
0

3gb 13C NMR

Sample Name : nsn401rac(for567and583)
OD-H, 1.0 mL/min, Hex:IPA = 3/2

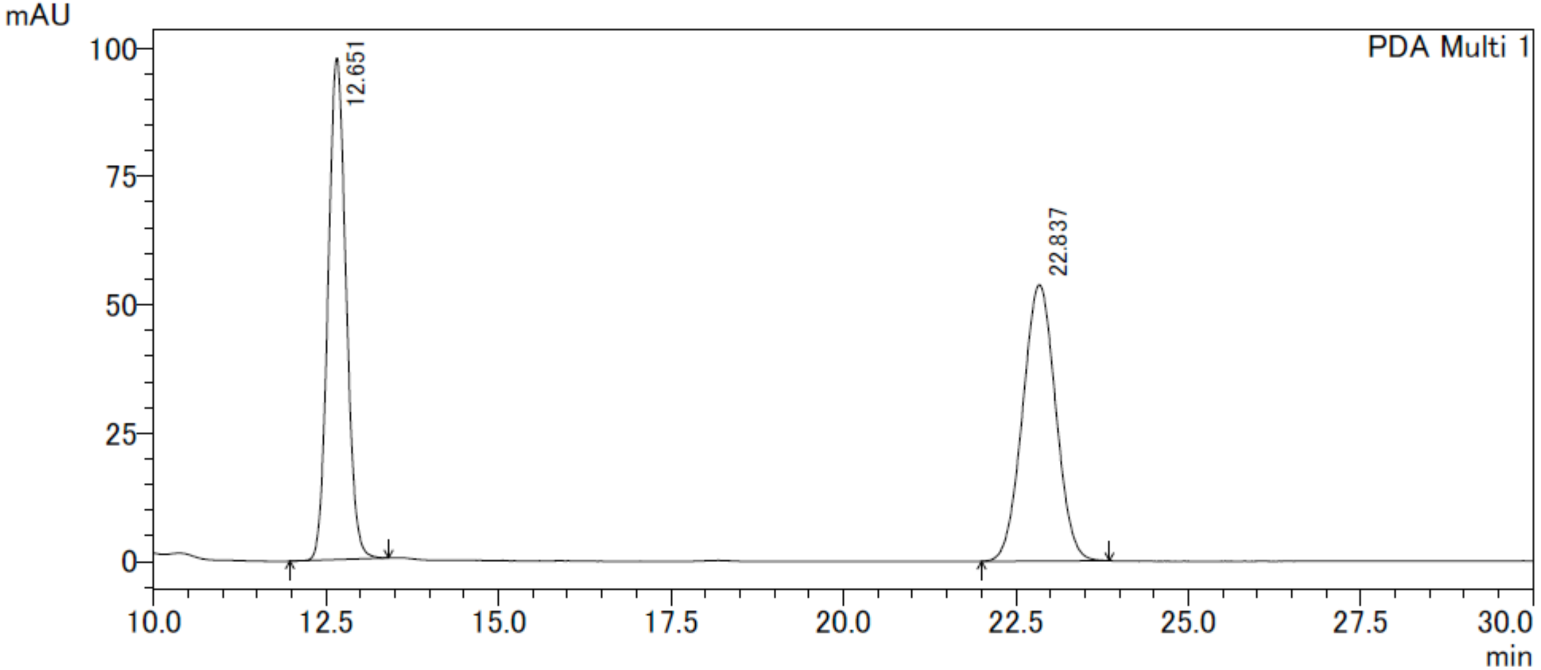

1 PDA Multi 1/220nm 4nm

PDA Ch1 220nm 4nm

Peak#	Ret. Time	Area	Height	Area%
1	12.651	1777954	97729	49.742
2	22.837	1796402	53828	50.258

3gb racemic

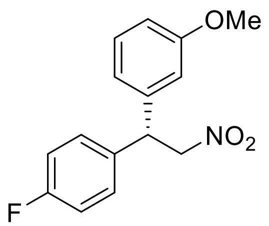
OMe
NO_2
F

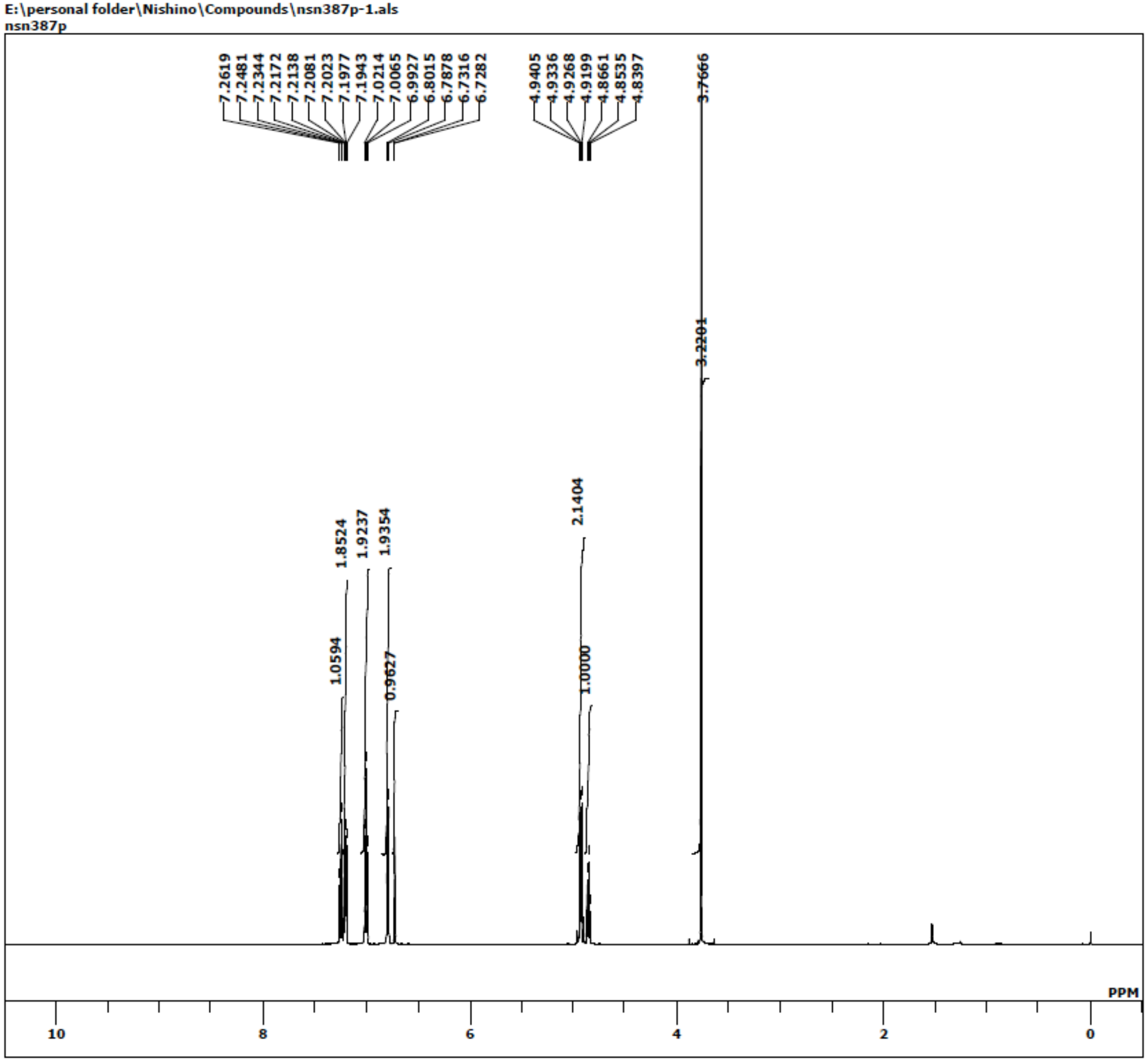
E:\personal folder\Nishino\Compounds\nsn387p-1.als
nsn387p
7.2619
7.2481
7.2344
7.2172
7.2138
7.2081
7.2023
7.1977
7.1943
7.0214
7.0065
6.9927
6.8015
6.7878
6.7316
6.7282
4.9405
4.9336
4.9268
4.9199
4.8661
4.8535
4.8397
3.7666
3.2201
2.1404
1.8524
1.9237
1.9354
1.0594
0.9627
1.0000
PPM
10
8
6
4
2
0

3ga 1H NMR

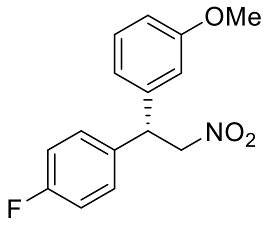
OMe
NO2
F

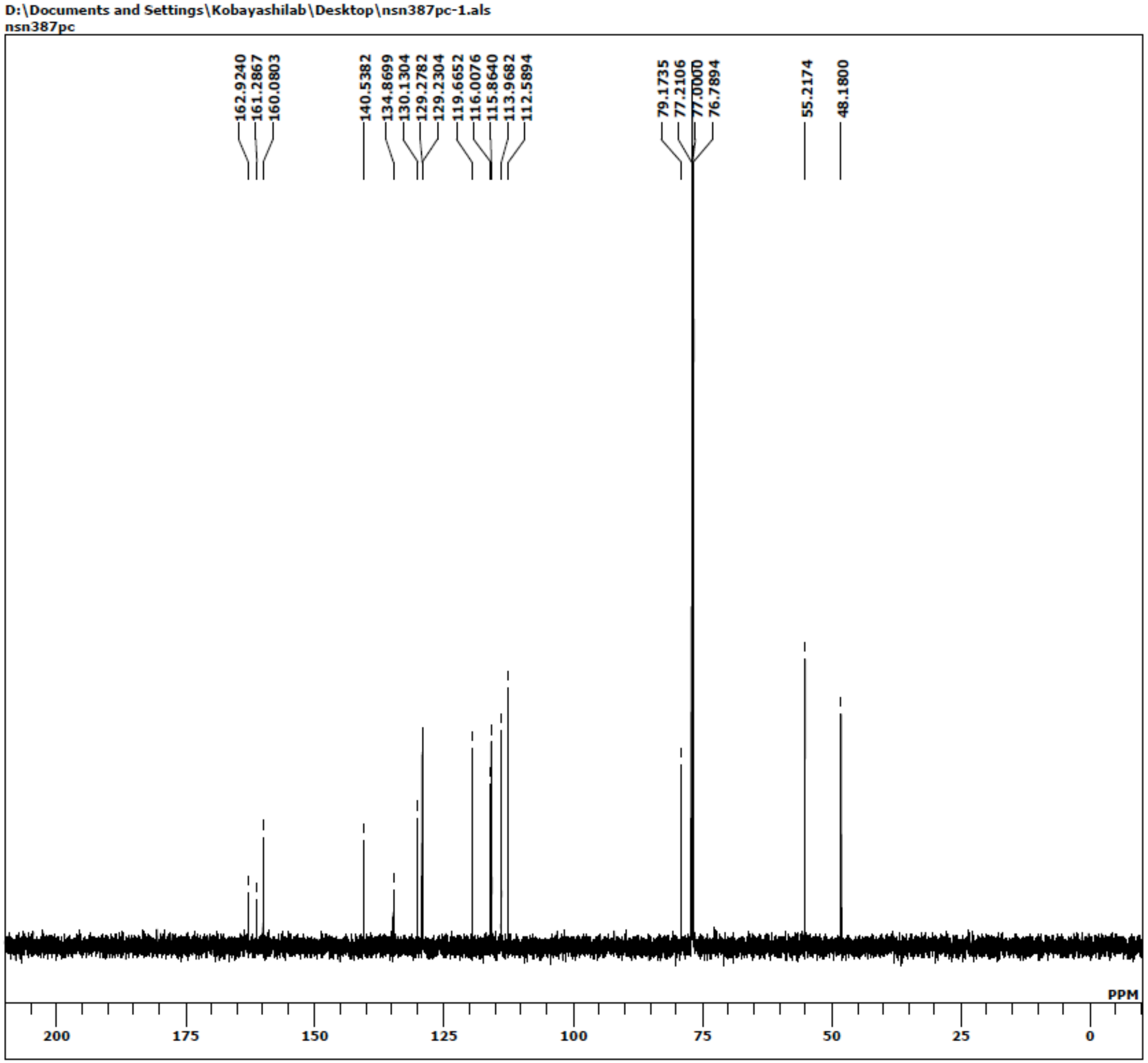
D:\Documents and Settings\Kobayashilab\Desktop\nsn387pc-1.als
nsn387pc
162.9240
161.2867
160.0803
140.5382
134.8699
130.1304
129.2782
129.2304
119.6652
116.0076
115.8640
113.9682
112.5894
79.1735
77.2106
77.0000
76.7894
55.2174
48.1800
PPM
200
175
150
125
100
75
50
25
0

3ga 13C NMR

Sample Name : nsn379(for568)
OD-H, 1.0 mL/min, Hex:IPA = 3/2

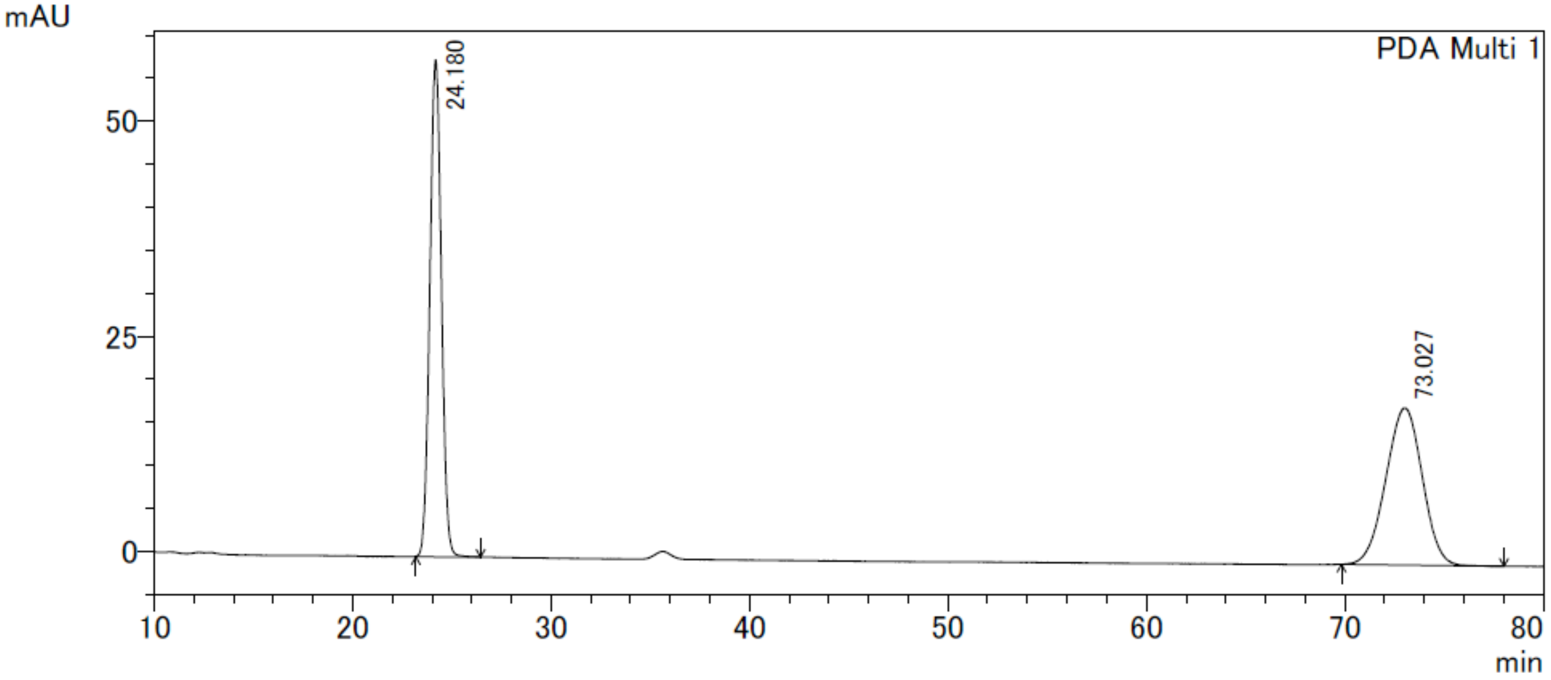

1 PDA Multi 1/220nm 4nm

PDA Ch1 220nm 4nm

Peak#	Ret. Time	Area	Height	Area%
1	24.180	2332232	57750	50.050
2	73.027	2327608	18250	49.950

3ga racemic

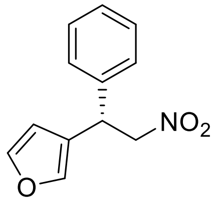

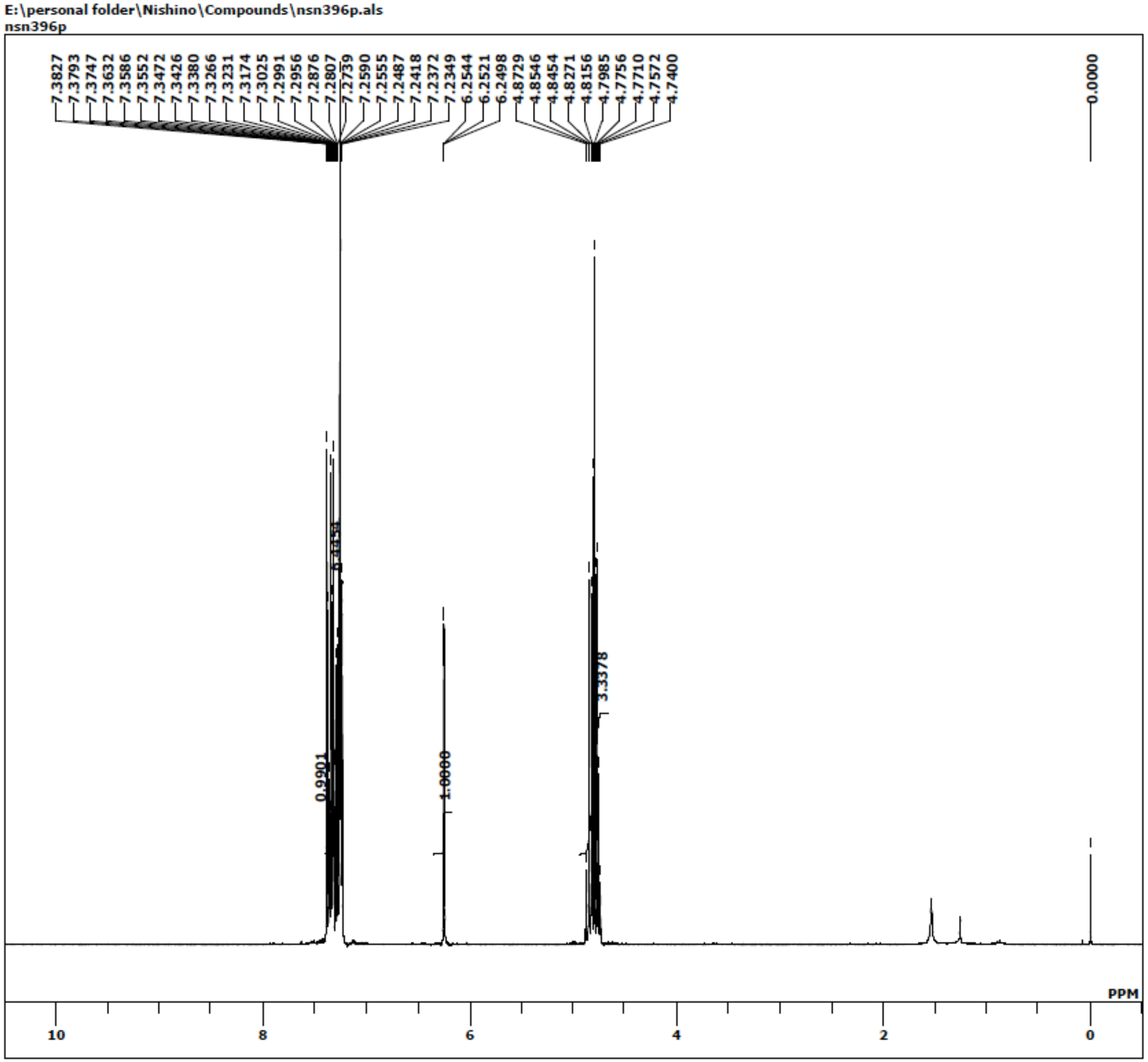
E:\personal folder\Nishino\Compounds\nsn396p.als
nsn396p
7.3827
7.3793
7.3747
7.3632
7.3586
7.3552
7.3472
7.3426
7.3380
7.3266
7.3231
7.3174
7.3025
7.2991
7.2956
7.2876
7.2807
7.2739
7.2590
7.2555
7.2487
7.2418
7.2372
7.2349
6.2544
6.2521
6.2498
4.8729
4.8546
4.8454
4.8271
4.8156
4.7985
4.7756
4.7710
4.7572
4.7400
0.0000
0.9901
1.0000
3.3378
PPM
10
8
6
4
2
0

3hb 1H NMR

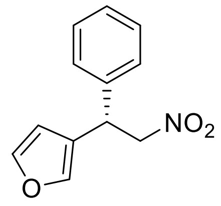

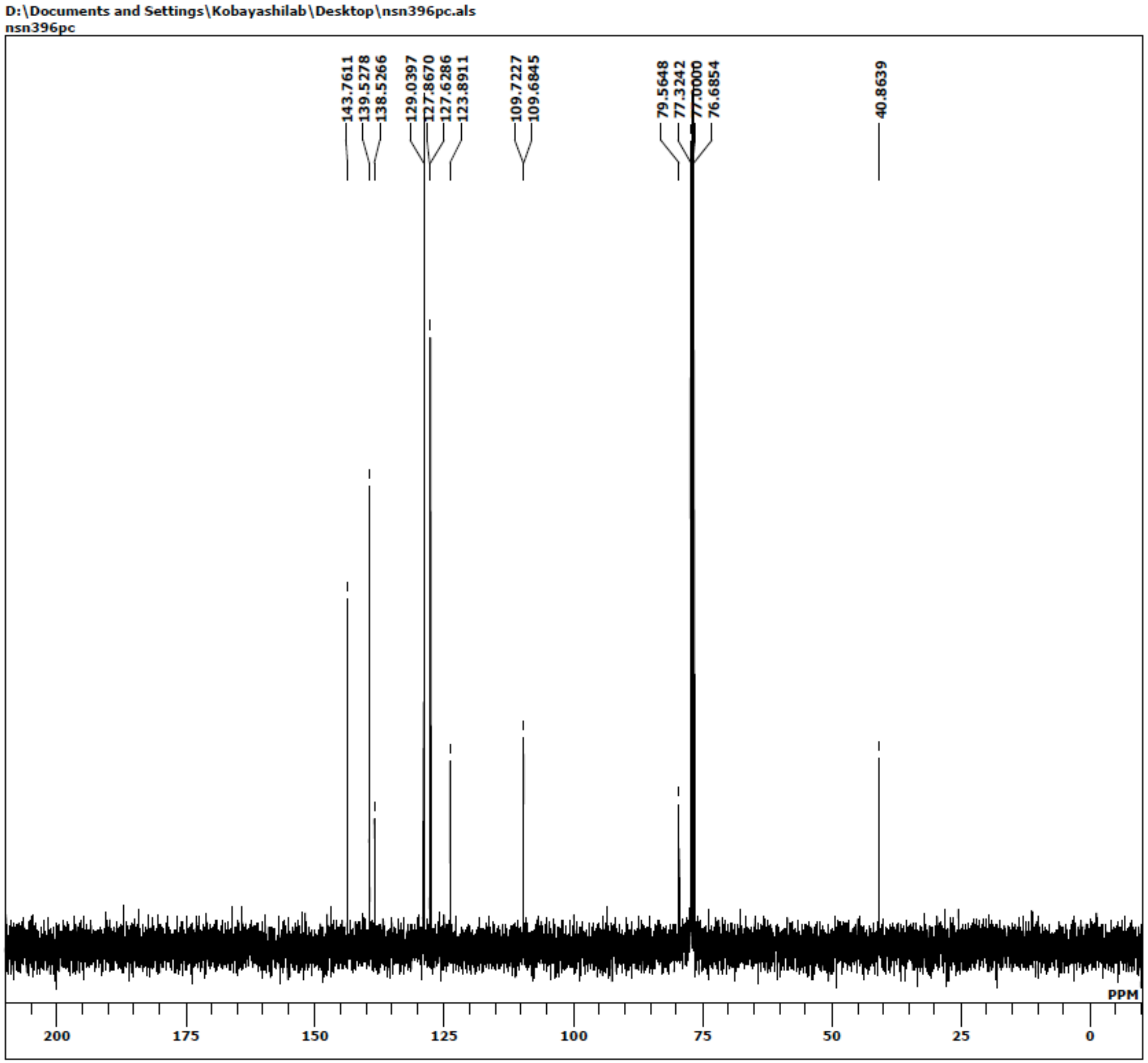

D:\Documents and Settings\Kobayashilab\Desktop\nsn396pc.als
nsn396pc
143.7611
139.5278
138.5266
129.0397
127.8670
127.6286
123.8911
109.7227
109.6845
79.5648
77.3242
77.0000
76.6854
40.8639
PPM
200
175
150
125
100
75
50
25
0

3hb 13C NMR

Sample Name : nsn399rac(for569)
OD-H, 1.0 mL/min, Hex:IPA = 3/2

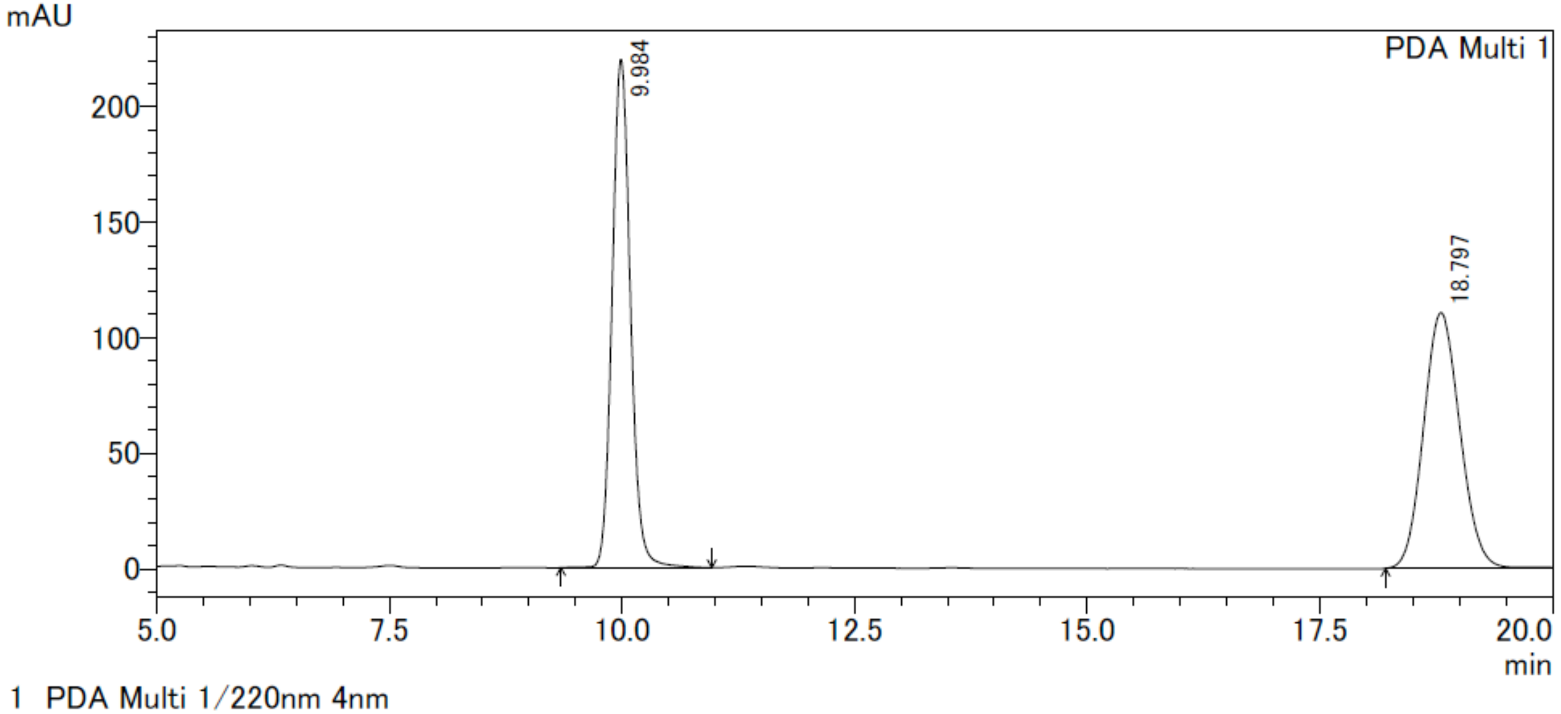

PDA Ch1 220nm 4nm

Peak#	Ret. Time	Area	Height	Area%
1	9.984	3024378	220004	50.083
2	18.797	3014361	110705	49.917

3hb racemic

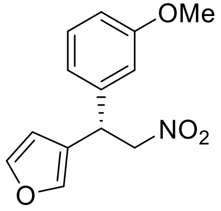
OMe
NO2
O

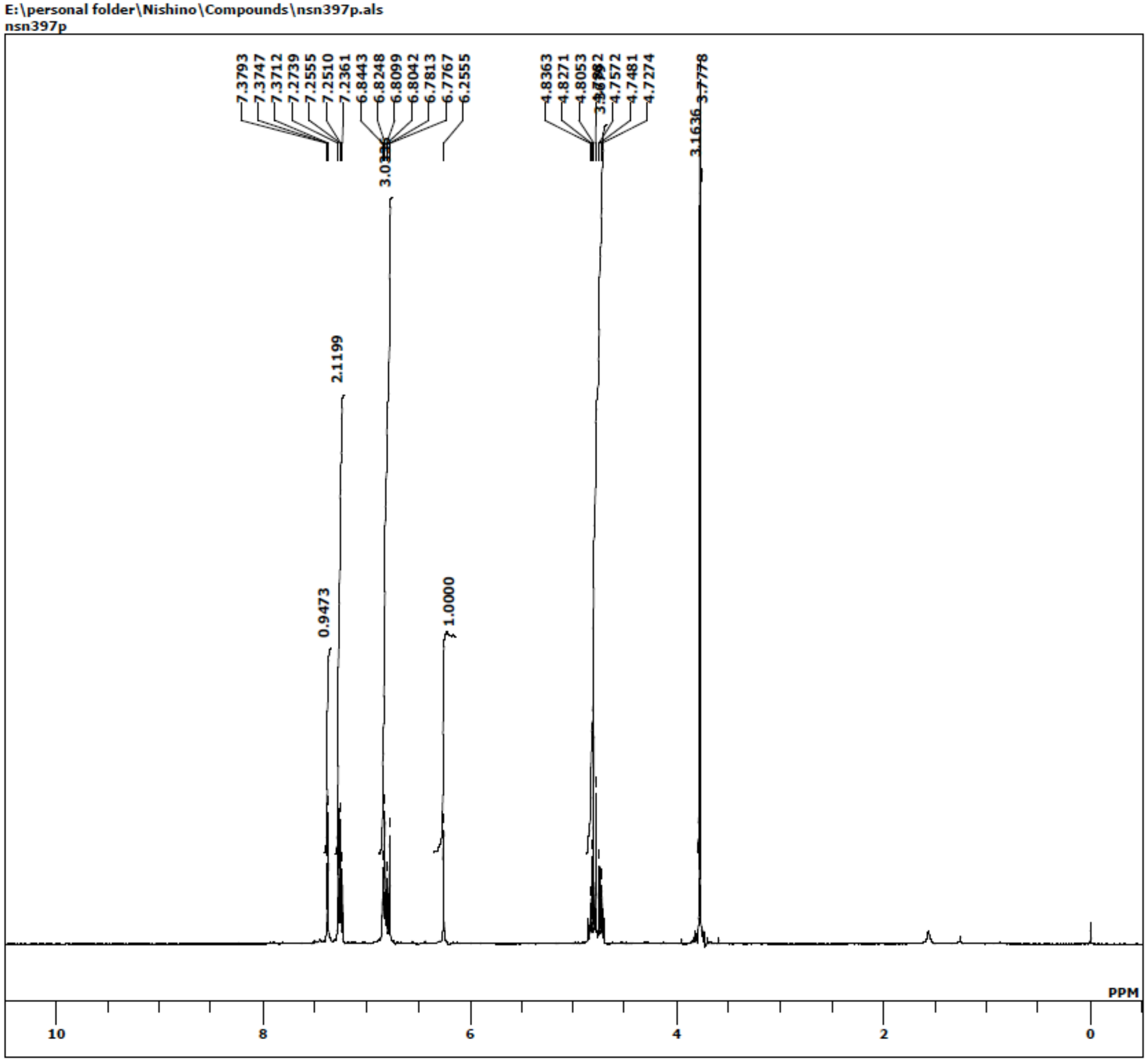
E:\personal folder\Nishino\Compounds\nsn397p.als
nsn397p
7.3793
7.3747
7.3712
7.2739
7.2555
7.2510
7.2361
6.8443
6.8248
6.8099
6.8042
6.7813
6.7767
6.2555
4.8363
4.8271
4.8053
4.7572
4.7481
4.7274
3.7778
3.1636
0.9473
2.1199
1.0000
PPM
10
8
6
4
2
0

3ha 1H NMR

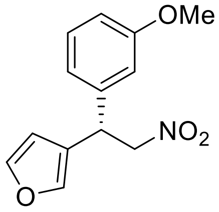
OMe
NO2
O

3ha 13C NMR

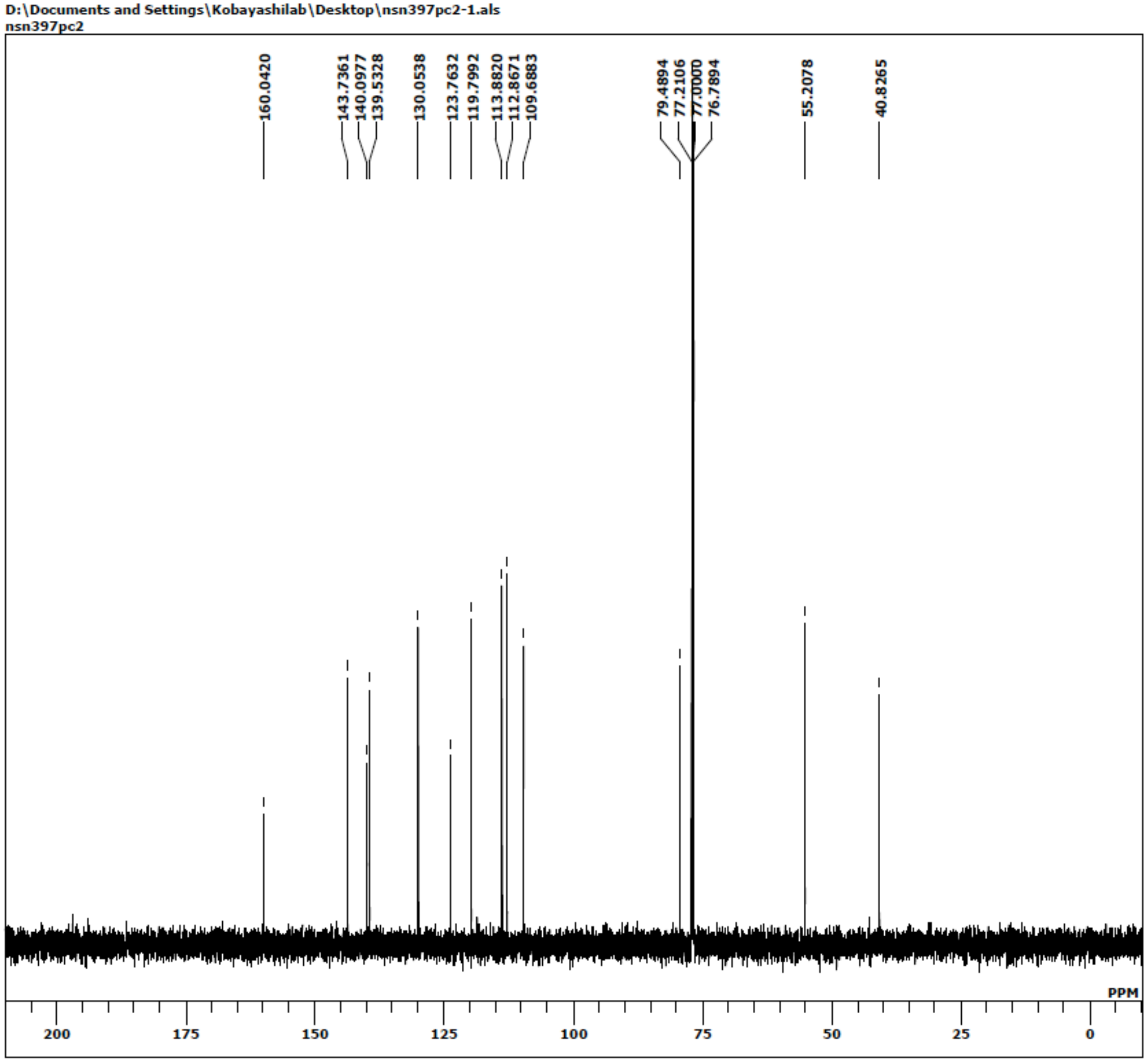
D:\Documents and Settings\Kobayashilab\Desktop\nsn397pc2-1.als
nsn397pc2
160.0420
143.7361
140.0977
139.5328
130.0538
123.7632
119.7992
113.8820
112.8671
109.6883
79.4894
77.2106
77.0000
76.7894
55.2078
40.8265
PPM
200
175
150
125
100
75
50
25
0

Sample Name : nsn400rac(for570)
OD-H, 1.0 mL/min, Hex:IPA = 3/2

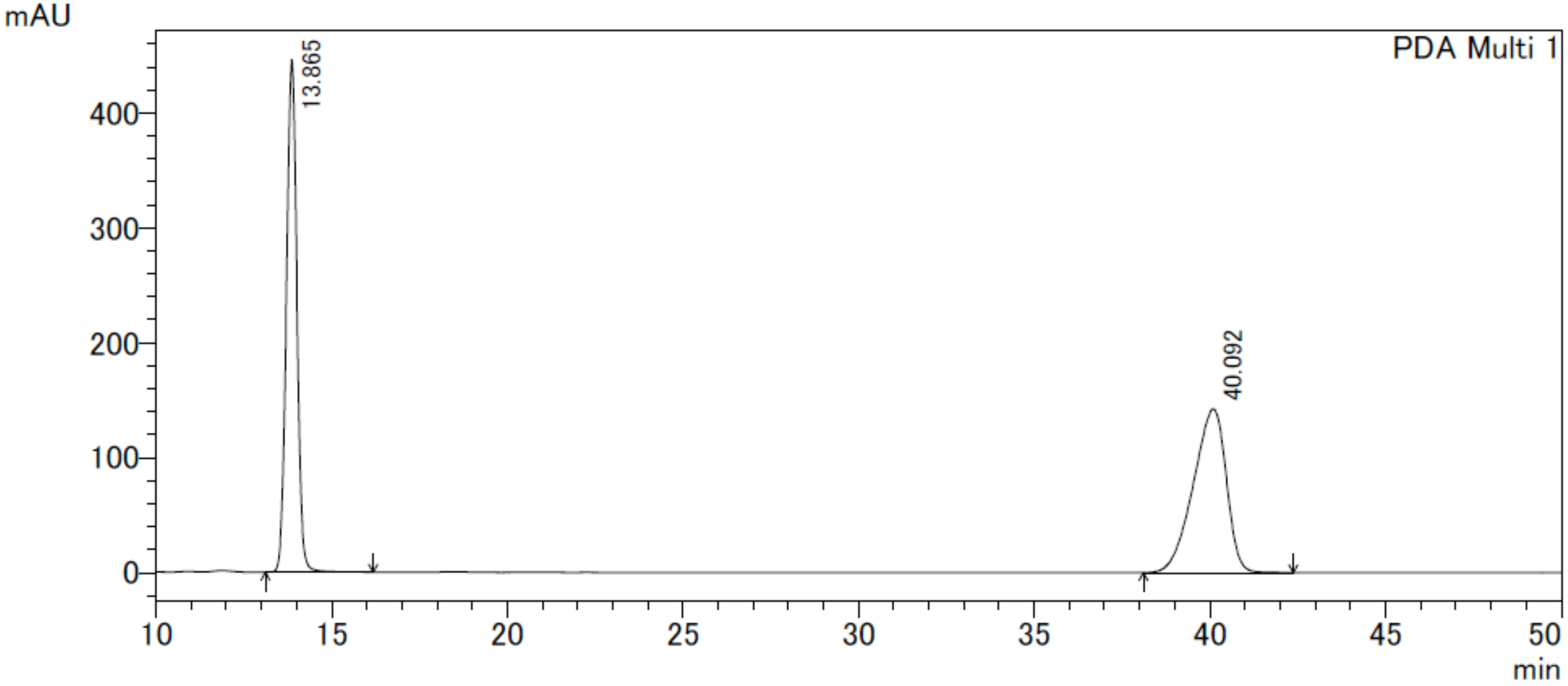

1 PDA Multi 1/220nm 4nm

PDA Ch1 220nm 4nm

Peak#	Ret. Time	Area	Height	Area%
1	13.865	9261027	446039	49.872
2	40.092	9308628	142390	50.128

3ha racemic

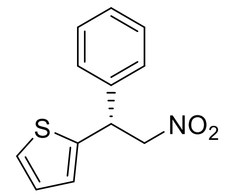

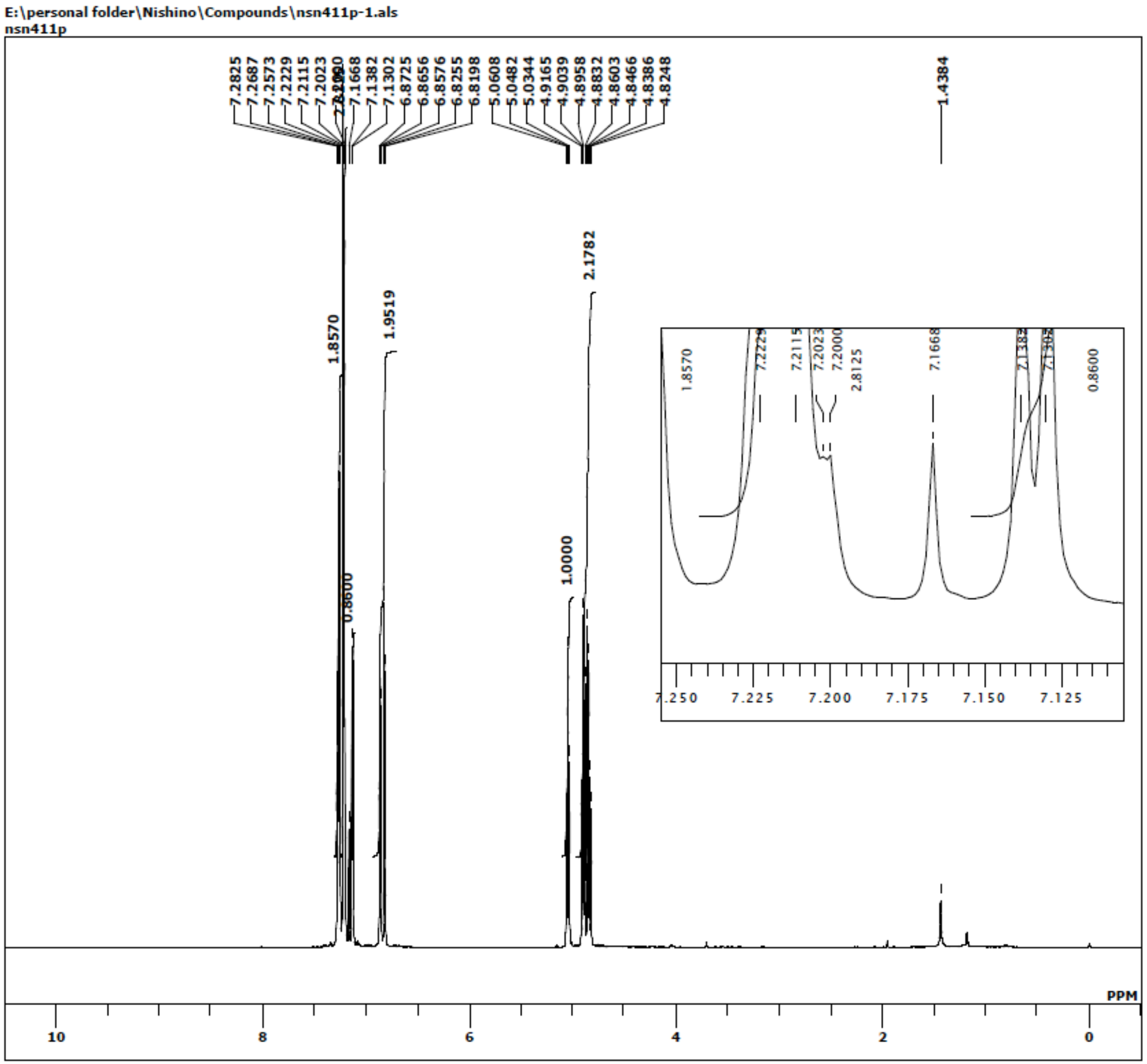

E:\personal folder\Nishino\Compounds\nsn411p-1.als
nsn411p
PPM

3ib 1H NMR

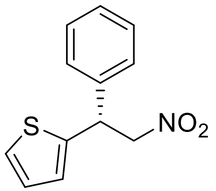

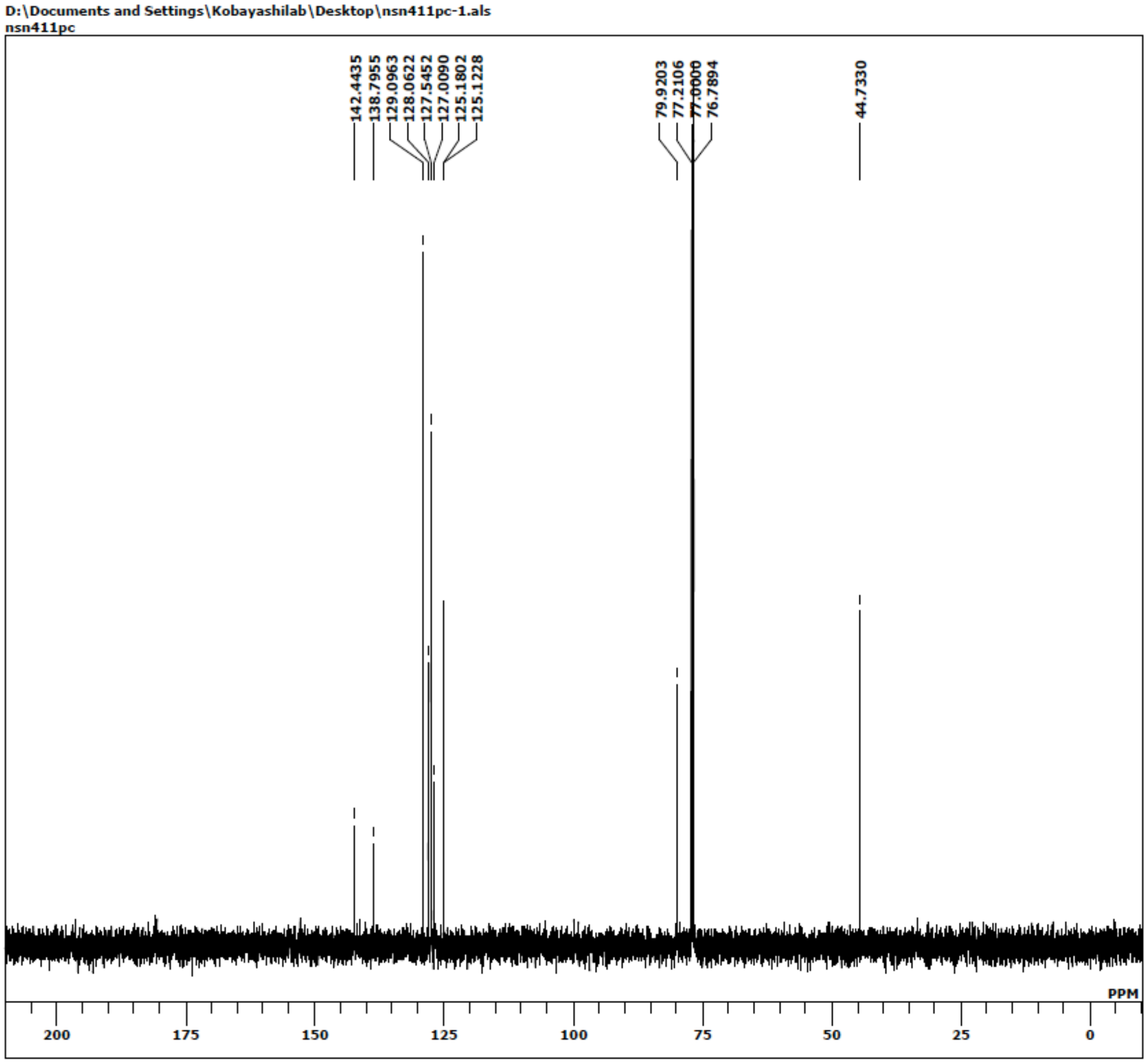
D:\Documents and Settings\Kobayashilab\Desktop\nsn411pc-1.als
nsn411pc
142.4435
138.7955
129.0963
128.0622
127.5452
127.0090
125.1802
125.1228
79.9203
77.2106
77.0000
76.7894
44.7330
PPM
200
175
150
125
100
75
50
25
0

3ib 13C NMR

Sample Name : nsn413rac(for571)
OD-H, 1.0 mL/min, Hex:IPA = 3/2

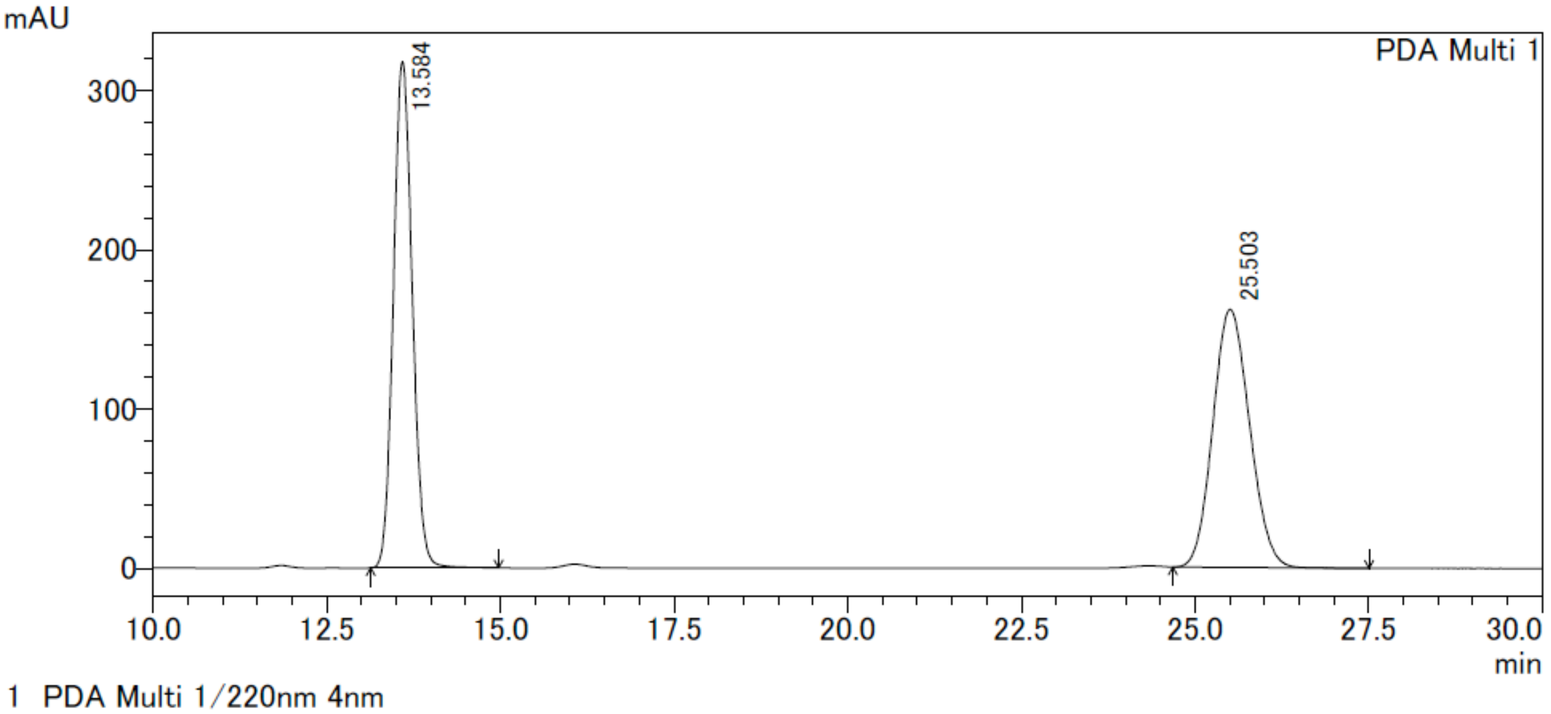

PDA Ch1 220nm 4nm

Peak#	Ret. Time	Area	Height	Area%
1	13.584	6011473	317783	50.214
2	25.503	5960319	162043	49.786

3ib racemic

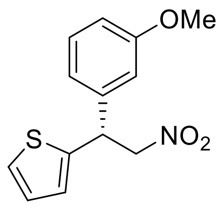

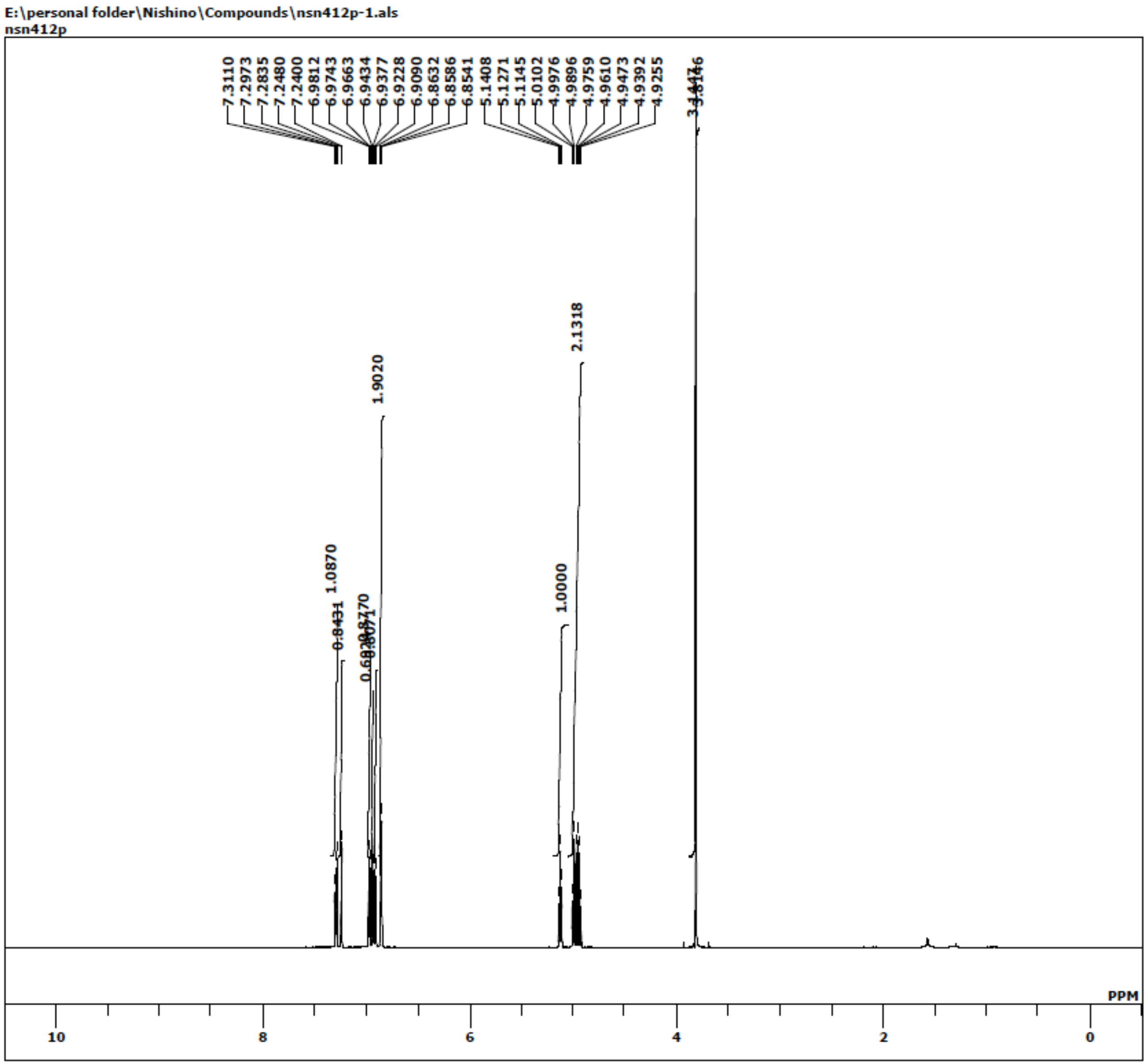
E:\personal folder\Nishino\Compounds\nsn412p-1.als
nsn412p
PPM

3ia 1H NMR

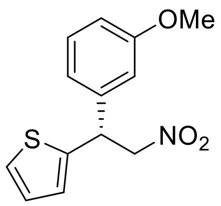
OMe
S
NO2

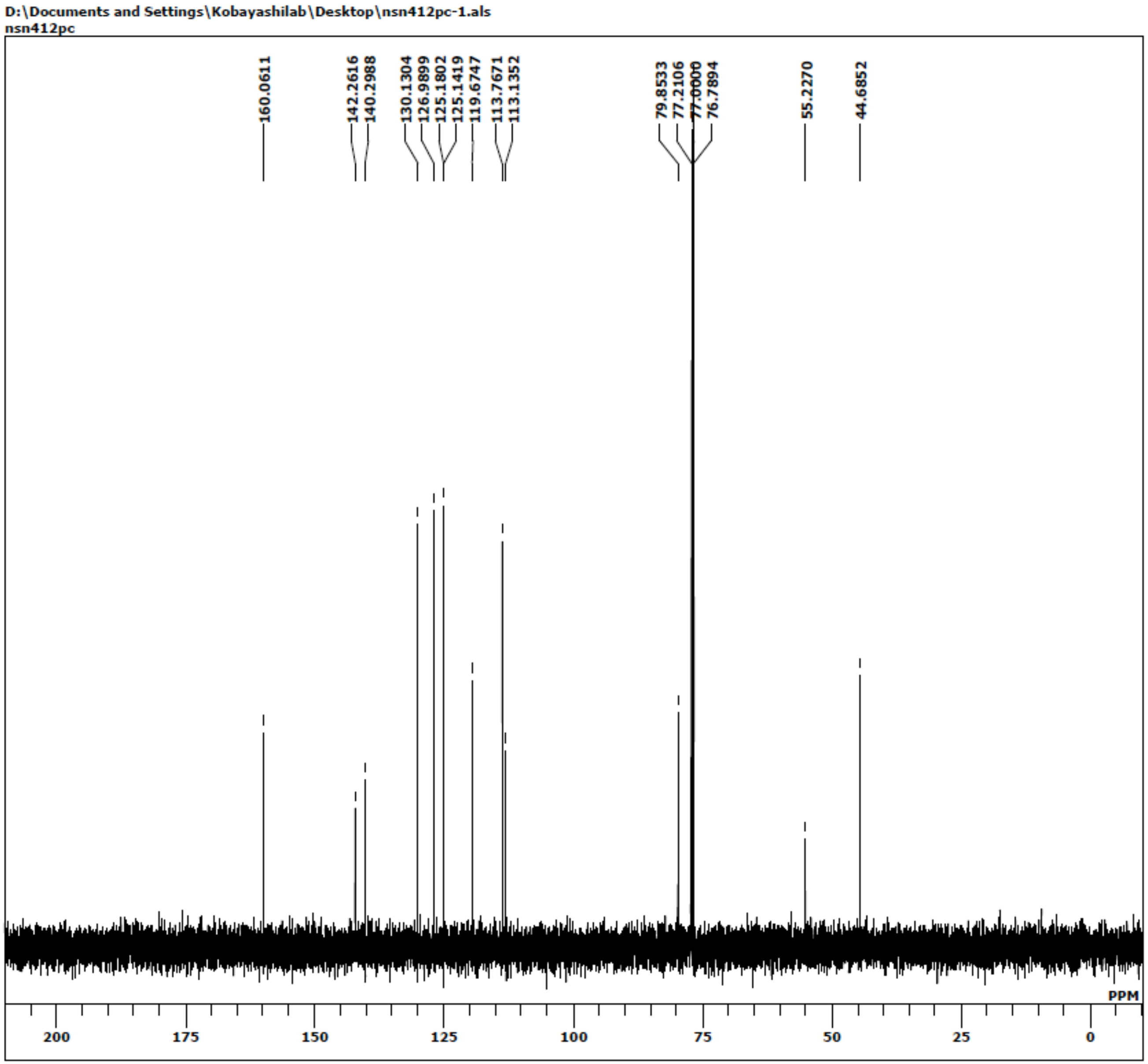
D:\Documents and Settings\Kobayashilab\Desktop\nsn412pc-1.als
nsn412pc
160.0611
142.2616
140.2988
130.1304
126.9899
125.1802
125.1419
119.6747
113.7671
113.1352
79.8533
77.2106
77.0000
76.7894
55.2270
44.6852
PPM
200
175
150
125
100
75
50
25
0

3ia 13C NMR

Sample Name : nsn414rac(for572)
OD-H, 1.0 mL/min, Hex:IPA = 3/2

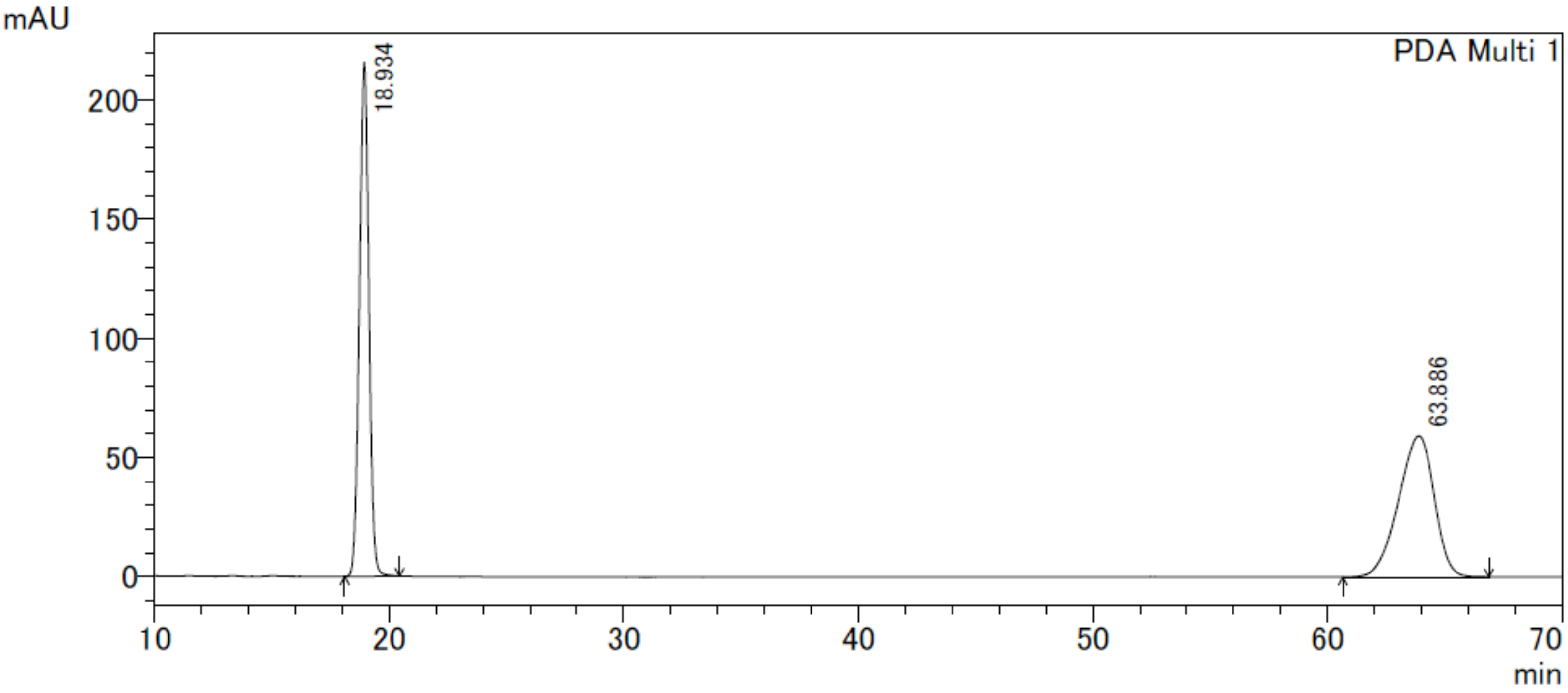

1 PDA Multi 1/220nm 4nm

PDA Ch1 220nm 4nm

Peak#	Ret. Time	Area	Height	Area%
1	18.934	6259445	215657	49.941
2	63.886	6274312	59180	50.059

3ia racemic

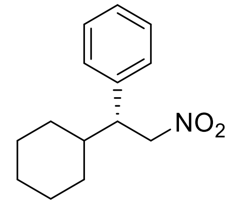

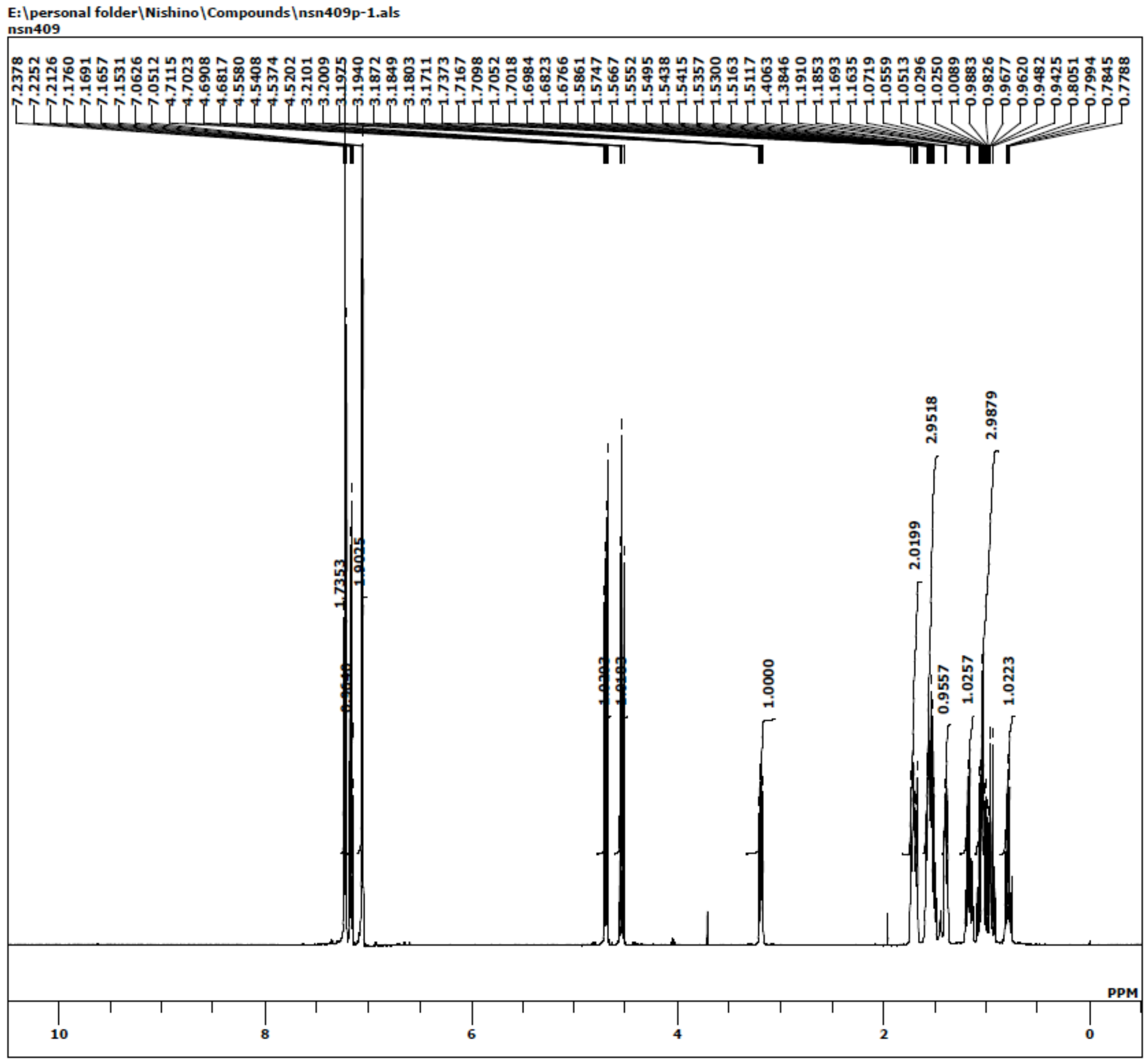

3jb 1H NMR

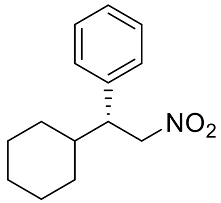

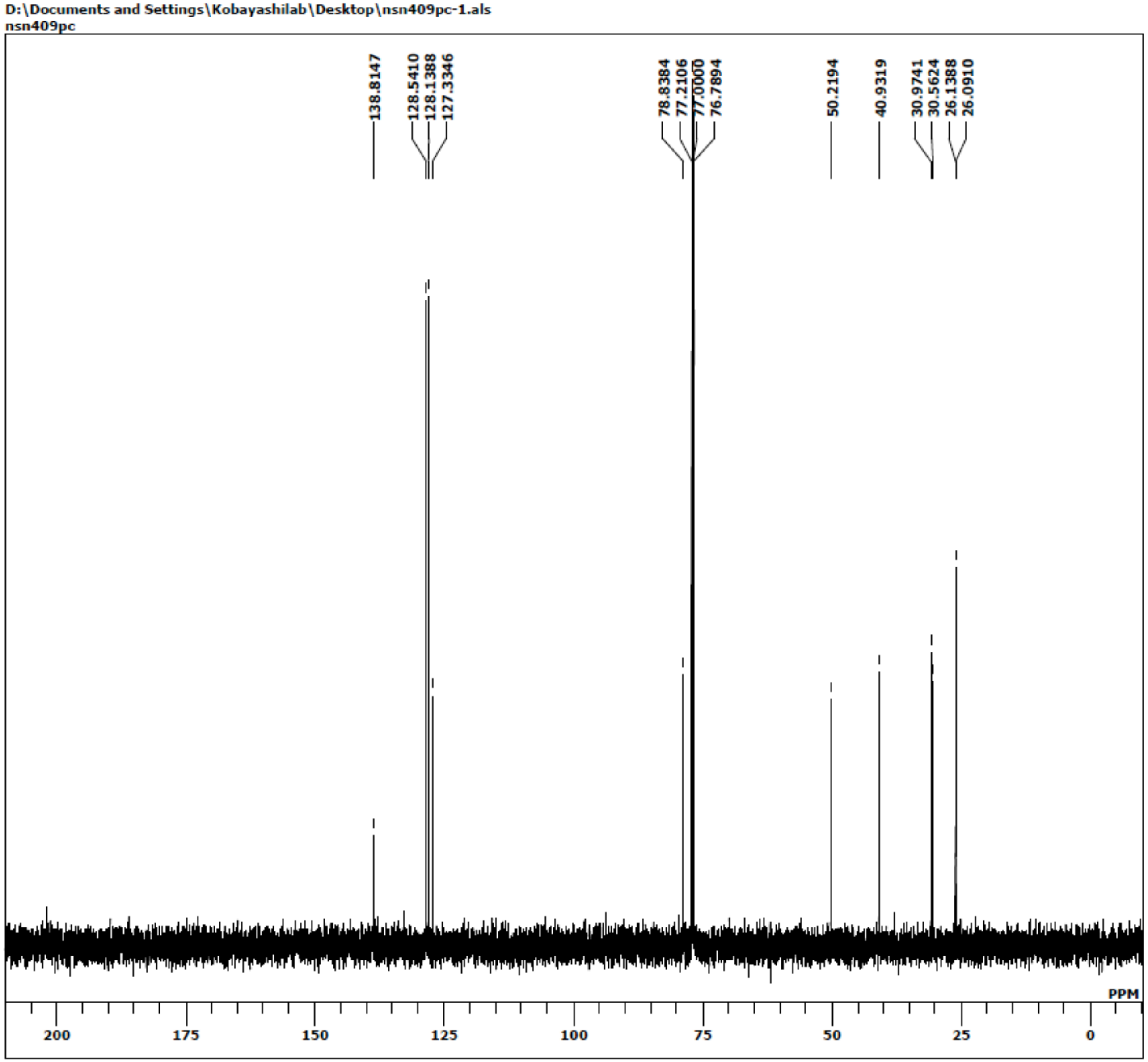
D:\Documents and Settings\Kobayashilab\Desktop\nsn409pc-1.als
nsn409pc
138.8147
128.5410
128.1388
127.3346
78.8384
77.2106
77.0000
76.7894
50.2194
40.9319
30.9741
30.5624
26.1388
26.0910
PPM
200
175
150
125
100
75
50
25
0

3jb 13C NMR

Sample Name : nsn415rac(for574)
OD-H, 1.0 mL/min, Hex:IPA = 3/2

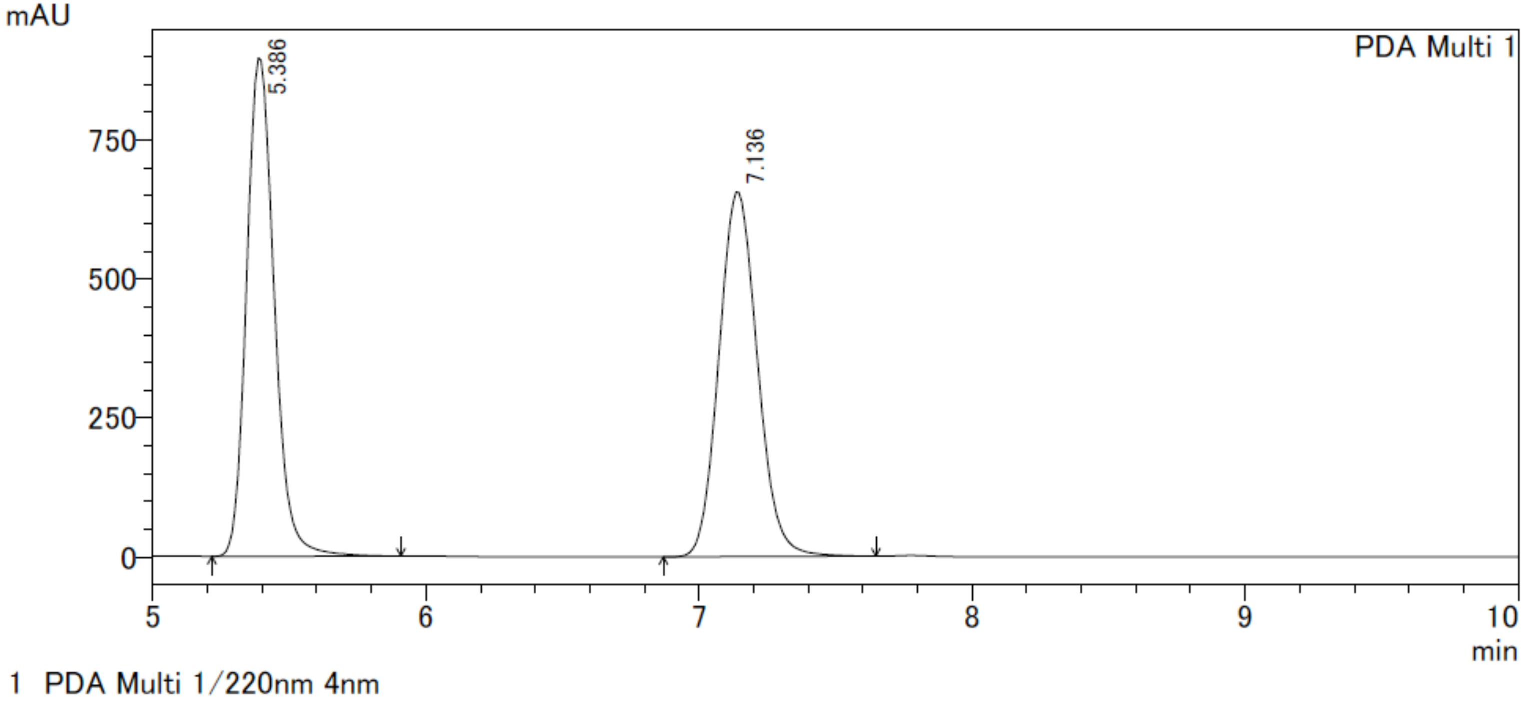

PDA Ch1 220nm 4nm

Peak#	Ret. Time	Area	Height	Area%
1	5.386	6314881	896462	49.517
2	7.136	6438185	655779	50.483

3jb racemic

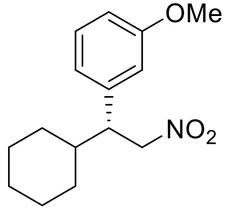
OMe
NO2

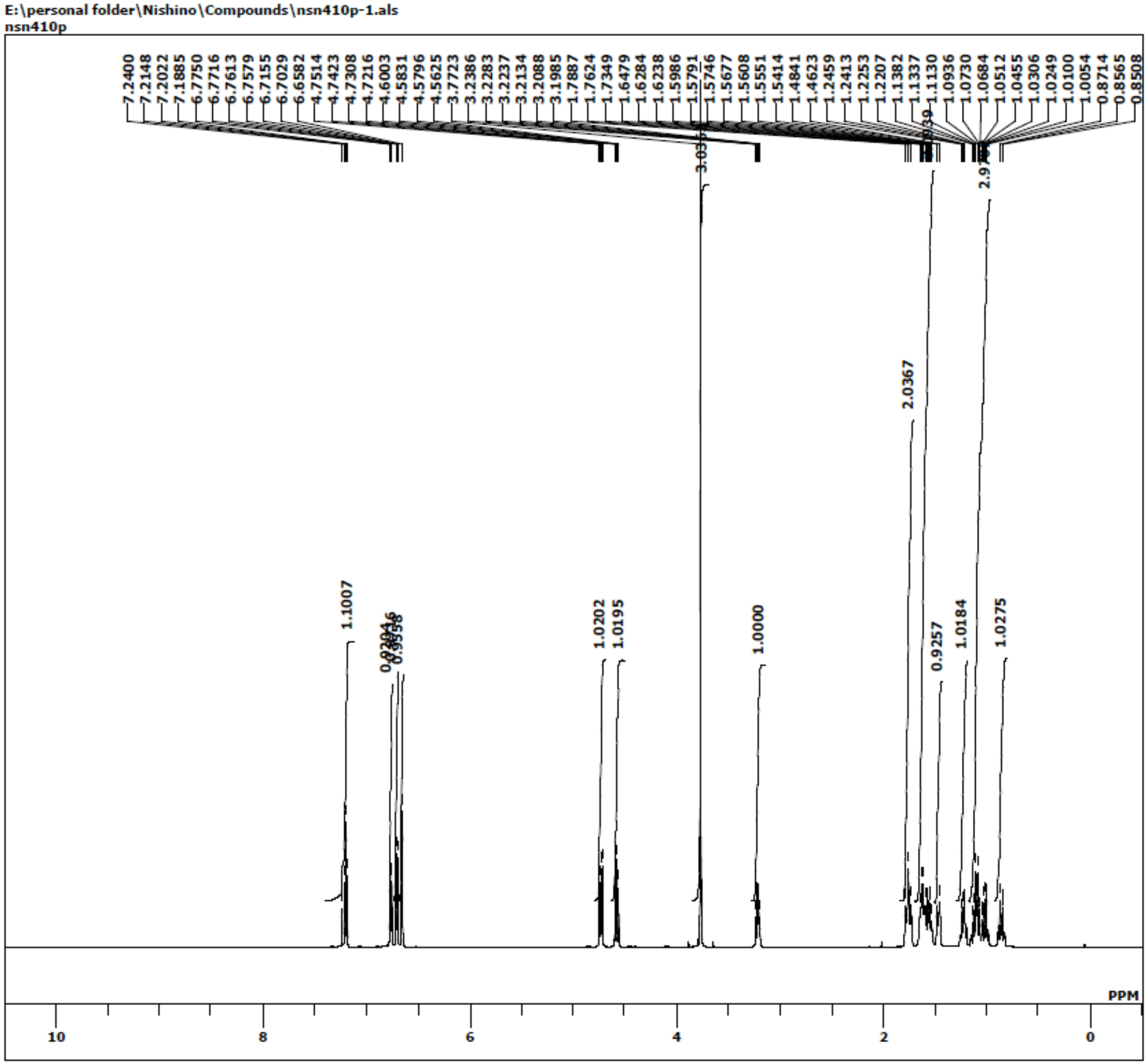
E:\personal folder\Nishino\Compounds\nsn410p-1.als
nsn410p
7.2400
7.2148
7.2022
7.1885
6.7750
6.7716
6.7613
6.7579
6.7155
6.7029
6.6582
4.7514
4.7423
4.7308
4.7216
4.6003
4.5831
4.5796
4.5625
3.7723
3.2386
3.2283
3.2237
3.2134
3.2088
3.1985
1.7887
1.7624
1.7349
1.6479
1.6284
1.6238
1.5986
1.5791
1.5746
1.5677
1.5608
1.5551
1.5414
1.4841
1.4623
1.2459
1.2413
1.2253
1.2207
1.1382
1.1337
1.1130
1.0936
1.0730
1.0684
1.0512
1.0455
1.0306
1.0249
1.0100
1.0054
0.8714
0.8565
0.8508
1.1007
1.0202
1.0195
1.0000
2.0367
0.9257
1.0184
1.0275
PPM
10
8
6
4
2
0

3ja 1H NMR

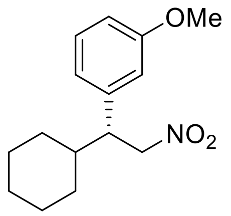
OMe
NO2

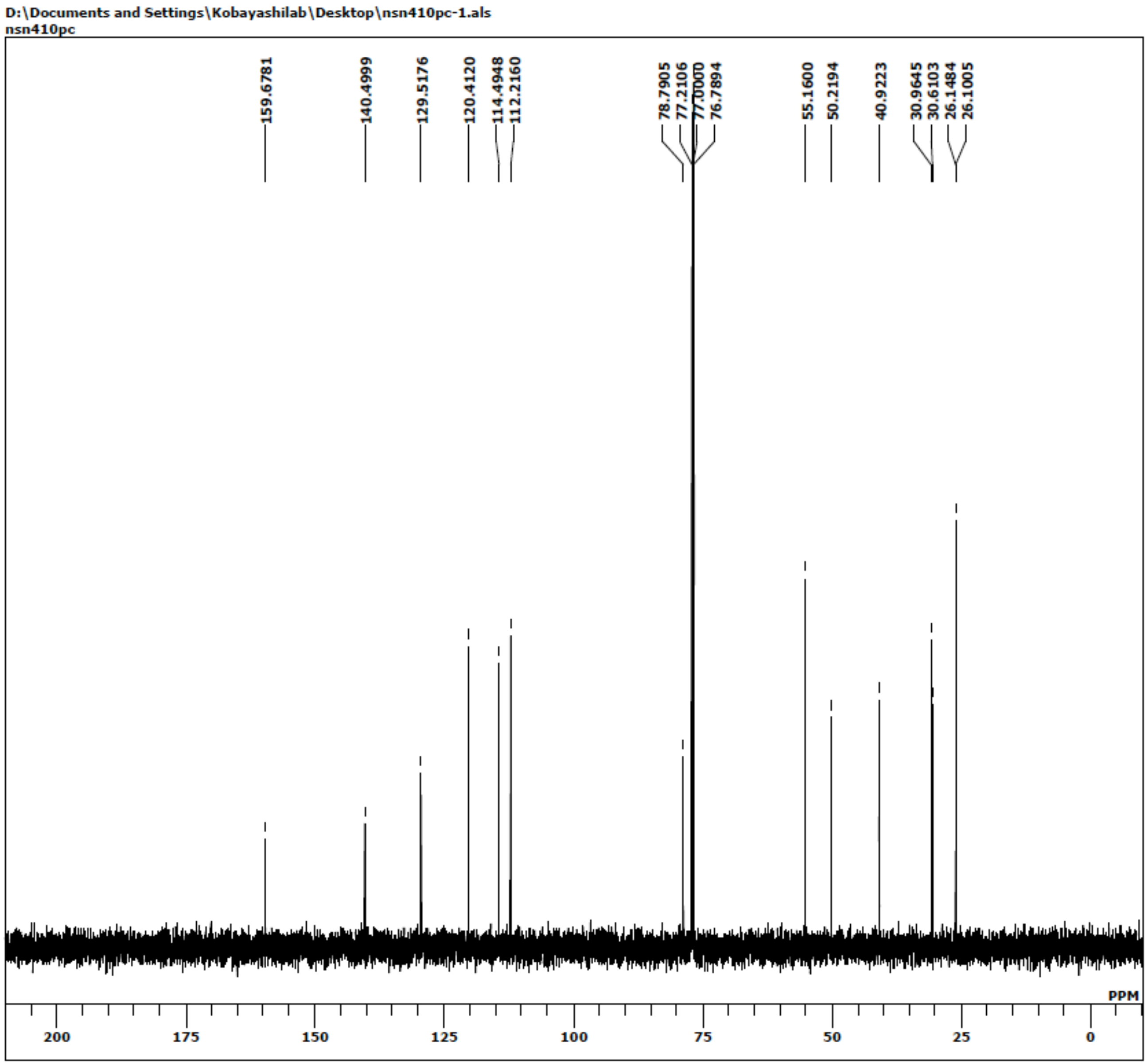
D:\Documents and Settings\Kobayashilab\Desktop\nsn410pc-1.als
nsn410pc
159.6781
140.4999
129.5176
120.4120
114.4948
112.2160
78.7905
77.2106
77.0000
76.7894
55.1600
50.2194
40.9223
30.9645
30.6103
26.1484
26.1005
PPM
200
175
150
125
100
75
50
25
0

3ja 13C NMR

Sample Name : nsn431rac(for575)
OD-H, 1.0 mL/min, Hex:IPA = 3/2

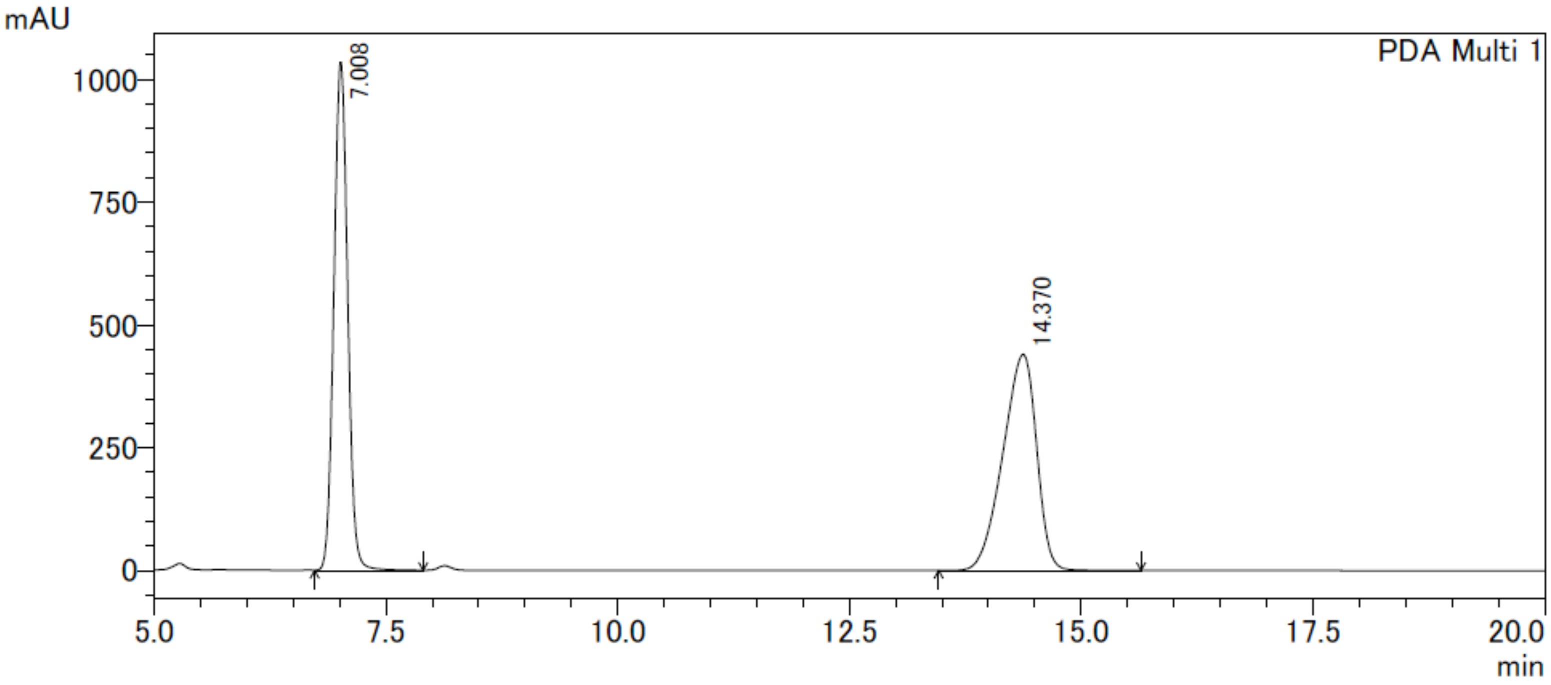

1 PDA Multi 1/220nm 4nm

PDA Ch1 220nm 4nm

Peak#	Ret. Time	Area	Height	Area%
1	7.008	10797847	1033510	49.223
2	14.370	11138806	439921	50.777

3ja racemic

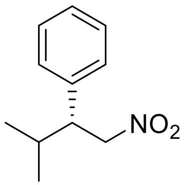
NO2

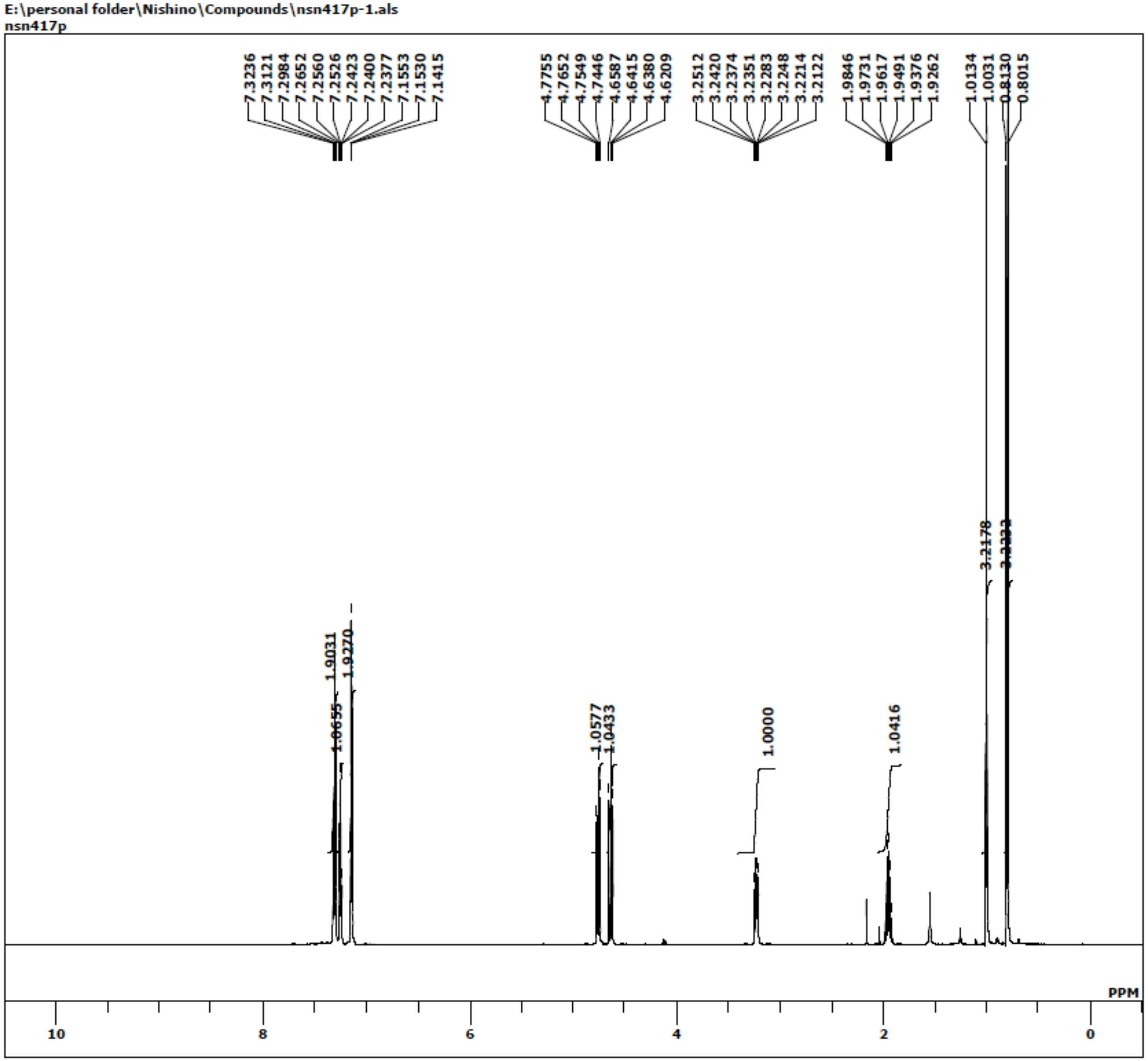
E:\personal folder\Nishino\Compounds\nsn417p-1.als
nsn417p
7.3236
7.3121
7.2984
7.2652
7.2560
7.2526
7.2423
7.2400
7.2377
7.1553
7.1530
7.1415
4.7755
4.7652
4.7549
4.7446
4.6587
4.6415
4.6380
4.6209
3.2512
3.2420
3.2374
3.2351
3.2283
3.2248
3.2214
3.2122
1.9846
1.9731
1.9617
1.9491
1.9376
1.9262
1.0134
1.0031
0.8130
0.8015
1.9031
1.0655
1.9270
1.0577
1.0433
1.0000
1.0416
3.2178
PPM
10
8
6
4
2
0

3kb 1H NMR

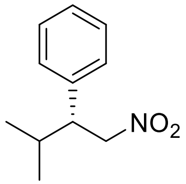
NO2

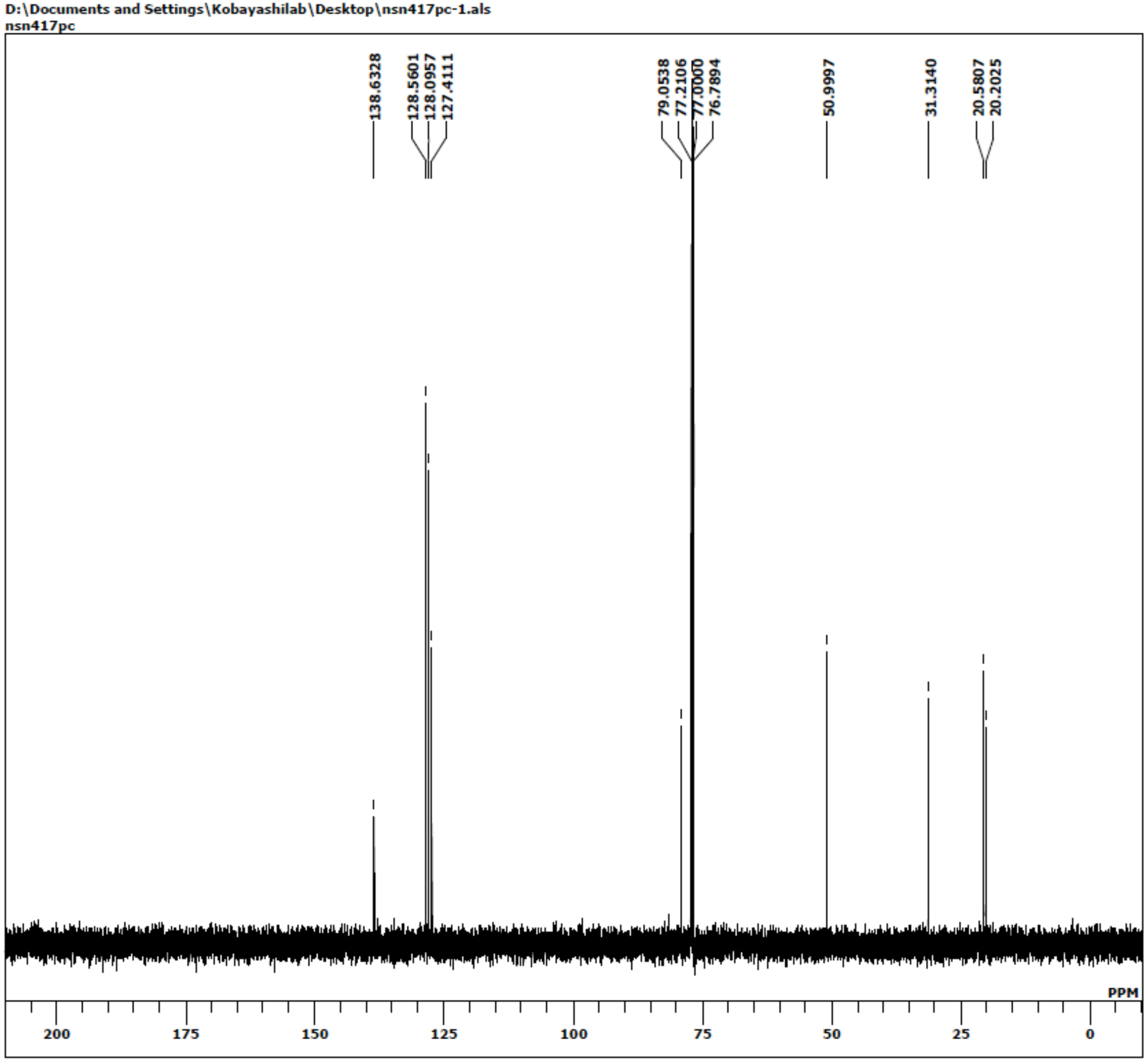
D:\Documents and Settings\Kobayashilab\Desktop\nsn417pc-1.als
nsn417pc
138.6328
128.5601
128.0957
127.4111
79.0538
77.2106
77.0000
76.7894
50.9997
31.3140
20.5807
20.2025
PPM
200
175
150
125
100
75
50
25
0

3kb 13C NMR

Sample Name : nsn419rac(for576)
OD-H, 1.0 mL/min, Hex:IPA = 3/2

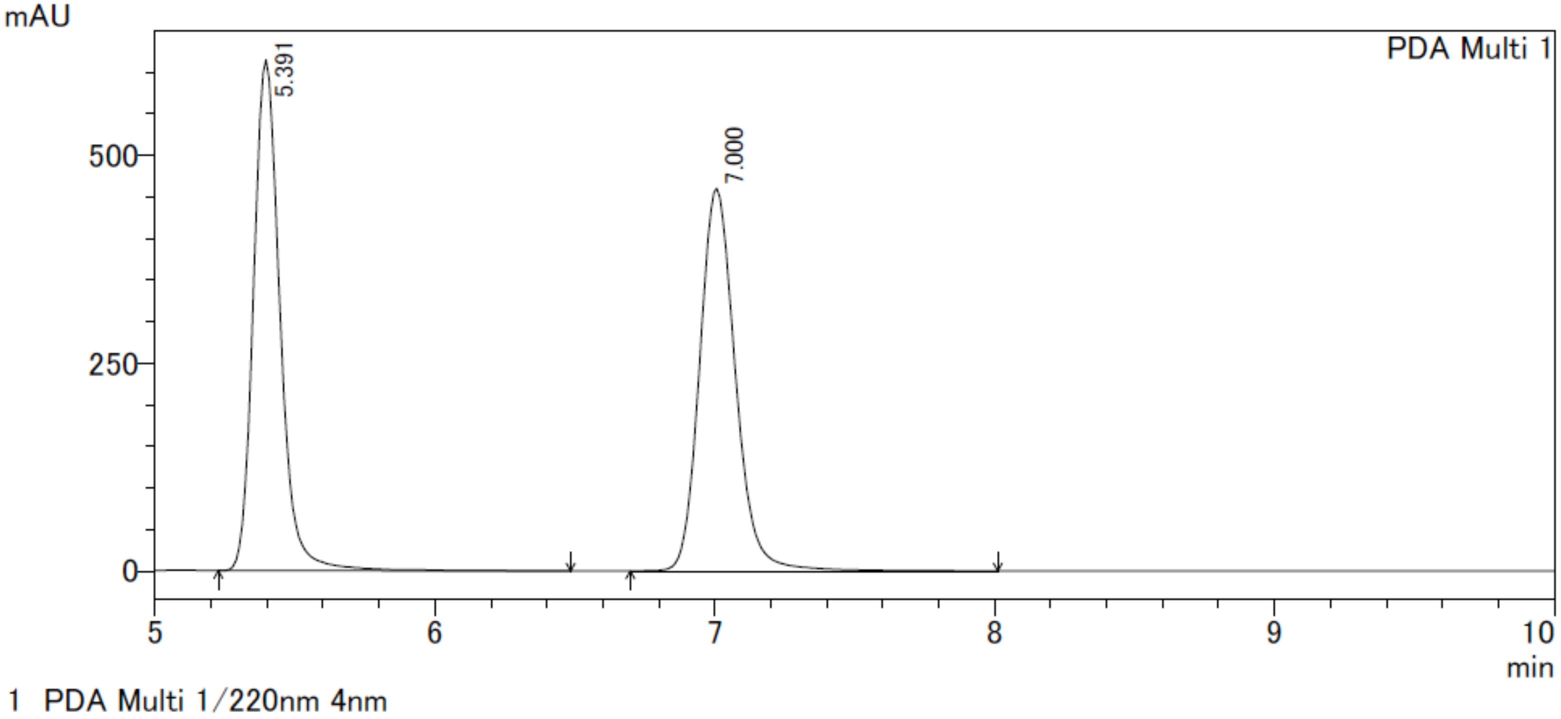

PDA Ch1 220nm 4nm

Peak#	Ret. Time	Area	Height	Area%
1	5.391	4048959	614476	49.760
2	7.000	4088006	460129	50.240

3kb racemic

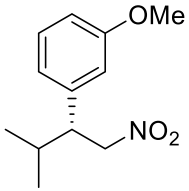
OMe
NO2

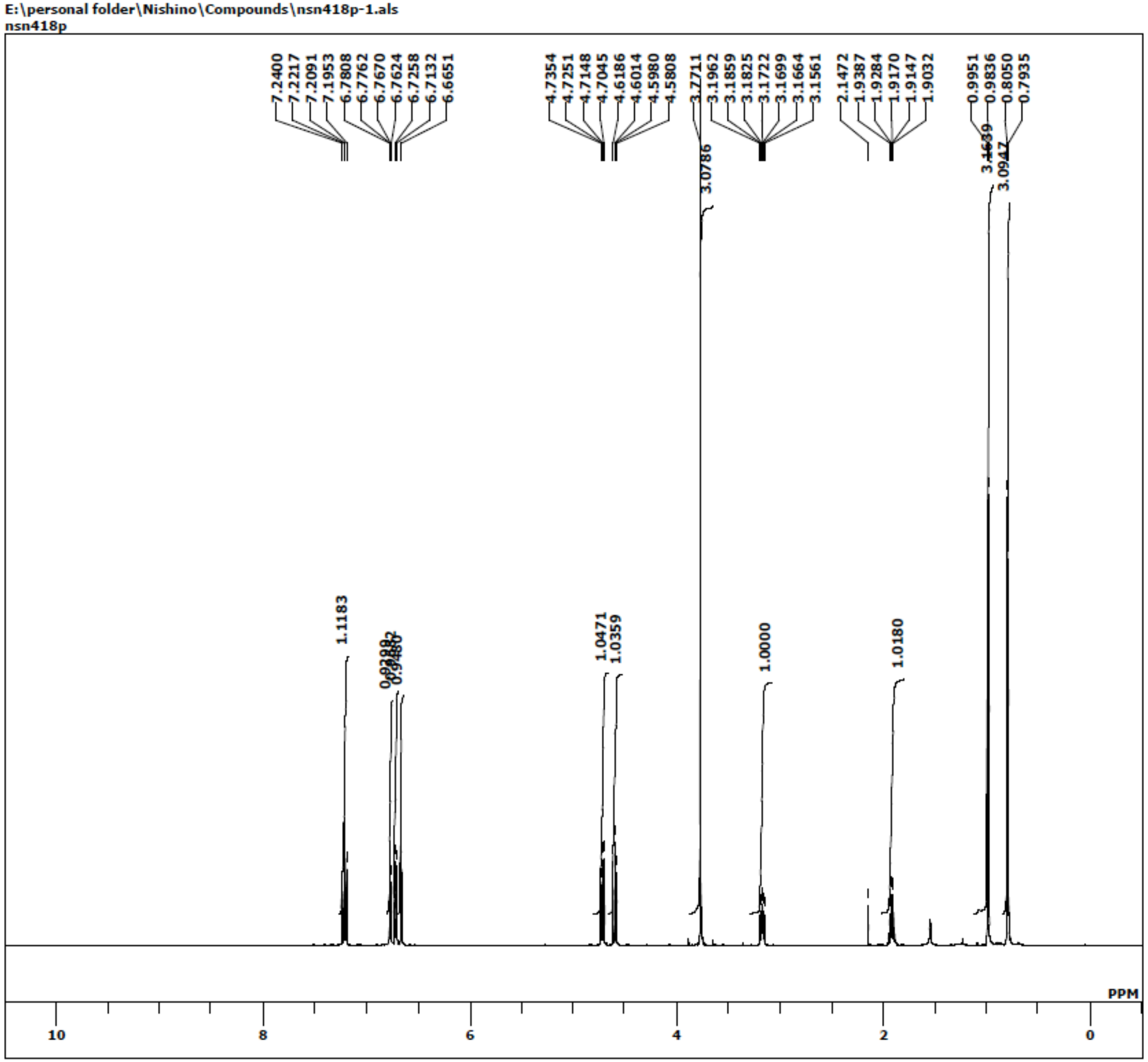
E:\personal folder\Nishino\Compounds\nsn418p-1.als
nsn418p
7.2400
7.2217
7.2091
7.1953
6.7808
6.7762
6.7670
6.7624
6.7258
6.7132
6.6651
4.7354
4.7251
4.7148
4.7045
4.6186
4.6014
4.5980
4.5808
3.7711
3.1962
3.1859
3.1825
3.1722
3.1699
3.1664
3.1561
2.1472
1.9387
1.9284
1.9170
1.9147
1.9032
0.9951
0.9836
0.8050
0.7935
1.1183
1.0471
1.0359
3.0786
1.0000
1.0180
PPM
10
8
6
4
2
0

3ka 1H NMR

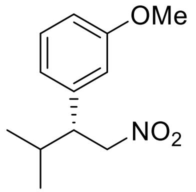

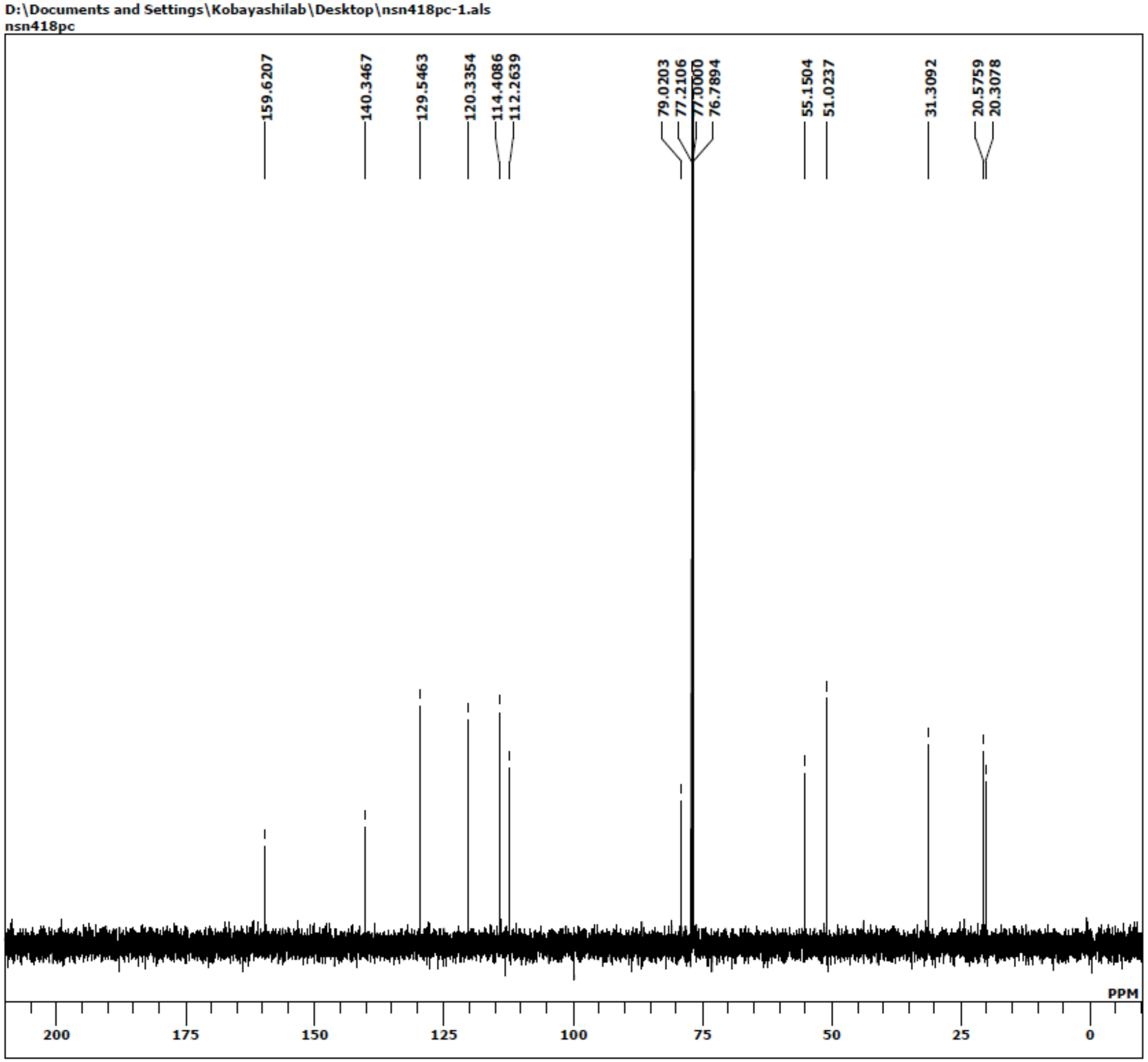

D:\Documents and Settings\Kobayashilab\Desktop\nsn418pc-1.als
nsn418pc
159.6207
140.3467
129.5463
120.3354
114.4086
112.2639
79.0203
77.2106
77.0000
76.7894
55.1504
51.0237
31.3092
20.5759
20.3078
PPM
200
175
150
125
100
75
50
25
0

3ka 13C NMR

Sample Name : nsn420rac(for577)
OD-H, 1.0 mL/min, Hex:IPA = 3/2

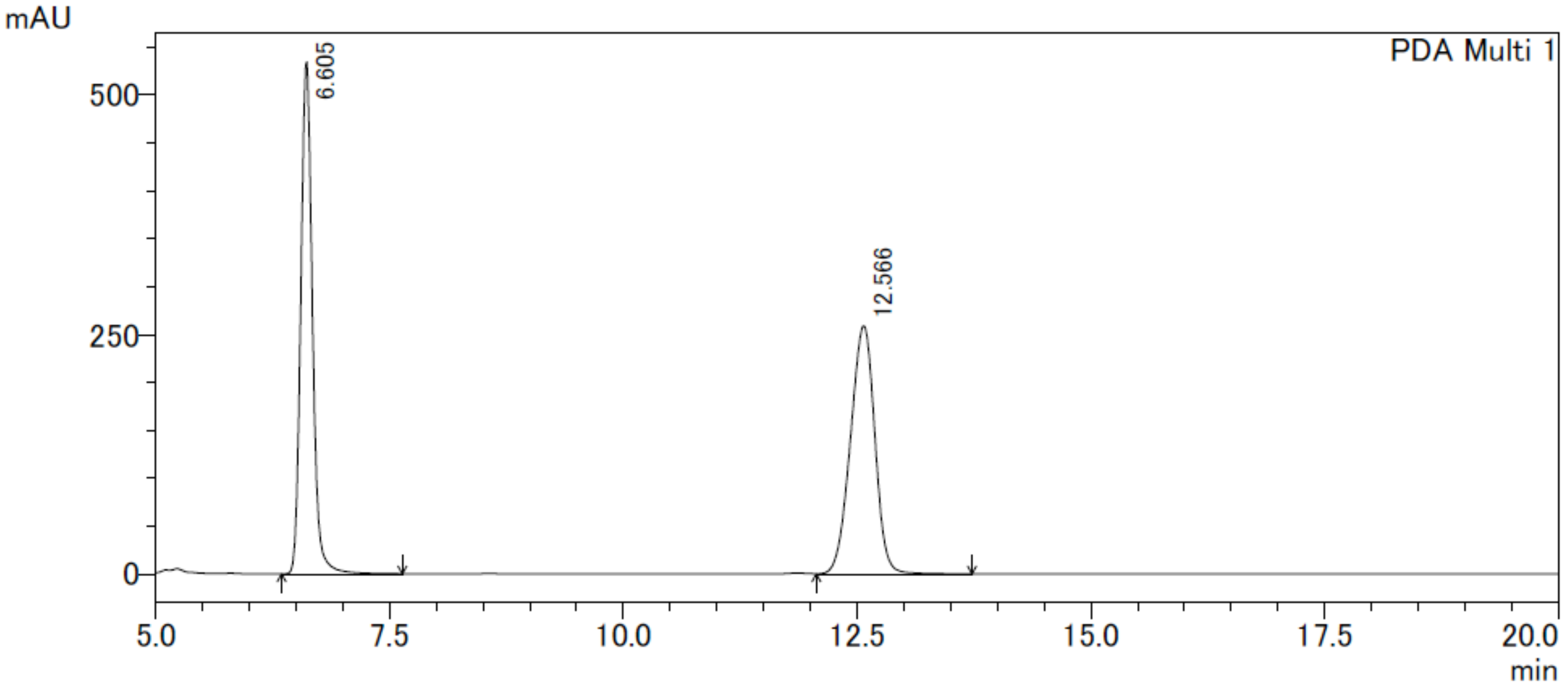

1 PDA Multi 1/220nm 4nm

PDA Ch1 220nm 4nm

Peak#	Ret. Time	Area	Height	Area%
1	6.605	4676319	534198	49.932
2	12.566	4689130	258808	50.068

3ka racemic

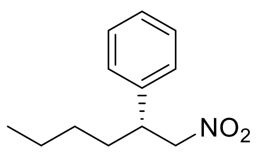

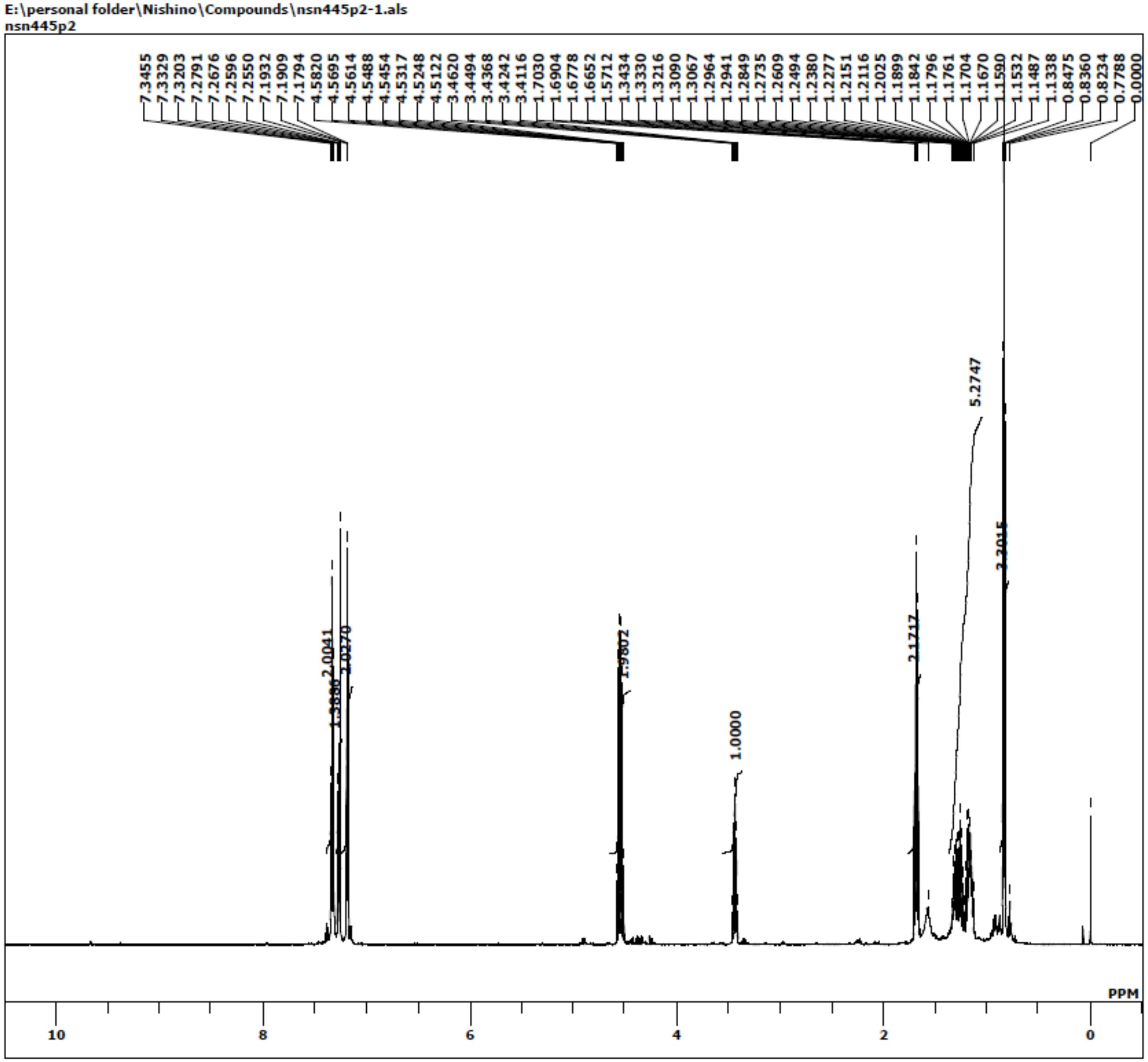
E:\personal folder\Nishino\Compounds\nsn445p2-1.als
nsn445p2
PPM

3lb 1H NMR

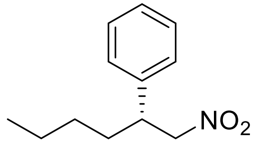

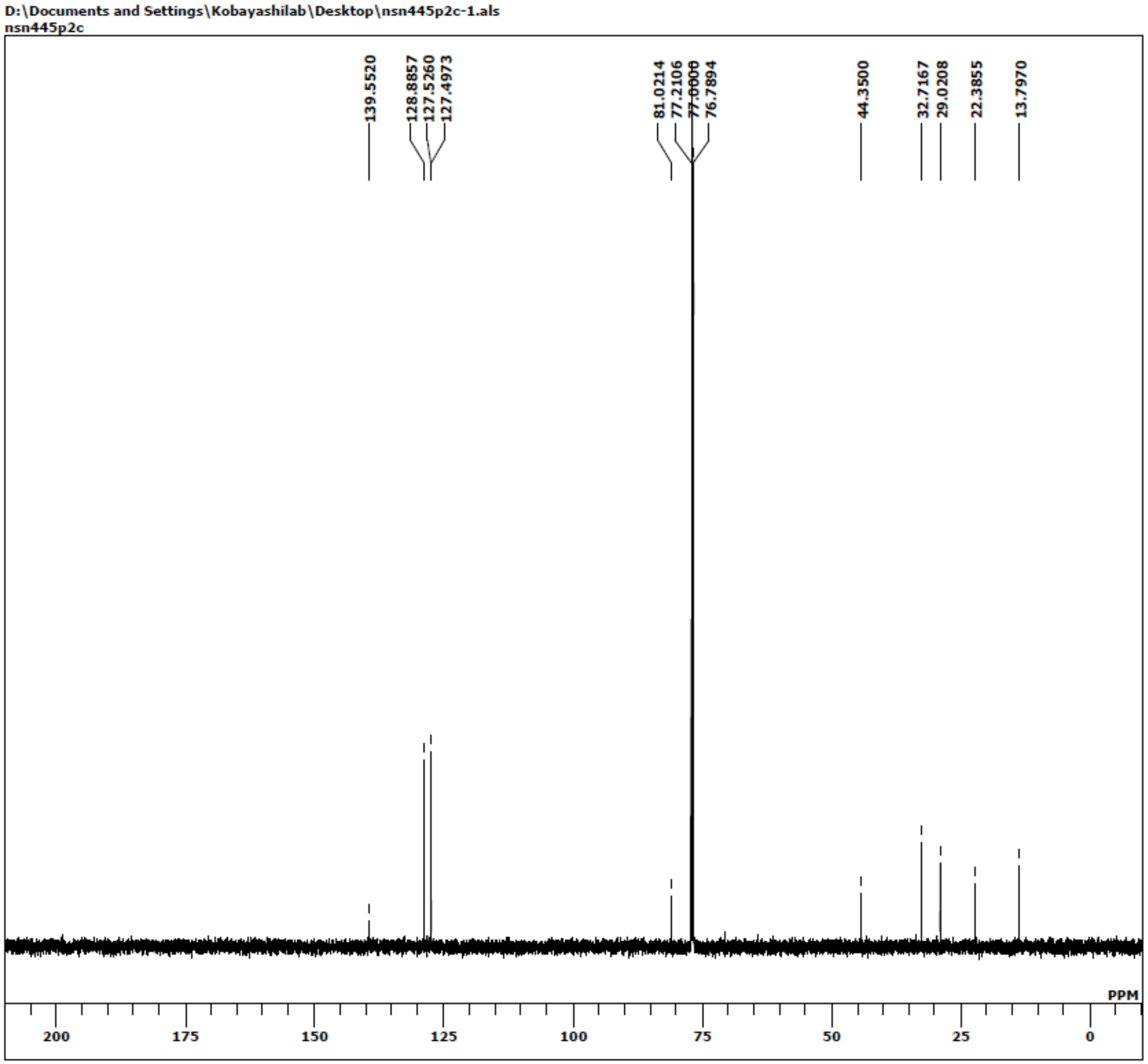
D:\Documents and Settings\Kobayashilab\Desktop\nsn445p2c-1.als
nsn445p2c
139.5520
128.8857
127.5260
127.4973
81.0214
77.2106
77.0000
76.7894
44.3500
32.7167
29.0208
22.3855
13.7970
PPM
200
175
150
125
100
75
50
25
0

3lb 13C NMR

Sample Name : nsn445rac(for578)
OD-H, 1.0 mL/min, Hex:IPA = 99/1

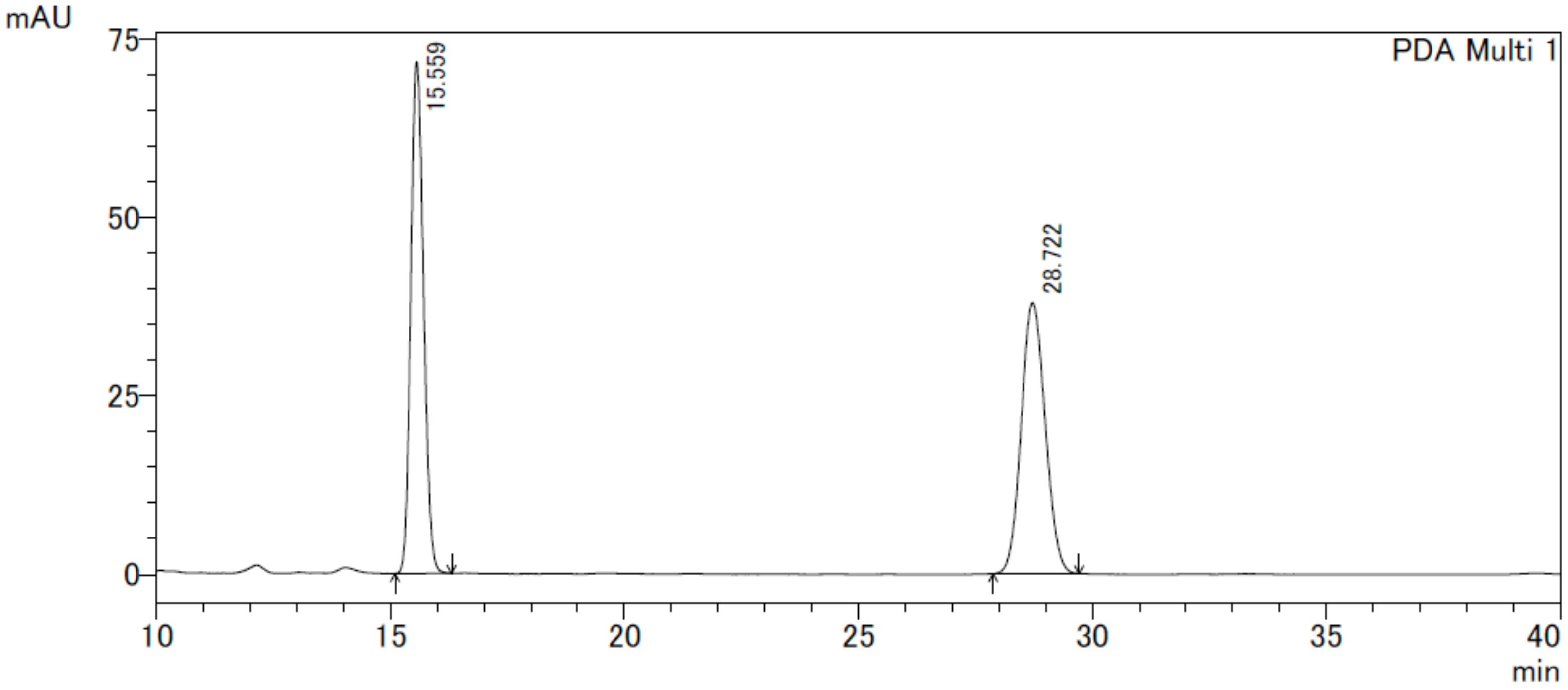

1 PDA Multi 1/220nm 4nm

PDA Ch1 220nm 4nm

Peak#	Ret. Time	Area	Height	Area%
1	15.559	1358783	71611	50.041
2	28.722	1356550	37948	49.959

3lb racemic

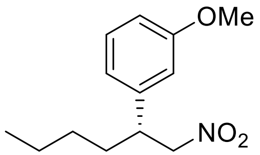
OMe
NO2

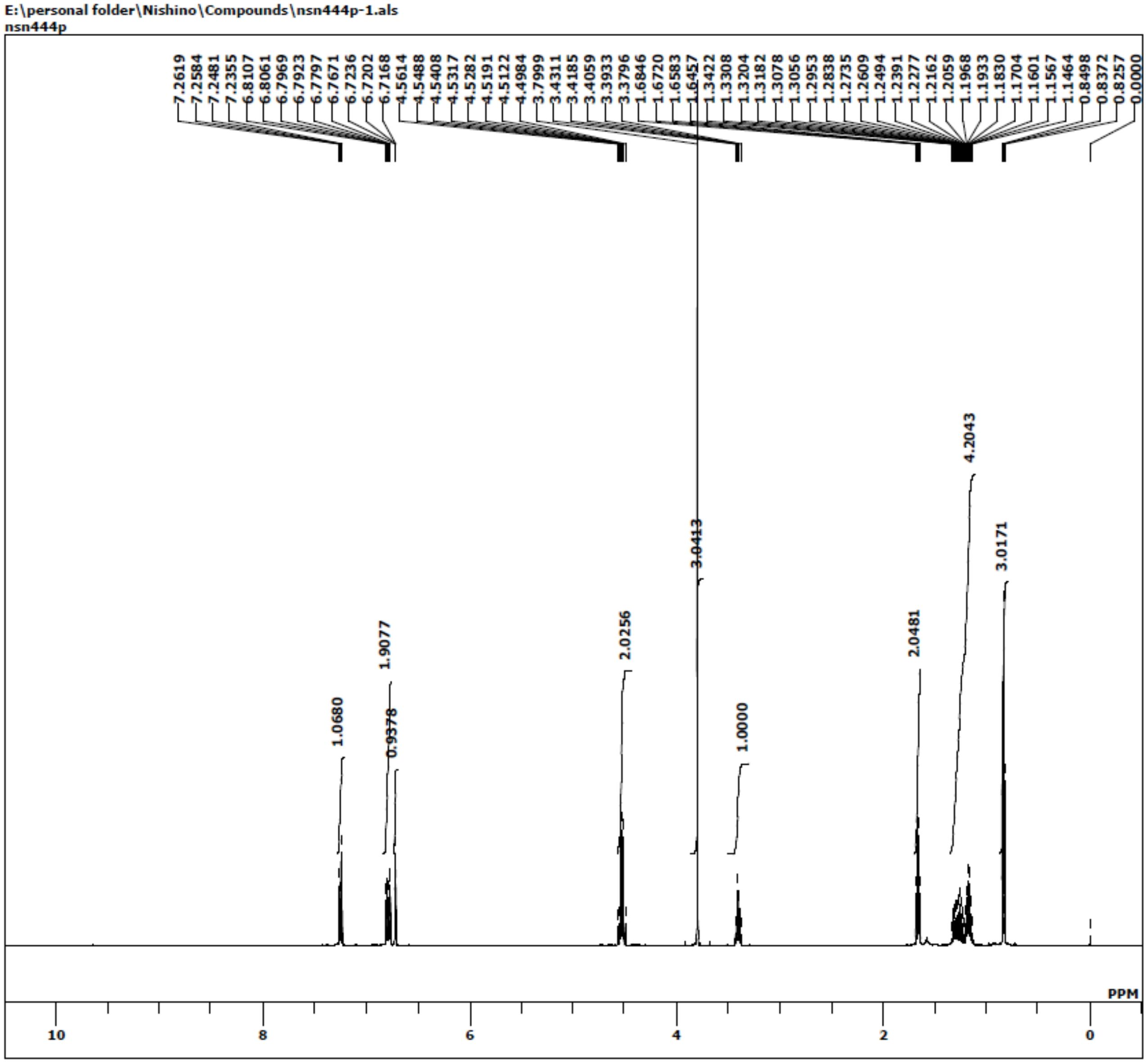
E:\personal folder\Nishino\Compounds\nsn444p-1.als
nsn444p
7.2619
7.2584
7.2481
7.2355
6.8107
6.8061
6.7969
6.7923
6.7797
6.7671
6.7236
6.7202
6.7168
4.5614
4.5488
4.5408
4.5317
4.5282
4.5191
4.5122
4.4984
3.7999
3.4311
3.4185
3.4059
3.3933
3.3796
1.6846
1.6720
1.6583
1.6457
1.3422
1.3308
1.3204
1.3182
1.3078
1.3056
1.2953
1.2838
1.2735
1.2609
1.2494
1.2391
1.2277
1.2162
1.2059
1.1968
1.1933
1.1830
1.1704
1.1601
1.1567
1.1464
0.8498
0.8372
0.8257
0.0000
1.0680
1.9077
0.9378
2.0256
3.0413
1.0000
2.0481
4.2043
3.0171
PPM
10
8
6
4
2
0

3la 1H NMR

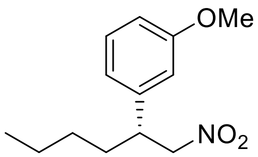

OMe
NO2

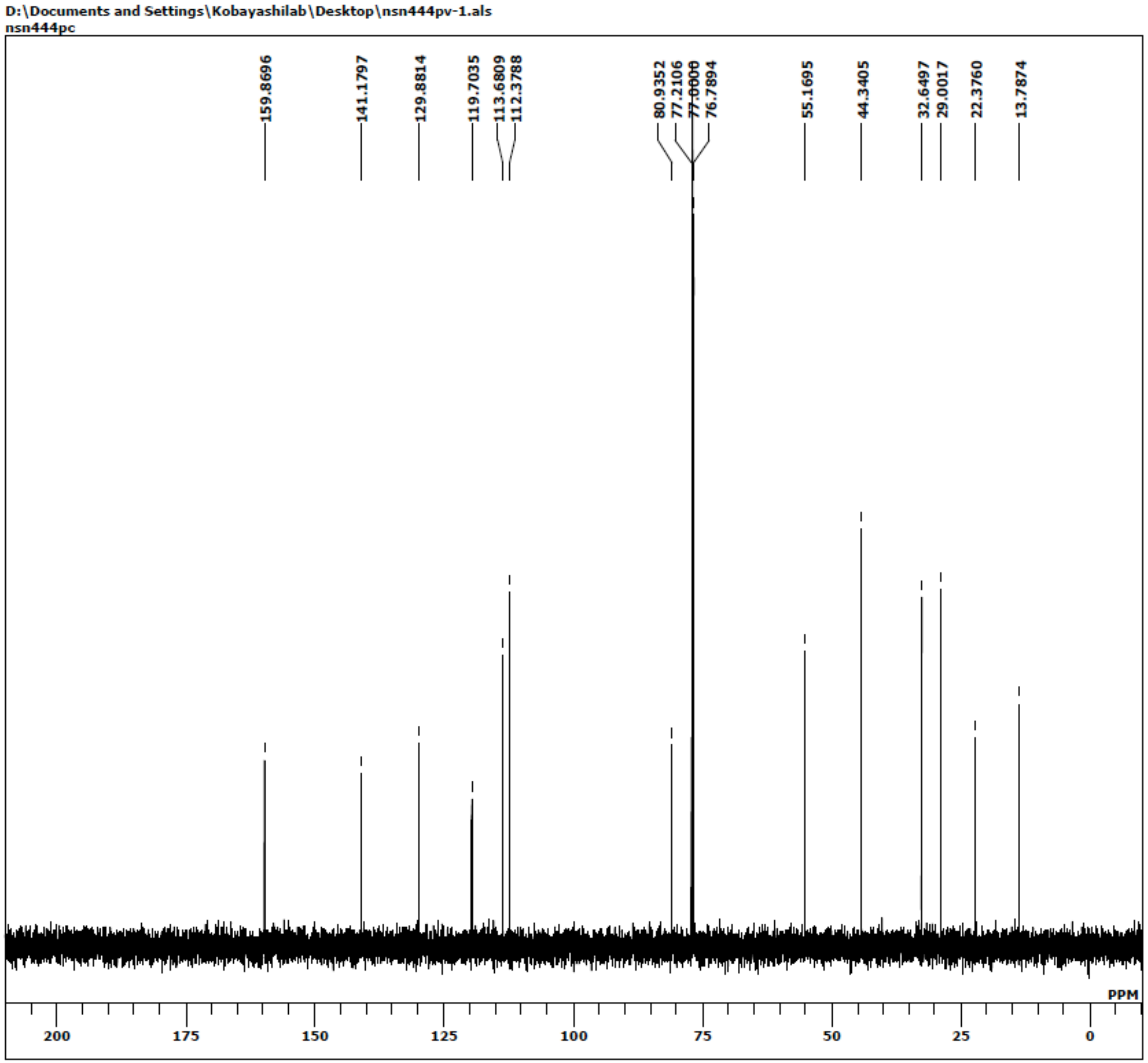

D:\Documents and Settings\Kobayashilab\Desktop\nsn444pv-1.als
nsn444pc
159.8696
141.1797
129.8814
119.7035
113.6809
112.3788
80.9352
77.2106
77.0000
76.7894
55.1695
44.3405
32.6497
29.0017
22.3760
13.7874
PPM
200
175
150
125
100
75
50
25
0

3la 13C NMR

Sample Name : nsn446rac(for579)
OD-H, 1.0 mL/min, Hex:IPA = 99/1

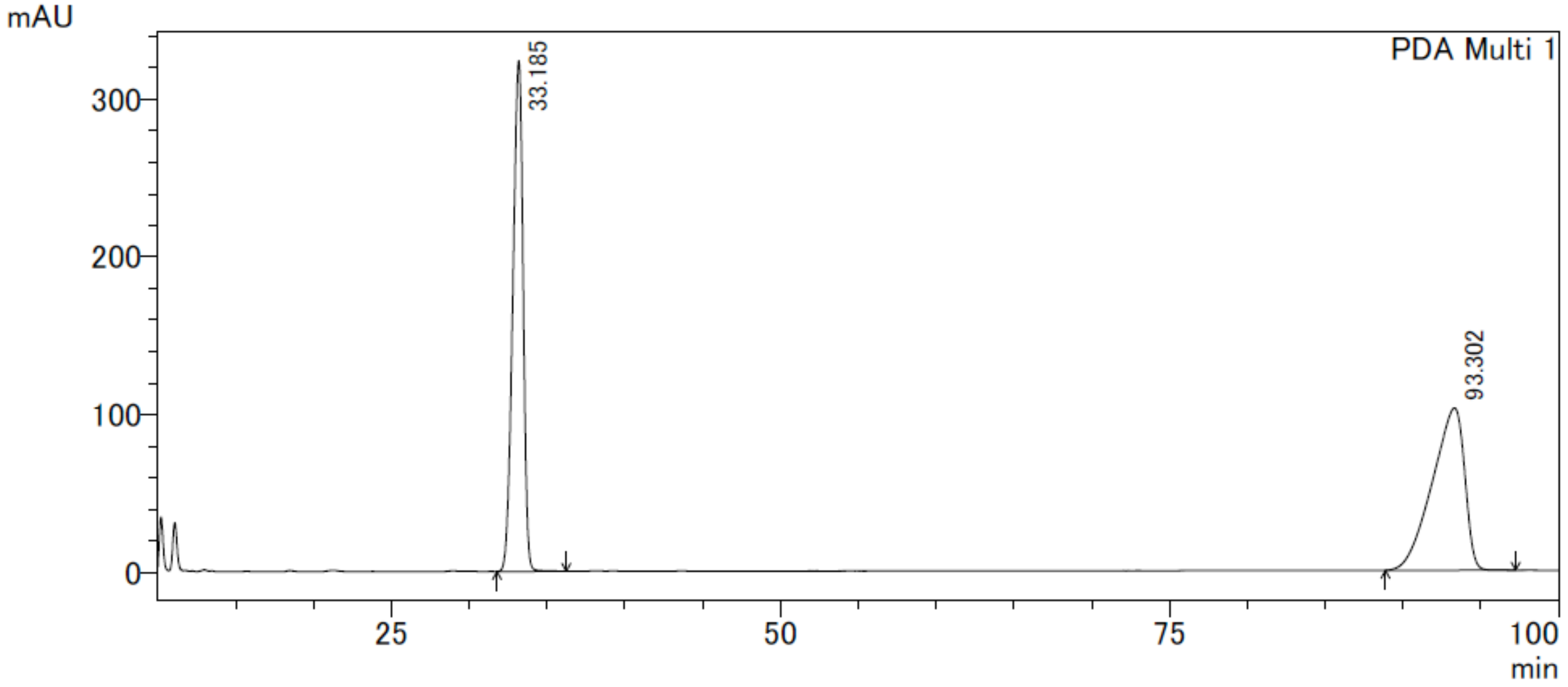

1 PDA Multi 1/220nm 4nm

PDA Ch1 220nm 4nm

Peak#	Ret. Time	Area	Height	Area%
1	33.185	14860401	323933	49.865
2	93.302	14940705	102968	50.135

3la racemic

HPLC Table 3 entry 1

==== Shimadzu LCsolution 分析レポート ====

D:¥Nishino¥nsn586.lcd

分析者 : System Administrator
サンプル名 : nsn586
サンプルID : nsn586
測定条件 :OD-H, 1.0 mL/min, Hex:IPA = 1/1
注入量 : 1 uL
データファイル : nsn586.lcd
メソッドファイル : product.lcm

レポートファイル : Default.lcr
分析日時 : 2015/01/26 14:44:28
解析日時 : 2015/01/26 18:34:51

<クロマトグラム>

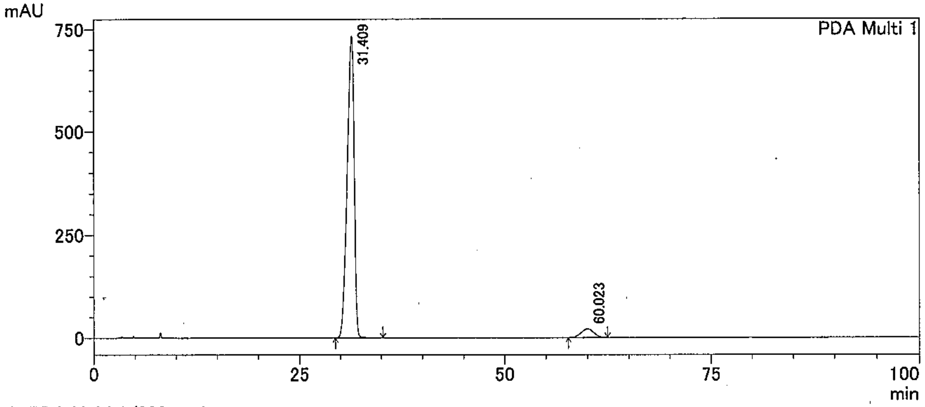

1 PDA Multi 1/220nm 4nm

ﾋﾟｰｸﾃｰﾌﾞﾙ D:¥Nishino¥nsn586.lcd

PDA Ch1 220nm 4nm

ピーク#	保持時間	面積	高さ	面積%	高さ%
1	31.409	43637262	733400	95.042	97.189
2	60.023	2276208	21213	4.958	2.811
Total		45913470	754613	100.000	100.000

ピーク : peak 保持時間:retention time 面積:area 高さ:height

HPLC Table 3 entry 2

==== Shimadzu LCsolution 分析レポート ====

D:¥Nishino¥nsn588.lcd

分析者	: System Administrator
サンプル名	: nsn588
サンプルID	: nsn588
測定条件	:OD-H, 1.0 mL/min, Hex:IPA = 3/2
注入量	: 1 uL
データファイル	: nsn588.lcd
メソッドファイル	: product.lcm
レポートファイル	: Default.lcr
分析日時	: 2015/01/26 18:36:27
解析日時	: 2015/01/26 20:00:06

<クロマトグラム>

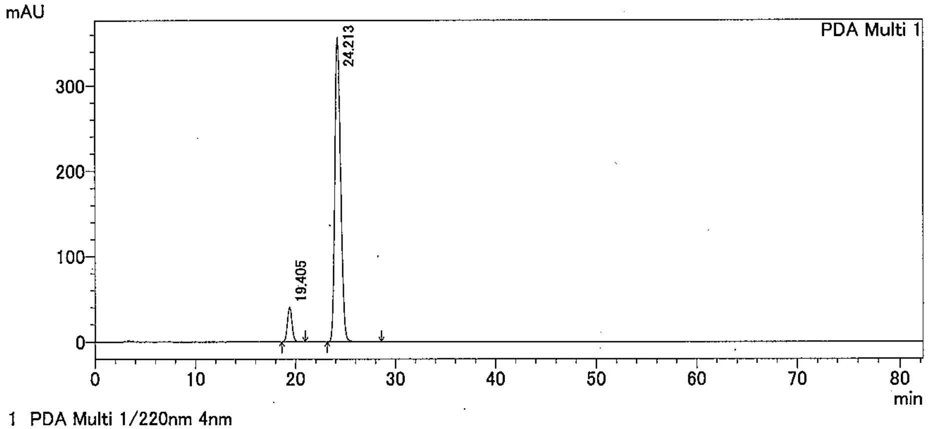

1 PDA Multi 1/220nm 4nm

ピークテーブル D:¥Nishino¥nsn588.lcd

PDA Ch1 220nm 4nm

ピーク#	保持時間	面積	高さ	面積%	高さ%
1	19.405	1284425	40682	8.166	10.227
2	24.213	14444805	357097	91.834	89.773
Total		15729229	397779	100.000	100.000

ピーク ： **peak** 保持時間**:retention time** 面積**:area** 高さ**:height**

HPLC Table 3 entry 3

==== Shimadzu LCsolution 分析レポート ====

D:¥Nishino¥nsn580.lcd

分析者	: System Administrator
サンプル名	: nsn580
サンプルID	: nsn580
測定条件 :OD-H, 1.0 mL/min, Hex:IPA = 3/2	
注入量	: 1 uL
データファイル	: nsn580.lcd
メソッドファイル	: product.lcm
レポートファイル	: Default.lcr
分析日時	: 2015/01/19 11:21:48
解析日時	: 2015/01/22 11:45:08

<クロマトグラム>

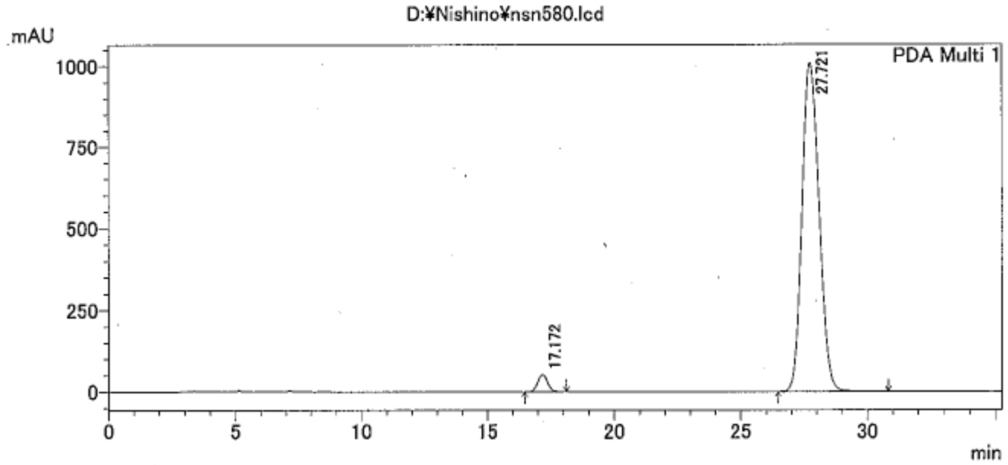

1 PDA Multi 1/220nm 4nm

ピークテーブル D:¥Nishino¥nsn580.lcd

PDA Ch1 220nm 4nm

ピーク#	保持時間	面積	高さ	面積%	高さ%
1	17.172	1414175	52857	2.894	4.978
2	27.721	47446140	1009054	97.106	95.022
Total		48860315	1061911	100.000	100.000

ピーク ： **peak** 保持時間**:retention time** 面積**:area** 高さ**:height**

HPLC Table 3 entry 4

==== Shimadzu LCsolution 分析レポート ====

D:¥Nishino¥nsn583.lcd

分析者　: System Administrator
サンプル名　: nsn583
サンプルID　: nsn583
測定条件　:OD-H, 1.0 mL/min, Hex:IPA = 3/2
注入量　: 1 uL
データファイル　: nsn583.lcd
メソッドファイル　: product.lcm

レポートファイル　: Default.lcr
分析日時　: 2015/01/19 13:03:16
解析日時　: 2015/01/22 11:46:30

<クロマトグラム>

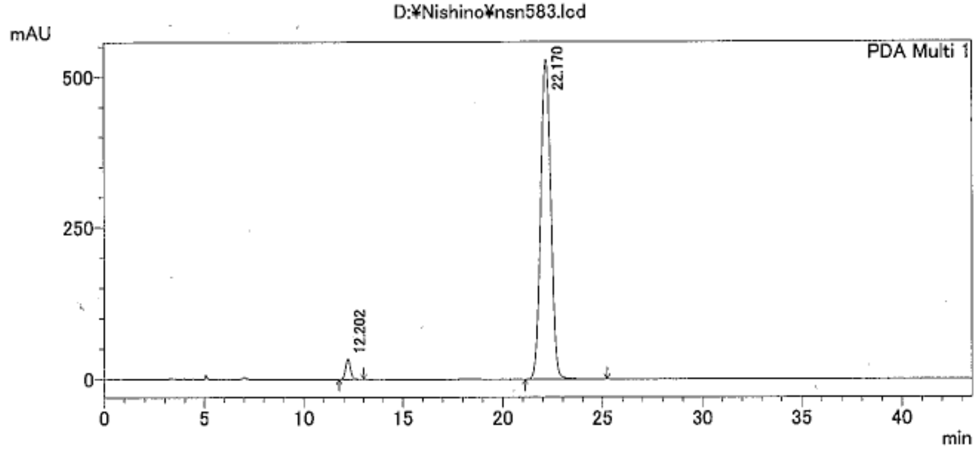

1 PDA Multi 1/220nm 4nm

ピークテーブル D:¥Nishino¥nsn583.lcd

PDA Ch1 220nm 4nm

ピーク#	保持時間	面積	高さ	面積%	高さ%
1	12.202	585173	33263	3.057	5.926
2	22.170	18557760	528086	96.943	94.074
Total		19142933	561349	100.000	100.000

ピーク ： **peak** 保持時間**:retention time** 面積**:area** 高さ**:height**

HPLC Table 3 entry 5

==== Shimadzu LCsolution 分析レポート ====

D:¥Nishino¥nsn573.lcd

分析者 : System Administrator
サンプル名 : nsn573
サンプルID : nsn573
測定条件 :OD-H, 1.0 mL/min, Hex:IPA = 9/1
注入量 : 1 uL
データファイル : nsn573.lcd
メソッドファイル : product.lcm

レポートファイル : Default.lcr
分析日時 : 2015/01/24 9:50:56
解析日時 : 2015/01/24 11:39:40

<クロマトグラム>

D:¥Nishino¥nsn573.lcd

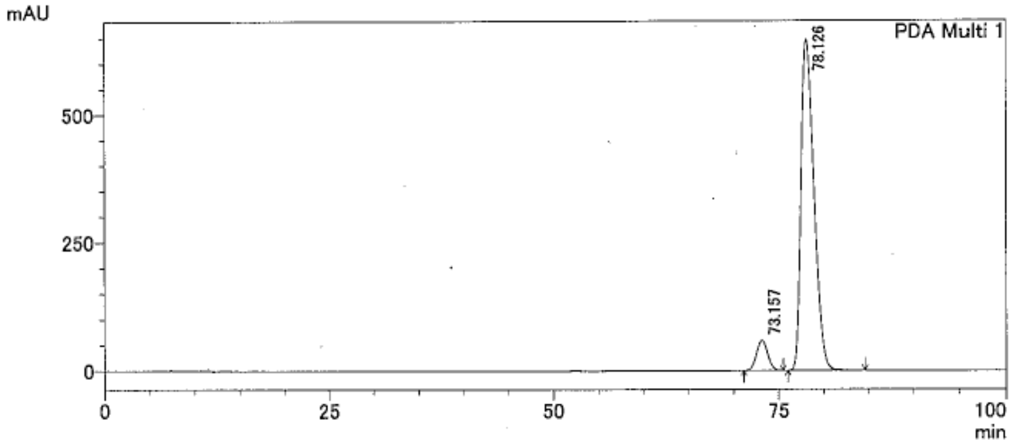

1 PDA Multi 1/220nm 4nm

ピークテーブル D:¥Nishino¥nsn573.lcd

PDA Ch1 220nm 4nm

ピーク#	保持時間	面積	高さ	面積%	高さ%
1	73.157	5026848	58687	7.250	8.328
2	78.126	64304459	646034	92.750	91.672
Total		69331307	704721	100.000	100.000

ピーク : **peak** 保持時間**:retention time** 面積**:area** 高さ**:height**

HPLC Table 3 entry 6

==== Shimadzu LCsolution 分析レポート ====

D:¥Nishino¥nsn581.lcd

分析者 : System Administrator
サンプル名 : nsn581
サンプルID : nsn581
測定条件 :OD-H, 1.0 mL/min, Hex:IPA = 3/2
注入量 : 1 uL
データファイル : nsn581.lcd
メソッドファイル : product.lcm

レポートファイル : Default.lcr
分析日時 : 2015/01/19 14:30:32
解析日時 : 2015/01/22 11:47:27

<クロマトグラム>

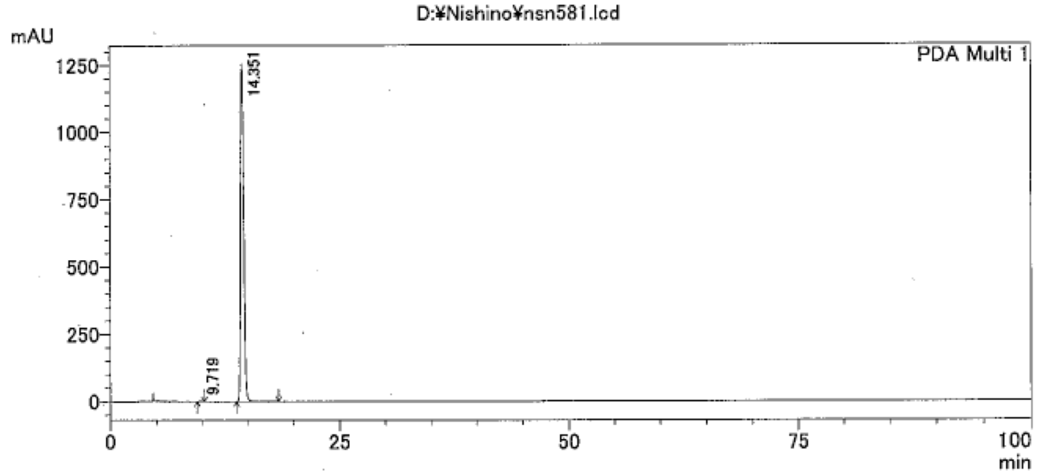

1 PDA Multi 1/220nm 4nm

ピークテーブル D:¥Nishino¥nsn581.lcd

PDA Ch1 220nm 4nm

ピーク#	保持時間	面積	高さ	面積%	高さ%
1	9.719	62927	4498	0.209	0.357
2	14.351	30083866	1254794	99.791	99.643
Total		30146792	1259292	100.000	100.000

ピーク : **peak** 保持時間**:retention time** 面積**:area** 高さ**:height**

HPLC Table 3 entry 7

==== Shimadzu LCsolution 分析レポート ====

D:¥Nishino¥nsn582.lcd

分析者 : System Administrator
サンプル名 : nsn582
サンプルID : nsn582
測定条件 :OD-H, 1.0 mL/min, Hex:IPA = 3/2
注入量 : 1 uL
データファイル : nsn582.lcd
メソッドファイル : product.lcm

レポートファイル : Default.lcr
分析日時 : 2015/01/20 16:10:30
解析日時 : 2015/01/22 11:49:20

<クロマトグラム>

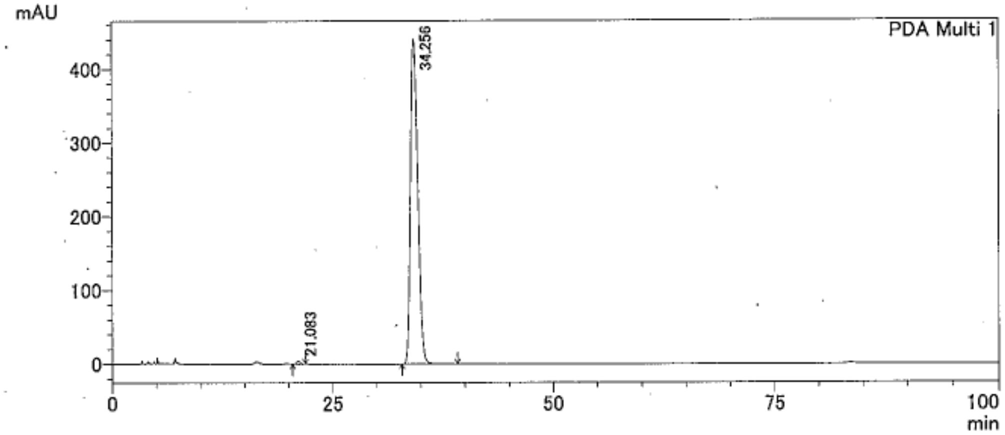

1 PDA Multi 1/220nm 4nm

ピークテーブル D:¥Nishino¥nsn582.lcd

PDA Ch1 220nm 4nm

ピーク#	保持時間	面積	高さ	面積%	高さ%
1	21.083	122173	3786	0.482	0.851
2	34.256	25224779	440914	99.518	99.149
Total		25346951	444700	100.000	100.000

ピーク : **peak** 保持時間:**retention time** 面積:**area** 高さ:**height**

HPLC Table 3 entry 8

==== Shimadzu LCsolution 分析レポート ====

D:¥Nishino¥nsn589.lcd

分析者 : System Administrator
サンプル名 : nsn589
サンプルID : nsn589
測定条件 :OD-H, 1.0 mL/min, Hex:IPA = 1/1
注入量 : 1 uL
データファイル : nsn589.lcd
メソッドファイル : product.lcm

レポートファイル : Default.lcr
分析日時 : 2015/01/26 11:57:23
解析日時 : 2015/01/26 18:34:09

<クロマトグラム>

D:¥Nishino¥nsn589.lcd

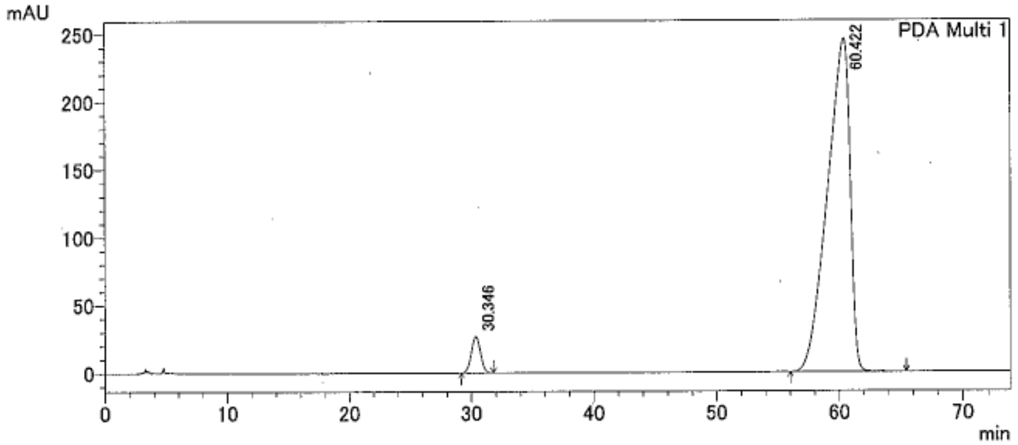

1 PDA Multi 1/220nm 4nm

ピークテーブル D:¥Nishino¥nsn589.lcd

PDA Ch1 220nm 4nm

ピーク#	保持時間	面積	高さ	面積%	高さ%
1	30.346	1383847	26852	4.206	9.859
2	60.422	31520135	245508	95.794	90.141
Total		32903982	272360	100.000	100.000

ピーク : **peak** 保持時間**:retention time** 面積**:area** 高さ**:height**

HPLC Table 3 entry 9

==== Shimadzu LCsolution 分析レポート ====

D:¥Nishino¥nsn560.lcd

分析者 : System Administrator
サンプル名 : nsn560
サンプルID : nsn560
測定条件 :OD-H, 1.0 mL/min, Hex:IPA = 3/2
注入量 : 1 uL
データファイル : nsn560.lcd
メソッドファイル : product.lcm

レポートファイル : Default.lcr
分析日時 : 2015/01/14 18:44:11
解析日時 : 2015/01/14 21:21:28

<クロマトグラム>

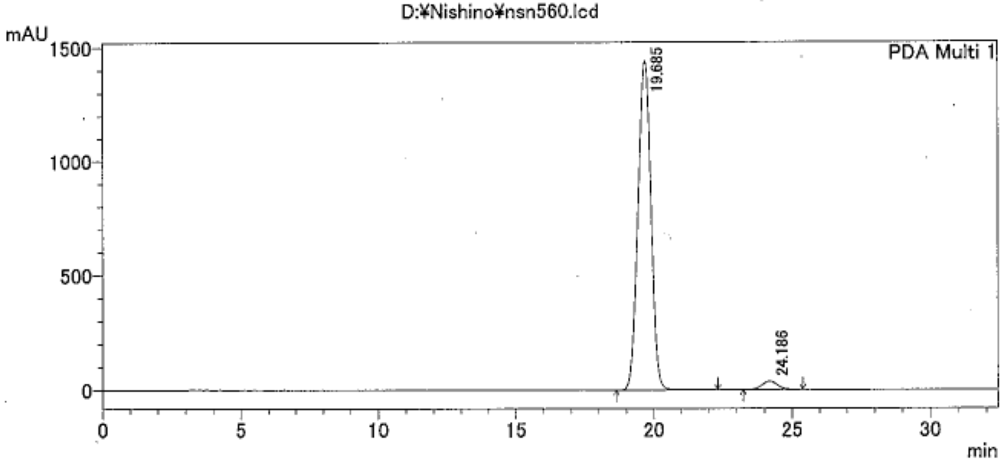

1 PDA Multi 1/220nm 4nm

ピークテーブル D:¥Nishino¥nsn560.lcd

PDA Ch1 220nm 4nm

ピーク#	保持時間	面積	高さ	面積%	高さ%
1	19.685	47583930	1441848	97.118	97.539
2	24.186	1411862	36373	2.882	2.461
Total		48995792	1478221	100.000	100.000

ピーク ： **peak** 保持時間**:retention time** 面積**:area** 高さ**:height**

HPLC Table 3 entry 10

==== Shimadzu LCsolution 分析レポート ====

D:¥Nishino¥nsn562.lcd

分析者 : System Administrator
サンプル名 : nsn562
サンプルID : nsn562
測定条件 :OD-H, 1.0 mL/min, Hex:IPA = 1/1
注入量 : 1 uL
データファイル : nsn562.lcd
メソッドファイル : product.lcm

レポートファイル : Default.lcr
分析日時 : 2015/01/13 11:42:34
解析日時 : 2015/01/13 16:38:05

<クロマトグラム>

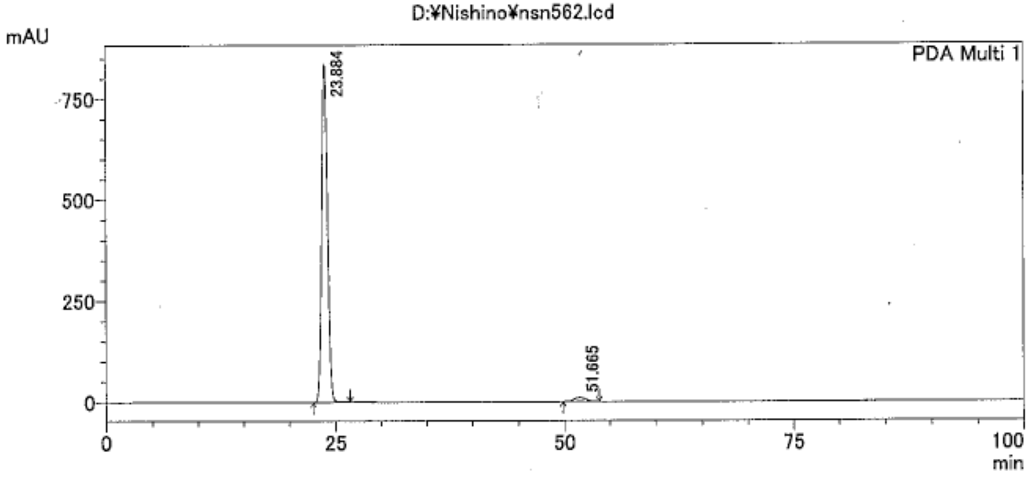

1 PDA Multi 1/220nm 4nm

ピークテーブル D:¥Nishino¥nsn562.lcd

PDA Ch1 220nm 4nm

ピーク#	保持時間	面積	高さ	面積%	高さ%
1	23.884	36911877	835648	97.514	98.831
2	51.665	941036	9888	2.486	1.169
Total		37852913	845536	100.000	100.000

ピーク ： **peak** 保持時間**:retention time** 面積**:area** 高さ**:height**

HPLC Table 3 entry 11

==== Shimadzu LCsolution 分析レポート ====

D:¥Nishino¥nsn565.lcd

分析者 : System Administrator
サンプル名 : nsn565
サンプルID : nsn565
測定条件 :OD-H, 1.0 mL/min, Hex:IPA = 9/1
注入量 : 1 uL
データファイル : nsn565.lcd
メソッドファイル : product.lcm

レポートファイル : Default.lcr
分析日時 : 2015/01/23 18:45:04
解析日時 : 2015/01/24 11:39:21

<クロマトグラム>

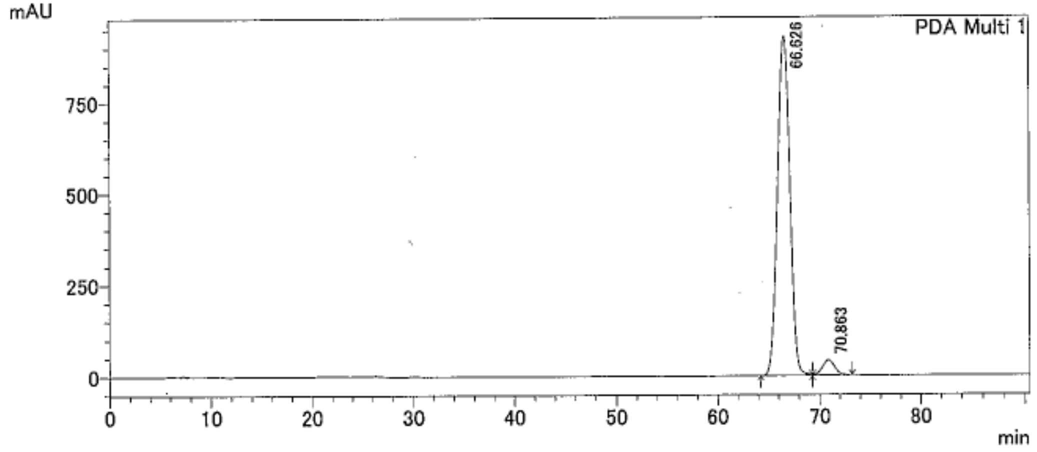

1 PDA Multi 1/220nm 4nm

ピークテーブル D:¥Nishino¥nsn565.lcd

PDA Ch1 220nm 4nm

ピーク#	保持時間	面積	高さ	面積%	高さ%
1	66.626	78170045	928085	95.744	95.747
2	70.863	3474542	41223	4.256	4.253
Total		81644587	969308	100.000	100.000

ピーク：**peak** 保持時間**:retention time** 面積**:area** 高さ**:height**

HPLC Table 3 entry 12

==== Shimadzu LCsolution 分析レポート ====

D:¥Nishino¥nsn566.lcd

分析者 : System Administrator
サンプル名 : nsn566
サンプルID : nsn566
測定条件 :OD-H, 1.0 mL/min, Hex:IPA = 3/2
注入量 : 1 uL
データファイル : nsn566.lcd
メソッドファイル : product.lcm

レポートファイル : Default.lcr
分析日時 : 2015/01/15 13:29:16
解析日時 : 2015/01/15 18:27:55

<クロマトグラム>

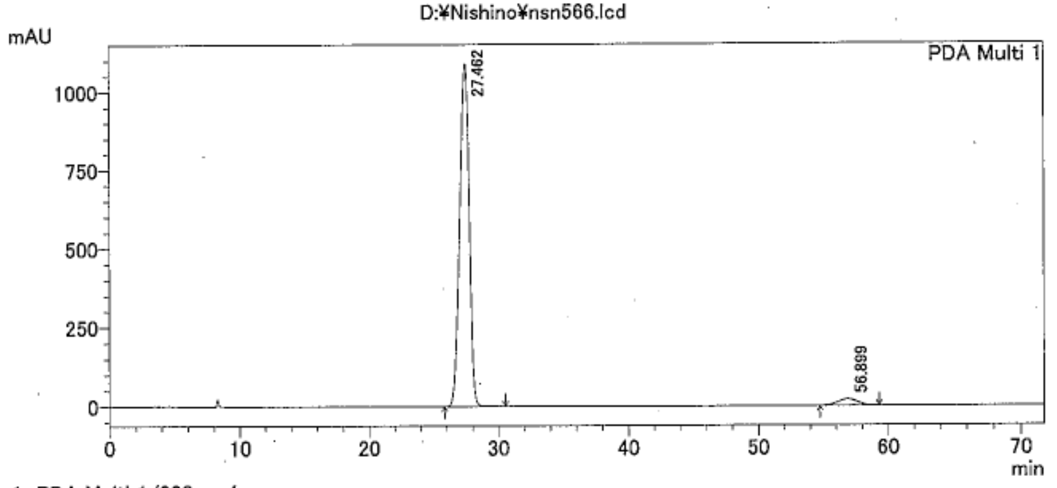

1 PDA Multi 1/220nm 4nm

ピークテーブル D:¥Nishino¥nsn566.lcd

PDA Ch1 220nm 4nm

ピーク#	保持時間	面積	高さ	面積%	高さ%
1	27.462	55666063	1089375	96.483	98.203
2	56.899	2029285	19936	3.517	1.797
Total		57695348	1109311	100.000	100.000

ピーク : **peak** 保持時間:**retention time** 面積:**area** 高さ:**height**

HPLC Table 3 entry 13

==== Shimadzu LCsolution 分析レポート ====

D:¥Nishino¥nsn563.lcd

分析者 : System Administrator
サンプル名 : nsn563
サンプルID : nsn563
測定条件 :OD-H, 1.0 mL/min, Hex:IPA = 3/2
注入量 : 1 uL
データファイル : nsn563.lcd
メソッドファイル : product.lcm

レポートファイル : Default.lcr
分析日時 : 2015/01/14 20:04:13
解析日時 : 2015/01/14 21:22:58

<クロマトグラム>

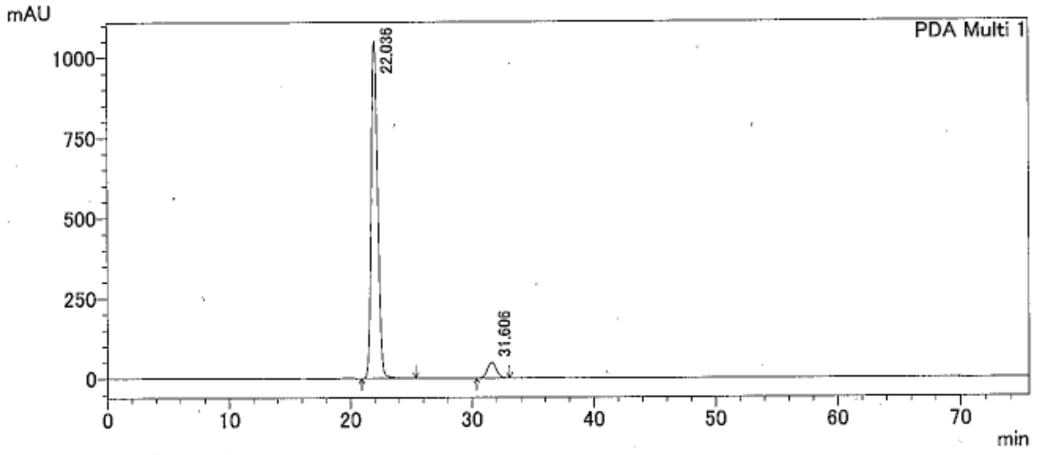

1 PDA Multi 1/220nm 4nm

ピークテーブル D:¥Nishino¥nsn563.lcd

PDA Ch1 220nm 4nm

ピーク#	保持時間	面積	高さ	面積%	高さ%
1	22.036	39063611	1050953	93.983	95.532
2	31.606	2500839	49153	6.017	4.468
Total		41564451	1100106	100.000	100.000

ピーク：**peak** 保持時間**:retention time** 面積**:area** 高さ**:height**

HPLC Table 3 entry 14

==== Shimadzu LCsolution 分析レポート ====

D:¥Nishino¥nsn564.lcd

分析者	: System Administrator
サンプル名	: nsn564
サンプルID	: nsn564
測定条件 :OD-H, 1.0 mL/min, Hex:IPA = 1/1	
注入量	: 1 uL
データファイル	: nsn564.lcd
メソッドファイル	: product.lcm
レポートファイル	: Default.lcr
分析日時	: 2015/01/13 16:36:02
解析日時	: 2015/01/14 8:53:54

<クロマトグラム>

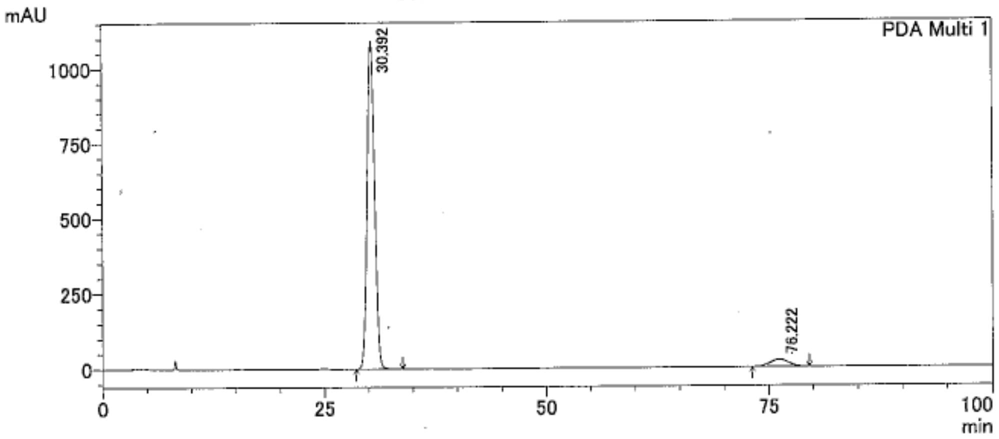

1 PDA Multi 1/220nm 4nm

ピークテーブル D:¥Nishino¥nsn564.lcd

PDA Ch1 220nm 4nm

ピーク#	保持時間	面積	高さ	面積%	高さ%
1	30.392	64373906	1091851	94.927	97.879
2	76.222	3439890	23659	5.073	2.121
Total		67813796	1115510	100.000	100.000

ピーク：peak 保持時間:retention time 面積:area 高さ:height

HPLC Table 3 entry 15

==== Shimadzu LCsolution 分析レポート ====

D:¥Nishino¥nsn567.lcd

分析者 : System Administrator
サンプル名 : nsn567
サンプルID : nsn567
測定条件 :OD-H, 1.0 mL/min, Hex:IPA = 3/2
注入量 : 1 uL
データファイル : nsn567.lcd
メソッドファイル : product.lcm

レポートファイル : Default.lcr
分析日時 : 2015/01/19 12:32:06
解析日時 : 2015/01/22 11:46:10

<クロマトグラム>

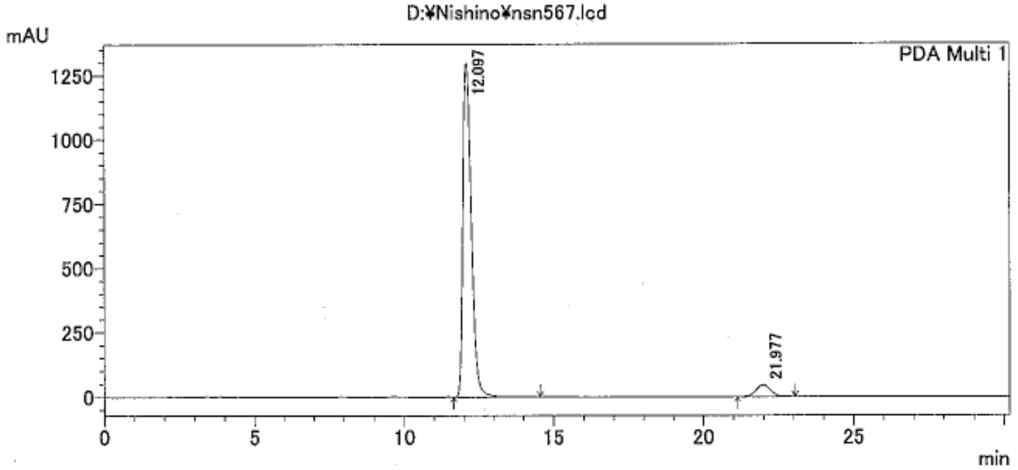

1 PDA Multi 1/220nm 4nm

ピークテーブル D:¥Nishino¥nsn567.lcd

PDA Ch1 220nm 4nm

ピーク#	保持時間	面積	高さ	面積%	高さ%
1	12.097	25708740	1296970	94.571	96.677
2	21.977	1475761	44576	5.429	3.323
Total		27184502	1341546	100.000	100.000

ピーク ： **peak** 保持時間**:retention time** 面積**:area** 高さ**:height**

HPLC Table 3 entry 16

==== Shimadzu LCsolution 分析レポート ====

D:¥Nishino¥nsn568.lcd

分析者 : System Administrator
サンプル名 : nsn568
サンプルID : nsn568
測定条件 :OD-H, 1.0 mL/min, Hex:IPA = 3/2
注入量 : 1 uL
データファイル : nsn568.lcd
メソッドファイル : product.lcm

レポートファイル : Default.lcr
分析日時 : 2015/01/15 10:58:55
解析日時 : 2015/01/15 18:34:35

<クロマトグラム>

D:¥Nishino¥nsn568.lcd

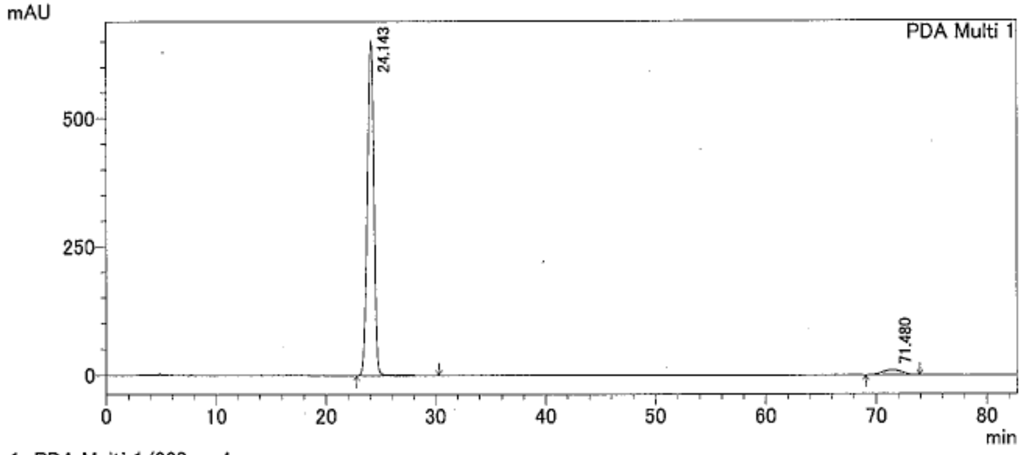

1 PDA Multi 1/220nm 4nm

ピークテーブル D:¥Nishino¥nsn568.lcd

PDA Ch1 220nm 4nm

ピーク#	保持時間	面積	高さ	面積%	高さ%
1	24.143	27240837	652299	95.734	98.488
2	71.480	1213859	10016	4.266	1.512
Total		28454696	662314	100.000	100.000

ピーク ： **peak** 保持時間**:retention time** 面積**:area** 高さ**:height**

HPLC Table 3 entry 17

==== Shimadzu LCsolution 分析レポート ====

D:¥Nishino¥nsn569.lcd

分析者	: System Administrator
サンプル名	: nsn569
サンプルID	: nsn569
測定条件 :OD-H, 1.0 mL/min, Hex:IPA = 3/2	
注入量	: 1 uL
データファイル	: nsn569.lcd
メソッドファイル	: product.lcm
レポートファイル	: Default.lcr
分析日時	: 2015/01/15 15:09:14
解析日時	: 2015/01/15 18:31:50

<クロマトグラム>

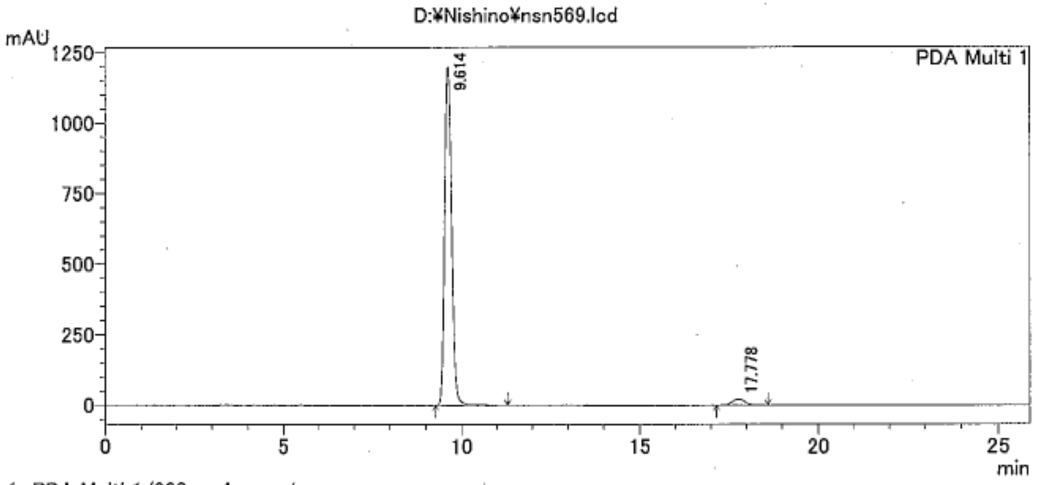

1 PDA Multi 1/220nm 4nm

ピークテーブル D:¥Nishino¥nsn569.lcd

PDA Ch1 220nm 4nm

ピーク#	保持時間	面積	高さ	面積%	高さ%
1	9.614	16246798	1200320	96.815	98.240
2	17.778	534475	21506	3.185	1.760
Total		16781272	1221826	100.000	100.000

ピーク : **peak** 保持時間**:retention time** 面積**:area** 高さ**:height**

HPLC Table 3 entry 18

==== Shimadzu LCsolution 分析レポート ====

D:¥Nishino¥nsn570.lcd

分析者 : System Administrator
サンプル名 : nsn570
サンプルID : nsn570
測定条件 :OD-H, 1.0 mL/min, Hex:IPA = 3/2
注入量 : 1 uL
データファイル : nsn570.lcd
メソッドファイル : product.lcm

レポートファイル : Default.lcr
分析日時 : 2015/01/15 17:15:50
解析日時 : 2015/01/15 18:32:38

<クロマトグラム>

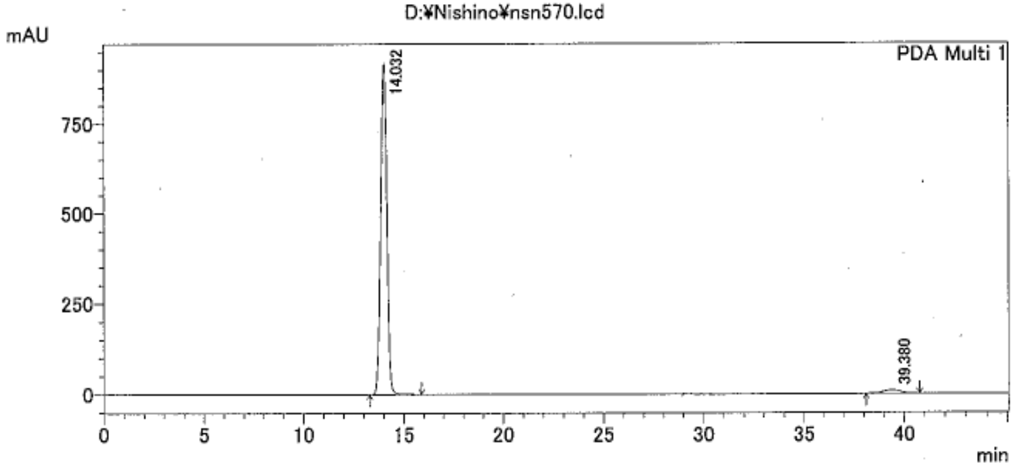

1 PDA Multi 1/220nm 4nm

ﾋﾟｰｸﾃｰﾌﾞﾙ D:¥Nishino¥nsn570.lcd

PDA Ch1 220nm 4nm

ピーク#	保持時間	面積	高さ	面積%	高さ%
1	14.032	19817489	919554	97.250	99.005
2	39.380	560436	9240	2.750	0.995
Total		20377925	928793	100.000	100.000

ピーク : **peak** 保持時間**:retention time** 面積**:area** 高さ**:height**

HPLC Table 3 entry 19

==== Shimadzu LCsolution 分析レポート ====

D:¥Nishino¥nsn571.lcd

分析者 : System Administrator
サンプル名 : nsn571
サンプルID : nsn571
測定条件 :OD-H, 1.0 mL/min, Hex:IPA = 3/2
注入量 : 1 uL
データファイル : nsn571.lcd
メソッドファイル : product.lcm

レポートファイル : Default.lcr
分析日時 : 2015/01/16 10:55:20
解析日時 : 2015/01/19 9:27:35

<クロマトグラム>

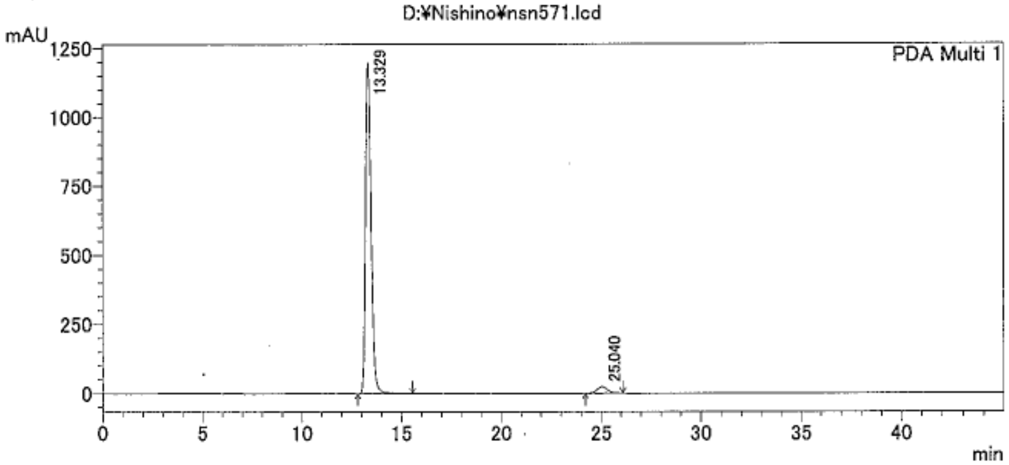

1 PDA Multi 1/220nm 4nm

ピークテーブル D:¥Nishino¥nsn571.lcd

PDA Ch1 220nm 4nm

ピーク#	保持時間	面積	高さ	面積%	高さ%
1	13.329	23336209	1197206	96.579	98.122
2	25.040	826508	22916	3.421	1.878
Total		24162717	1220123	100.000	100.000

ピーク ： **peak** 保持時間**:retention time** 面積**:area** 高さ**:height**

HPLC Table 3 entry 20

==== Shimadzu LCsolution 分析レポート ====

D:¥Nishino¥nsn572.lcd

分析者 : System Administrator
サンプル名 : nsn572
サンプルID : nsn572
測定条件 :OD-H, 1.0 mL/min, Hex:IPA = 3/2
注入量 : 1 uL
データファイル : nsn572.lcd
メソッドファイル : product.lcm

レポートファイル : Default.lcr
分析日時 : 2015/01/16 12:58:37
解析日時 : 2015/01/19 9:28:12

<クロマトグラム>

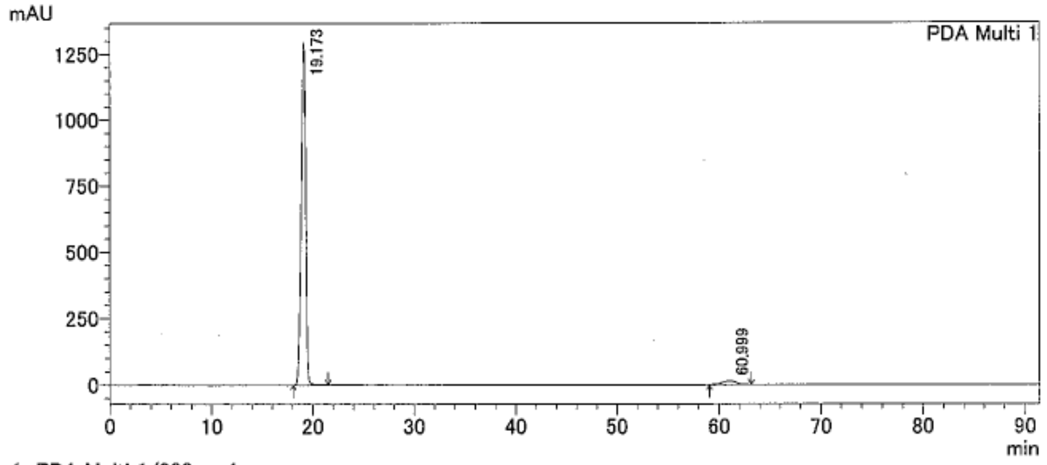

1 PDA Multi 1/220nm 4nm

ピークテーブル D:¥Nishino¥nsn572.lcd

PDA Ch1 220nm 4nm

ピーク#	保持時間	面積	高さ	面積%	高さ%
1	19.173	39354747	1294702	96.812	98.947
2	60.999	1295811	13780	3.188	1.053
Total		40650558	1308482	100.000	100.000

ピーク : **peak** 保持時間**:retention time** 面積**:area** 高さ**:height**

HPLC Table 3 entry 21

==== Shimadzu LCsolution 分析レポート ====

D:¥Nishino¥nsn574.lcd

分析者 : System Administrator
サンプル名 : nsn574
サンプルID : nsn574
測定条件 :OD-H, 1.0 mL/min, Hex:IPA = 3/2
注入量 : 1 uL
データファイル : nsn574.lcd
メソッドファイル : product.lcm

レポートファイル : Default.lcr
分析日時 : 2015/01/16 14:50:20
解析日時 : 2015/01/19 9:29:25

<クロマトグラム>

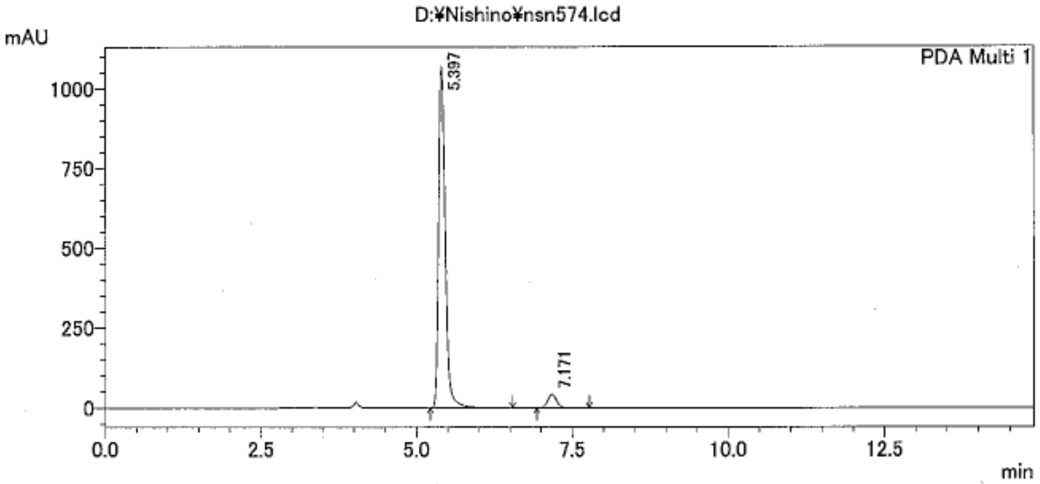

1 PDA Multi 1/220nm 4nm

ピークテーブル D:¥Nishino¥nsn574.lcd

PDA Ch1 220nm 4nm

ピーク#	保持時間	面積	高さ	面積%	高さ%
1	5.397	7976508	1070782	95.055	96.162
2	7.171	414959	42734	4.945	3.838
Total		8391468	1113516	100.000	100.000

ピーク ： **peak** 保持時間**:retention time** 面積**:area** 高さ**:height**

HPLC Table 3 entry 22

==== Shimadzu LCsolution 分析レポート ====

D:¥Nishino¥nsn575.lcd

分析者 : System Administrator
サンプル名 : nsn575
サンプルID : nsn575
測定条件 :OD-H, 1.0 mL/min, Hex:IPA = 3/2
注入量 : 1 uL
データファイル : nsn575.lcd
メソッドファイル : product.lcm

レポートファイル : Default.lcr
分析日時 : 2015/01/15 18:23:50
解析日時 : 2015/01/15 18:49:47

<クロマトグラム>

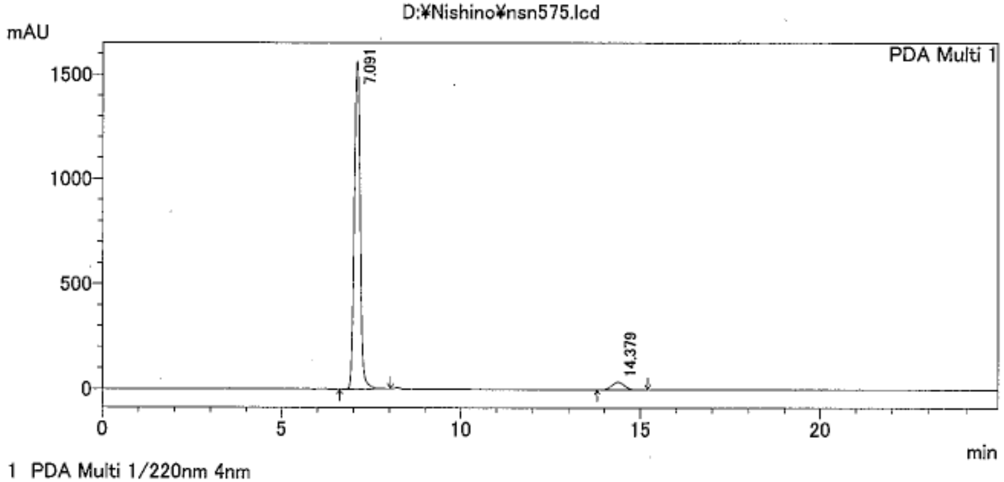

1 PDA Multi 1/220nm 4nm

ﾋﾟｰｸﾃｰﾌﾞﾙ D:¥Nishino¥nsn575.lcd

PDA Ch1 220nm 4nm

ピーク#	保持時間	面積	高さ	面積%	高さ%
1	7.091	18118541	1564103	95.443	97.783
2	14.379	865087	35471	4.557	2.217
Total		18983627	1599573	100.000	100.000

ピーク : **peak** 保持時間**:retention time** 面積**:area** 高さ**:height**

HPLC Table 3 entry 23

==== Shimadzu LCsolution 分析レポート ====

D:¥Nishino¥nsn576.lcd

分析者 : System Administrator
サンプル名 : nsn576
サンプルID : nsn576
測定条件 :OD-H, 1.0 mL/min, Hex:IPA = 3/2
注入量 : 1 uL
データファイル : nsn576.lcd
メソッドファイル : product.lcm

レポートファイル : Default.lcr
分析日時 : 2015/01/16 15:54:31
解析日時 : 2015/01/19 9:30:19

<クロマトグラム>

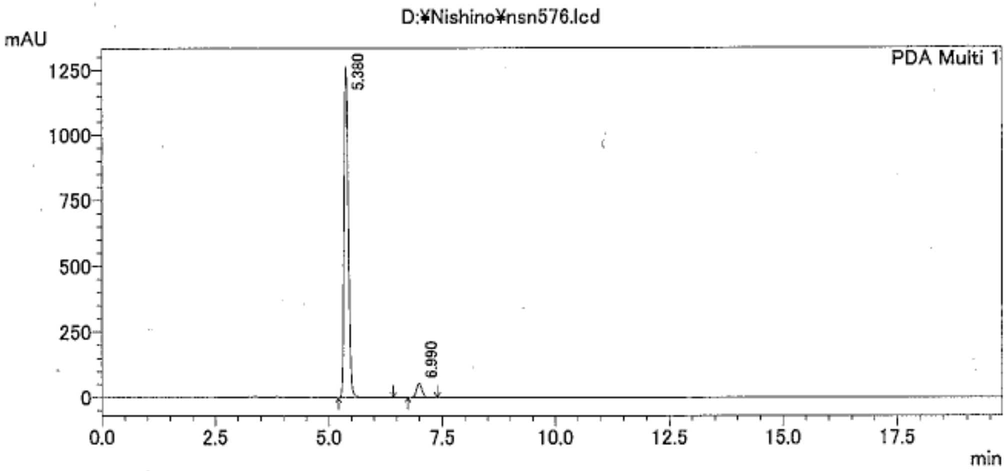

1 PDA Multi 1/220nm 4nm

ピークテーブル D:¥Nishino¥nsn576.lcd

PDA Ch1 220nm 4nm

ピーク#	保持時間	面積	高さ	面積%	高さ%
1	5.380	8315193	1262757	94.919	95.923
2	6.990	445133	53671	5.081	4.077
Total		8760326	1316428	100.000	100.000

ピーク：**peak** 保持時間**:retention time** 面積**:area** 高さ**:height**

HPLC Table 3 entry 24

==== Shimadzu LCsolution 分析レポート ====

D:¥Nishino¥nsn577.lcd

分析者 : System Administrator
サンプル名 : nsn577
サンプルID : nsn577
測定条件 :OD-H, 1.0 mL/min, Hex:IPA = 3/2
注入量 : 1 uL
データファイル : nsn577.lcd
メソッドファイル : product.lcm

レポートファイル : Default.lcr
分析日時 : 2015/01/16 17:46:45
解析日時 : 2015/01/19 9:31:02

<クロマトグラム>

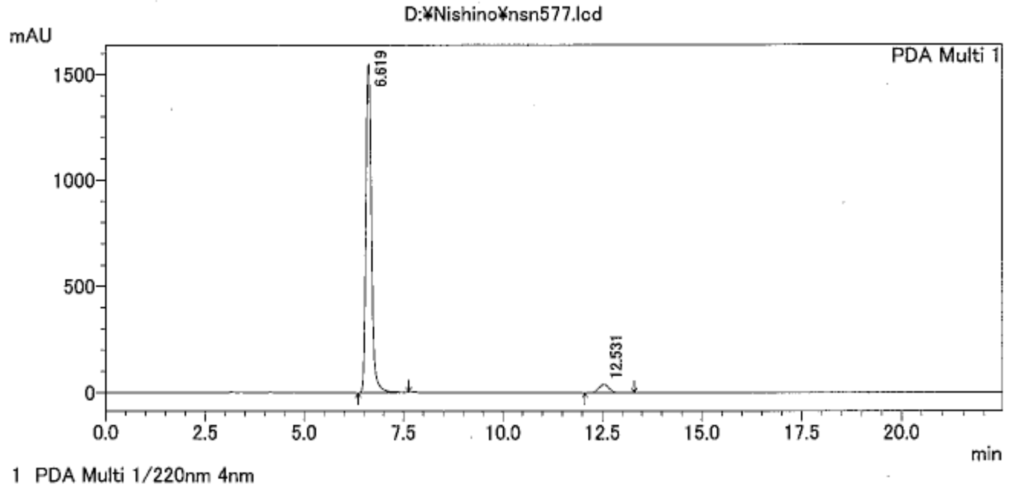

1 PDA Multi 1/220nm 4nm

ピークテーブル D:¥Nishino¥nsn577.lcd

PDA Ch1 220nm 4nm

ピーク#	保持時間	面積	高さ	面積%	高さ%
1	6.619	15141597	1550805	95.617	97.579
2	12.531	694025	38476	4.383	2.421
Total		15835622	1589281	100.000	100.000

ピーク : **peak** 保持時間**:retention time** 面積**:area** 高さ**:height**

HPLC Table 3 entry 25

==== Shimadzu LCsolution 分析レポート ====

D:¥Nishino¥nsn578.lcd

分析者 : System Administrator
サンプル名 : nsn578
サンプルID : nsn578
測定条件 :OD-H, 1.0 mL/min, Hex:IPA = 99/1
注入量 : 1 uL
データファイル : nsn578.lcd
メソッドファイル : product.lcm

レポートファイル : Default.lcr
分析日時 : 2015/01/22 13:33:18
解析日時 : 2015/01/22 17:32:45

<クロマトグラム>

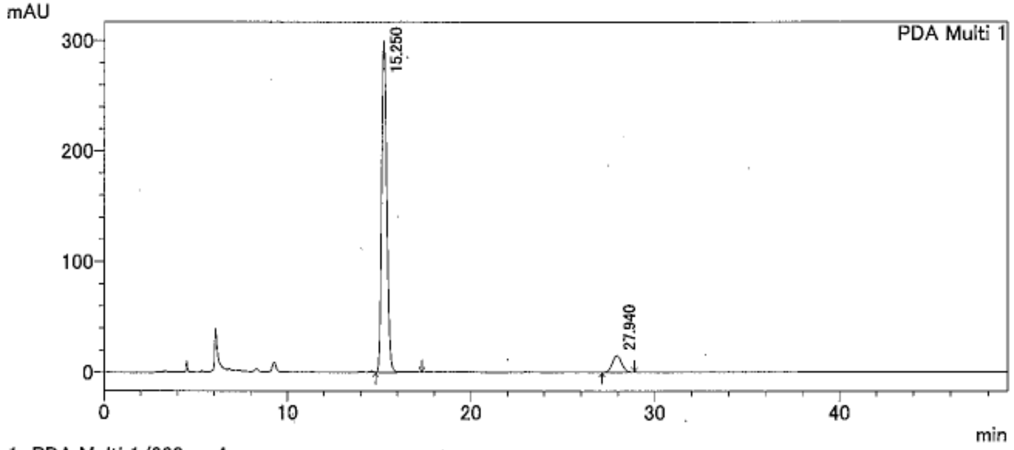

1 PDA Multi 1/220nm 4nm

ピークテーブル D:¥Nishino¥nsn578.lcd

PDA Ch1 220nm 4nm

ピーク#	保持時間	面積	高さ	面積%	高さ%
1	15.250	5819057	299671	91.783	95.264
2	27.940	520963	14897	8.217	4.736
Total		6340020	314568	100.000	100.000

ピーク ： **peak** 保持時間**:retention time** 面積**:area** 高さ**:height**

HPLC Table 3 entry 26

==== Shimadzu LCsolution 分析レポート ====

D:¥Nishino¥nsn579.lcd

分析者 : System Administrator
サンプル名 : nsn579
サンプルID : nsn579
測定条件 :OD-H, 1.0 mL/min, Hex:IPA = 99/1
注入量 : 1 uL
データファイル : nsn579.lcd
メソッドファイル : product.lcm

レポートファイル : Default.lcr
分析日時 : 2015/01/22 16:09:20
解析日時 : 2015/01/22 18:53:40

<クロマトグラム>

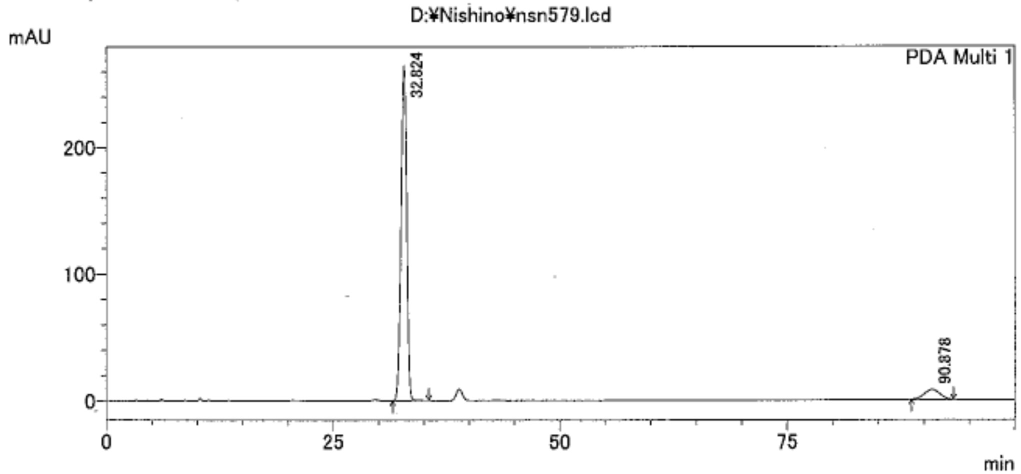

1 PDA Multi 1/220nm 4nm

ピークテーブル D:¥Nishino¥nsn579.lcd

PDA Ch1 220nm 4nm

ピーク#	保持時間	面積	高さ	面積%	高さ%
1	32.824	11742360	264591	92.835	97.183
2	90.878	906261	7671	7.165	2.817
Total		12648621	272262	100.000	100.000

ピーク : **peak** 保持時間**:retention time** 面積**:area** 高さ**:height**

HPLC Table 9 entry 1

==== Shimadzu LCsolution 分析レポート ====

D:¥Nishino¥nsn620.lcd

分析者 : System Administrator
サンプル名 : nsn620
サンプルID : nsn620
測定条件 :OD-H,1.0 mL/min, Hex:IPA = 1 /1
注入量 : 1 uL
データファイル : nsn620.lcd
メソッドファイル : product.lcm

レポートファイル : Default.lcr
分析日時 : 2015/02/23 15:41:04
解析日時 : 2018/07/07 17:41:45

<クロマトグラム>

D:¥Nishino¥nsn620.lcd

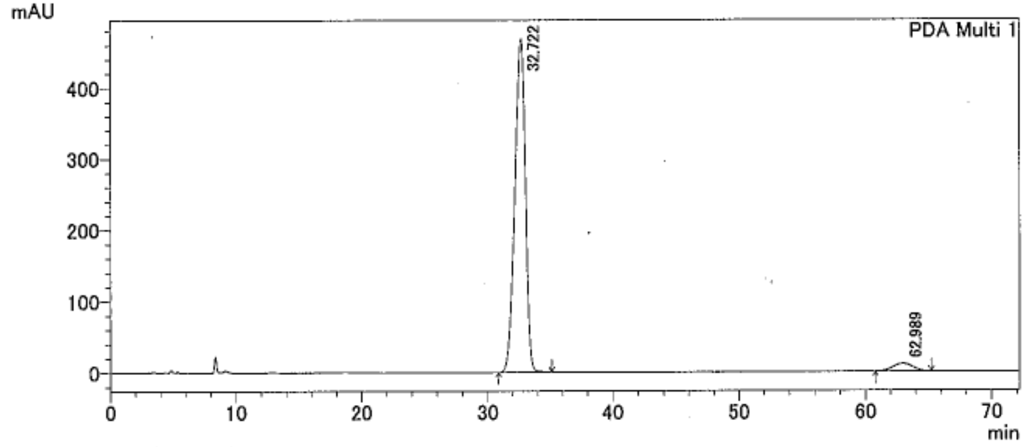

1 PDA Multi 1/220nm 4nm

ピークテーブル D:¥Nishino¥nsn620.lcd

PDA Ch1 220nm 4nm

ピーク#	保持時間	面積	高さ	面積%	高さ%
1	32.722	27731506	468585	95.874	97.742
2	62.989	1193513	10826	4.126	2.258
Total		28925018	479411	100.000	100.000

ピーク ： **peak** 保持時間**:retention time** 面積**:area** 高さ**:height**

HPLC Table 9 entry 2

==== Shimadzu LCsolution 分析レポート ====

D:¥Nishino¥nsn679.lcd

分析者 : System Administrator
サンプル名 : nsn679
サンプルID : nsn679
測定条件 :OD-H, 1.0mL/min, Hex:IPA =3/2
注入量 : 1 uL
データファイル : nsn679.lcd
メソッドファイル : product.lcm

レポートファイル : Default.lcr
分析日時 : 2015/05/19 10:21:34
解析日時 : 2015/05/20 8:13:31

<クロマトグラム>

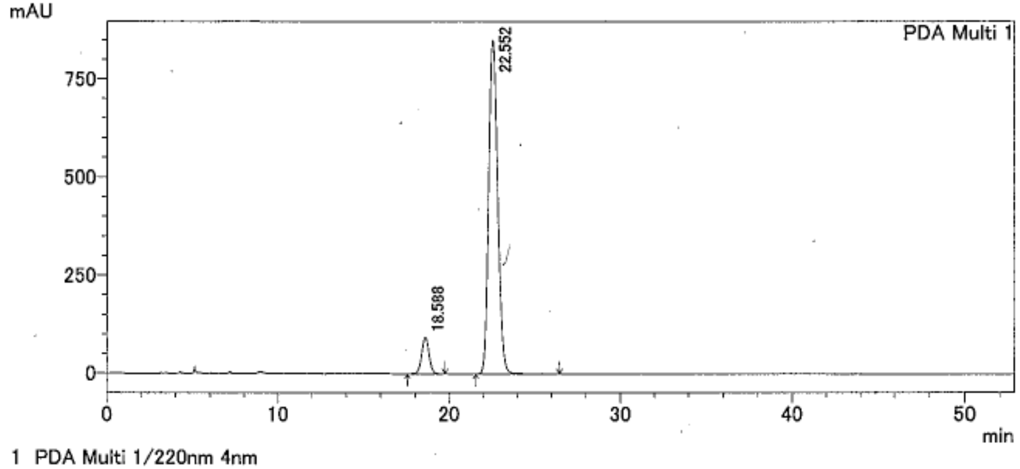

ﾋﾟｰｸﾃｰﾌﾞﾙ D:¥Nishino¥nsn679.lcd

PDA Ch1 220nm 4nm

ピーク#	保持時間	面積	高さ	面積%	高さ%
1	18.588	2787721	93608	8.137	9.908
2	22.552	31472314	851146	91.863	90.092
Total		34260035	944753	100.000	100.000

ピーク : **peak** 保持時間**:retention time** 面積**:area** 高さ**:height**

HPLC Table 9 entry 3

==== Shimadzu LCsolution 分析レポート ====

D:¥Nishino¥nsn681.lcd

分析者 : System Administrator
サンプル名 : nsn681
サンプルID : nsn681
測定条件 :OD-H, 1.0mL/min, Hex:IPA =3/2
注入量 : 1 uL
データファイル : nsn681.lcd
メソッドファイル : product.lcm

レポートファイル : Default.lcr
分析日時 : 2015/05/19 14:33:50
解析日時 : 2015/05/20 8:16:57

<クロマトグラム>

D:¥Nishino¥nsn681.lcd

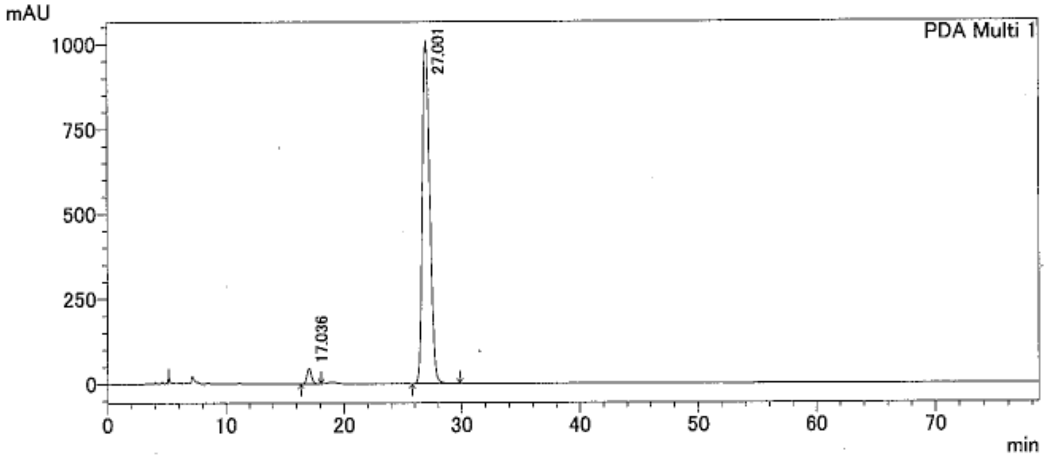

1 PDA Multi 1/220nm 4nm

ピークテーブル D:¥Nishino¥nsn681.lcd

PDA Ch1 220nm 4nm

ピーク#	保持時間	面積	高さ	面積%	高さ%
1	17.036	1228835	46053	2.678	4.373
2	27.001	44652613	1007096	97.322	95.627
Total		45881448	1053150	100.000	100.000

ピーク : **peak** 保持時間**:retention time** 面積**:area** 高さ**:height**

HPLC Table 9 entry 4

==== Shimadzu LCsolution 分析レポート ====

D:¥Nishino¥nsn680.lcd

分析者 : System Administrator
サンプル名 : nsn680
サンプルID : nsn680
測定条件 :OD-H, 1.0mL/min, Hex:IPA =3/2
注入量 : 1 uL
データファイル : nsn680.lcd
メソッドファイル : product.lcm

レポートファイル : Default.lcr
分析日時 : 2015/05/19 11:15:11
解析日時 : 2015/05/20 8:24:34

<クロマトグラム>

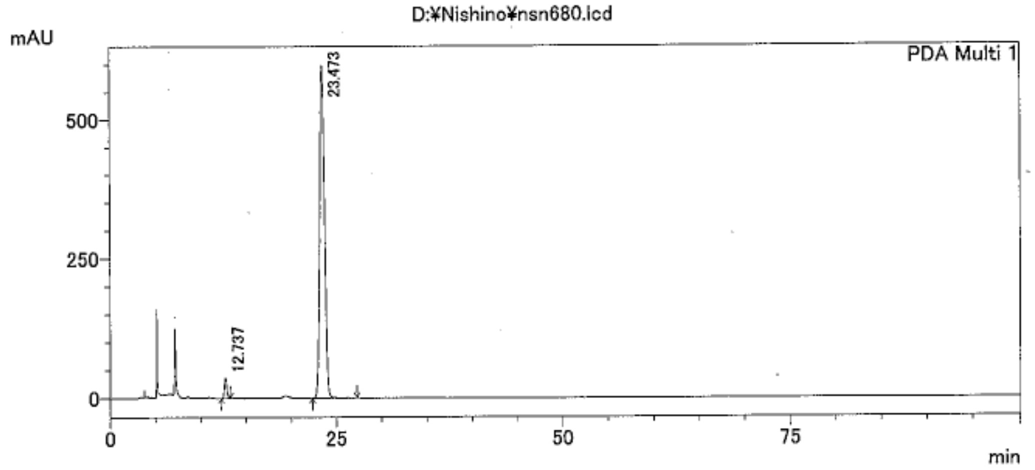

1 PDA Multi 1/220nm 4nm

ピークテーブル D:¥Nishino¥nsn680.lcd

PDA Ch1 220nm 4nm

ピーク#	保持時間	面積	高さ	面積%	高さ%
1	12.737	659991	36272	2.855	5.709
2	23.473	22460303	599043	97.145	94.291
Total		23120294	635314	100.000	100.000

ピーク ： **peak** 保持時間**:retention time** 面積**:area** 高さ**:height**

HPLC Table 9 entry 5

==== Shimadzu LCsolution 分析レポート ====

D:¥Nishino¥nsn682.lcd

分析者 : System Administrator
サンプル名 : nsn682
サンプルID : nsn682
測定条件 :OD-H, 1.0mL/min, Hex:IPA =3/2
注入量 : 1 uL
データファイル : nsn682.lcd
メソッドファイル : product.lcm

レポートファイル : Default.lcr
分析日時 : 2015/05/19 15:54:10
解析日時 : 2015/05/20 8:18:18

<クロマトグラム>

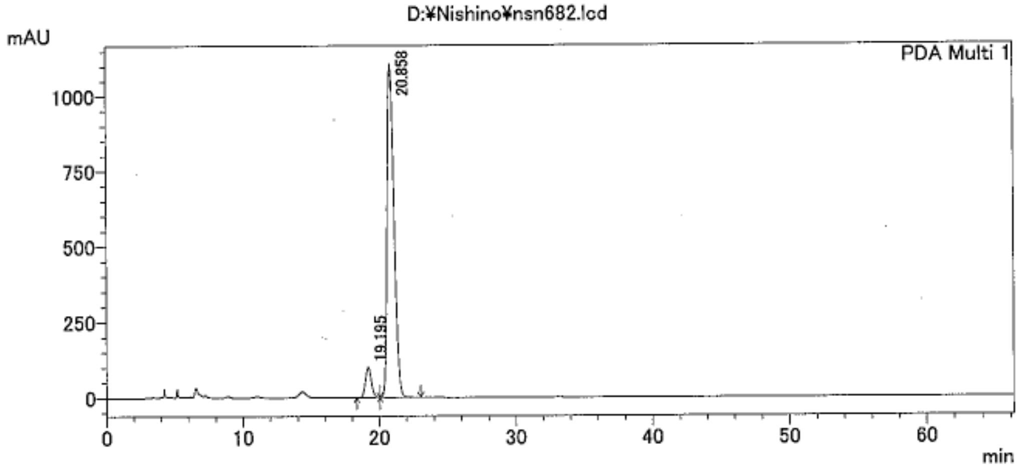

1 PDA Multi 1/220nm 4nm

ピークテーブル D:¥Nishino¥nsn682.lcd

PDA Ch1 220nm 4nm

ピーク#	保持時間	面積	高さ	面積%	高さ%
1	19.195	3054088	100621	7.300	8.335
2	20.858	38784218	1106633	92.700	91.665
Total		41838306	1207254	100.000	100.000

ピーク : **peak** 保持時間**:retention time** 面積**:area** 高さ**:height**

HPLC Table 9 entry 6

==== Shimadzu LCsolution 分析レポート ====

D:¥Nishino¥nsn703.lcd

分析者 : System Administrator
サンプル名 : nsn703
サンプルID : nsn703
測定条件 :OD-H, 1.0mL/min, Hex:IPA =3/2
注入量 : 1 uL
データファイル : nsn703.lcd
メソッドファイル : product.lcm

レポートファイル : Default.lcr
分析日時 : 2015/05/23 13:32:30
解析日時 : 2018/07/07 17:47:47

<クロマトグラム>

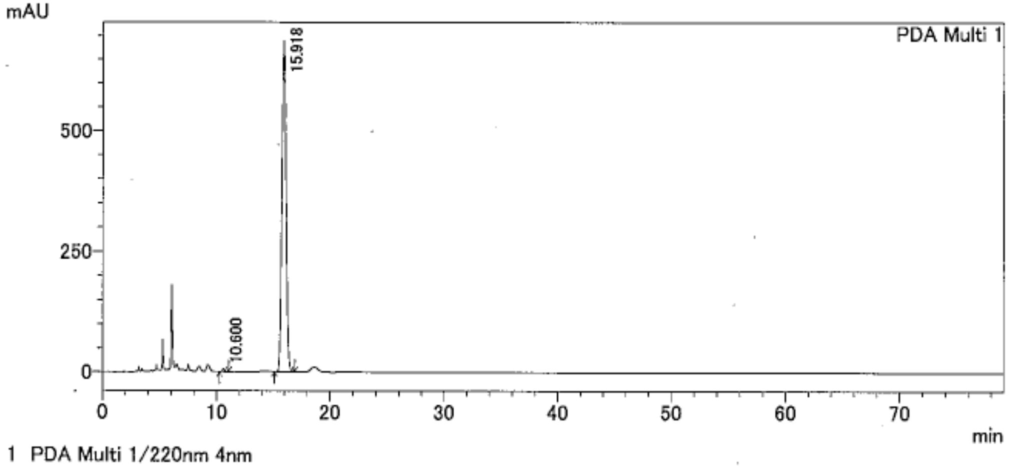

1 PDA Multi 1/220nm 4nm

ピークテーブル D:¥Nishino¥nsn703.lcd

PDA Ch1 220nm 4nm

ピーク#	保持時間	面積	高さ	面積%	高さ%
1	10.600	92466	6330	0.520	0.911
2	15.918	17683292	688482	99.480	99.089
Total		17775758	694812	100.000	100.000

ピーク ： **peak** 保持時間**:retention time** 面積**:area** 高さ**:height**

HPLC Table 9 entry 7

==== Shimadzu LCsolution 分析レポート ====

D:¥Nishino¥nsn711.lcd

分析者 : System Administrator
サンプル名 : nsn711
サンプルID : nsn711
測定条件 :OD-H, 1.0mL/min, Hex:IPA =3/2
注入量 : 1 uL
データファイル : nsn711.lcd
メソッドファイル : product.lcm

レポートファイル : Default.lcr
分析日時 : 2015/05/23 14:52:36
解析日時 : 2015/05/27 8:34:56

<クロマトグラム>

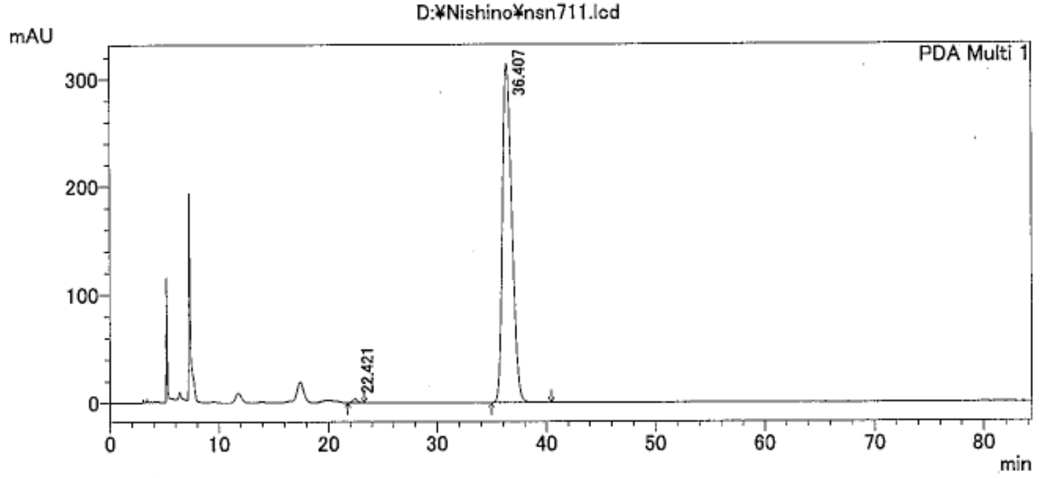

1 PDA Multi 1/220nm 4nm

ピークテーブル D:¥Nishino¥nsn711.lcd

PDA Ch1 220nm 4nm

ピーク#	保持時間	面積	高さ	面積%	高さ%
1	22.421	119037	3471	0.623	1.092
2	36.407	18985218	314448	99.377	98.908
Total		19104255	317920	100.000	100.000

ピーク ： **peak** 保持時間**:retention time** 面積**:area** 高さ**:height**

HPLC Table 9 entry 8

==== Shimadzu LCsolution 分析レポート ====

D:¥Nishino¥nsn647.lcd

分析者 : System Administrator
サンプル名 : nsn647
サンプルID : nsn647
測定条件 :OD-H, 1.0 mL/min, Hex:IPA = 1/1
注入量 : 1 uL
データファイル : nsn647.lcd
メソッドファイル : product.lcm

レポートファイル : Default.lcr
分析日時 : 2015/04/01 15:28:32
解析日時 : 2015/04/01 19:35:12

<クロマトグラム>

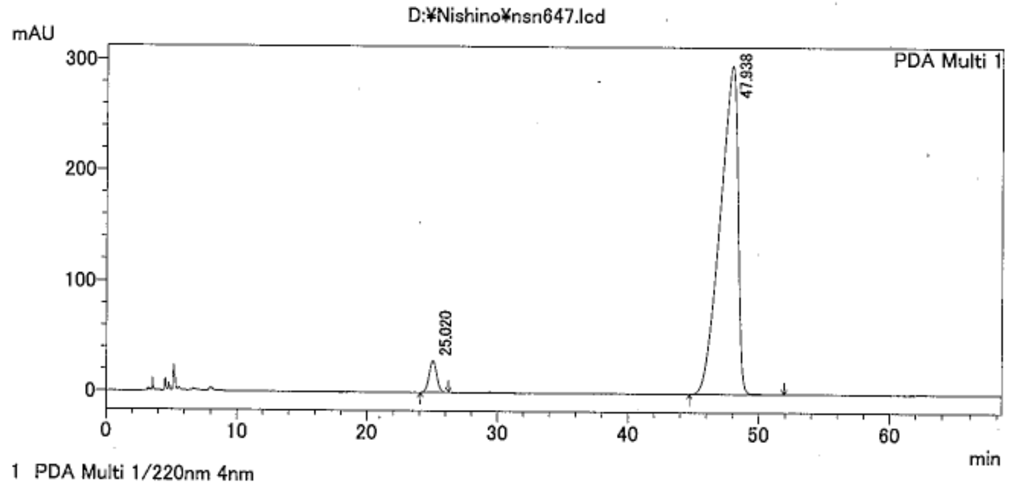

1 PDA Multi 1/220nm 4nm

ピークテーブル D:¥Nishino¥nsn647.lcd

PDA Ch1 220nm 4nm

ピーク#	保持時間	面積	高さ	面積%	高さ%
1	25.020	1189884	28629	4.084	8.815
2	47.938	27946386	296143	95.916	91.185
Total		29136270	324773	100.000	100.000

ピーク : **peak** 保持時間**:retention time** 面積**:area** 高さ**:height**

HPLC Table 9 entry 9

==== Shimadzu LCsolution 分析レポート ====

D:¥Nishino¥nsn646.lcd

分析者 : System Administrator
サンプル名 : nsn646
サンプルID : nsn646
測定条件 :OD-H, 1.0mL/min, Hex:IPA =3/2
注入量 : 1 uL
データファイル : nsn646.lcd
メソッドファイル : product.lcm

レポートファイル : Default.lcr
分析日時 : 2015/04/07 14:24:40
解析日時 : 2015/04/07 19:22:09

<クロマトグラム>

D:¥Nishino¥nsn646.lcd

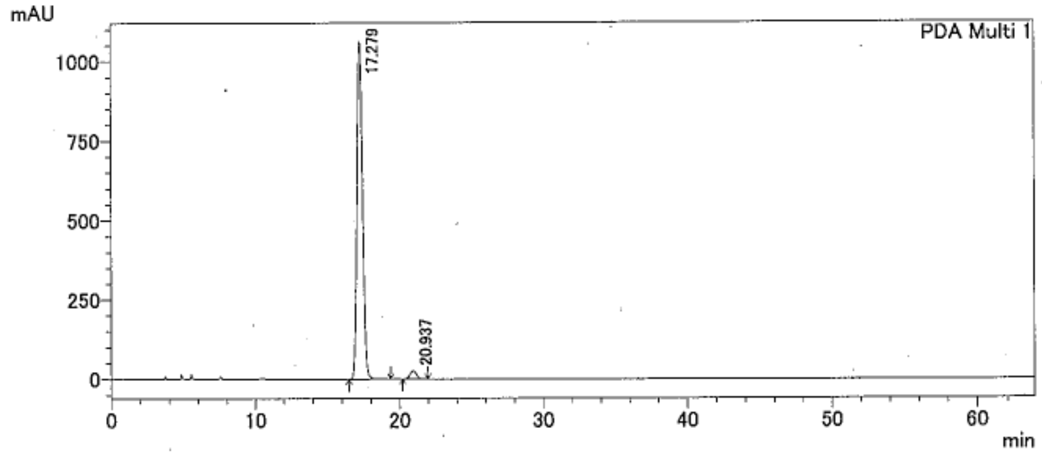

1 PDA Multi 1/220nm 4nm

ピークテーブル D:¥Nishino¥nsn646.lcd

PDA Ch1 220nm 4nm

ピーク#	保持時間	面積	高さ	面積%	高さ%
1	17.279	28757564	1061989	97.242	97.641
2	20.937	815648	25659	2.758	2.359
Total		29573213	1087647	100.000	100.000

ピーク ： **peak** 保持時間**:retention time** 面積**:area** 高さ**:height**

HPLC Table 9 entry 10

==== Shimadzu LCsolution 分析レポート ====

D:¥Nishino¥nsn653.lcd

分析者 : System Administrator
サンプル名 : nsn653
サンプルID : nsn653
測定条件 :OD-H, 1.0 mL/min, Hex:IPA = 1/1
注入量 : 1 uL
データファイル : nsn653.lcd
メソッドファイル : product.lcm

レポートファイル : Default.lcr
分析日時 : 2015/04/01 16:37:57
解析日時 : 2015/04/01 19:35:26

<クロマトグラム>

D:¥Nishino¥nsn653.lcd

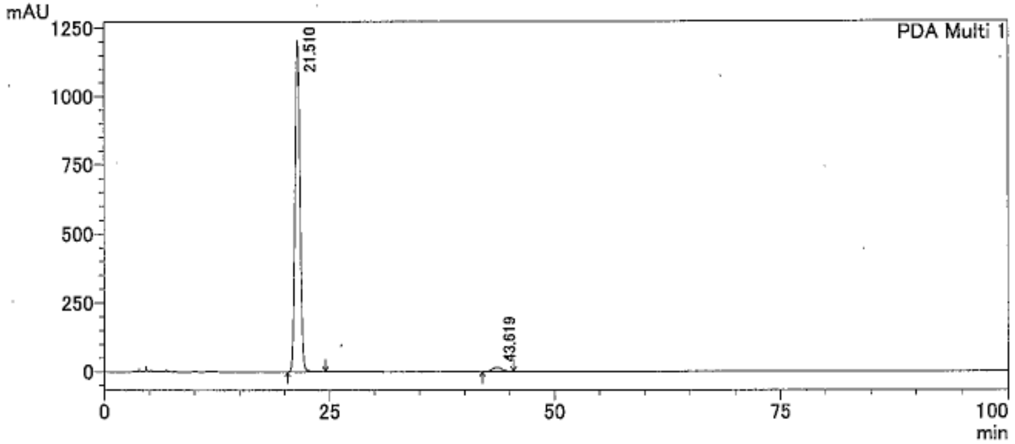

1 PDA Multi 1/220nm 4nm

ピークテーブル D:¥Nishino¥nsn653.lcd

PDA Ch1 220nm 4nm

ピーク#	保持時間	面積	高さ	面積%	高さ%
1	21.510	46372457	1204734	97.489	98.733
2	43.619	1194448	15465	2.511	1.267
Total		47566905	1220198	100.000	100.000

ピーク : **peak** 保持時間:**retention time** 面積:**area** 高さ:**height**

HPLC Table 9 entry 11

==== Shimadzu LCsolution 分析レポート ====

D:¥Nishino¥nsn664.lcd

分析者 : System Administrator
サンプル名 : nsn664
サンプルID : nsn664
測定条件 :OD-H, 1.0mL/min, Hex:IPA =3/2
注入量 : 1 uL
データファイル : nsn664.lcd
メソッドファイル : product.lcm

レポートファイル : Default.lcr
分析日時 : 2015/05/18 15:40:00
解析日時 : 2015/05/20 8:11:37

<クロマトグラム>

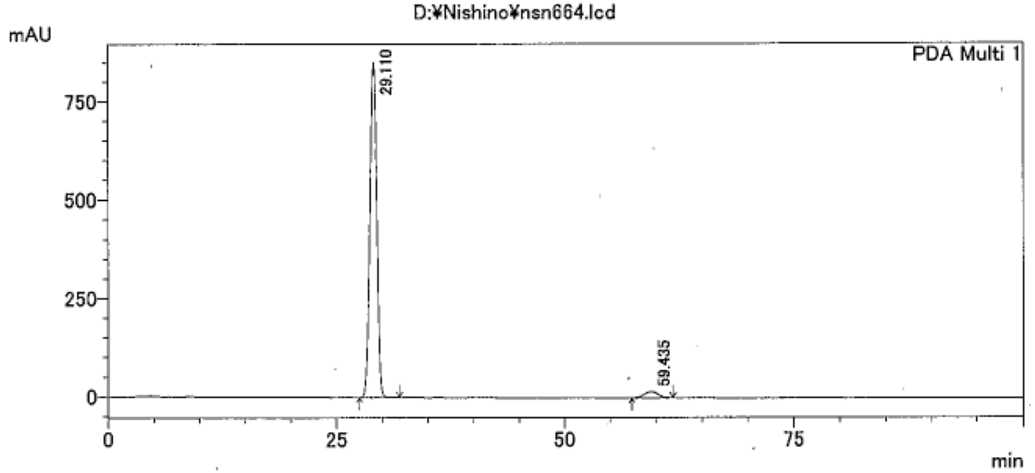

1 PDA Multi 1/220nm 4nm

ピークテーブル D:¥Nishino¥nsn664.lcd

PDA Ch1 220nm 4nm

ピーク#	保持時間	面積	高さ	面積%	高さ%
1	29.110	44842563	851629	96.597	98.221
2	59.435	1579681	15422	3.403	1.779
Total		46422243	867051	100.000	100.000

ピーク : **peak** 保持時間**:retention time** 面積**:area** 高さ**:height**

HPLC Table 9 entry 12

==== Shimadzu LCsolution 分析レポート ====

D:¥Nishino¥nsn649.lcd

分析者	: System Administrator
サンプル名	: nsn649
サンプルID	: nsn649
測定条件	:OD-H, 0.7mL/min, Hex:IPA =3/2
注入量	: 1 uL
データファイル	: nsn649.lcd
メソッドファイル	: product.lcm
レポートファイル	: Default.lcr
分析日時	: 2015/04/07 12:32:48
解析日時	: 2015/04/07 19:22:00

<クロマトグラム>

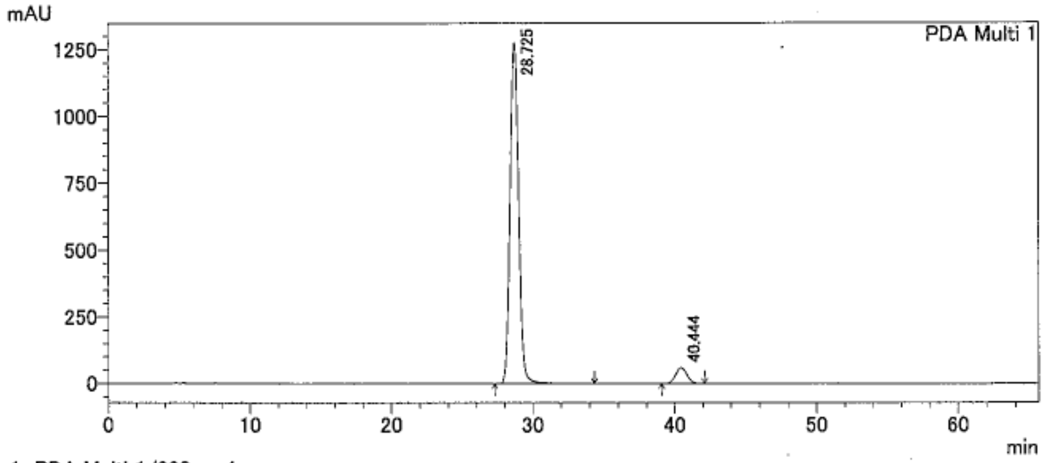

1 PDA Multi 1/220nm 4nm

ピークテーブル D:¥Nishino¥nsn649.lcd

PDA Ch1 220nm 4nm

ピーク#	保持時間	面積	高さ	面積%	高さ%
1	28.725	53931359	1276409	94.187	95.559
2	40.444	3328610	59315	5.813	4.441
Total		57259969	1335724	100.000	100.000

ピーク : **peak** 保持時間:**retention time** 面積:**area** 高さ:**height**

HPLC Table 9 entry 13

==== Shimadzu LCsolution 分析レポート ====

D:¥Nishino¥nsn655.lcd

分析者 : System Administrator
サンプル名 : nsn655
サンプルID : nsn655
測定条件 :OD-H, 1.0 mL/min, Hex:IPA = 1/1
注入量 : 1 uL
データファイル : nsn655.lcd
メソッドファイル : product.lcm

レポートファイル : Default.lcr
分析日時 : 2015/04/01 18:18:46
解析日時 : 2015/04/01 19:35:37

<クロマトグラム>

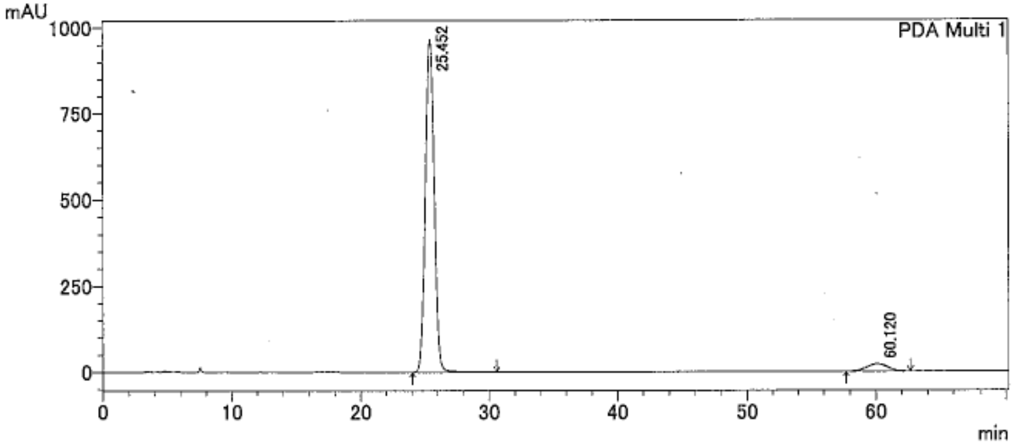

1 PDA Multi 1/220nm 4nm

ﾋﾟｰｸﾃｰﾌﾞﾙ D:¥Nishino¥nsn655.lcd

PDA Ch1 220nm 4nm

ピーク#	保持時間	面積	高さ	面積%	高さ%
1	25.452	45787805	966286	95.131	97.862
2	60.120	2343679	21114	4.869	2.138
Total		48131484	987399	100.000	100.000

ピーク : **peak** 保持時間**:retention time** 面積**:area** 高さ**:height**

HPLC Table 9 entry 14

==== Shimadzu LCsolution 分析レポート ====

D:¥Nishino¥nsn648.lcd

分析者　: System Administrator
サンプル名　: nsn648
サンプルID　: nsn648
測定条件　:OD-H, 1.0mL/min, Hex:IPA =3/2
注入量　: 1 uL
データファイル　: nsn648.lcd
メソッドファイル　: product.lcm

レポートファイル　: Default.lcr
分析日時　: 2015/04/07 15:29:21
解析日時　: 2015/04/07 19:22:26

<クロマトグラム>

D:¥Nishino¥nsn648.lcd

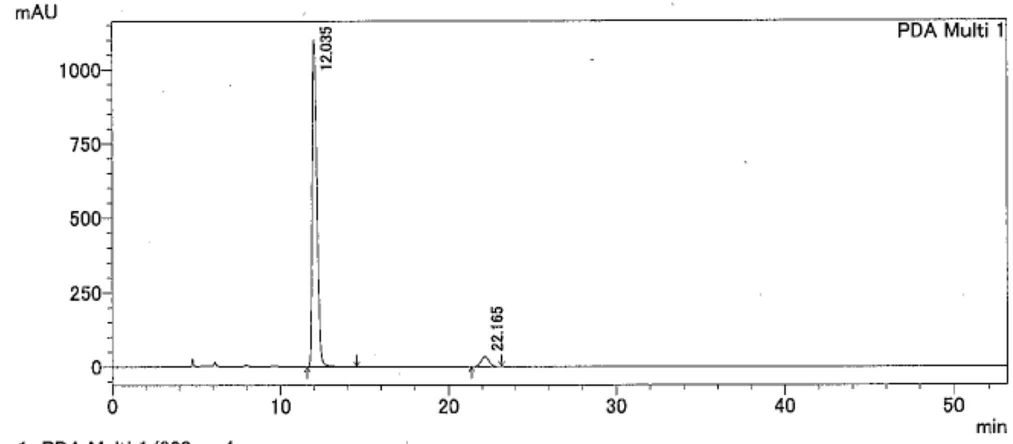

1 PDA Multi 1/220nm 4nm

ﾋﾟｰｸﾃｰﾌﾞﾙ D:¥Nishino¥nsn648.lcd

PDA Ch1 220nm 4nm

ピーク#	保持時間	面積	高さ	面積%	高さ%
1	12.035	21137616	1101097	94.881	96.949
2	22.165	1140398	34648	5.119	3.051
Total		22278014	1135744	100.000	100.000

ピーク ： **peak** 保持時間**:retention time** 面積**:area** 高さ**:height**

HPLC Table 9 entry 15

==== Shimadzu LCsolution 分析レポート ====

D:¥Nishino¥nsn656.lcd

分析者 : System Administrator
サンプル名 : nsn656
サンプルID : nsn656
測定条件 :OD-H, 1.0mL/min, Hex:IPA =3/2
注入量 : 1 uL
データファイル : nsn656.lcd
メソッドファイル : product.lcm

レポートファイル : Default.lcr
分析日時 : 2015/04/07 16:23:20
解析日時 : 2015/04/07 19:22:39

<クロマトグラム>

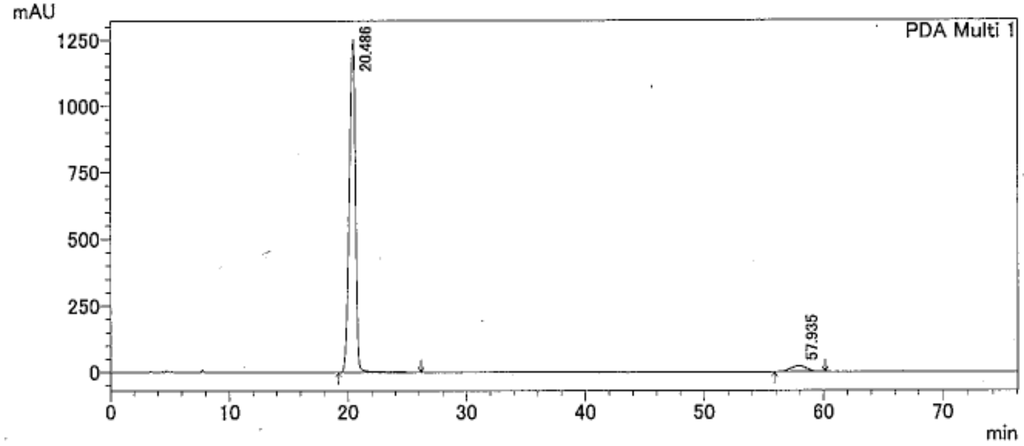

1 PDA Multi 1/220nm 4nm

ピークテーブル D:¥Nishino¥nsn656.lcd

PDA Ch1 220nm 4nm

ピーク#	保持時間	面積	高さ	面積%	高さ%
1	20.486	45629705	1251814	95.668	98.289
2	57.935	2066430	21790	4.332	1.711
Total		47696135	1273604	100.000	100.000

ピーク ： **peak** 保持時間**:retention time** 面積**:area** 高さ**:height**

HPLC Table 9 entry 16

==== Shimadzu LCsolution 分析レポート ====

D:¥Nishino¥nsn702.lcd

分析者 : System Administrator
サンプル名 : nsn702
サンプルID : nsn702
測定条件 :OD-H, 1.0mL/min, Hex:IPA =3/2
注入量 : 1 uL
データファイル : nsn702.lcd
メソッドファイル : product.lcm

レポートファイル : Default.lcr
分析日時 : 2015/05/22 17:29:52
解析日時 : 2015/05/27 8:31:12

<クロマトグラム>

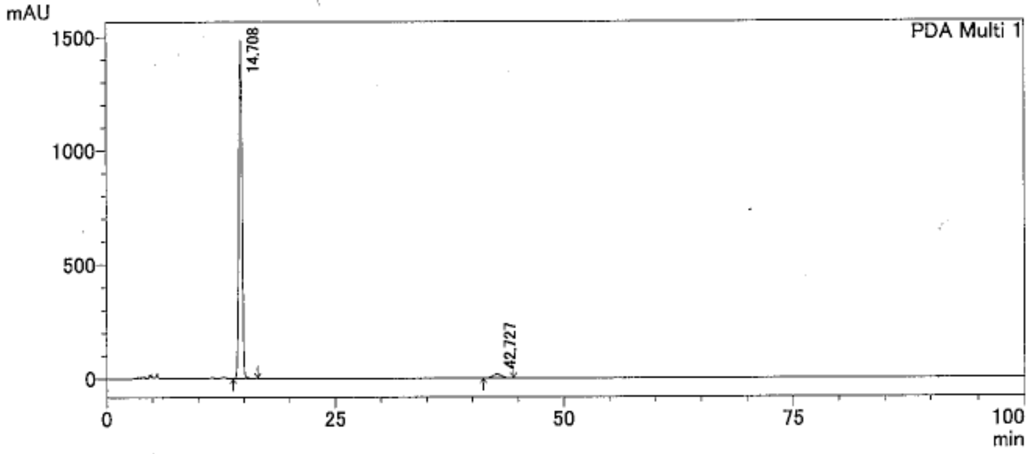

1 PDA Multi 1/220nm 4nm

ﾋﾟｰｸﾃｰﾌﾞﾙ D:¥Nishino¥nsn702.lcd

PDA Ch1 220nm 4nm

ピーク#	保持時間	面積	高さ	面積%	高さ%
1	14.708	36534446	1484737	97.043	98.909
2	42.727	1113117	16371	2.957	1.091
Total		37647563	1501108	100.000	100.000

ピーク：**peak** 保持時間:**retention time** 面積:**area** 高さ:**height**

HPLC Table 9 entry 17

==== Shimadzu LCsolution 分析レポート ====

D:¥Nishino¥nsn698.lcd

分析者 : System Administrator
サンプル名 : nsn698
サンプルID : nsn698
測定条件 :OD-H, 1.0mL/min, Hex:IPA =3/2
注入量 : 1 uL
データファイル : nsn698.lcd
メソッドファイル : product.lcm

レポートファイル : Default.lcr
分析日時 : 2015/05/20 17:27:50
解析日時 : 2015/05/21 15:15:34

<クロマトグラム>

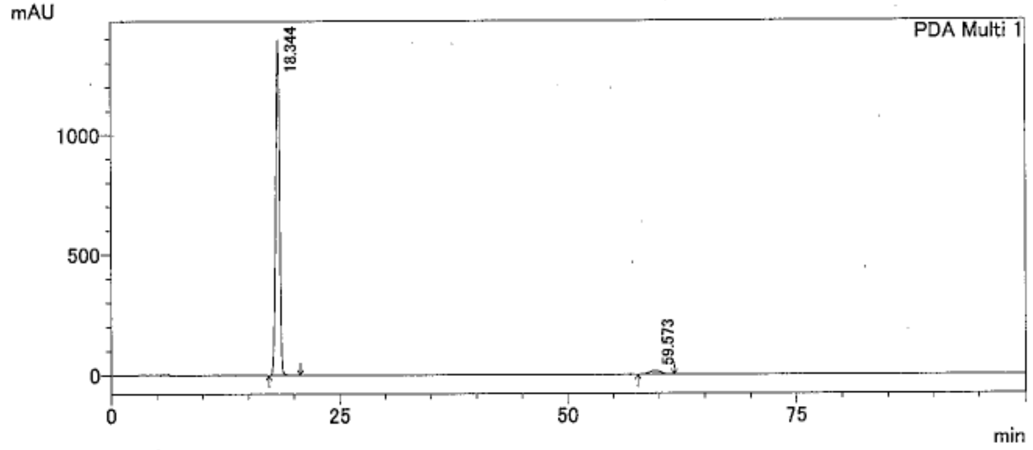

1 PDA Multi 1/220nm 4nm

ピークテーブル D:¥Nishino¥nsn698.lcd

PDA Ch1 220nm 4nm

ピーク#	保持時間	面積	高さ	面積%	高さ%
1	18.344	40796856	1395739	96.770	98.958
2	59.573	1361528	14702	3.230	1.042
Total		42158384	1410441	100.000	100.000

ピーク ： **peak** 保持時間**:retention time** 面積**:area** 高さ**:height**

HPLC Table 9 entry 18

==== Shimadzu LCsolution 分析レポート ====

D:¥Nishino¥nsn726.lcd

分析者 : System Administrator
サンプル名 : nsn726
サンプルID : nsn726
測定条件 :OD-H, 1.0mL/min, Hex:IPA =3/2
注入量 : 1 uL
データファイル : nsn726.lcd
メソッドファイル : product.lcm

レポートファイル : Default.lcr
分析日時 : 2015/06/13 16:28:39
解析日時 : 2015/06/16 18:31:14

<クロマトグラム>

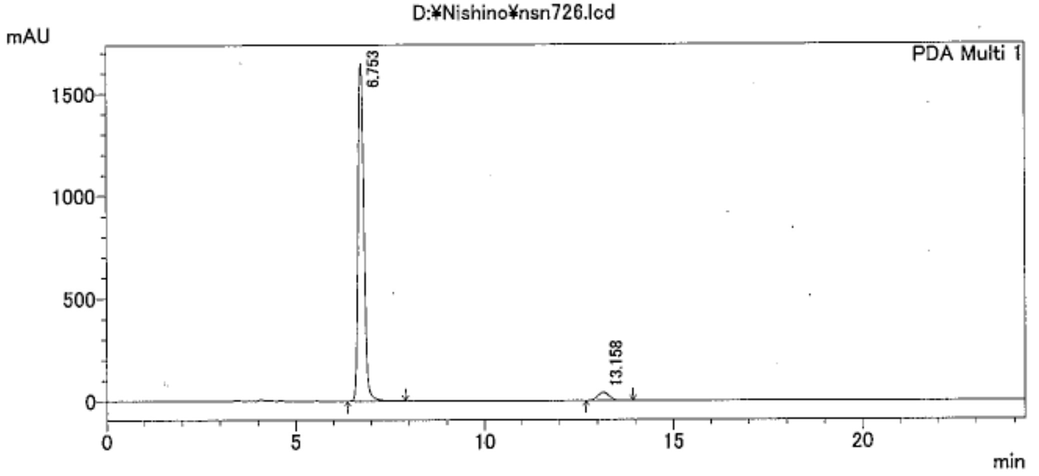

1 PDA Multi 1/220nm 4nm

ピークテーブル D:¥Nishino¥nsn726.lcd

PDA Ch1 220nm 4nm

ピーク#	保持時間	面積	高さ	面積%	高さ%
1	6.753	17963571	1646794	95.654	97.675
2	13.158	816074	39200	4.346	2.325
Total		18779645	1685994	100.000	100.000

ピーク : **peak** 保持時間**:retention time** 面積**:area** 高さ**:height**

HPLC Table 9 entry 19

==== Shimadzu LCsolution 分析レポート ====

D:¥Nishino¥nsn751.lcd

分析者 : System Administrator
サンプル名 : nsn751
サンプルID : nsn751
測定条件 :OD-H, 1.0mL/min, Hex:IPA =3/2
注入量 : 1 uL
データファイル : nsn751.lcd
メソッドファイル : product.lcm

レポートファイル : Default.lcr
分析日時 : 2015/07/09 16:27:45
解析日時 : 2015/07/09 17:43:23

<クロマトグラム>

D:¥Nishino¥nsn751.lcd

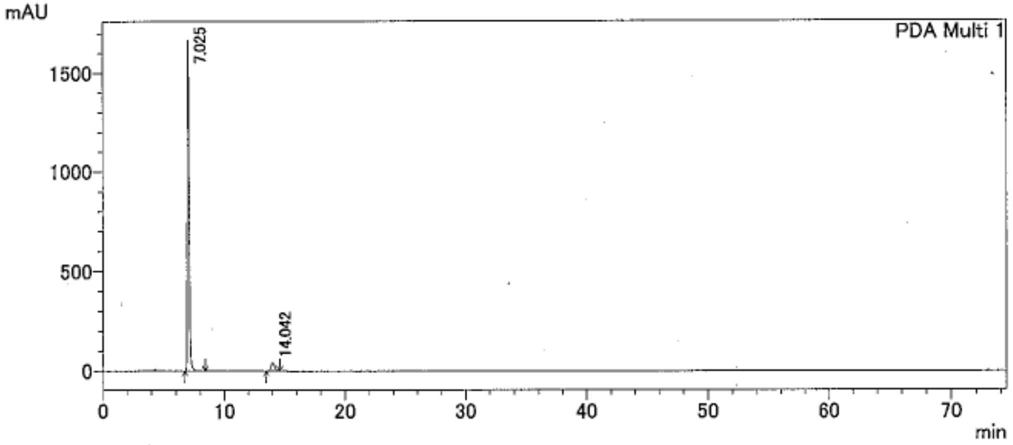

1 PDA Multi 1/220nm 4nm

ピークテーブル D:¥Nishino¥nsn751.lcd

PDA Ch1 220nm 4nm

ピーク#	保持時間	面積	高さ	面積%	高さ%
1	7.025	18135858	1667997	95.244	97.464
2	14.042	905519	43406	4.756	2.536
Total		19041376	1711403	100.000	100.000

ピーク：**peak** 保持時間**:retention time** 面積**:area** 高さ**:height**

HPLC Table 9 entry 20

==== Shimadzu LCsolution 分析レポート ====

D:¥Nishino¥nsn731.lcd

分析者 : System Administrator
サンプル名 : nsn731
サンプルID : nsn731
測定条件 :OD-H, 1.0mL/min, Hex:IPA =99/1
注入量 : 1 uL
データファイル : nsn731.lcd
メソッドファイル : product.lcm

レポートファイル : Default.lcr
分析日時 : 2015/06/15 11:07:00
解析日時 : 2015/06/16 18:32:27

<クロマトグラム>

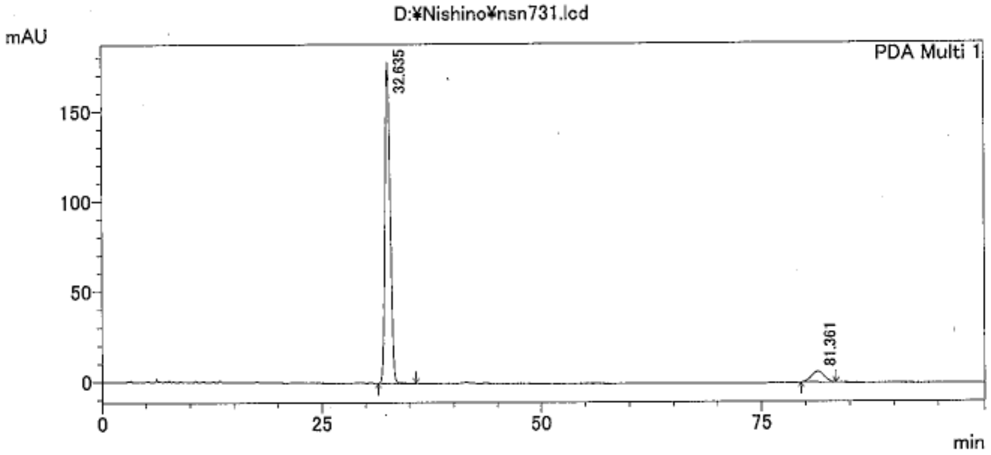

1 PDA Multi 1/220nm 4nm

ピークテーブル D:¥Nishino¥nsn731.lcd

PDA Ch1 220nm 4nm

ピーク#	保持時間	面積	高さ	面積%	高さ%
1	32.635	7489747	178132	92.728	96.816
2	81.361	587349	5858	7.272	3.184
Total		8077096	183990	100.000	100.000

ピーク ： **peak 保持時間:retention time 面積:area 高さ:height**

9. References

[1] Brown, H. C.; Mead, E. J.; Rao, B. C. S. *J. Am. Chem. Soc.* **1955**, *77*, 6209.
[2] (a) Okamoto, K.; Hayashi, T.; Rawal, V. H. *Chem. Commun.* **2009**, 4815; (b) Okamoto, K.; Hayashi, T.; Rawal, V. H. *Org. Lett.* **2008**, *10*, 4387.
[3] Yasukawa, T.; Miyamura, H.; Kobayashi, S. *J. Am. Chem. Soc.* **2012**, *134*, 16963.
[4] Bowman, R. K.; Johnson, J. S., *The Journal of Organic Chemistry* **2004**, *69*, 8537.
[5] Lin, L.; Yin, W.; Fu, X.; Zhang, J.; Ma, X.; Wang, R., *Organic & Biomolecular Chemistry* **2012**, *10*, 83.
[6] Kiyokawa, K.; Nagata, T.; Hayakawa, J.; Minakata, S., *Chemistry – A European Journal* **2015**, *21*, 1280.
[7] Chandrasekhar, S.; Shrinidhi, A., *Synthetic Communications* **2014**, *44*, 3008.
[8] Antoft-Finch, A.; Blackburn, T.; Snieckus, V., *J Am Chem Soc* **2009**, *131*, 17750.
[9] Gao, J.-R.; Wu, H.; Xiang, B.; Yu, W.-B.; Han, L.; Jia, Y.-X., *J Am Chem Soc* **2013**, *135*, 2983.
[10] Xing, J.; Chen, G.; Cao, P.; Liao, J., *European Journal of Organic Chemistry* **2012**, 1230.
